# Overcoming Challenges in Small‐Ring Transfer: Direct Decarboxylative Hydroalkylation of Alkenes via Iron‐Thiol Catalysis

**DOI:** 10.1002/anie.202508377

**Published:** 2025-07-07

**Authors:** Rahul Giri, Po‐Kai Peng, Anthony J. Fernandes, Shijin Yu, Julian G. West, Dmitry Katayev

**Affiliations:** ^1^ Department of Chemistry, Biochemistry and Pharmaceutical Sciences University of Bern Freiestrasse 3 Bern 3012 Switzerland; ^2^ Department of Chemistry Rice University 6100 Main St Houston Texas 77005 USA

**Keywords:** Photocatalysis, Ligand‐to‐metal charge transfer (LMCT), Iron catalysis, Fluorinated radicals, Density functional theory

## Abstract

Cyclopropanes, especially those substituted with trifluoromethyl (CF_3_) groups, are valuable scaffolds in medicinal chemistry. Their enhanced bioavailability contributes to the widespread presence of this motif in a variety of bioactive compounds. Despite the development of multiple synthetic strategies, a direct method for transferring CF_3_‐containing small rings from their most abundant carboxylic acid surrogates remains a significant challenge. In this work, we overcome the challenging decarboxylation of CF_3_‐cyclopropyl and CF_3_‐cyclobutyl carboxylic acids while leveraging the ligand‐to‐metal charge transfer (LMCT) excited state of photochemically activated iron complexes. The resulting radicals were then engaged in radical addition to alkenes, followed by a timely hydrogen atom transfer (HAT) process mediated by a thiol donor. This efficient iron‐thiol cooperative catalysis enables, for the first time, facile hydroalkylation of alkenes with highly reactive CF_3_‐containing cyclopropyl and cyclobutyl radicals. Synergistic experimental, spectroscopic, and density functional theory (DFT) studies support the proposed reaction mechanism. Additionally, we employed the radical stabilization energy (RSE) scale, developed using isodesmic equations computed at the DFT level, aiming to provide a better understanding of the reluctant radical decarboxylation of small‐strained cycloalkanes.

## Graphical abstract

Herein, we present a catalytic approach for the direct decarboxylation of challenging CF_3_‐cyclopropyl and cyclobutyl carboxylic acids using cooperative iron‐thiol catalysis. This strategy enables the efficient generation of valuable radicals that can be incorporated into activated and unactivated alkenes, offering access to elusive chemical space. Furthermore, we provide comprehensive mechanistic studies, supported by experimental, spectroscopic, and DFT data, which offer insights into radical stability in small‐ring systems.

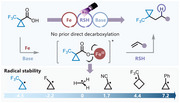

## Introduction

**Figure 1:**
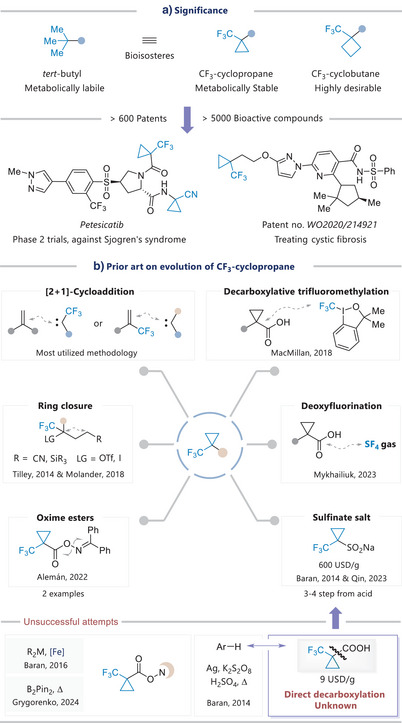
Introduction: a) Importance of the trifluoromethyl‐substituted small ring systems. b) Evolution of CF_3_‐cyclopropane synthesis.

Cyclopropane is the smallest cyclic structure in organic chemistry. Although ideal bond angles for sp^3^‐hybridized carbon atoms are expected to be 109.5°, those in cyclopropane adopt highly strained 60° angles.^[^ ^1^, ^2^, ^3^ ^]^ Appending a cyclopropyl ring with a trifluoromethyl (CF_3_) substituent is regarded as a bioisostere of the *tert*‐butyl group, with analogous properties also exhibited by CF_3_‐cyclobutane (Figure 1a).^[^ ^4^, ^5^, ^6^, ^7^ ^]^ Both CF_3_ cyclopropane and cyclobutane are privileged scaffolds, featured in over 5000 bioactive compounds and widely studied in the literature due to their presence in several drugs, including those undergoing clinical trials.^[^ ^4^, ^8^, ^9^, ^10^, ^11^, ^12^ ^]^ Their importance is further highlighted by the presence of these motifs in over 600 patents across biology and chemistry.^[^ ^13^, ^14^ ^]^

Trifluoromethylated small rings are in high demand due to their highlighted properties. In recent years, there has been a surge in methods to synthesize these molecules, with one popular strategy involving CF_3_ carbene intermediates in [2 + 1] cycloaddition reactions (Figure 1b). This elegant approach typically employs 2,2,2‐trifluorodiazoethane (CF₃CHN₂) and other carbene surrogates.^[^ ^15^ ^]^ Another complementary strategy for accessing these valuable compounds relies on the reaction of carbenes with CF₃‐substituted olefins.^[^ ^16^ ^]^ Despite its utility, the practicality of this approach is limited by challenges such as a step‐intensive synthesis, reliance on costly metals (e.g., Rh, Pd), and the general difficulties associated with handling carbenes.^[^ ^17^, ^18^ ^]^ A distinct approach employs ring closure of pre‐functionalized precursors, achieving the final cyclopropanes through the action of bases or silylium cations.^[^ ^19^, ^20^, ^21^, ^22^ ^]^ The MacMillan group employed a decarboxylative trifluoromethylation strategy to synthesize CF_3_‐cyclopropanes from carboxylic acids and Togni's reagent.^[^ ^23^ ^]^ Innovative methods have been reported by the Mykhailiuk group at Enamine for synthesizing CF_3_‐cyclopropanes and CF₃‐cyclobutanes, enhancing the accessibility of these highly sought‐after molecules. Their approach utilizes the deoxyfluorination of carboxylic acids with sulfur tetrafluoride (SF_4_).^[^ ^10^, ^13^ ^]^ However, a critical limitation of this chemistry lies in the use of the gaseous and extremely toxic SF_4,_ which requires specialized handling procedures and, notably, has a historical association with its use as a chemical warfare agent.^[^ ^24^ ^]^ Recently, Alemán and coworkers described the functionalization of acrylates in photo‐flow conditions using pre‐functionalized oxime esters to obtain *α*‐amino carbonyl adorned by CF_3_‐cyclopropanes.^[^ ^25^ ^]^ The synthesis of CF_3_‐cyclopropanes can also be accomplished using Baran's sodium 1‐(trifluoromethyl)cyclopropane sulfinate (TFCS‐Na) reagent. This reagent has been successfully applied for C–H functionalization of heteroarenes, albeit requiring super‐stoichiometric oxidants and elevated temperatures.^[^ ^26^ ^]^ Subsequently, the Qin group employed TFCS‐Na to functionalize a variety of heterocycles through an ionic sulfurane‐mediated C(sp^3^)–C(sp^2^) cross‐coupling enabled by the addition of Grignard reagents at −78 °C.^[^ ^27^ ^]^ While these two methods allowed the installation of the CF_3_‐cyclopropyl moiety on various heteroarenes, they remain severely limited to Minisci‐type reaction products. Furthermore, the TFCS‐Na reagent is extremely expensive (600 US$ g^−1^) because of its multistep synthesis starting from readily available 1‐(trifluoromethyl)cyclopropane‐1‐carboxylic acid (9 US$ g^−1^).

In this context, developing a novel strategy that bypasses the need for sulfinate salts by directly activating carboxylic acid precursors appears highly desirable. Indeed, the direct decarboxylation of commercially available acid emerges as a general method enabling efficient, cost‐effective, and step‐economical transformations for synthetic applications. However, despite notable efforts by Baran^[^ ^26^, ^28^ ^]^ and Grygorenko^[^ ^29^ ^]^ groups, no direct method for accessing CF_3_‐substituted cyclopropanes from cyclopropyl carboxylic acids has been developed.

Recent advances in light‐mediated iron‐catalyzed reactions^[^ ^30^, ^31^, ^32^, ^33^, ^34^, ^35^, ^36^ ^]^ have led to the development of innovative strategies for transferring valuable radicals via direct decarboxylation.^[^ ^37^, ^38^, ^39^, ^40^, ^41^, ^42^, ^43^, ^44^ ^]^ Notably, the West group introduced a method that utilizes iron catalysis to efficiently decarboxylate trifluoroacetic acid (TFA) under visible‐light irradiation, thereby generating trifluoromethyl radicals.^[^ ^45^ ^]^ This process involves in situ pre‐association of the iron metal center with the deprotonated trifluoromethyl carboxylate anion, followed by a light‐driven ligand‐to‐metal charge transfer (LMCT).^[^ ^35^, ^46^ ^]^ This approach leverages the unique reactivity of iron‐(III) salts under purple‐light irradiation to overcome the significant challenge posed by the high redox potential of TFA (*E*_red_ ca. 2.4 V).^[^ ^40^, ^45^ ^]^ When combined with a hydrogen atom transfer (HAT) catalyst serving as the hydrogen source, the method has been effectively employed for the hydro‐trifluoromethylation of a range of olefins.^[^ ^47^, ^48^, ^49^, ^50^ ^]^ Building on this work, we envisioned that this powerful concept could solve the challenging decarboxylation of cyclopropyl derivatives, in particular that of the 1‐(trifluoromethyl)‐cyclopropane‐1‐carboxylic acid, which has proven remarkably difficult to achieve under state‐of‐the‐art protocols (yields < 5%).^[^ ^26^, ^28^, ^29^, ^51^ ^]^

Herein, we report that applying in situ‐generated photoresponsive iron complexes successfully overcomes the challenging decarboxylation of high‐value cyclopropyl and cyclobutyl carboxylic acids. This reaction paradigm provides access to a broad chemical space, accommodating activated and unactivated olefins. Mechanistic investigation of the reaction through experimental, spectroscopic, and DFT studies supported our proposed radical mechanism.

## Results and Discussion

### Reaction Development

At the onset of this study, we aimed to understand the origin of the trifluoromethyl cyclopropane carboxylic acid's lack of reactivity in decarboxylation methodologies. Initial calculations of redox potentials suggested that, in contrast to TFA (*E*_red,calc_(CF_3_CO_2_^−^) = 2.5 V), this acid should readily undergo oxidative decarboxylation with estimated *E*_red,calc_(CF_3_cpCO_2_^−^) of 1.6 V. Consequently, we computed the radical decarboxylation step, which confirmed that it is indeed more difficult than for TFA (Δ*G* ^‡^ = 0.3 and Δ_r_ *G* = −24.0, in kcal mol^−1^), with both an increased activation barrier and reduced exergonicity (Δ*G* ^‡^ = 0.8 and Δ_r_ *G* = −14.0, in kcal mol^−1^). The rationale for this behavior likely lies in the geometry of the produced radical following decarboxylation. Furthermore, the geometry of the trifluoromethylcyclopropyl radical species exhibits a strong pyramidalization (*θ*_p_ = 20.2°) with an overall calculated hybridization of sp^3.1^, substantially higher than that of the CF_3_ radical.^[^ ^52^, ^53^ ^]^ This geometry results in poor stabilization of the pronounced σ‐radical by neighboring substituents, which accounts for its reluctant formation through decarboxylation, as observed experimentally. Recent research indicates that the effectiveness of LMCT‐mediated decarboxylation relies on the stability of the produced radical.^[^ ^34^, ^54^ ^]^

**Figure 2:**
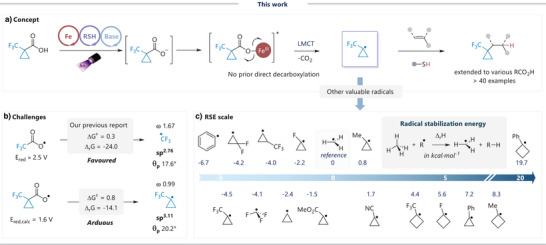
a) Concept of the direct decarboxylation of 1‐(trifluoromethyl)cyclopropane‐1‐carboxylic acid for alkene hydroalkylation; b) Challenges in the decarboxylation process; c) DFT‐based RSE scale computed at the (U)M062X‐D3/def2‐TZVP, SMD(MeCN)//(U)M062X‐D3/def2‐SVP, SMD(MeCN) level of theory, selected examples (see Supporting Information); *ω* = Global electrophilicity index.

To better understand the effect of substituent and ring strain on the decarboxylation, we therefore computed the radical stabilization energy (RSE) of relevant radical species using an isodesmic equation, expressed as the enthalpic energy resulting from the H atom transfer from CH_4_ to R^•^ radical (Figure 2; see Supporting Information for further details). On this scale, a radical described with a negative (or positive) value is less (or more) stable than the methyl radical, which is taken as a reference, and indicates a more (or less) challenging decarboxylation compared to the acetoxy radical.

The resulting scale reveals that the “naked” cyclopropyl radical is one of the most unstable species among those considered (Δ_r_ *H* = −2.4 kcal mol^−1^), close to the phenyl radical (Δ_r_ *H* = −6.7 kcal mol^−1^). The incorporation of a fluorine, ester, or methyl substituent tends to stabilize the radical, with Δ_r_ *H* values of −2.2, −1.5, and 0.8 kcal mol^−1^ for the F‐, CO_2_Me‐, and Me‐cyclopropyl radicals, respectively. It is worth noting that the CF_3_ substituent destabilizes the cyclopropyl radical, whether located at the *α* or *β* position of the radical center, with Δ_r_ *H* = −4.5 and −4.0 kcal mol^−1^, respectively. Beyond electronic effects, ring strain also plays a critical role in the stability of the radical, and cyclobutyl derivatives are substantially more stable. For example, the CF_3_‐cyclobutyl radical is significantly stabilized (Δ_r_ *H* = 4.4 kcal mol^−1^) compared to its cyclopropyl counterpart (Δ_r_ *H* = −4.5 kcal mol^−1^). The decarboxylation of the trifluoroacetoxy radical, which leads to the similarly unstable •CF_3_, is also difficult (Δ_r_ *H* = −4.1 kcal mol^−1^). Despite confirming the challenges related to the envisioned decarboxylation, these calculations collectively demonstrate that it remains a thermodynamically favorable process that could become kinetically accessible once suitable reaction conditions are identified.

We, thus, initiated the optimization of decarboxylation for the target compound, 1‐(trifluoromethyl)cyclopropane‐1‐carboxylic acid (**B**), employing a cooperative system comprising an earth‐abundant iron complex (Fe(NO_3_)_3_·9H_2_O), 2,4,6‐triisopropylbenzenethiol (TRIP thiol) as a redox‐active thiol, and sodium carbonate as the base. For the model olefin substrate, we chose pent‐4‐en‐1‐yl benzoate. After evaluating various conditions, the combination of catalytic iron(III) (20 mol%), TRIP thiol (20 mol%), and base (20 mol%) achieved the highest efficiency, yielding 76% conversion to the hydro‐functionalized product (Table 1, entry 1). This method notably bypassed the need for preactivation of the acid, showcasing a direct and efficient pathway for accessing trifluoromethyl‐substituted cyclopropane derivatives. We also examined the organophotocatalyzed method by Kim (4CzIPN),^[^ ^55^ ^]^ Yang's iodine(III)‐mediated strategy,^[^ ^56^ ^]^ and the use of the phthalimide ester of **B** under photoredox catalysis, among others (entries 2–5). Unfortunately, these attempts did not produce the desired product, instead resulting in either complicated mixtures or the recovery of unreacted starting material. Adjustments to the standard conditions revealed the critical importance of each reaction component in optimizing the reaction's efficiency. The use of MeCN as a solvent proved superior to DMF (entry 6), and alternative iron catalysts were less effective compared to Fe(NO₃)₃·9H₂O, highlighting the latter's unique compatibility with the reaction system (entry 7). Similarly, TRIP thiol demonstrated significantly greater activity than comparable thiols (entries 8 and 9). While other bases yielded moderate results, they proved less effective than Na_2_CO_3_ in promoting the reaction (entries 10 and 11). Reducing loadings to 10 mol% of each reaction component (Fe(NO_3_)_3_·9H_2_O, TRIP thiol, and Na_2_CO_3_) resulted in a slight decrease in reaction efficiency (entry 12). Additional screening revealed that 20 mol% loading conditions were more consistent and reliable compared to 10% and were therefore implemented as the standard reaction conditions.

**Table 1:** Reaction development.

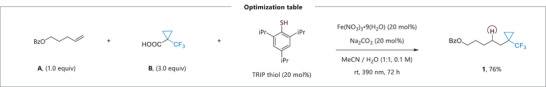			
Entry^a)^	Deviation from above	1 (%)^b)^	
1	No deviation	76 (72%)^c)^	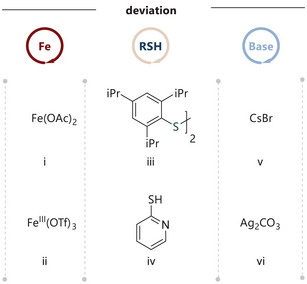
2^d)^	West conditions (**i** as iron source)	34	
3	Kim conditions^e)^	<5	
4	Yang conditions^e)^	<5	
5	Phthalimide ester of **B** ^e)^	<5	
6^d)^	DMF instead of MeCN	48	
7^d)^	**ii** as iron source	25	
8^d)^	**iii** instead of TRIP thiol	52	
9^d)^	**iv** instead of TRIP thiol	0	
10^d)^	**v** instead of Na_2_CO_3_	3	
11^d)^	**vi** instead of Na_2_CO_3_	24	
12^f)^	10 mol% of Fe(NO_3_)_3_·9H_2_O, TRIP thiol, Na_2_CO_3_	72	

^a)^ Reaction conditions: alkene (0.1 mmol, 1.0 equiv), acid B (3.0 equiv), Fe(NO_3_)_3_·9H_2_O, (20 mol%), TRIP thiol (20 mol%), Na_2_CO_3_ (20 mol%), and MeCN: H_2_O (0.1 M), 72 h, rt, 390 nm Kessil lamp (100% intensity).

^b)^ Yields are determined by ¹⁹F NMR with PhCF₃ serving as an internal standard.

^c)^ Isolated yield of compound **1**.

^d)^ Using 10 mol% of the iron source, 10 mol% of the HAT source, and 10 mol% of the base, with a reaction time of 48 h.

^e)^ The details of these reaction conditions are provided in the Supporting Information, page S8.

^f)^ 10 mol% of each reaction component was used.

### Scope of the Transformation

**Figure 3:**
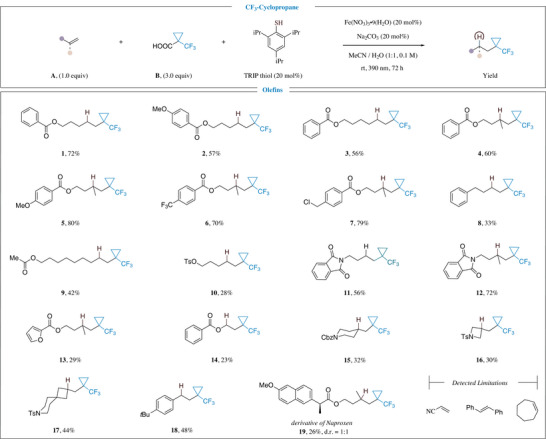
Substrate scope for the transfer of trifluoromethyl‐substituted cyclopropanes. General conditions: alkene (0.1 mmol, 1.0 equiv), acid **B** (3.0 equiv), Fe(NO_3_)_3_·9H_2_O, (20 mol%), TRIP thiol (20 mol%), Na_2_CO_3_ (20 mol%), and MeCN:H_2_O (1:1, 0.1 M), 72 h, rt, 390 nm Kessil lamp (100% intensity). The yields of isolated compounds are reported.Substrate scope for the transfer of trifluoromethyl‐substituted cyclopropanes. General conditions: alkene (0.1 mmol, 1.0 equiv), acid **B** (3.0 equiv), Fe(NO_3_)_3_·9H_2_O, (20 mol%), TRIP thiol (20 mol%), Na_2_CO_3_ (20 mol%), and MeCN:H_2_O (1:1, 0.1 M), 72 h, rt, 390 nm Kessil lamp (100% intensity). The yields of isolated compounds are reported.

After optimizing the reaction conditions, we examined the scope and limitations of this hydro(trifluoromethyl)‐cyclopropylation protocol with respect to various olefin substrates (Figure 3). The methodology is effective with simple unactivated alkenes, as demonstrated by benzoate‐containing alkenes with linear four‐ to six‐carbon chains, which efficiently afford the desired products (**1**–**7**). Functional group tolerance is notable in substrates bearing electron‐donating (methoxy) (**2**, **5**) and electron‐withdrawing (trifluoromethyl) (**6**) aromatic substitution. Furthermore, the reaction conditions also accommodate sensitive groups, such as benzyl chloride (**7**), emphasizing the method's compatibility with diverse electronic environments. Disubstituted olefins react smoothly to produce **4**–**7** in good yields, while simple olefins, such as phenylbutene, also yield adduct in respectable amounts (**8**). Long‐chain esters are compatible with the reaction conditions, yielding products (**9**). The reaction exhibits compatibility with common protecting groups in organic synthesis, including tosyl‐protected alcohol (**10**) and phthalimide‐masked amines (**11**, **12**). Furthermore, the methodology extends to structurally diverse and sensitive substrates, including furan heterocycle (**13**) and vinyl benzoate (**14**). Even substrates with steric hindrance demonstrate a noteworthy level of reactivity in this process. For instance, nitrogen‐containing cyclic and spirocyclic compounds, including six‐ and four‐membered rings with an exocyclic double bond, efficiently yielded products **15**–**17**. Styrenes also displayed moderate reactivity under these conditions (**18**), while naphthyl‐containing Naproxen derivative underwent smooth transformations to deliver the product **19**. This approach is notable for constructing molecules enriched with sp^3^‐hybridized carbon centers, adding structural complexity and three‐dimensionality to the resulting frameworks. Although the yields of certain substrates are low, it is primarily due to unreacted starting material affecting the mass balance, despite an extended reaction time.

The established reaction conditions failed to yield the desired product with electron‐deficient or internal alkenes such as acrylonitrile and *trans*‐stilbene, as the starting olefin remained unreacted. For cycloheptene, the main side product resulted from thiol addition to the alkene. These limitations likely stem from a combination of slower radical addition and the inefficient decarboxylation of acid **B**. Additionally, competing and unproductive reaction pathways, such as HAT between the carboxyl radical and the substrate, which regenerate acid **B** while forming a new radical that is later reduced by the thiol to reform the substrate, cannot be ruled out. Nevertheless, this methodology is highly valuable, as it enables the synthesis of challenging linear CF₃‐cyclopropanes (Figure 3), which are otherwise complex, or in some cases elusive, to access through a one‐step protocol.

After we investigated CF₃‐cyclopropane transfer to olefins, we explored this strategy for the hydroalkylation of alkenes using different substituted cyclopropyl groups, which are similarly challenging to introduce via their carboxylic acid precursors. Among these, CN‐cyclopropane is particularly interesting due to its versatility as a synthetic handle, as it can be readily converted into amines, carboxylic acids, or amides.

**Figure 4:**
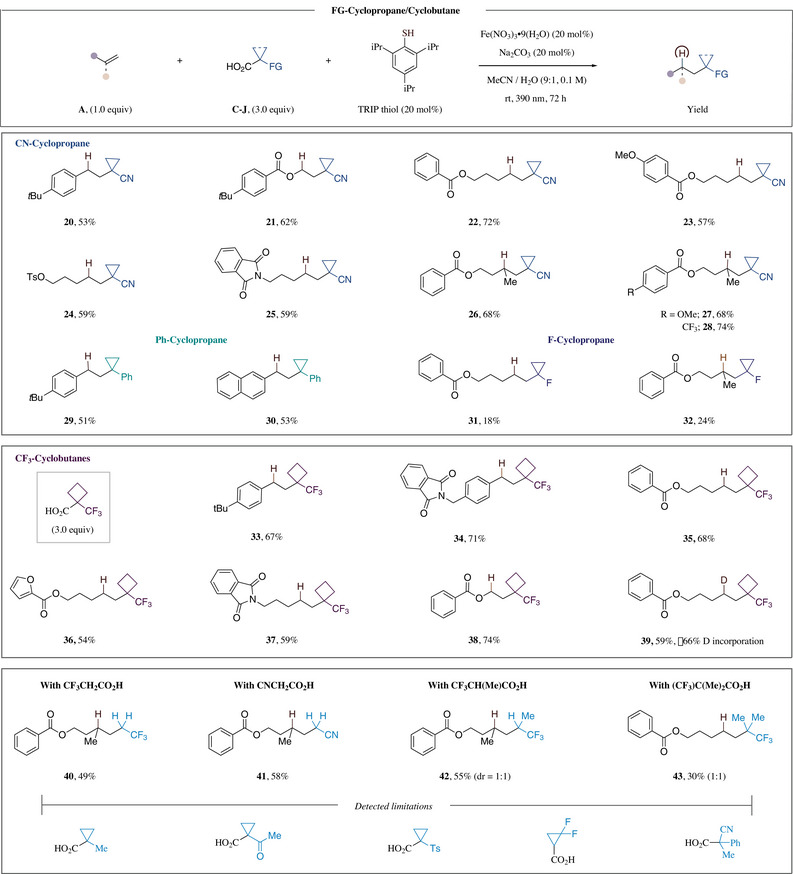
Substrate scope for the transfer of substituted cyclopropanes. General conditions: alkene (0.1 mmol, 1.0 equiv), acids **C–J** (3.0 equiv), Fe(NO_3_)_3_·9H_2_O, (20 mol%), TRIP thiol (20 mol%), Na_2_CO_3_ (20 mol%), and MeCN:H_2_O (9:1, 0.1 M), 72 h, rt, 390 nm Kessil lamp (100% intensity). The yields of isolated compounds are reported.Substrate scope for the transfer of substituted cyclopropanes. General conditions: alkene (0.1 mmol, 1.0 equiv), acids **C–J** (3.0 equiv), Fe(NO_3_)_3_·9H_2_O, (20 mol%), TRIP thiol (20 mol%), Na_2_CO_3_ (20 mol%), and MeCN:H_2_O (9:1, 0.1 M), 72 h, rt, 390 nm Kessil lamp (100% intensity). The yields of isolated compounds are reported.

Short reoptimization revealed that adjusting the solvent ratio to 9:1 MeCN/H₂O improved the reaction yield, likely due to enhanced solubility of this acid in the solvent mixture (Figure 4). In good agreement with the higher computed RSE value—and therefore facilitated decarboxylation from the carboxyl radical precursor—for this radical (Figure 2, Δ_r_ *H*(CNcp^•^) = 1.7 kcal mol^−1^), the reaction provided the corresponding product in good to high amounts.

Styrenes were found to be suitable substrates for this process, leading to the formation of the hydro‐cyanocyclopropylated product (**20**). Similarly, vinyl benzoate (**21**), long‐chain alkenes (**22**, **23**), protected alcohol and amine (**24**, **25**), and disubstituted alkenes (**26**–**28**) all underwent smooth transformation under the reaction conditions.

Phenyl‐substituted cyclopropane carboxylic acid could also be decarboxylated (Figure 2, Δ_r_ *H*(Phcp^•^) = 7.2 kcal mol^−1^) under the reaction conditions, thereby converting styrenyl substrates into their corresponding products **29** and **30**. The by‐products of the reaction include the substitution of one of the vinyl C─H bonds with a phenyl cyclopropyl group and the unreacted starting material olefin. The methodology was further applied to fluorine‐containing cyclopropanes, which are particularly intriguing yet challenging to synthesize using reported approaches. Under the current conditions, the reaction proceeded with relatively low efficiency yet still provided access to the elusive products from both mono‐ and bis‐substituted olefins in a single step (**31** and **32**, respectively). We experimentally observed a very slow decarboxylation of the acid precursor in these cases, with the mass balance consisting essentially of unreacted starting material, consistent with the low stability computed for this radical (Figure 2, Δ_r_ *H*(Fcp^•^) = −2.2 kcal mol^−1^).

The next challenge involved the transfer of CF₃‐cyclobutane to olefins (Δ_r_ *H*(CF_3_cbut^•^) = 4.4 kcal mol^−1^). Under standard conditions, this transformation proceeded efficiently and applied to both styrene and nonactivated terminal olefins. The reaction exhibited broad compatibility, yielding products in good yields. Styrenes substituted with *t*Bu or phthalimide groups afforded products **33** and **34**, respectively. Additionally, the reaction conditions smoothly converted substrates such as benzoates (**35**, **36**, **38**, and **39**), furan (**36**), and phthalimide‐derived olefins (**37**) into the desired products. Using D₂O as a co‐solvent instead of H₂O resulted in 66% deuterium incorporation, allowing us to synthesize compounds with isotopic labeling (**39**). To evaluate the protocol's generality beyond tertiary cyclic acids, we tested noncyclic primary, secondary, and tertiary acids. Trifluoropropanoic and 2‐cyanoacetic acids reacted with 3‐methylbut‐3‐en‐1‐yl benzoate, yielding products **40** and **41**, respectively. The secondary trifluoro‐2‐methylpropanoic acid furnished product **42** in decent yield, while the tertiary acid trifluoro‐2,2‐dimethylpropanoic acid gave product **43** in moderate yield. These results highlight the versatility and robustness of this method across diverse substrates. Despite its demonstrated generality, our developed protocol still has limitations in the decarboxylation leading to highly unstable radicals such as the difluorocyclopropyl radical (Δ_r_ *H*(*β*F_2_cp^•^) = −4.2 kcal mol^−1^), or methylcyclopropyl radical (Δ_r_ *H*(CH_3_cp^•^) = 0.8 kcal mol^−1^), among others.

### Proposed Mechanism

**Figure 5:**
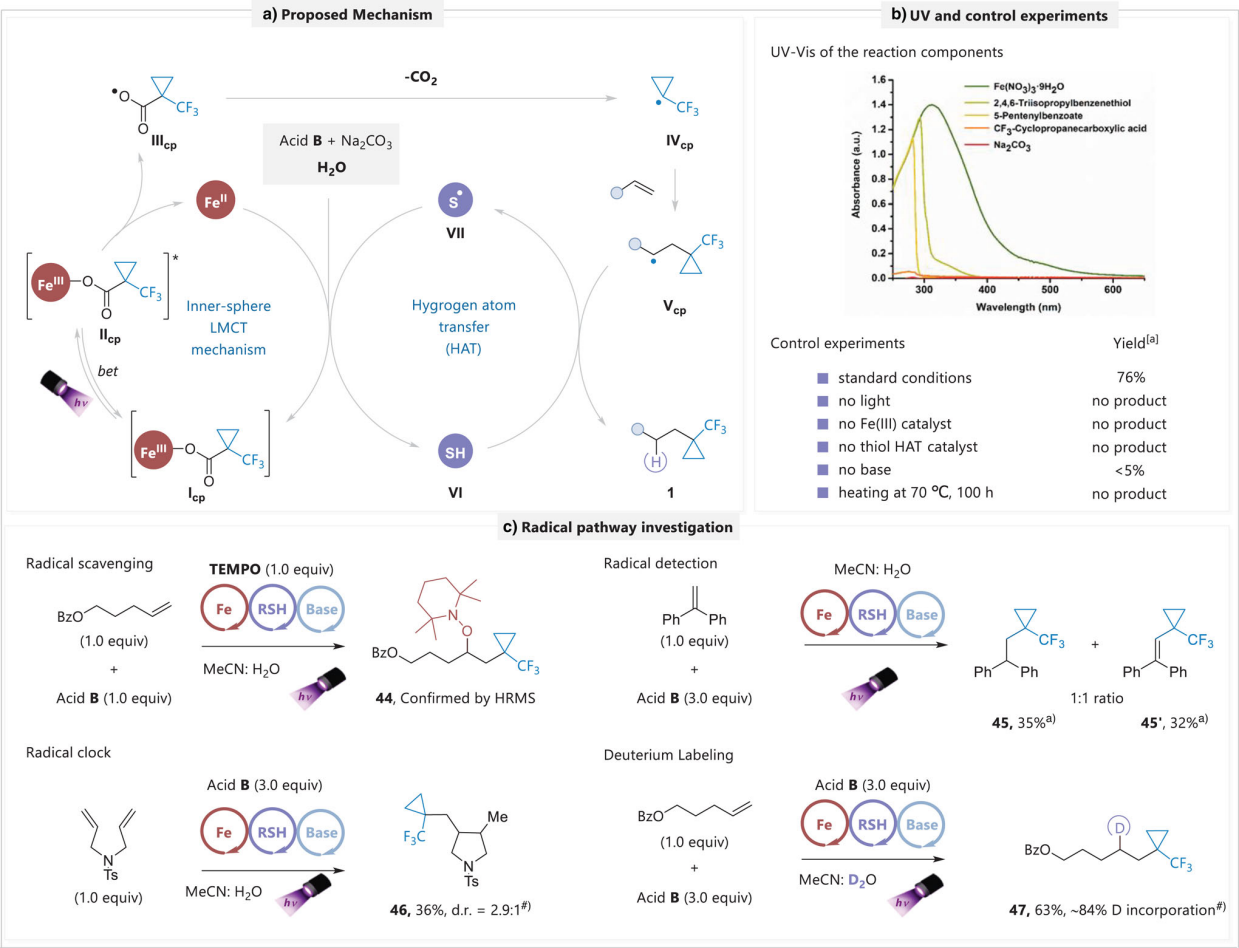
a) Proposed mechanism. b) UV–vis and control experiments. c) Radical trapping experiments. ^a)^Yields are determined by ¹⁹F NMR with PhCF₃ serving as an internal standard. ^#)^The yields of isolated compounds are reported.

To shine light on the mechanistic processes involved in these reactions, we began our studies by analyzing the UV–vis spectrum of the individual components, which showed that only Fe(NO₃)₃·9H₂O absorbed under 390 nm light irradiation. A series of control experiments revealed the crucial role of each reaction component and parameter (Figure 5b). The proposed reaction mechanism starts with the deprotonation of the acid by the base, generating a carboxylate anion. Ligand exchange at the iron center forms an Fe(III)‐carboxylate complex (**I_cp_**, Figure 5a). The ligand‐to‐metal charge‐transfer of this complex is then populated upon purple light irradiation.

This excited state arises from an electronic transition in which a filled orbital on the ligand (the carboxylate group) transfers an electron to an empty d^5^ orbital of the metal center (Fe(III)). This transition decreases the bond order of one (or more) Fe–carboxylate bonds, making the Fe‐L complex **II_cp_** labile and facilitating homolytic bond cleavage.

**Figure 6:**
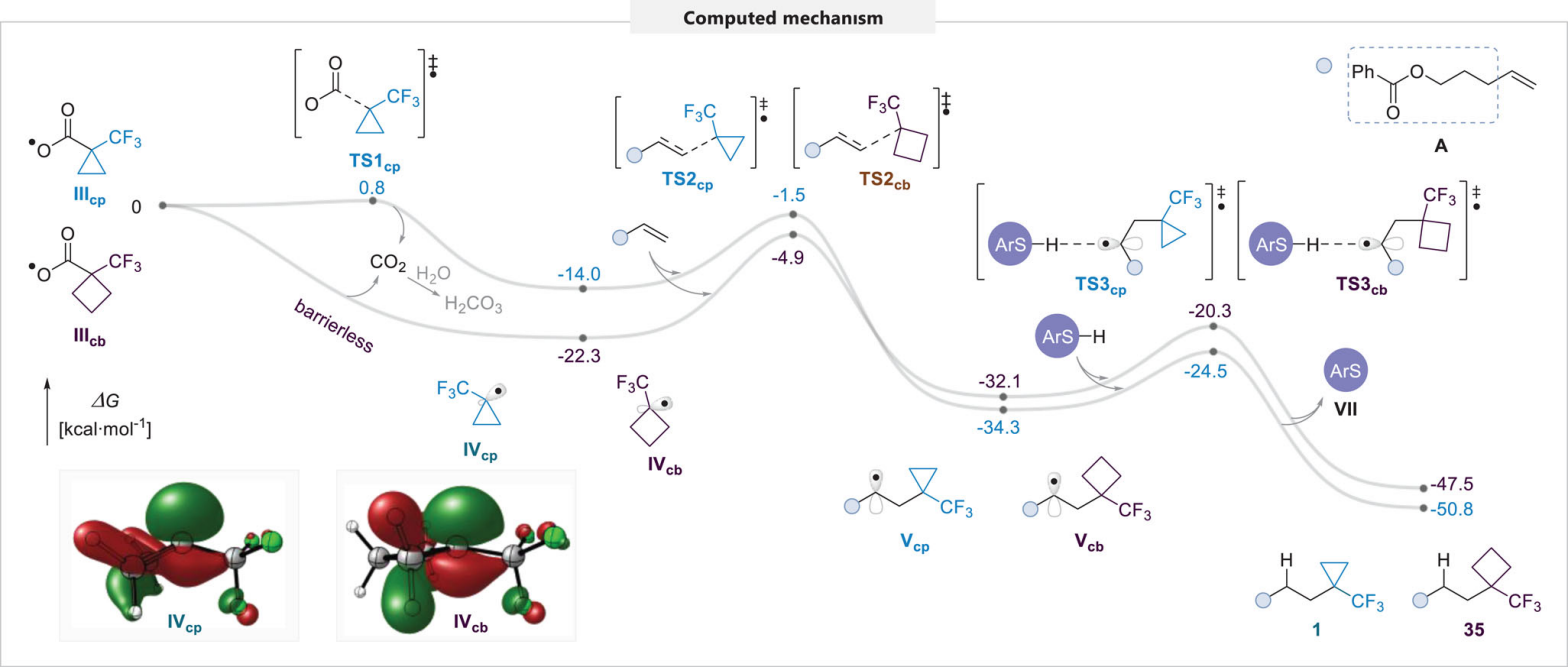
Computed Gibbs free energy reaction profile at the (U)M062X‐D3/def2‐TZVP, SMD(MeCN)//(U)M062X‐D3/def2‐SVP, SMD(MeCN) level of theory. Insets: Singly occupied molecular orbital (SOMO) of **IV_cp_** and **IV_cb_**.

This cleavage generates Fe(II) and a carboxyl radical **III_cp_**, which undergoes a low‐barrier decarboxylation event to extrude CO_2_ (which hydrates to carbonic acid) and produce a (trifluoromethyl)cyclopropyl radical (**IV_cp_**), which was supported by DFT calculations (Δ*G* ^‡^ = 0.8 and Δ_r_ *G* = −14.0 in kcal mol^−1^, Figure 6). However, experimental observations suggest that this kinetically facile step is most likely hindered by a rapid and unproductive back electron transfer (bet) or radical recombination regenerating **I_cp_**, which slows down this process. Additionally, the influence of solvent quenching of the excited state **II_cp_** cannot be excluded. Recent studies have shown that the formation of the carboxyl radical is the rate‐determining step in such processes.^[^ ^34^, ^54^ ^]^ This radical **IV_cp_** adds to the alkene substrate (or to various radical traps, yielding products **45**, **45′**, and **46**, Figure 5c), forming a transient alkyl radical intermediate **V_cp_** (Δ*G* ^‡^ = 12.5 and Δ_r_ *G* = −20.1 in kcal mol^−1^). Complementary studies by HRMS analyses showed that this radical (**V_cp_**) could be effectively trapped when TEMPO was added to the reaction mixture (**44**, Figure 5c). Concurrently, the thiol (**VI**) functions as a hydrogen atom transfer (HAT) reagent, leading to the formation of the hydro‐trifluoromethyl cyclopropane product **2** (Δ*G* ^‡^ = 9.8 and Δ_r_ *G* = −16.5 in kcal mol^−1^). Finally, the formed thiyl radical (**VII**) is reduced by Fe(II), regenerating the Fe(III) catalyst and a thiophenolate anion, which likely deprotonates another acid **B** and sustains the catalytic cycle. When the reaction was conducted in the presence of D_2_O, ca. 84% deuterium incorporation was measured in the product by ^1^H NMR (**47**), which is consistent with hydrogen–deuterium (H/D) exchange between the thiol and the solvent (see compound **39** as well). The acid also undergoes H/D exchange, which does not directly impact the mechanism. The reaction mechanism involving the cyclobutyl derivatives **III_cb_** was also computed to provide a better comparison of the reactivity difference with the cyclopropyl derivative **III_cp_** (Figure 6). Interestingly and in agreement with the RSE scale, the decarboxylation of **III_cb_** was found to proceed without barrier and to be more exergonic than for the cyclopropyl derivative (Δ_r_ *G* = −22.3 vs. Δ_r_ *G* = −14.0 in kcal mol^−1^), confirming the facilitated decarboxylation in this case. The better stability of the cyclobutyl radical **IV_cb_** is reflected in the higher activation energy computed for the subsequent radical addition step (Δ*G* ^‡^ = 17.4 and Δ_r_ *G* = −9.8 in kcal mol^−1^). Eventually, the barrier for the final HAT step was found slightly higher for the cyclobutyl derivative (Δ*G* ^‡^ = 11.8 and Δ_r_ *G* = −15.4, in kcal mol^−1^), likely due to steric reasons upon approach by the bulky TRIP thiol reagent.

## Conclusion

In conclusion, we have developed a general and efficient methodology for the direct decarboxylation of readily available and inexpensive 1‐(trifluoromethyl)cyclopropane‐1‐carboxylic acid to access CF₃‐cyclopropane derivatives through an inner‐sphere, ligand‐to‐metal charge transfer mechanism. This visible‐light‐driven transformation, relying on an earth‐abundant iron catalyst and a redox‐active thiol, exhibits broad applicability across various acids and high functional group tolerance, accommodating both styrenes and unactivated alkenes. The protocol is robust and overcomes significant limitations of existing methods for synthesizing substituted cyclopropane and cyclobutane derivatives while utilizing mild and practical reaction conditions. Finally, a comprehensive mechanistic proposal is presented based on experimental, spectroscopic, and computational synergistic studies. This work showcases the synthetic potential of LMCT‐driven processes and highlights the remarkable value of simple isodesmic equations, computed at the DFT level, in predicting and rationalizing chemical reactivity efficiently. We anticipate that this strategy will pave the way for high‐value bioisosteric transformations, thereby facilitating and streamlining the drug discovery process.

## Supporting Information

The authors have cited additional references within the Supporting Information.^[^ ^57^, ^58^, ^59^, ^60^, ^61^, ^62^, ^63^, ^64^, ^65^, ^66^, ^67^, ^68^, ^69^, ^70^, ^71^, ^72^, ^73^, ^74^, ^75^, ^76^, ^77^, ^78^, ^79^, ^80^, ^81^, ^82^, ^83^, ^84^, ^85^ ^]^

## Conflict of Interests

The authors declare no conflict of interest.

## Supporting information



Supporting Information

## Data Availability

The data that support the findings of this study are available in the Supporting Information of this article.
