# Supplementary material for: Overcoming Challenges in Small‐Ring Transfer: Direct Decarboxylative Hydroalkylation of Alkenes via Iron‐Thiol Catalysis
Source: Angew Chem Int Ed Engl. 2025 Jul 7;64(34):e202508377. doi: 10.1002/anie.202508377 (PMC12253929; doi:10.1002/anie.202508377)

Supporting Information

Overcoming Challenges in Small-Ring Transfer: Direct Decarboxylative Hydroalkylation of Alkenes *via* Iron-Thiol Catalysis

Rahul Giri,[†,a] Po-Kai Peng,[†,b] Anthony J. Fernandes, [a] Shijin Yu,[b] Julian G. West, [*,b] Dmitry Katayev[*,a]

[a]Department of Chemistry, Biochemistry and Pharmaceutical Sciences, University of Bern, Freiestrasse 3, 3012 Bern, Switzerland

[b]Department of Chemistry, Rice University, 6100 Main St, Houston, Texas 77005, United States

†These authors contributed equally to this work

*Corresponding authors

E-mail: jgwest@rice.edu, dmitry.katayev@unibe.ch

Table of Contents

1. Material and methods ---------- 3

1.1 Photoreactor ---------- 4

2. Reaction development ---------- 5

2.1. Iron source ---------- 5

2.2. Concentration ---------- 5

2.3. Iron nitrate loading ---------- 6

2.4. Thiol source ---------- 6

2.5. Base optimization ---------- 7

2.6. Time optimization ---------- 7

2.7. Loading of catalytic reagents ---------- 8

2.8. Screening of known methods for decarboxylation ---------- 8

3. A closer look at the reaction mixture ---------- 9

4. Availability of acids ---------- 10

5. General procedures ---------- 10

6. Mechanistic studies ---------- 12

6.1 Radical scavenging ---------- 12

6.2 Detection of radical ---------- 13

6.3 Radical clock ---------- 14

6.4 Deuterium labeling ---------- 15

6.5 UV-Vis studies ---------- 16

6.6 Steric effects on HAT ---------- 19

7. NMR description ---------- 20

8. Computational details ---------- 37

8.1 Computational methods ---------- 37

8.2 Radical stabilization energy scale ---------- 37

8.3 Philicity indices and other parameters ---------- 41

8.4 Redox potentials ---------- 43

8.5 Computed reaction mechanism ---------- 45

8.6 Computed structures ---------- 46

8.6.1 RSE scale ---------- 46

8.6.2 Reaction mechanism ---------- 54

9. NMR spectra ---------- 67

1. Material and methods

All reactions were performed in flame-dried glassware under an argon atmosphere containing a teflon-coated stirring bar and dry septum. In addition, glassware was dried overnight at 120 °C before use. Starting materials are commercially available and were purchased from Thermoscientific – Acros, Sigma Aldrich, Apollo Scientific, Fluorochem, TCI, and Chemie Brunschwig AG unless otherwise noted. Anhydrous acetonitrile was distilled over CaH_2 and stored over pre-conditioned 3 Å mol sieves for at least 12 h before use.

Analytical thin-layer chromatography (TLC) was performed on Merck silica gel 60 F254 TLC glass plates and visualized with 254 nm light and potassium permanganate staining solutions followed by heating when required. Purification of reaction products was carried out by flash chromatography using Brunschwig silica 32-63, 60Å under 0.3-0.5 bar over pressure. Medium pressure liquid chromatography (MPLC) was performed on a CombiFlash R_f200 System from Teledyne ISCO with a built-in UV-detector and fraction collector or manually using silica gel SilicaFlash P60, 40-63 µm. Teledyne ISCO RediSep Rf flash columns were used, featuring a particle size of 0.035–0.070 mm and a mesh range of 230–400. Normal phase preparatory HPLC purification was conducted on a Teledyne Isco CombiFlash EZ Prep system using a Macherey-Nagel VP 250/21 Nucleosil 50-5 columns.

^{1}H- and ^{13}C-NMR spectra were recorded on Bruker Ultrashield 300 (operating at 300.1 MHz and 75.5 MHz, respectively), Bruker Ascend 400 (operating at 400.1 MHz and 100.6 MHz, respectively), Bruker AVANCE III 500 (operating at 500.1 MHz and 125.6 MHz, respectively), ^{19}F-NMR spectra on Bruker DPX-300 and Bruk er Ultrashield 300 (at 282 MHz) and Bruker DPX-400 and Bruker Ascend 400 (at 376 MHz) Bruker DPX-500 and Bruker AVANCE III 500 (at 477 MHz). The chemical shifts are reported in parts per million (ppm) and coupling constants (*J*) are given in Hertz (Hz). ^{1}H-NMR spectra are reported with the solvent resonance as the reference unless noted otherwise ($CDCl_3$ at 7.26 ppm or as indicated). Peaks are reported as (s = singlet, d = doublet, t = triplet, q = quartet, m = multiplet or unresolved, coupling constant(s) in Hz, integration). ^{13}C-NMR spectra were recorded with ^{1}H-decoupling and are reported with the solvent resonance as the reference unless noted otherwise ($CDCl_3$ at 77.16 ppm or as indicated). ^{19}F-NMR spectra were recorded with ^{1}H-decoupling or coupled.

A Bruker Tensor III spectrometer equipped with a golden gate was used to record infrared spectra. HR-MS (ESI^+) mass spectra were measured on a Bruker FTMS 4.7T BioAPEX II and Thermo Scientific LTQ Orbitrap XL equipped with a static nanospray ion source and mass spectrometry service operated on VG-TRIBRID for electron impact ionization (EI), or Varian IonSpec Spectrometer for electrospray ionization (ESI) and are reported as (*m/z,*). Electron impact ionization mass spectra (EI-MS) were run on a gas chromatography – mass spectrometry (GC-MS) instrument of Agilent 8890 series GC system and Agilent 5977B GC/MSD. UV-Vis spectroscopy was measured FS5 Spectrofluorometer from Edinburgh Instruments.

The references in the supplementary information file are numbered in accordance with the main manuscript. Any additional references not cited in the manuscript are numbered sequentially, starting after the last reference number used in the main text.

1.1 Photoreactor

The 390 nm Kessil lamps were purchased directly from Kessil and operated at 100% intensity across all substrates. Either the Hepatochem EvoluChemTM PhotoRedOx Box, as shown in **Figure S1**, was used, or a custom-designed 390 nm photoreactor was employed for high-power applications. This custom photoreactor featured 20 evenly spaced LEDs, each rated at 10 W, resulting in a total power output of 200 W. The reaction temperatures in each piece of equipment were determined to be between 25-35 ºC. The custom-designed photoreactor at UNIBE, DCBP, is a significantly modified version of the initial design conceived by B. Jelier in collaboration with the mechanical workshop of the Department of Chemistry and Applied Biosciences at ETH Zurich, as reported in *Angew. Chem. Int. Ed.* **2018**, *57*, 13784.[56]

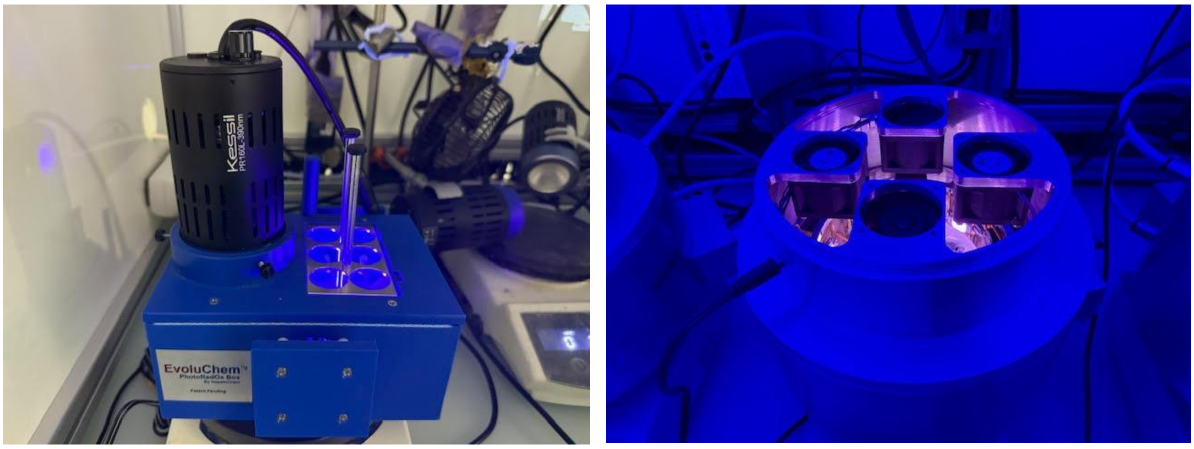

Figure S1: Setup: Hepatochem EvoluChemTM PhotoRedOx Box equipped with a 390 nm Kessil LED light (left). Custom-designed photoreactor with 20 LEDs, a cooling frame with a chiller, and four fans (right).

[56] B. J. Jelier, P. F. Tripet, E. Pietrasiak, I. Franzoni, G. Jeschke, A. Togni, *Angew. Chem. Int. Ed.* **2018**, *57*, 13784–13789; *Angew. Chem.* **2018**, *130,* 13980 –13985.

2. Reaction development

2.1. Iron source

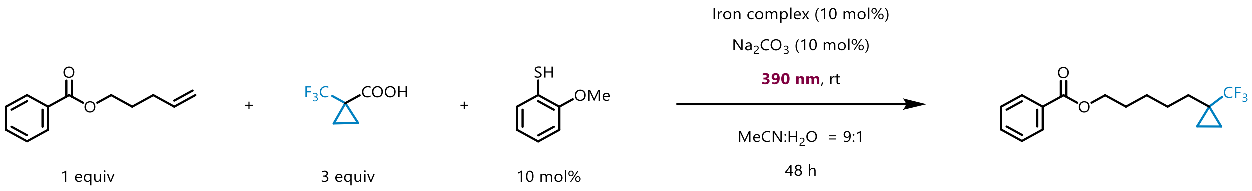

Entry	Iron complex (10 mol%)	Yield %
1	Iron(II) acetate	34
2	Iron(II) triflate	5
3	Iron(III) triflate	25
4	Iron(III) chloride	8
5	Iron(II) chloride	23
6	Iron(III) bromide	20
7	Iron(II) Bromide	8
8	Ferrous(II) oxalate dihydrate	5
9	Iron-salen complex; *(CAS Number 18601-34-8)*	13
10	Tris(acetylacetonato)iron(III)	18
11	Iron(III) nitrate nonahydrate	35

Table S1. Yields are determined by ^{19}F NMR using $PhCF_3$ as an internal standard.

2.2. Concentration

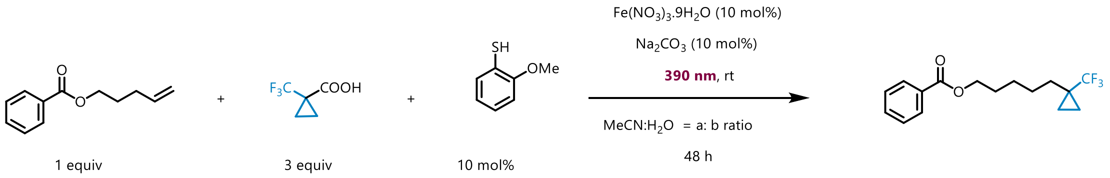

Entry	Ratio of solvents (MeCN: H_2O)	Yield %
1	Only MeCN	3
2	1:9	34
3	3:7	31
4	1:1	40
5	7:3	31
6	9:1	35
7	Only H_2O	0

Table S2. Yields are determined by ^{19}F NMR using $PhCF_3$ as an internal standard.

2.3. Iron nitrate loading

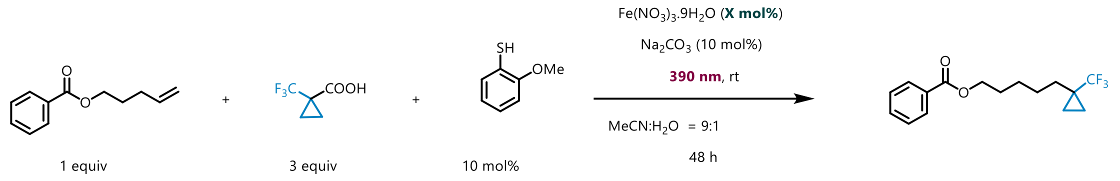

Entry	$Fe(NO_3)_3 . 9H_2O$ (X mol%)	Yield %
1	5	32
2	10	40
3	20	42
4	30	26
5	50	10
6	100	2
7	200	0

Table S3. Yields are determined by ^{19}F NMR using $PhCF_3$ as an internal standard.

2.4. Thiol source

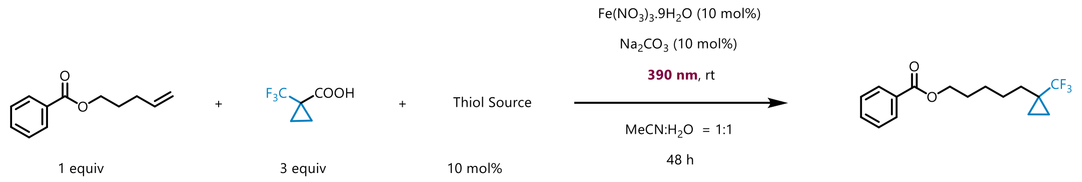

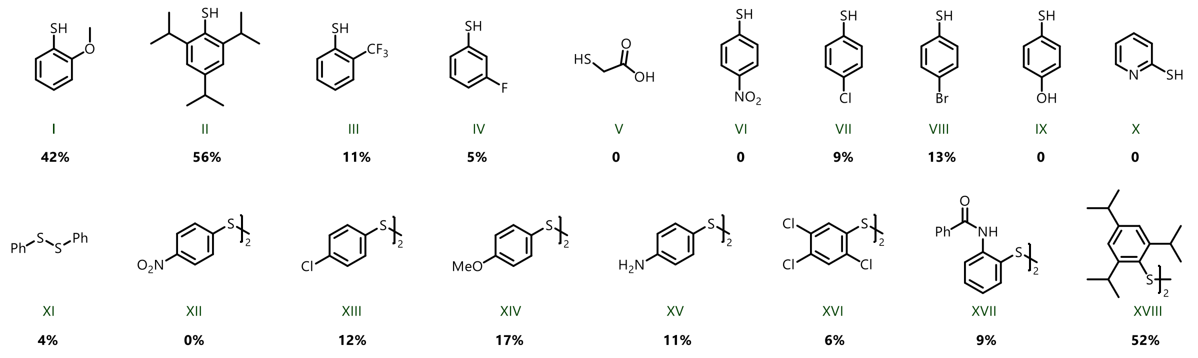

Table S4. Yields are determined by ^{19}F NMR using $PhCF_3$ as an internal standard.

2.5. Base optimization

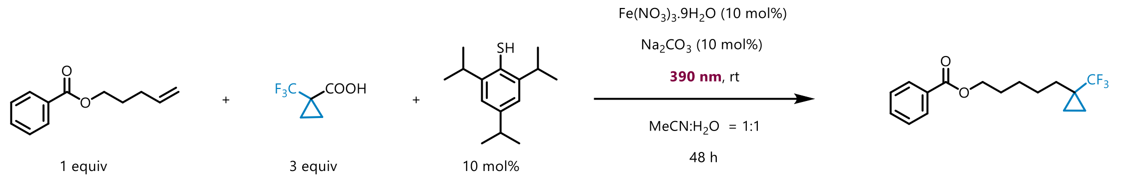

Entry	Base (10 mol%)	Yield %
1	Na_2CO_3	56
2	Ag_2CO_3	24
3	K_2CO_3	34
4	Cs_2CO_3	36
5	Cesium pivalate	9
6	Cesium benzoate	11
7	CsF	10
8	CsBr	3
9	No Base	0

Table S5. Yields are determined by ^{19}F NMR using $PhCF_3$ as an internal standard.

2.6. Time optimization

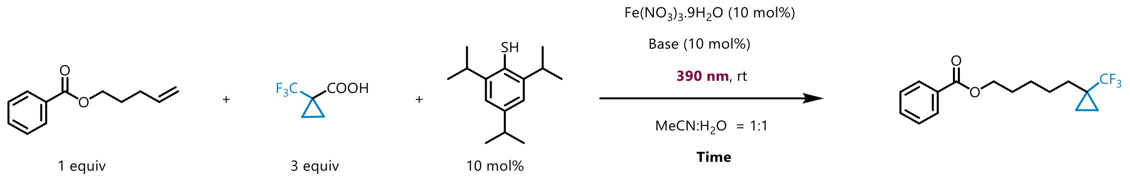

Entry	Time (hours)	Yield %
1	24	42
2	48	56
3	48 (DMF : H_2O 1:1)	48
4	72	72

Table S6. Yields are determined by ^{19}F NMR using $PhCF_3$ as an internal standard.

2.7. Loading of catalytic reagents

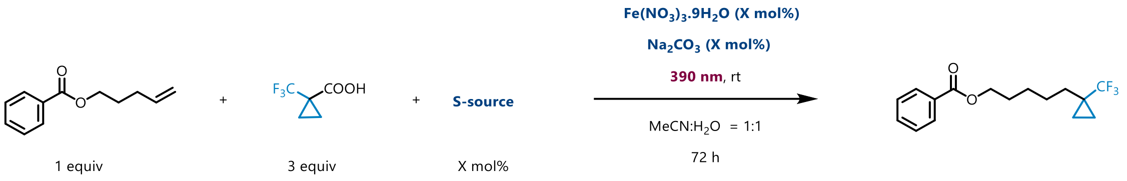

Entry	$[Fe(NO_3)_3 . 9H_2O$	TRIP-Thiol	Na_2CO_3	Yield %
1	5 mol%	5 mol%	5 mol%	32
2	10 mol%	10 mol%	10 mol%	72
3	20 mol%	20 mol%	20 mol%	76

Table S7. Yields are determined by ^{19}F NMR using $PhCF_3$ as an internal standard.

2.8. Screening of known methods for decarboxylation

We followed similar conditions from the reported protocols and applied them to the acid of our interest (**Figure S2**).[54,55,57] Our results showed that only the conditions used by the West group yielded the product in a reasonable amount (34%).[44] The phthalimide ester was synthesized following the reported protocol, and its spectroscopic data are consistent with the literature data.[27]

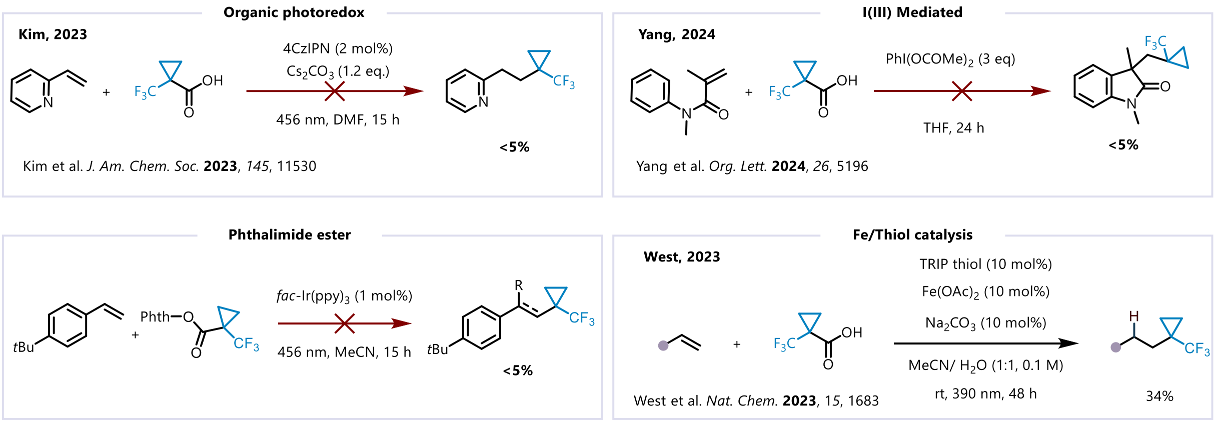

Figure S2: The summary of reported protocols for decarboxylation.

[27] F. Toriyama, J. Cornella, L. Wimmer, T.-G. Chen, D. D. Dixon, G. Creech, P. S. Baran, *J. Am. Chem. Soc.* **2016**, *138*, 11132–11135.
[44] K.-J. Bian, Y.-C. Lu, D. Nemoto, S.-C. Kao, X. Chen, J. G. West, *Nat. Chem.* **2023**, *15*, 1683–1692.
[54] S. Han, K. L. Samony, R. N. Nabi, C. A. Bache, D. K. Kim, *J. Am. Chem. Soc.* **2023**, *145*, 11530–11536.
[55] Z.-P. Ye, M. Guo, Y.-Q. Ye, C.-P. Yuan, H.-L. Wang, J.-S. Yang, H.-B. Chen, H.-Y. Xiang, K. Chen, H. Yang, *Org. Lett.* **2024**, *26*, 5196–5201.
[57] A. Tlahuext-Aca, R. A. Garza-Sanchez, M. Schäfer, F. Glorius, *Org. Lett.* **2018**, *20*, 1546–1549.

3. A closer look at the reaction mixture

A preliminary analysis of the reaction mixture was performed using NMR (**Figure S3**), revealing a competitive reaction that occurred after decarboxylation. The quenching of the formed cyclopropyl-CF_3 radical by a hydrogen atom transfer (HAT) process, either by thiol or water, was observed. Since this competing process is significant, a higher loading of the CF_3-cyclopropyl reagent is required to ensure that the reaction reaches completion.

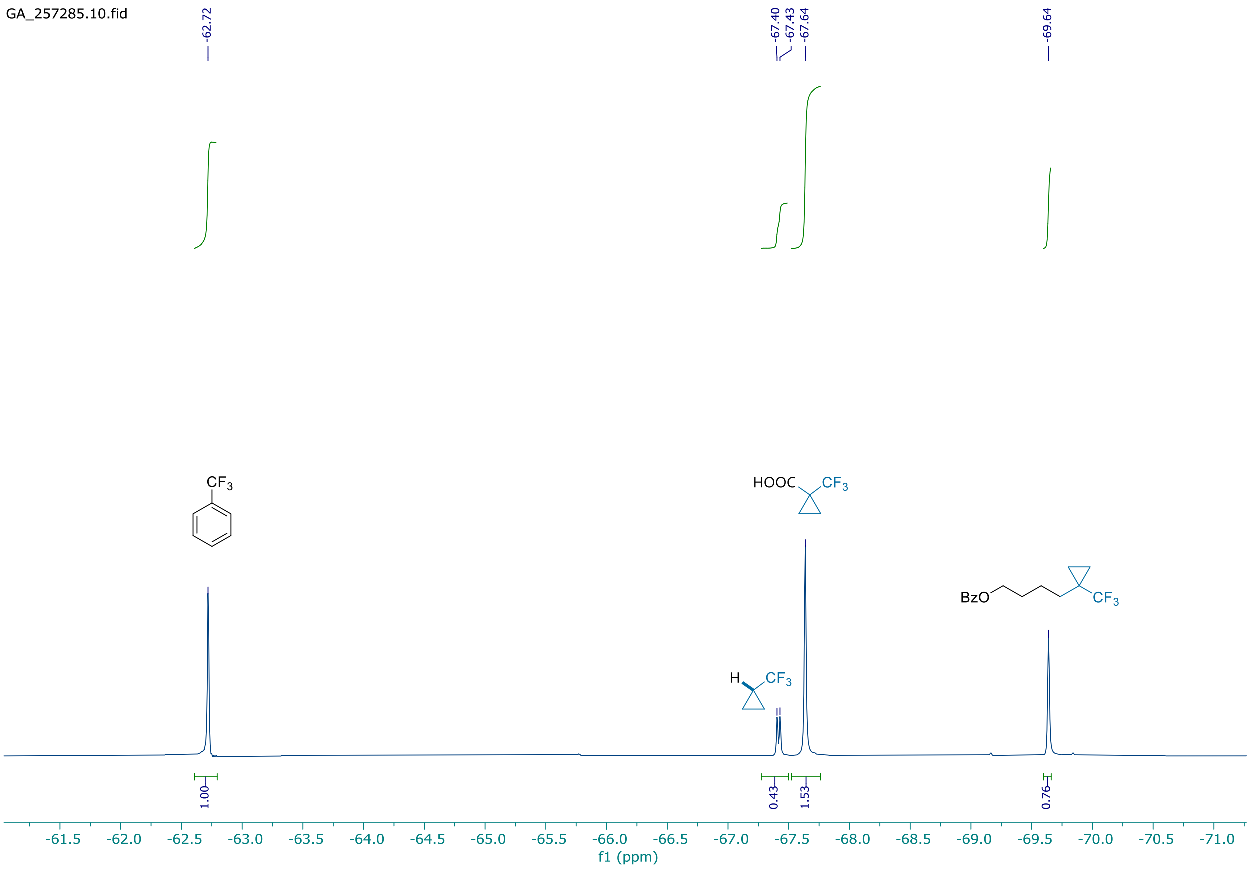

Figure S3: The ^{19}F-NMR analysis of the crude mixture.

4. Availability of acids

All acids (**Figure S4**) were purchased commercially and used without any further purification.

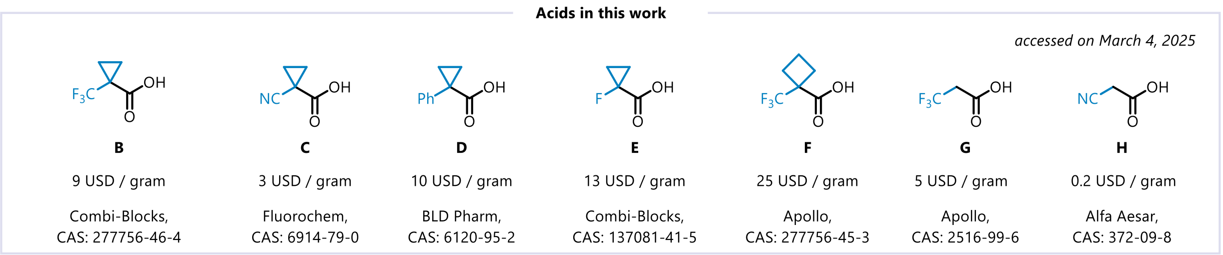

Figure S4: Reagents availability.

5. General procedures

General procedure A

The Synthesis of Trifluoromethyl-cyclopropyl compounds

To the corresponding olefin (1.0 equiv, 0.2 mmol or as described), carboxylic acid (3.0 equiv), Na_2CO_3 (4mg, 0.2 equiv), and $Fe(NO_3)_3 \cdot 9H_2O$ (16 mg, 20 mol%) in frame–dried 7 mL vial was added TRIP-SH (0.2 equiv) and anhydrous MeCN and water (v/v = 1:1) (0.1 M) at room temperature. The mixture was then purged with N_2 for 30 seconds and sealed at the ambient temperature under the irradiation of 390 nm Kessil light for 72 h. After the reaction was completed, the solid was filtered, washed with diethyl ether (10 mL), and the crude mixture was then purified via flash column chromatography on silica gel with appropriate eluents to give the desired product.

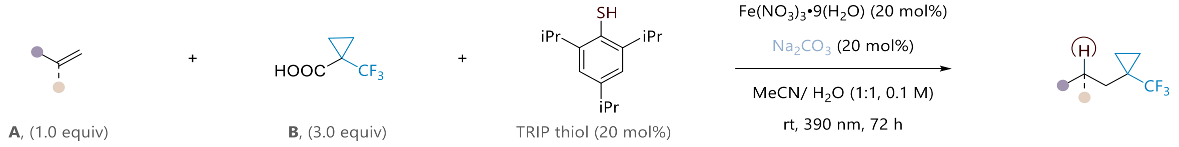

General procedure B

The Synthesis of substituted-cyclic compounds

To the corresponding olefin (1.0 equiv, 0.2 mmol or as described), corresponding carboxylic acid (3.0 equiv), Na_2CO_3 (4mg, 20 mol%), and $Fe(NO_3)_3 \cdot 9H_2O$ (16 mg, 20 mol%) in frame–dried 7 mL vial was added TRIP-SH (20 mol%) and anhydrous MeCN and water (v/v = 9:1) (0.1 M) at room temperature. The mixture was then purged with N_2 for 30 seconds and sealed at the ambient temperature under the irradiation of 390 nm Kessil light for 48 to 72 h. After the reaction was completed, the solid was filtered, washed with diethyl ether (10 mL), and the crude mixture was then purified via flash column chromatography on silica gel with appropriate eluents to give the desired product.

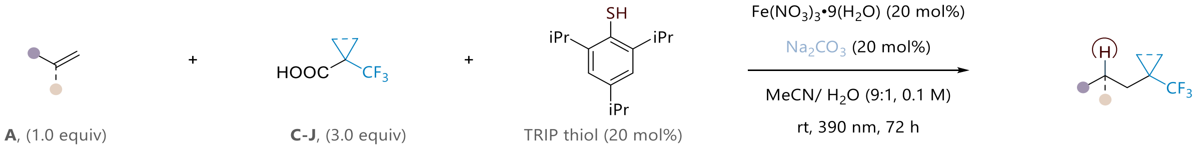

Observed limitation of olefins:

Electron-poor olefins, such as acrylonitrile, exhibited low reactivity under the reaction conditions. Additionally, steric hindrance prevents the reaction with substrates such as trans-stilbene. In the case of cycloheptene, thiol addition predominates rather than the desired transformation. Furthermore, 2-vinylpyridine resulted in the formation of a complex mixture, likely due to polymerization (**Figure S5**).

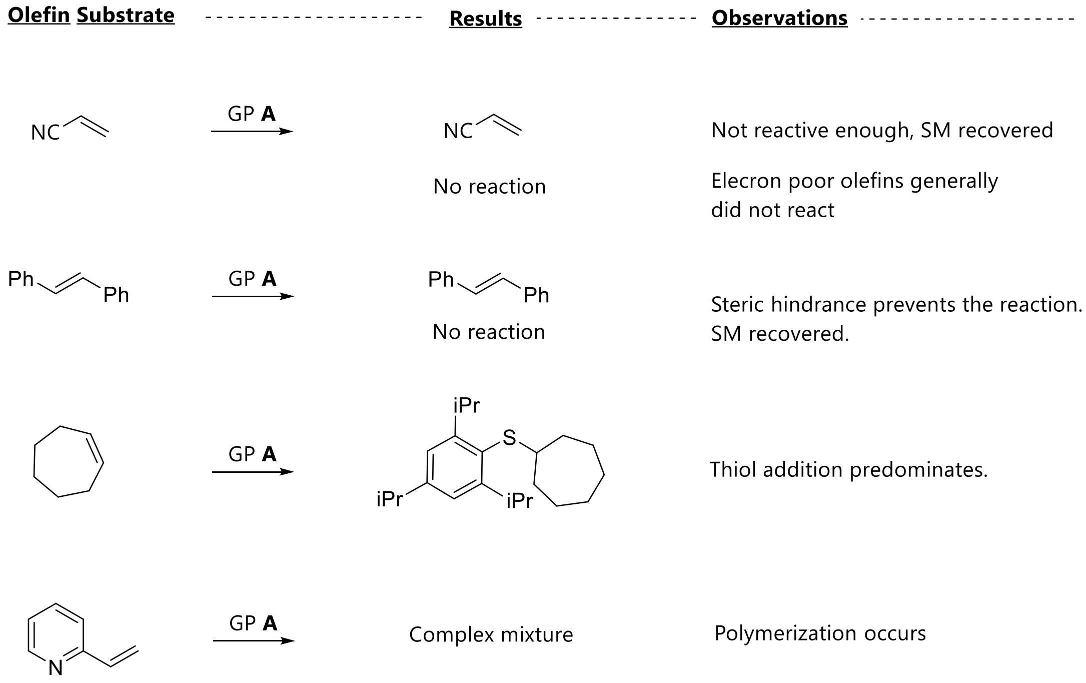

Figure S5: Observed limitation of the methodology.

6. Mechanistic studies

6.1 Radical scavenging

When the standard reaction is conducted in the presence of (2,2,6,6-Tetramethylpiperidin-1-yl)oxyl (TEMPO) as a radical scavenger, the reaction is completely inhibited, and the desired product is not detected by HRMS. Instead, a TEMPO-trapped adduct is observed (**Figure S6**). Although the regioselectivity of this trapping remains uncertain, the result suggests that long-lived radicals are intercepted, indicating that the reaction likely proceeds through a radical pathway.

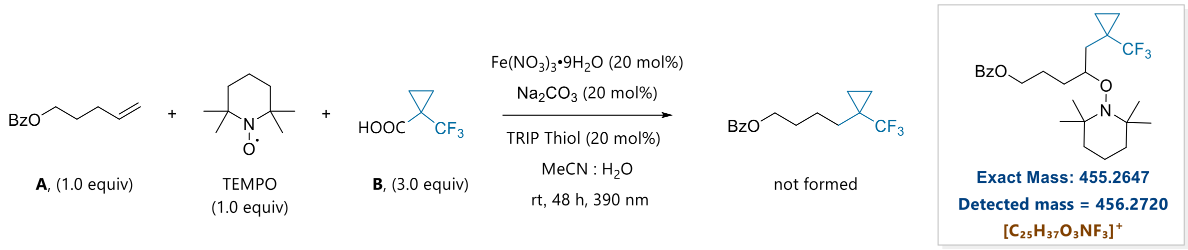

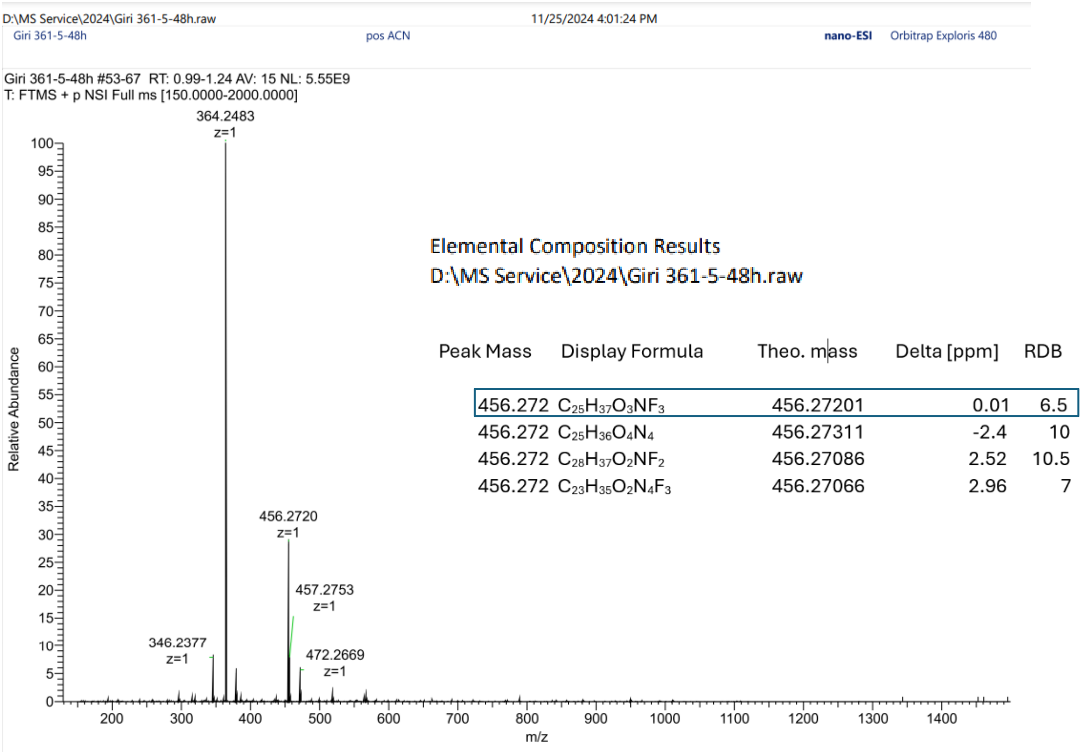

Figure S6: HRMS analysis of the reaction sample with TEMPO.

Procedure: A flame-dried 8 mL crimp-cap vial was charged with Iron(III) nitrate nonahydrate (20 mg, 50 µmol, 20.0 mol%), olefin (0.25 mmol, 1.0 equiv), acid reagent (0.75 mmol, 3.0 equiv), TEMPO (39 mg,1.0 equiv), and Na_2CO_3 (5 mg, 50 µmol, 20.0 mol%). A magnetic stir bar was added, and the vial was sealed with a crimp cap. The contents of the vial were then subjected to three vacuum/N_2 cycles. MeCN (1.25 mL) and H_2O (1.25 mL) was added with a 2 mL syringe. The 2,4,6-Triisopropylbenzenethiol (12 µL, 50 µmol, 20.0 mol%) was introduced to the solution *via* Hamilton microsyringes. The reaction mixture was irradiated at room temperature under 390 nm Kessil lamps for 48 h. The crude was analyzed with HRMS.

6.2 Detection of radical

Further, using 1,1-diphenylethylene as a radical trapping reagent under the standard reaction conditions resulted in the formation of two distinct products in a 1:1 ratio, with yields of 35% and 38%, respectively. The reaction is likely indicative of the radical-mediated reactions (**Figure S7**).

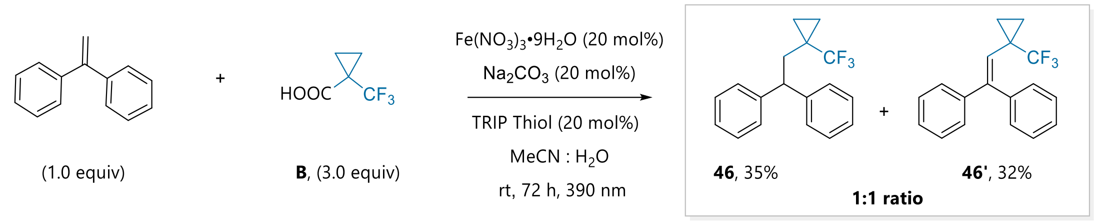

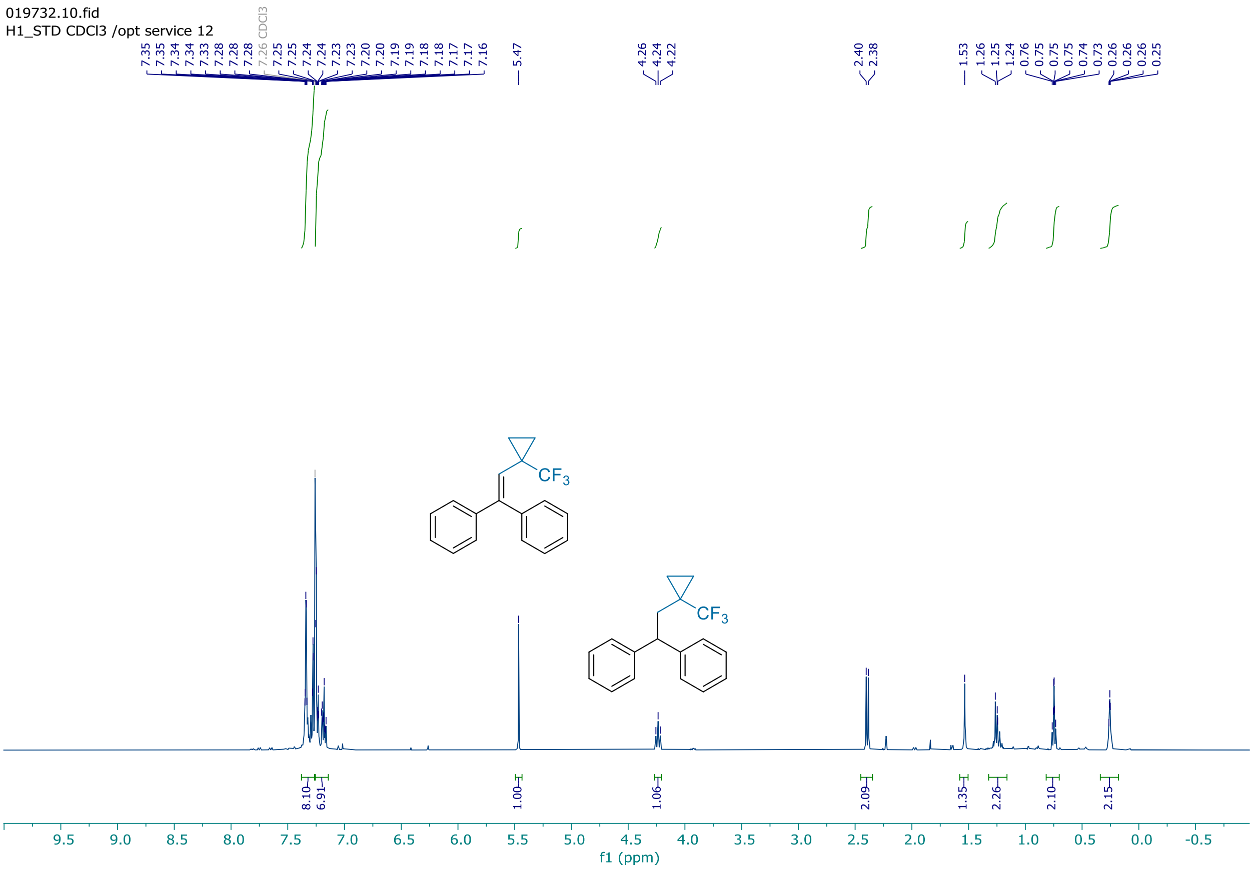

Figure S7: ^{1}H-NMR analysis of the reaction mixture.

6.3 Radical clock

The CF_3-cyclopropyl radical is generated and undergoes rapid cyclization onto a *N*,*N*-diallyl-4-methylbenzenesulfonamide system, forming a stabilized pyrrolidine radical intermediate. This radical is then trapped by a thiol, leading to the final methylated product. The reaction proceeds with a 36% yield and a 2.9:1 diastereomeric ratio, confirming the radical nature of the transformation (**Figure S8**).

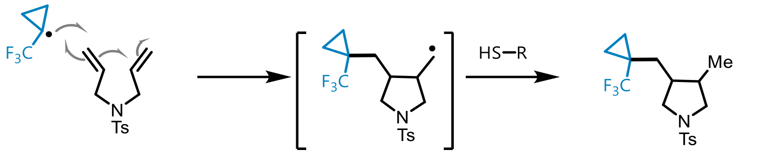

46, 36%, d.r. = 2.9:1

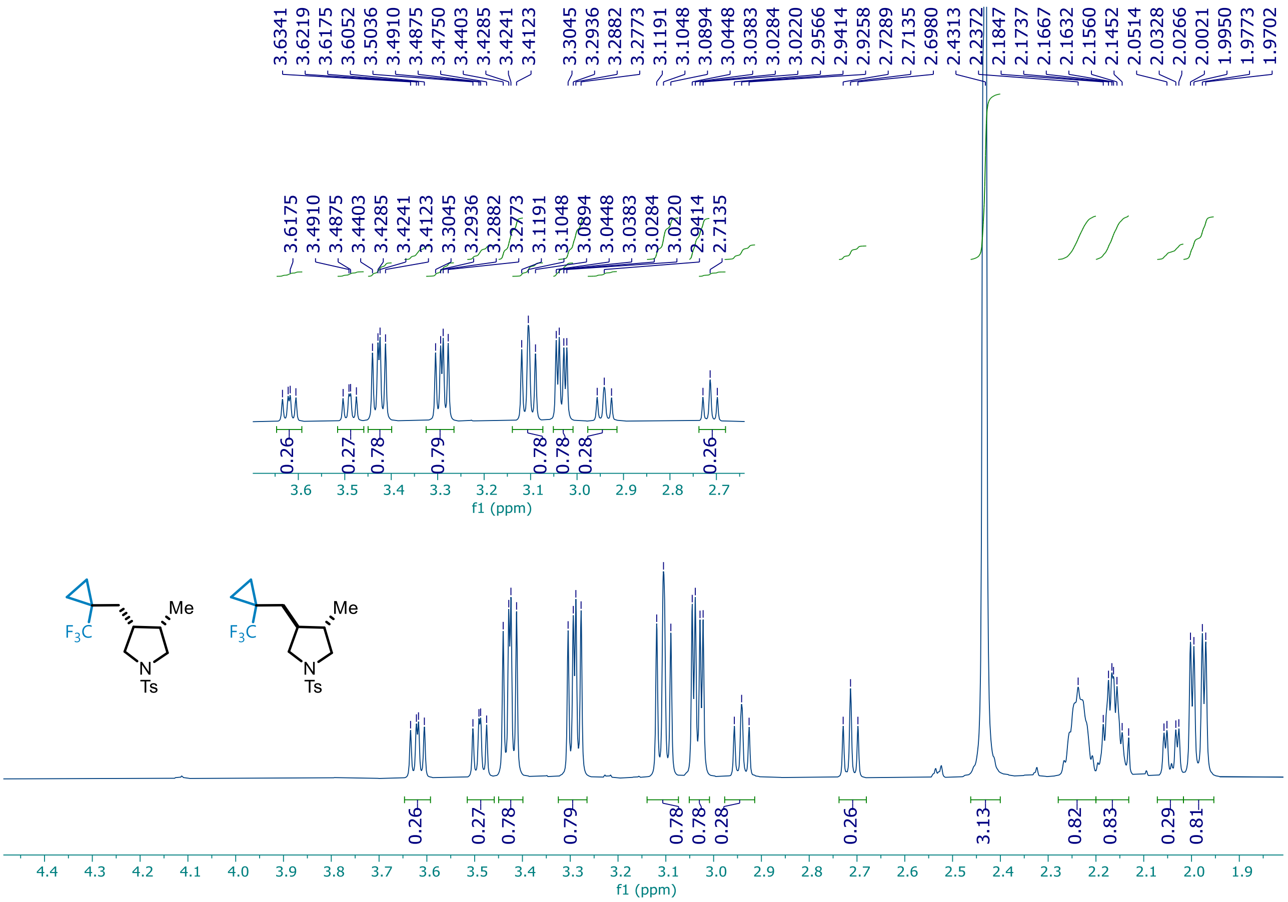

Figure S8: ^{1}H-NMR analysis of the reaction mixture.

6.4 Deuterium labeling

When the reaction is carried out in a MeCN/D_2O solvent mixture using the standard conditions, approximately 84% deuterium incorporation is observed (**Figure S9**). This substantial incorporation indicates that the thiol group abstracts D from D_2O, resulting in the formation of product **47**. The degree of deuterium incorporation is determined by NMR analysis and comparison with compound 1.

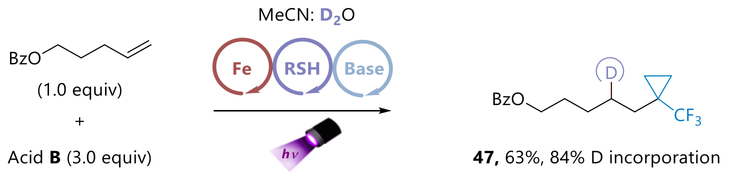

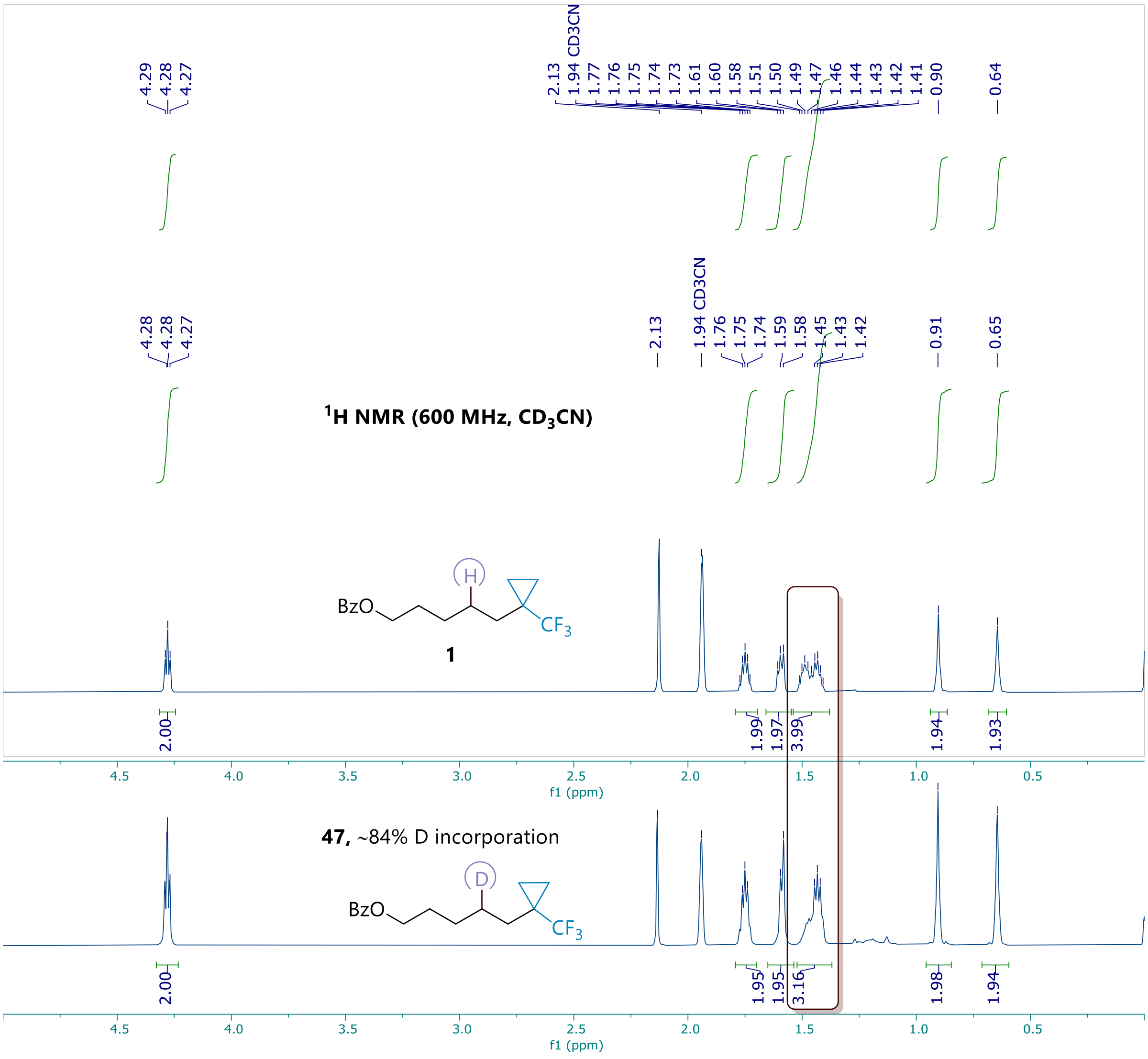

Figure S9: ^{1}H-NMR analysis of the pure compounds **1** and **47**.

6.5 UV-Vis studies

<u>Experiment 1:</u> UV–Vis absorption spectroscopy was employed to further investigate the reaction mechanism. Initially, the absorption spectra of the starting materials were recorded in acetonitrile separately (Figure 10).

Sample preparation: To prepare a 10 mL sample solution, the following amounts of reagents were dissolved in acetonitrile: $Fe(NO_3)_3 \cdot 9H_2O$ (2 mM, 0.020 mmol, 8.08 mg), TRIP thiol (10 mM, 0.10 mmol, 27.0 mg), 5-pentenenyl benzoate (10 mM, 0.10 mmol, 20.4 mg), CF_3-cyclopropane carboxylic acid (10 mM, 0.10 mmol, 15.4 mg), and Na_2CO_3 (10 mM, 0.10 mmol, 10.6 mg). Each reagent was thoroughly dissolved in acetonitrile, and the final volume was adjusted to 10 mL. For UV-Vis analysis, 2.5 mL of the prepared solution was placed into a quartz cuvette, and the absorption spectra were recorded over the desired wavelength range (250 nm to 700 nm) (Figure 10).

Observations: All individual spectra are summarized in a single **Figure S10** for clarity.

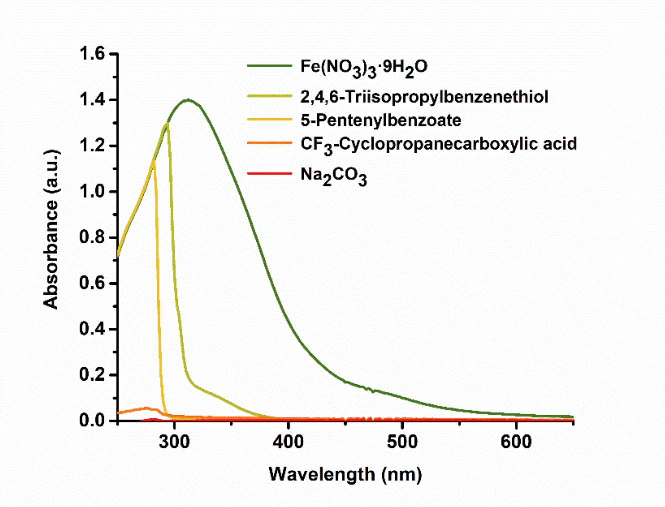

Figure S10: UV-Vis spectra obtained for a solution containing the reaction components in MeCN. $Fe(NO_3)_3 \cdot 9H_2O$ (2 mM), TRIP thiol (10 mM), 5-pentenenyl benzoate (10 mM), acid reagent (10 mM), and Na_2CO_3 (10 mM).

<u>Experiment 2:</u> To understand the stability of ferric nitrate under irradiation over time, a solution of $Fe(NO_3)_3 \cdot 9H_2O$ in MeCN was investigated, and the recorded spectra are summarized in **Figure S11**.

Sample preparation: $Fe(NO_3)_3 \cdot 9H_2O$ (5 mM, 20.2 mg) was weighted and dissolved in acetonitrile to a final volume of 10 mL. For UV-Vis analysis, 2.5 mL of the prepared solution was placed into a quartz cuvette, and the absorption spectra were recorded over different times.

Observations: The slow decomposition of $Fe(NO_3)_3 \cdot 9H_2O$ under 390 nm light suggests that the iron catalyst is generally stable under irradiation, indicating potential photostability. However, further testing reveals that after 3 hours and up to 20 hours of prolonged exposure, the iron nitrate decomposes very slowly, likely due to the photodecomposition of the ligands.

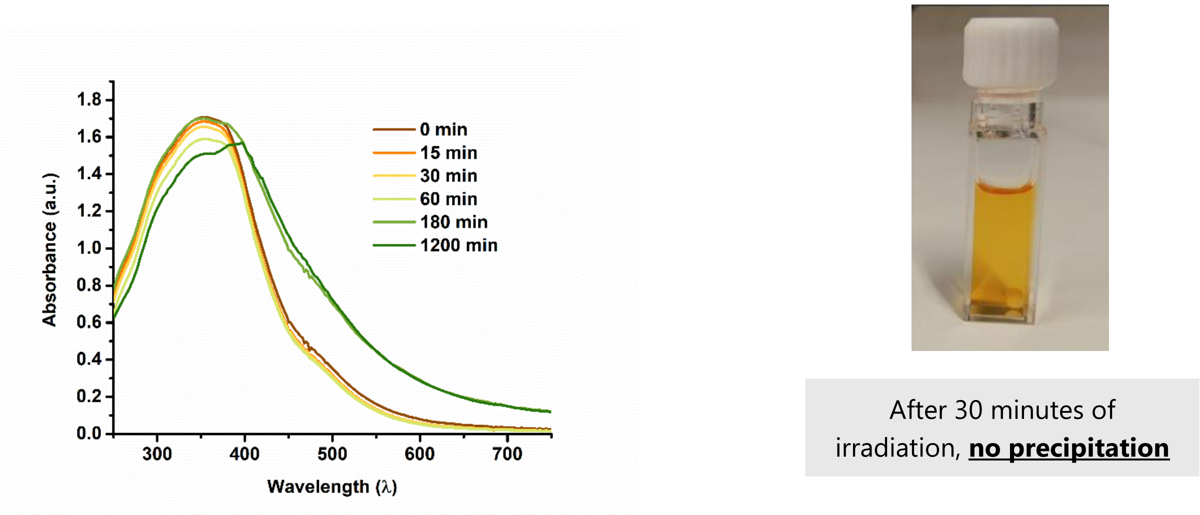

Figure S11: UV-Vis spectra of a solution of $Fe(NO_3)_3·9H_2O$ (5 mM) in MeCN irradiated at 390 nm.

Experiment 3: In the next step of studies, we investigated a mixture of $Fe(NO_3)_3·9H_2O$, 1-(trifluoromethyl)cyclopropane-1-carboxylic acid and Na_2CO_3 in MeCN under irradiation (**Figure S12**).

Sample preparation: To prepare a 10 mL sample solution with a 1:1:1 ratio of all components, $Fe(NO_3)_3·9H_2O$ (20.2 mg, 5 mM), CF_3-cyclopropane acid (7.7 mg, 5 mM), and Na_2CO_3 (4.2 mg, 5 mM) were weighed and dissolved separately in acetonitrile to a final volume of 10 mL each. Equal volumes of these solutions (0.8 mL) were combined to prepare 2.4 mL of the final sample, which was then placed in a quartz cuvette for UV-Vis measurement.

Observations: A gradual decrease in absorbance over time (300–400 nm) was observed, along with the precipitation of the iron complex (Figure 12). These changes suggest ongoing redox processes (e.g., Fe^{III}-Fe^{II}), likely involving ligand exchange, the formation of iron (poly)carboxylate complexes, and their subsequent decomposition over time. The formed precipitate may be a result of an acid-base reaction between the H_2O ligand (pK_a = 1.5-2.5) at the iron complex and Na_2CO_3. Alongside, we attempted to detect mono- or poly(carboxylate) iron species through HRMS; however, no clear species were identified.

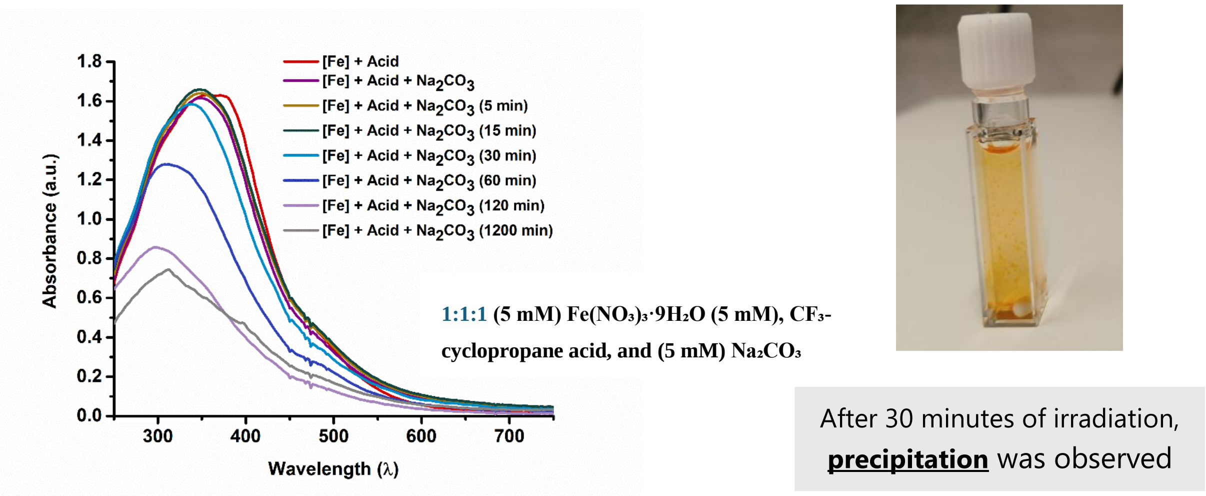

Figure S12: Time-resolved UV-Vis spectra were recorded for a solution of $Fe(NO_3)_3·9H_2O$ (5 mM), CF_3-cyclopropane acid (5 mM), and Na_2CO_3 (5 mM), in MeCN, while irradiating the solution at 390 nm.

Experiment 4: To better replicate the reaction environment and further identify the reactive species, the UV-Vis spectrum was measured using a mixture that corresponds to those employed under the actual reaction conditions, specifically a 1:1 mixture of MeCN and water (**Figure S13**).

Sample preparation: To prepare a 10 mL sample in a mixture of these solvents (v/v = 1:1), (15 mM, 23.1 mg) of CF_3-cyclopropane acid, (5 mM, 20.2 mg) of $Fe(NO_3)_3{\cdot}9H_2O$, and (3 mM, 2.5 mg) of Na_2CO_3 were accurately weighed. These components were dissolved together in 5 mL of anhydrous acetonitrile and 5 mL of deionized water. A 2.5 mL aliquot of the resulting solution was taken and used for UV-Vis spectral measurements.

Observations: The UV-Vis spectra revealed absorption bands centered around 230 nm and 280 nm. The significant change in absorption occurs only when water is added as a solvent. It is also evident from the spectra that, over time, there is an increase in the band, especially at 280 nm. While the peak at 230 nm usually corresponds to the Fe^{3+} ion in solution and does not significantly change, the peak at 280 nm arises from the formation of a hydrated iron complex (no precipitate is observed), which increases over a period of 60 minutes, as illustrated in Figure 13. It is highly likely that, due to the low concentration of carboxylic acid in the reaction mixture when using a 1:1 MeCN/H_2O solvent system, an equilibrium forms between the $Fe(H_2O)_6^{3+}$ and $Fe(H_2O)_5(RCO_2)^{2+}$ complexes. The formation of a monocarboxylate complex, as a future reactive species, at low concentration would also explain the long reaction time observed in this catalytic system.

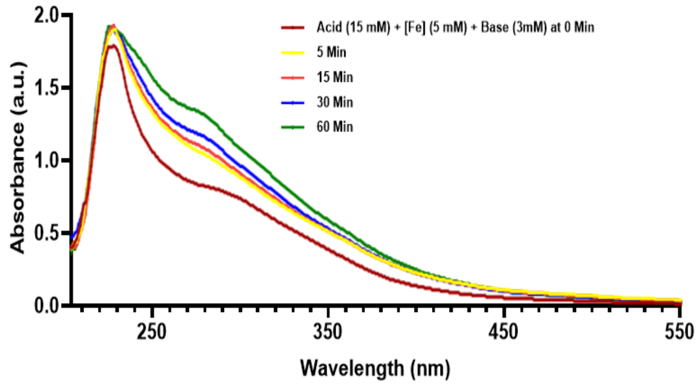

3:1:0.6 (15 mM), CF_3-cyclopropane acid, (5 mM) $Fe(NO_3)_3{\cdot}9H_2O$ and (3 mM) Na_2CO_3

Figure S13: Time-resolved UV-Vis spectra of $Fe(NO_3)_3{\cdot}9H_2O$, Na_2CO_3, and CF_3-cyclopropane acid (5:3:15 mM) in MeCN:H_2O (1:1), while irradiating the solution at 390 nm.

6.6 Steric effects on HAT

The effectiveness of bulky TRIP thiol in promoting hydrogen atom transfer (HAT) in 1,1-disubstituted alkenes under standard conditions has been studied. Despite the potential steric hindrance at the alkyl radical site that can impede HAT, a comparison of various thiols showed that TRIP thiol outperformed simpler, less sterically hindered alternatives (**Figure S14**).

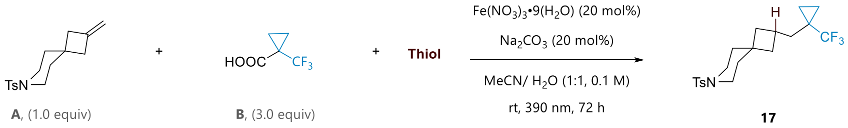

Thiol	Yield of 17
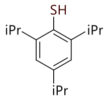 TRIP thiol (20 mol%)	44%
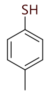 4-tolyl thiol (20 mol%)	34%
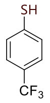 4-CF_3Ph-thiol (20 mol%)	0%
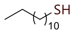 1-Dodecanethiol (20 mol%)	11%

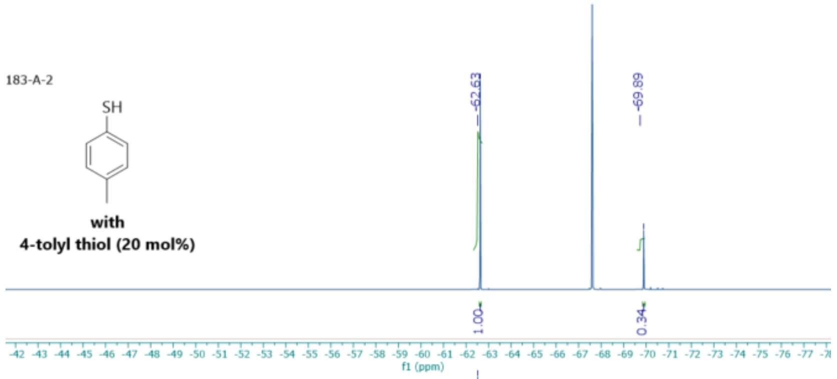

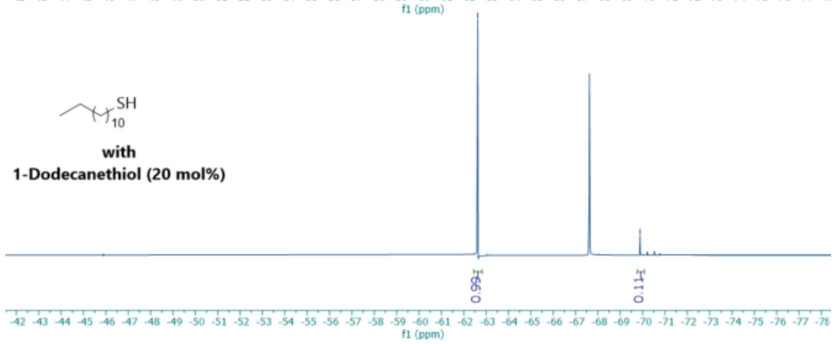

Figure S14: Yields were determined by ^{19}F NMR analysis of the crude with $PhCF_3$ as an external standard.

7. NMR description

■ 5-(1-(Trifluoromethyl)cyclopropyl)pentyl benzoate (1)

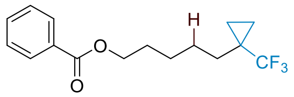

This compound was obtained according to general procedure **A** from pent-4-en-1-yl benzoate (48.0 mg, 0.25 mmol), Isolated as colorless gel (54 mg, 72% yield) after purification by flash column chromatography (4 g SiO_2, hexane/EA = 20:1).

^{1}H NMR (300 MHz, $CDCl_3$): δ 8.10 – 7.96 (m, 2H), 7.63 – 7.49 (m, 1H), 7.49 – 7.38 (m, 2H), 4.32 (t, *J* = 6.6 Hz, 2H), 1.91 – 1.66 (m, 2H), 1.62 – 1.36 (m, 6H), 1.03 – 0.80 (m, 2H), 0.61 – 0.48 (m, 2H).

^{13}C{^{1}H} NMR (101 MHz, $CDCl_3$): δ 166.8, 133.0, 129.7, 128.5, 127.8 (q, $^1J_{C\text{–}F}$ = 273.9 Hz), 65.1, 32.1, 28.8, 26.5 (d, *J* = 2.1 Hz), 22.4 (q, $^2J_{C\text{–}F}$ = 32.1 Hz), 8.9 (q, $^3J_{C\text{–}F}$ = 2.8 Hz).

^{19}F NMR (282 MHz, $CDCl_3$): δ -69.67 (-CF_3).

HRMS (ESI) m/z Calculated for $C_{16}H_{19}O_2F_3Na$ [M+Na]$^+$: 323.1229; found: 323.1225.

■ 5-(1-(Trifluoromethyl)cyclopropyl)pentyl 4-methoxybenzoate (2)

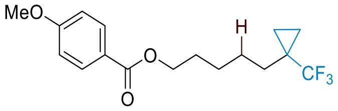

This compound was obtained according to general procedure **A** from pent-4-en-1-yl 4-methoxybenzoate (44.0 mg, 0.20 mmol). Purification was accomplished via flash column chromatography with appropriate eluents (EtOAc : hexane = 1 : 100) to give the product as a colorless gel (38 mg) in 57% yield.

^{1}H NMR (600 MHz, $CDCl_3$): δ 7.99 (dt, *J* = 8.9, 2.4 Hz, 2H), 6.92 (dt, *J* = 8.9, 2.4 Hz, 2H), 4.28 (t, *J* = 6.6 Hz, 2H), 3.86 (s, 3H), 1.76 (quin, *J* = 7.1 Hz, 2H), 1.59–1.40 (m, 6H), 0.94–0.92 (m, 2H), 0.58–0.52 (m, 2H).

^{13}C{^{1}H} NMR (150 MHz, $CDCl_3$): δ 166.4, 163.3, 131.5, 127.5 (q, $^1J_{C\text{–}F}$ = 273 Hz), 122.8, 113.5, 64.6, 55.4, 31.9, 28.6, 26.3, 22.2 (q, $^2J_{C\text{–}F}$ = 33 Hz), 21.6, 8.7 (q, $^3J_{C\text{–}F}$ = 3 Hz).

^{19}F NMR (564 MHz, $CDCl_3$): δ -69.66 (-CF_3).

HRMS (ESI) m/z Calculated for $C_{17}H_{21}F_3O_3$ [M+H]$^+$: 331.1516; found: 331.1510.

■ 6-(1-(Trifluoromethyl)cyclopropyl)hexyl benzoate (3)

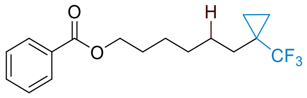

This compound was obtained according to general procedure **A** from hex-5-en-1-yl benzoate (51.0 mg, 0.25 mmol), Isolated as colorless gel (44 mg, 56% yield) after purification by flash column chromatography (4 g SiO_2, hexane/EA = 20:1).

^{1}H NMR (300 MHz, $CDCl_3$): δ 8.16 – 7.91 (m, 2H), 7.64 – 7.49 (m, 1H), 7.49 – 7.37 (m, 2H), 4.31 (t, *J* = 6.6 Hz, 2H), 1.85 – 1.63 (m, 2H), 1.60 – 1.24 (m, 9H), 0.97 – 0.82 (m, 2H).

$^{13}C\{^1H\}$ NMR (75 MHz, $CDCl_3$): δ 166.8, 133.0, 129.7, 129.6 (q, ^1J = 273.7 Hz), 128.5, 65.1, 32.0, 29.6, 28.8, 26.6, 26.1, 22.4 (q, J = 32.1 Hz), 8.8 (q, J = 2.8 Hz).

^{19}F NMR (282 MHz, $CDCl_3$): δ -69.68.

HRMS (ESI) m/z Calculated for $C_{17}H_{21}O_2F_3Na$ $[M+H]^+$: 337.1385; found: 337.1383.

■ **3-Methyl-4-(1-(trifluoromethyl)cyclopropyl)butyl benzoate (4)**

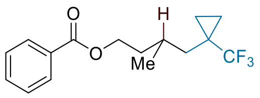

The titled compound was obtained according to general procedure **A** from 3-methylbut-3-en-1-yl benzoate (38.0 mg, 0.20 mmol). Purification was accomplished via flash column chromatography with appropriate eluents (EtOAc : hexane = 1 : 100) to give the product as a colorless gel (36 mg) in 60% yield.

^1H NMR (600 MHz, $CDCl_3$): δ 8.04–8.03 (m, 2H), 7.56 (t, J = 7.4 Hz, 1H), 7.44 (t, J = 7.5 Hz, 2H), 4.40–4.33 (m, 2H), 2.02–1.92 (m, 2H), 1.75 (dd, J = 14.8, 6.4 Hz, 1H), 1.54–1.48 (m, 1H), 1.33 (dd, J = 14.7, 8.3 Hz, 1H), 1.04–0.98 (m, 5H), 0.62–0.52 (m, 2H).

$^{13}C\{^1H\}$ NMR (150 MHz, $CDCl_3$): δ 166.7, 132.9, 130.4, 129.5, 128.4, 127.49 (q, $^1J_{C–F}$= 273 Hz), 63.1, 40.1, 35.7, 28.4, 20.4 (q, $^2J_{C–F}$ = 31.5 Hz), 19.9, 9.4 (d, $^3J_{C–F}$= 3 Hz).

^{19}F NMR (564 MHz, $CDCl_3$): δ -69.05 (-**CF_3**).

HRMS $[M]^+$ = Calculated for $C_{16}H_{19}F_3O_2$ $[M+H]^+$: 301.1410; found: 301.1407.

■ **3-Methyl-4-(1-(trifluoromethyl)cyclopropyl)butyl 4-methoxybenzoate (5)**

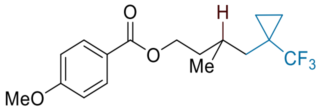

The titled compound was obtained according to general procedure **A** from 3-methylbut-3-en-1-yl 4-methoxybenzoate (44.0 mg, 0.20 mmol). Purification was accomplished via flash column chromatography with appropriate eluents (EtOAc : hexane = 1 : 100) to give the product as a colorless gel (53 mg) in 80% yield.

^1H NMR (600 MHz, $CDCl_3$): δ 8.00–7.97 (m, 2H), 6.93–6.91 (m, 2H), 4.37–4.29 (m, 2H), 3.86 (s, 3H), 2.02–1.89 (m, 2H), 1.75 (dd, J = 14.7, 6.4 Hz, 1H), 1.51–1.49 (m, 1H), 1.31 (dd, J = 14.7, 8.3 Hz, 1H), 1.03–0.96 (m, 5H), 0.62–0.51 (m, 2H).

$^{13}C\{^1H\}$ NMR (150 MHz, $CDCl_3$): δ 166.4, 163.3, 131.5, 127.0(q, $^1J_{C–F}$= 273 Hz), 122.8, 113.6, 62.7, 55.4, 40.1, 35.8, 28.4, 20.4 (q, $^2J_{C–F}$ = 31.5 Hz), 19.9, 9.3 (d, $^3J_{C–F}$= 3 Hz).

^{19}F NMR (564 MHz, $CDCl_3$): δ -69.05 (-**CF_3**).

HRMS (ESI) m/z Calculated for $C_{17}H_{21}F_3O_3$ $[M+Na]^+$: 353.1335; found: 353.1329.

■ **3-Methyl-4-(1-(trifluoromethyl)cyclopropyl)butyl 4-(trifluoromethyl)benzoate (6)**

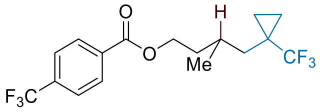

The titled compound was obtained according to general procedure **A** from 3-methylbut-3-en-1-yl 4-(trifluoromethyl)benzoate (52.0 mg, 0.20 mmol). Purification was accomplished via flash column chromatography with appropriate eluents (EtOAc : hexane = 1 : 100) to give the product as a colorless gel (52 mg) in 70% yield.

^{1}H NMR (600 MHz, $CDCl_3$) δ 8.14 (d, J = 8.1 Hz, 2H), 7.70 (d, J = 8.1 Hz, 2H), 4.44–4.36 (m, 2H), 2.04–1.94 (m, 2H), 1.74 (dd, J = 14.7, 6.4 Hz, 1H), 1.54–1.49 (m, 1H), 1.34 (dd, J = 14.6, 8.2 Hz, 1H), 1.04–0.97 (m, 5H), 0.62–0.51 (m, 2H).

^{13}C{^{1}H} NMR (150 MHz, $CDCl_3$) δ 165.4, 134.4 (q, $^{2}J_{C–F}$= 33 Hz), 133.6, 129.9, 127.5 (q, $^{1}J_{C–F}$= 273 Hz), 125.4 (q, $^{3}J_{C–F}$= 3.0 Hz), 123.6 (q, $^{1}J_{C–F}$= 271 Hz), 63.6, 40.1, 35.6, 28.3, 20.4 (q, $^{2}J_{C–F}$ = 31.5 Hz), 19.8, 9.35(d, $^{3}J_{C–F}$= 3 Hz).

^{19}F NMR (564 MHz, $CDCl_3$): δ -63.15 (-ArC**F**$_3$), -69.02 (-C**F**$_3$).

HRMS (ESI) m/z Calculated for $C_{17}H_{18}F_6O_2$ $[M+H]^+$: 369.1284; found: 369.1263.

■ **3-Methyl-4-(1-(trifluoromethyl)cyclopropyl)butyl 4-(chloromethyl)benzoate (7)**

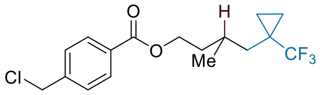

The titled compound was obtained according to general procedure **A** from 3-methylbut-3-en-1-yl 4-(chloromethyl)benzoate (48.0 mg, 0.20 mmol). Purification was accomplished via flash column chromatography with appropriate eluents (EtOAc : hexane = 1 : 100) to give the product as a colorless gel (55 mg) in 79% yield.

^{1}H NMR (600 MHz, $CDCl_3$) δ 8.02 (d, J = 7.6 Hz, 2H), 7.46 (d, J = 7.6 Hz, 2H), 4.62 (s, 2H), 4.41–4.33 (m, 2H), 2.02–1.92 (m, 2H), 1.75 (dd, J = 14.7, 6.4 Hz, 1H), 1.54–1.48 (m, 1H), 1.33 (dd, J = 14.7, 8.3 Hz, 1H), 1.03–0.97 (m, 5H), 0.61–0.52 (m, 2H).

^{13}C{^{1}H} NMR (150 MHz, $CDCl_3$) δ 166.1, 142.2, 130.3, 130.0, 128.5, 127.5 (q, $^{1}J_{C–F}$= 273 Hz), 63.2, 45.4, 40.1, 35.7, 28.4, 20.4 (q, $^{2}J_{C–F}$ = 33 Hz), 19.9, 9.4 (d, $^{3}J_{C–F}$= 3 Hz).

^{19}F NMR (564 MHz, $CDCl_3$): δ -69.02 (-C**F**$_3$).

HRMS (ESI) m/z Calculated for $C_{17}H_{20}ClF_3O_2$ $[M+H]^+$: 349.1169; found: 349.1177.

■ **(4-(1-(Trifluoromethyl)cyclopropyl)butyl)benzene (8)**

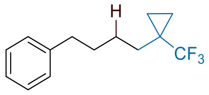

The titled compound was obtained according to general procedure **A** from but-3-en-1-ylbenzene (48.0 mg, 0.20 mmol). Purification was accomplished via flash column chromatography with appropriate eluents (EtOAc : hexane = 1 : 100) to give the product as a colorless gel (16 mg) in 33% yield.

^{1}**H NMR** (600 MHz, $CDCl_3$) δ 7.30–7.27 (m, 2H), 7.20–7.17 (m, 3H), 2.62 (t, J = 7.7 Hz, 2H), 1.64–1.57 (m, 4H), 1.50–1.46 (m, 2H), 0.93–0.91 (m, 2H), 0.57–0.52 (m, 2H).

^{13}**C{^{1}H} NMR** (150 MHz, $CDCl_3$) δ 142.4, 128.33, 128,29, 127.7(q, $^{1}J_{C\text{–}F}$= 273 Hz), 125.7, 35.8, 31.7, 31.6, 26.2, 22.2 (q, $^{2}J_{C\text{–}F}$ = 31.5 Hz), 8.67 (d, $^{3}J_{C\text{–}F}$= 3 Hz).

^{19}**F NMR** (564 MHz, $CDCl_3$): δ -69.69 (-**C**F_3).

■ 9-(1-(Trifluoromethyl)cyclopropyl)nonyl acetate (9)

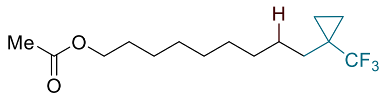

The titled compound was obtained according to general procedure **A** from non-8-en-1-yl acetate (40.0 mg, 0.20 mmol). Purification was accomplished via flash column chromatography with appropriate eluents (EtOAc : hexane = 1 : 100) to give the product as a colorless gel (26 mg) in 42% yield.

^{1}**H NMR** (600 MHz, $CDCl_3$): δ 4.04 (t, J = 6.8 Hz, 2H), 2.04 (s, 3H), 1.63–1.51 (m, 4H), 1.31–1.23 (m, 14H), 0.91–0.89 (m, 2H), 0.54–0.52 (m, 2H).

^{13}**C{^{1}H} NMR** (150 MHz, $CDCl_3$): δ 171.2, 127.7 (q, $^{1}J_{C\text{–}F}$= 272 Hz), 64.4, 31.9, 29.8, 29.44, 29.40, 29.2, 28.6, 26.5, 25.9, 22.2 (q, $^{2}J_{C\text{–}F}$ = 31.6 Hz), 21.0, 8.63 (d, $^{3}J_{C\text{–}F}$= 2.8 Hz);

^{19}**F NMR** (564 MHz, $CDCl_3$): δ -69.75 (-**C**F_3).

HRMS (ESI) m/z Calculated for $C_{15}H_{25}F_3O_2$ $[M+Na]^+$: 317.1699; found: 317.1688.

■ 5-(1-(Trifluoromethyl)cyclopropyl)pentyl 4-methylbenzenesulfonate (10)

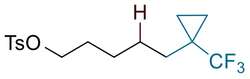

The titled compound was obtained according to general procedure **A** from pent-4-en-1-yl 4-methylbenzenesulfonate (48.0 mg, 0.20 mmol). Purification was accomplished via flash column chromatography with appropriate eluents (EtOAc : hexane = 1 : 100) to give the product as a colorless gel (20 mg) in 28% yield.

^{1}**H NMR** (600 MHz, $CDCl_3$): δ 7.78 (d, J = 8.3 Hz, 2H), 7.34 (d, J = 8.0 Hz, 2H), 4.01 (t, J = 6.4 Hz, 2H), 2.45 (s, 3H), 1.66–1.61 (m, 2H), 1.49–1.46 (m, 2H), 1.37–1.26 (m, 4H).

^{13}**C{^{1}H} NMR** (150 MHz, $CDCl_3$): δ 144.7, 133.1, 129.8, 127.8, 127.5 (q, $^{1}J_{C\text{–}F}$= 273 Hz), 70.3, 31.8, 28.6, 25.9, 25.5, 22.1 (q, $^{2}J_{C\text{–}F}$ = 32 Hz), 21.6, 8.7 (d, $^{3}J_{C\text{–}F}$= 3 Hz).

^{19}**F NMR** (564 MHz, $CDCl_3$): δ -69.65 (-**C**F_3).

HRMS (ESI) m/z Calculated for $C_{16}H_{21}F_3O_3S$ $[M+Na]^+$: 373.1056; found: 373.1045.

■ **2-(4-(1-(Trifluoromethyl)cyclopropyl)butyl)isoindoline-1,3-dione (11)**

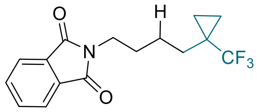

The titled compound was obtained according to general procedure **A** from 2-(but-3-en-1-yl)isoindoline-1,3-dione (40.0 mg, 0.20 mmol). Purification was accomplished via flash column chromatography with appropriate eluents (EtOAc : hexane = 1 : 100) to give the product as a colorless gel (35 mg) in 56% yield.

^{1}H NMR (600 MHz, $CDCl_3$): δ 7.83 (dd, J = 5.4, 3.0 Hz, 2H), 7.70 (dd, J = 5.4, 3.0 Hz, 2H), 3.66 (t, J = 7.3 Hz, 2H), 1.68–1.63 (m, 2H), 1.60–1.57 (m, 2H), 1.50–1.45 (m, 2H), 0.92–0.90 (m, 2H), 0.57–0.52 (m, 2H).

^{13}C{^{1}H} NMR (150 MHz, $CDCl_3$): δ 168.3, 133.9, 132.1, 127.5 (q, $^1J_{C–F}$= 271 Hz), 123.2, 37.5, 31.5, 28.6, 23.8, 22.1 (q, $^2J_{C–F}$ = 33 Hz), 8.76–8.75 (m).

^{19}F NMR (564 MHz, $CDCl_3$): δ -69.67 (-C**F**$_3$).

HRMS (ESI) m/z Calculated for $C_{16}H_{16}F_3NO_2$ $[M+H]^+$: 312.1206; found: 312.1200.

■ **2-(3-Methyl-4-(1-(trifluoromethyl)cyclopropyl)butyl)isoindoline-1,3-dione (12)**

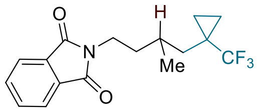

The titled compound was obtained according to general procedure **A** from 2-(3-methylbut-3-en-1-yl)isoindoline-1,3-dione (48.0 mg, 0.20 mmol). Purification was accomplished via flash column chromatography with appropriate eluents (EtOAc : hexane = 1 : 100) to give the product as a colorless gel (47 mg) in 72% yield.

^{1}H NMR (600 MHz, $CDCl_3$) δ 7.83 (dd, J = 5.4, 3,0 Hz, 2H), 7.70 (dd, J = 5.4, 3.0 Hz, 2H), 3.70 (t, J = 7.4 Hz, 2H), 1.84–1.78 (m, 2H), 1.72 (dd, J = 14.7, 6.1 Hz, 1H), 1.44–1.39 (m, 1H), 1.29 (dd, J = 14.7, 8.0 Hz, 1H), 1.04 (d, J = 6.4 Hz, 3H), 0.99–0.93 (m, 2H), 0.59–0.50 (m, 2H).

^{13}C{^{1}H} NMR (150 MHz, $CDCl_3$) δ 168.3, 133.8, 132.1, , 127.4 (q, $^1J_{C–F}$= 273 Hz), 123.1, 39.8, 36.0, 35.5, 29.1, 20.3 (q, $^2J_{C–F}$ = 31.5 Hz), 19.7, 9.35 (d, $^3J_{C–F}$= 2.2 Hz).

^{19}F NMR (564 MHz, $CDCl_3$): δ -69.09 (-C**F**$_3$).

HRMS (ESI) m/z Calculated for $C_{17}H_{18}F_3NO_2$ $[M+Na]^+$: 348.1182; found: 348.1179.

■ **3-Methyl-4-(1-(trifluoromethyl)cyclopropyl)butyl furan-2-carboxylate (13)**

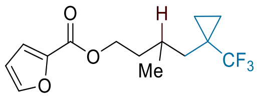

The titled compound was obtained according to general procedure **A** from 3-methylbut-3-en-1-yl furan-2-carboxylate (36.0 mg, 0.20 mmol). Purification was accomplished via flash column chromatography with appropriate eluents (EtOAc : hexane = 1 : 100) to give the product as a colorless gel (17 mg) in 29% yield.

^{1}H NMR (600 MHz, $CDCl_3$) δ 7.57 (m, 1H), 7.15 (d, J = 3.4 Hz, 1H), 6.50 (dd, J = 3.4, 1.7 Hz, 1H), 4.37–4.31 (m, 2H), 1.97–1.88 (m, 2H), 1.72 (dd, J = 14.7, 8.2 Hz, 1H), 1.52–1.46 (m, 1H), 1.31 (dd, J = 14.7, 8.2 Hz, 1H), 1.01–0.98 (m, 5H), 0.61–0.51 (m, 2H).

^{13}C{^{1}H} NMR (150 MHz, $CDCl_3$) δ 158.8, 146.3, 144.8, 127.0 (q, $^{1}J_{C–F}$= 273 Hz), 117.8, 111.8, 63.0, 40.0, 35.2, 28.3, 20.4 (q, $^{2}J_{C–F}$ = 32 Hz), 19.8, 9.3 (d, $^{3}J_{C–F}$= 2.2 Hz).

^{19}F NMR (564 MHz, $CDCl_3$): δ -69.08 (-CF_3).

HRMS (ESI) m/z Calculated for $C_{14}H_{17}F_3O_3$ $[M+Na]^+$: 313.1022; found: 313.1017.

■ 2-(1-(Trifluoromethyl)cyclopropyl)ethyl benzoate (14)

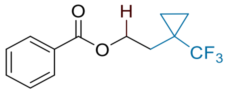

The titled compound was obtained according to general procedure **A** from vinyl benzoate (48.0 mg, 0.20 mmol). Purification was accomplished via flash column chromatography with appropriate eluents (EtOAc : hexane = 1 : 100) to give the product as a colorless gel (12 mg) in 23% yield.

^{1}H NMR (600 MHz, $CDCl_3$) δ 8.02 (d, J = 8.4 Hz, 2H), 7.57 (t, J = 7.4 Hz, 1H), 7.45 (t, J = 7.9 Hz, 2H), 4.46 (t, J = 7.1 Hz, 2H), 2.05 (t, J = 7.1 Hz, 2H), 1.05–1.03 (m, 2H), 0.74–0.70 (m, 2H).

^{13}C{^{1}H} NMR (150 MHz, $CDCl_3$) δ 166.4, 133.0, 130.1, 129.5, 128.4, 127.2(q, $^{1}J_{C–F}$= 273 Hz), 62.5, 31.1, 20.2 (q, $^{2}J_{C–F}$ = 33 Hz), 8.88 (d, $^{3}J_{C–F}$= 3 Hz).

^{19}F NMR (564 MHz, $CDCl_3$): δ -69.76 (-CF_3).

HRMS (ESI) m/z Calculated for $C_{13}H_{13}F_3O_2$ $[M+NH_4]^+$: 276.0895; found: 276.1206.

■ Benzyl 4-((1-(trifluoromethyl)cyclopropyl)methyl)piperidine-1-carboxylate (15)

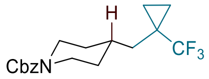

The titled compound was obtained according to general procedure **A** from benzyl 4-methylenepiperidine-1-carboxylate (46.0 mg, 0.20 mmol). Purification was accomplished via flash column chromatography with appropriate eluents (EtOAc : hexane = 1 : 100) to give the product as a colorless gel (23 mg) in 32% yield.

^{1}H NMR (600 MHz, $CDCl_3$) δ 7.37–7.29 (m, 5H), 5.12 (s, 2H), 4.18 (d, J = 11.5 Hz, 2H), 2.77 (t, J = 12.6 Hz, 2H), 1.82–1.80 (m, 3H), 1.47 (d, J = 7.0 Hz, 2H), 1.06–0.97 (m, 4H), 0.54–0.51 (m, 2H).

^{13}C{^{1}H} NMR (150 MHz, $CDCl_3$) δ 155.3, 137.0, 128.5, 127.9, 127.8, 127.4 (q, $^{1}J_{C–F}$= 273 Hz), 67.0, 44.1, 39.8, 34.4, 32.5, 19.9 (q, $^{2}J_{C–F}$ = 31 Hz), 9.31 (d, $^{3}J_{C–F}$= 3.0 Hz).

^{19}F NMR (564 MHz, $CDCl_3$): δ -68.94 (-CF_3).

HRMS (ESI) m/z Calculated for $C_{18}H_{22}F_3NO_2$ $[M+Na]^+$: 364.1495; found: 364.1492.

■ 1-Tosyl-3-((1-(trifluoromethyl)cyclopropyl)methyl)azetidine (16)

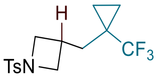

The titled compound was obtained according to general procedure **A** from 3-methylene-1-tosylazetidine (45.0 mg, 0.20 mmol). Purification was accomplished via flash column chromatography with appropriate eluents (EtOAc : hexane = 1 : 100) to give the product as a colorless gel (20 mg) in 30% yield.

. **^{1}H NMR** (600 MHz, $CDCl_3$) δ 7.72 (d, *J* = 7.9 Hz, 2H), 7.37 (d, *J* = 7.9 Hz, 2H), 3.86 (t, *J* = 8.1 Hz, 2H), 3.43 (t, *J* = 7.0 Hz, 2H), 2.63–2.58 (m, 1H), 2.47 (s, 3H), 1.61 (d, *J* = 7.4 Hz, 2H), 0.92–0.90 (m, 2H), 0.50–0.43 (m, 2H).

^{13}C{^{1}H} NMR (150 MHz, $CDCl_3$) δ 144.1, 131.6, 129.7, 128.3, 126.9 (q, $^1J_{C\text{–}F}$= 273 Hz), 55.9, 36.2, 26.8, 21.6, 21.0 (q, $^2J_{C\text{–}F}$ = 31 Hz), 8.63 (d, $^3J_{C\text{–}F}$= 3.0 Hz).

^{19}F NMR (564 MHz, $CDCl_3$): δ -69.69 (-C**F**$_3$).

HRMS (ESI) m/z Calculated for $C_{15}H_{18}F_3NO_2S$ $[M+H]^+$: 334.1083; found: 334.1072.

■ 7-Tosyl-2-((1-(trifluoromethyl)cyclopropyl)methyl)-7-azaspiro[3.5]nonane (17)

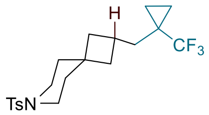

The titled compound was obtained according to general procedure **A** from 2-methylene-7-tosyl-7-azaspiro[3.5]nonane (54.0 mg, 0.20 mmol). Purification was accomplished via flash column chromatography with appropriate eluents (EtOAc : hexane = 1 : 100) to give the product as a colorless gel (35 mg) in 44% yield.

^{1}H NMR (600 MHz, $CDCl_3$) δ 7.62 (d, *J* = 7.8 Hz, 2H), 7.31 (d, *J* = 7.8 Hz, 2H), 2.96–2.94 (m, 2H), 2.86–2.85 (m, 2H), 2.44 (s, 3H), 2.29 (1H), 1.88 (t, *J* = 10.5 Hz, 2H), 1.69 (t, *J* = 5.4 Hz, 2H), 1.63 (d, *J* = 7.3 Hz, 2H), 1.56–1.55 (m, 2H), 1.28 (t, *J* = 10.5 Hz, 2H), 0.86–0.84 (m, 2H), 0.51–0.46 (m, 2H).

^{13}C{^{1}H} NMR (150 MHz, $CDCl_3$) δ 143.3, 133.3, 129.6, 127.6, 127.3 (q, $^1J_{C\text{–}F}$= 273 Hz), 43.2, 42.9, 39.2, 39.0, 38.5, 35.4, 33.4, 26.5, 21.5, 21.3 (q, $^2J_{C\text{–}F}$ = 33 Hz), 8.13 (d, $^3J_{C\text{–}F}$= 3.0 Hz).

^{19}F NMR (564 MHz, $CDCl_3$): δ -69.98 (-C**F**$_3$).

HRMS (ESI) m/z Calculated for $C_{20}H_{26}F_3NO_2S$ $[M+H]^+$: 402.1709; found: 420.1700.

■ 1-(tert-Butyl)-4-(2-(1-(trifluoromethyl)cyclopropyl)ethyl)benzene (18)

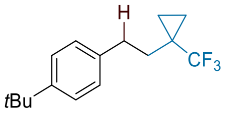

This compound was obtained according to general procedure **1** from 1-(tert-butyl)-4-vinylbenzene (85.0 µL, 0.25 mmol), Isolated as a transparent gel (33 mg, 48% yield) after purification by flash column chromatography (4 g SiO_2, hexane/EA = 20:1).

^{1}H NMR (400 MHz, $CDCl_3$): δ 7.32 – 7.28 (m, 2H), 7.13 – 7.08 (m, 2H), 2.74 – 2.70 (m, 2H), 1.88 – 1.83 (m, 2H), 1.31 (s, 9H), 1.00 – 0.96 (m, 2H), 0.60 – 0.57 (m, 2H).

^{13}C NMR (101 MHz, $CDCl_3$): δ 149.0, 138.7, 128.1, 127.5 (q, *J* = 289.0 Hz), 125.5, 34.4, 32.6, 31.6, 24.6, 22.4 (q, *J* = 43.4 Hz), 9.1 (q, *J* = 2.6 Hz).

^{19}F NMR (282 MHz, $CDCl_3$): δ -69.35.

HRMS (GC-EI) m/z Calculated for $C_{16}H_{21}F_3$ $[M]^+$: 270.1590; found: 270.1592.

■ 3-Methyl-4-(1-(trifluoromethyl)cyclopropyl)butyl-(2S)-2-(6-methoxynaphthalen-2-yl)-propanoate (19)

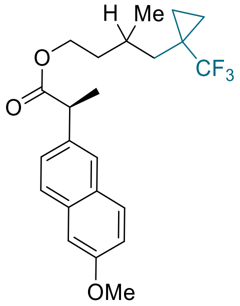

The titled compound was obtained according to general procedure **A** from 3-methylbut-3-en-1-yl (S)-2-(6-methoxynaphthalen-2-yl)propanoate (62.0 mg, 0.20 mmol). Purification was accomplished via flash column chromatography with appropriate eluents (EtOAc : hexane = 1 : 100) to give the product as a colorless gel (20 mg) in 26% yield.

^{1}H NMR (600 MHz, $CDCl_3$): δ 7.70 (d, J = 8.7 Hz, 2H), 7.66 (s, 1H), 7.40 (d, J = 8.7 Hz, 1H), 7.17 – 7.08 (m, 2H), 4.11 (tt, J = 12.8, 5.9 Hz, 2H), 3.91 (s, 3H), 3.84 (q, J = 7.0 Hz, 1H), 1.84 – 1.62 (m, 4H), 1.58 (d, J = 6.7 Hz, 4H), 1.32 (dt, J = 13.5, 6.8 Hz, 1H), 1.16 (dd, J = 14.4, 8.7 Hz, 1H), 1.05 – 0.72 (m, 5H).0.52 – 0.46 (m, 1H), 0.42 – 0.34 (m, 1H).

^{13}C{^{1}H} NMR (150 MHz, $CDCl_3$): 174.8, 157.7, 135.9, 133.8, 129.4, 127.3, 126.6 (q, $^{1}J_{C\text{-}F}$= 274.0 Hz), 126.4, 126.4, 126.1, 119.1, 105.7, 63.0, 62.9, 55.4, 45.7, 39.9, 39.8, 35.8, 28.3, 20.4 (q, $^{2}J_{C\text{-}F}$ = 32.0 Hz), 19.8 (d, $^{3}J_{C\text{-}F}$ = 2.6 Hz), 18.5, 9.6, 9.2.

^{19}F NMR (564 MHz, $CDCl_3$): δ 69.15(-C**F**$_3$), -69.19 (-C**F**$_3$ of its diastereomer).

HRMS (ESI) m/z Calculated for $C_{23}H_{27}F_3O_3$ $[M+H]^+$: 409.1985; found: 409.1983.

■ 1-(4-(tert-Butyl)phenethyl)cyclopropane-1-carbonitrile (20)

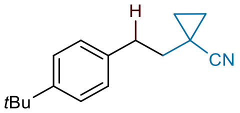

This compound was obtained according to general procedure **B** from 1-(tert-butyl)-4-vinylbenzene (45.0 μL, 0.25 mmol), Isolated as transparent gel (30 mg, 53% yield) after purification by flash column chromatography (4 g SiO_2, hexane/EA = 20:1).

^{1}H NMR (300 MHz, $CDCl_3$): δ 7.36 – 7.28 (m, 2H), 7.17 – 7.08 (m, 2H), 2.93 – 2.81 (m, 2H), 1.83 – 1.66 (m, 2H), 1.31 (s, 9H), 1.24 – 1.15 (m, 2H), 0.77 – 0.67 (m, 2H).

^{13}C NMR (75 MHz, $CDCl_3$): δ 149.3, 137.5, 128.2, 125.5, 37.5, 34.5, 33.5, 31.5, 14.1, 9.8.

HRMS (ESI) m/z Calculated for $C_{16}H_{21}NNa$ $[M+Na]^+$: 250.1566; found: 250.1563.

■ 2-(1-Cyanocyclopropyl)ethyl 4-(tert-butyl)benzoate (21)

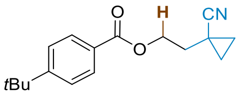

This compound was obtained according to general procedure **B** from vinyl 4-(tert-butyl)benzoate (51.0 μL, 0.25 mmol), Isolated as transparent gel (42 mg, 62% yield) after purification by flash column chromatography (4 g SiO_2, hexane/EA = 20:1).

^{1}H NMR (300 MHz, $CDCl_3$): δ 8.06 – 7.96 (m, 1H), 7.53 – 7.41 (m, 1H), 4.52 (t, *J* = 6.2 Hz, 2H), 1.94 (t, *J* = 6.2 Hz, 2H), 1.38 – 1.26 (m, 11H), 0.97 – 0.86 (m, 2H).

^{13}C NMR (75 MHz, $CDCl_3$): δ 166.6, 157.0, 129.7, 127.2, 125.6, 123.1, 62.6, 35.2, 34.4, 31.2, 14.0, 7.5.

HRMS (ESI) m/z Calculated for $C_{17}H_{21}O_2NNa$ $[M+Na]^+$: 294.1464; found: 294.1458.

■ 5-(1-Cyanocyclopropyl)pentyl 4-methoxybenzoate (22)

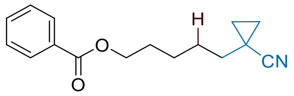

The titled compound was obtained according to general procedure **B** from pent-4-en-1-yl benzoate (19.0 mg, 0.10 mmol). Purification was accomplished via flash column chromatography with appropriate eluents (EtOAc : hexane = 1 : 20) to give the product as a colorless gel (18 mg) in 72% yield.

^{1}H NMR (600 MHz, $CDCl_3$): δ 8.04 (d, *J* = 8.1 Hz, 2H), 7.55 (t, *J* = 7.4 Hz, 1H), 7.44 (t, *J* = 7.6 Hz, 2H), 4.33 (t, *J* = 6.6 Hz, 2H), 1.82–1.77 (m, 2H), 1.68–1.66 (m, 2H), 1.52–1.46 (m, 4H), 1.22–1.20 (m, 2H), 0.78–0.76 (m, 2H).

^{13}C NMR (150 MHz, $CDCl_3$): δ 166.6, 132.9, 130.3, 129.5, 128.3, 123.5, 64.7, 35.1, 28.6, 27.4, 25.6, 13.9, 9.7.

HRMS (ESI) m/z Calculated for $C_{16}H_{19}NO_2$ $[M+H]^+$: 258.1489; found: 258.1482.

■ 5-(1-Cyanocyclopropyl)pentyl 4-methoxybenzoate (23)

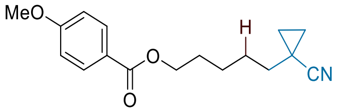

The titled compound was obtained according to general procedure **B** from pent-4-en-1-yl 4-methoxybenzoate (44.0 mg, 0.20 mmol). Purification was accomplished via flash column chromatography with appropriate eluents (EtOAc : hexane = 1 : 20) to give the product as a colorless gel (33 mg) in 57% yield.

^{1}H NMR (600 MHz, $CDCl_3$): δ 7.99 (d, *J* = 9.0 Hz, 2H), 6.91 (d, *J* = 9.0 Hz, 2H), 4.29 (t, *J* = 6.5 Hz, 2H), 3.86 (s, 3H), 1.80–1.76 (m, 2H), 1.68–1.65 (m, 2H), 1.52–1.46 (m, 4H), 1.22–1.20 (m, 2H), 0.78–0.76 (m, 2H).

^{13}C NMR (150 MHz, $CDCl_3$): δ 166.3, 163.2, 131.5, 123.4, 122.7, 113.5, 64.4, 55.3, 35.1, 28.6, 27.4, 25.5, 13.8, 9.7.

HRMS (ESI) m/z Calculated for $C_{17}H_{22}NO_3$ $[M+H]^+$: 288.1594; found: 288.1589.

■ 5-(1-Cyanocyclopropyl)pentyl 4-methylbenzenesulfonate (24)

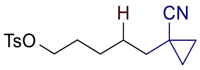

The titled compound was obtained according to general procedure **B** from pent-4-en-1-yl 4-methylbenzenesulfonate (0.20 mmol). Purification was accomplished via flash column chromatography with appropriate eluents (EtOAc : hexane = 1 : 20) to give the product as a colorless gel (36 mg) in 59% yield.

^{1}H NMR (600 MHz, $CDCl_3$): δ 7.79 (d, *J* = 8.0 Hz, 2H), 7.35 (d, *J* = 8.0 Hz, 2H), 4.03 (t, *J* = 6.4 Hz, 2H), 2.45 (s, 3H), 1.69–1.64 (m, 2H), 1.54–1.53 (m, 2H), 1.41–1.35 (m, 4H), 1.21–1.18 (m, 2H), 0.76–0.74 (m, 2H).

^{13}C NMR (150 MHz, $CDCl_3$): δ 144.7, 133.0, 129.8, 127.8, 123.3, 70.2, 35.0, 28.6, 27.0, 24.8, 21.6, 13.8, 9.6.

HRMS (ESI) m/z Calculated for $C_{16}H_{21}NO_3S$ $[M+H]^+$: 308.1315; found: 308.1308.

■ **1-(5-(1,3-Dioxoisoindolin-2-yl)pentyl)cyclopropane-1-carbonitrile (25)**

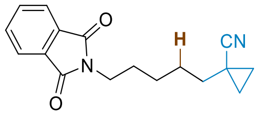

This compound was obtained according to general procedure **B** from 2-(pent-4-en-1-yl)isoindoline-1,3-dione (54 mg, 0.25 mmol), Isolated as pale yellow (42 mg, 59% yield) after purification by flash column chromatography (4 g SiO_2, hexane/EA = 20:1).

^{1}H NMR (300 MHz, $CDCl_3$): δ 7.84 (dd, *J* = 5.5, 3.1 Hz, 2H), 7.71 (dd, *J* = 5.5, 3.0 Hz, 2H), 3.69 (t, *J* = 7.2 Hz, 2H), 1.80 – 1.52 (m, 4H), 1.51 – 1.31 (m, 4H), 1.24 – 1.09 (m, 2H), 0.90 – 0.58 (m, 2H).

^{13}C NMR (75 MHz, $CDCl_3$): δ 168.6, 134.0, 132.3, 123.3, 37.9, 35.2, 28.5, 27.3, 26.4, 14.0, 9.9.

HRMS (ESI) m/z Calculated for $C_{17}H_{18}O_2N_2Na$ $[M+Na]^+$: 305.1260; found: 305.1255.

■ **4-(1-Cyanocyclopropyl)-3-methylbutyl benzoate (26)**

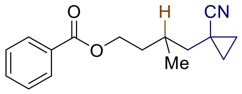

The titled compound was obtained according to general procedure **B** from 3-methylbut-3-en-1-yl benzoate (38.0 mg, 0.20 mmol). Purification was accomplished via flash column chromatography with appropriate eluents (EtOAc : hexane = 1 : 20) to give the product as a colorless gel (35 mg) in 68% yield.

^{1}H NMR (600 MHz, $CDCl_3$): δ 8.05 (d, *J* = 8.4 Hz, 2H), 7.56 (t, *J* = 7.4, 1.3 Hz, 1H), 7.44 (t, *J* = 6.9 Hz, 2H), 4.43–4.34 (m, 2H), 2.14–2.08 (m, 1H), 1.98–1.94 (m, 1H), 1.68–1.60 (m, 2H), 1.31–1.25 (m, 3H), 1.12 (t, *J* = 6.7 Hz, 3H), 0.85–0.78 (m, 2H).

^{13}C NMR (150 MHz, $CDCl_3$): δ 166.6, 132.9, 130.2, 129.5, 128.4, 123.4, 62.8, 42.0, 35.2, 29.8, 19.3, 15.0, 13.8, 8.2.

HRMS (ESI) m/z Calculated for $C_{16}H_{19}NO_2$ $[M+H]^+$: 258.1489; found: 258.1484.

■ **5-(1-Cyanocyclopropyl)pentyl 4-methoxybenzoate (27)**

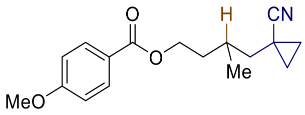

The titled compound was obtained according to general procedure **B** from 3-methylbut-3-en-1-yl 4-methoxybenzoate (44.0 mg, 0.20 mmol). Purification was accomplished via flash column chromatography with appropriate eluents (EtOAc : hexane = 1 : 20) to give the product as a colorless gel (39 mg) in 68% yield.

^{1}H NMR (600 MHz, $CDCl_3$): δ 8.00 (d, *J* = 8.0 Hz, 2H), 6.92 (d, *J* = 8.0 Hz, 2H), 4.93–4.31 (m, 2H), 3.85 (s, 3H), 2.13–2.08 (m, 1H), 1.96–1.91 (m, 1H), 1.67–1.59 (m, 2H), 1.29–1.26 (m, 3H), 1.12 (t, *J* = 6.6 Hz, 3H), 0.85–0.78 (m, 2H).

^{13}C NMR (150 MHz, $CDCl_3$): δ 166.3, 163.3, 131.5, 123.4, 122.6, 113.5, 62.4, 55.3, 41.9, 35.2, 29.7, 19.2, 14.9, 13.8, 8.1.

HRMS (ESI) m/z Calculated for $C_{17}H_{21}NO_3$ $[M+H]^+$: 288.1594; found: 288.1588.

■ 5-(1-Cyanocyclopropyl)pentyl 4-methoxybenzoate (28)

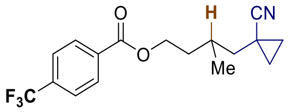

The titled compound was obtained according to general procedure **B** from 3-methylbut-3-en-1-yl 4-(trifluoromethyl)benzoate (52.0 mg, 0.20 mmol). Purification was accomplished via flash column chromatography with appropriate eluents (EtOAc : hexane = 1 : 20) to give the product as a colorless gel **26** (48 mg) in 74% yield.

^{1}H NMR (600 MHz, $CDCl_3$): δ 8.17 (d, *J* = 8.0 Hz, 2H), 7.71 (d, *J* = 8.0 Hz, 2H), 4.46–4.38 (m, 2H), 2.15–2.09 (m, 1H), 2.02–1.97 (m, 1H), 1.67–1.61 (m, 2H), 1.31–1.26 (m, 3H), 1.12 (t, *J* = 6.6 Hz, 3H), 0.85–0.78 (m, 2H).

^{13}C NMR (150 MHz, $CDCl_3$): δ 165.3, 134.3 (q, $^2J_{C–F}$= 33 Hz), 133.4, 129.9, 125.36 (d, $^3J_{C–F}$= 33 Hz), 123.57 (q, $^1J_{C–F}$= 271 Hz), 123.3, 63.3, 41.9, 35.0, 29.7, 19.2, 14.8, 13.9, 8.1.

^{19}F NMR (564 MHz, $CDCl_3$): δ -63.11 (-ArC**F**$_3$).

HRMS (ESI) m/z Calculated for $C_{17}H_{18}F_3NO_2$ $[M+H]^+$: 326.1362; found: 326.1358.

■ 1-(tert-Butyl)-4-(2-(1-phenylcyclopropyl)ethyl)benzene (29)

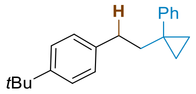

This compound was obtained according to general procedure **B** from 1-(tert-butyl)-4-vinylbenzene (45.0 µL, 0.25 mmol), Isolated as transparent gel (35 mg, 51% yield) after purification by flash column chromatography (4 g SiO_2, hexane/EA = 20:1).

^{1}H NMR (400 MHz, $CDCl_3$): δ 7.40 – 7.17 (m, 7H), 7.03 (d, *J* = 8.3 Hz, 2H), 2.62 – 2.49 (m, 2H), 1.93 – 1.80 (m, 2H), 1.29 (s, 9H), 0.94 – 0.79 (m, 2H), 0.76 – 0.65 (m, 2H).

^{13}C NMR (101 MHz, $CDCl_3$): δ 148.5, 145.2, 139.6, 129.1, 128.3, 128.0, 126.1, 125.3, 34.5, 33.1, 31.6, 25.9, 13.4.

HRMS (EI) m/z Calculated for $C_{20}H_{23}$ [M– CH_3]: 263.1794; found: 263.1799.

■ **2-(2-(1-Phenylcyclopropyl)ethyl)naphthalene (30)**

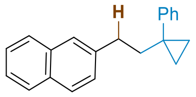

This compound was obtained according to general procedure **B** from 2-vinylnaphthalene (38.5 mg, 0.25 mmol), Isolated as white gel (36 mg, 53% yield) after purification by flash column chromatography (4 g SiO_2, hexane/EA = 20:1).

^{1}H NMR (300 MHz, $CDCl_3$): ^{1}H δ 7.82 – 7.68 (m, 3H), 7.59 – 7.14 (m, 9H), 2.81 – 2.66 (m, 2H), 2.03 – 1.91 (m, 2H), 0.93 – 0.83 (m, 2H), 0.76 – 0.69 (m, 2H).

^{13}C NMR (101 MHz, $CDCl_3$): δ 145.1, 133.8, 129.2, 128.4, 127.9, 127.7, 127.5, 127.5, 126.3, 126.2, 126.0, 125.2, 42.6, 33.9, 26.0, 13.3.

HRMS (EI) m/z Calculated for $C_{20}H_{21}$ $[M]^+$: 263.1794; found: 263.1799.

■ **5-(1-Fluorocyclopropyl)pentyl benzoate (31)**

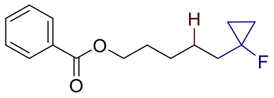

The titled compound was obtained according to general procedure **B** from pent-4-en-1-yl benzoate (38.0 mg, 0.20 mmol). Purification was accomplished via flash column chromatography with appropriate eluents (EtOAc : hexane = 1:20) to give the product as a colorless gel (9 mg) in 18% yield.

^{1}H NMR (600 MHz, $CDCl_3$): δ 8.14 (d, J = 8.1 Hz, 2H), 7.70 (d, J = 8.2 Hz, 2H), 4.44 – 4.36 (m, 2H), 2.04 – 1.94 (m, 2H), 1.73 (dd, J = 14.7, 6,4 Hz, 1H), 1.54 – 1.49 (m, 1H), 1.34 (dd, J = 14.6, 8.2 Hz, 1H), 1.04 – 0.97 (m, 5H), 0.62 – 0.51 (m, 2H).

^{13}C NMR (150 MHz, $CDCl_3$): δ 166.7, 132.8, 130.4, 129.5, 128.3, 79.2 (d, J = 214.5 Hz), 65.0, 34.9 (d, J = 21.1 Hz), 28.7, 25.9, 25.3, 10.5, 10.4.

^{19}F NMR (565 MHz, $CDCl_3$): δ -181.6 (ttt, J = 28.5, 19.0, 9.2 Hz).

HRMS (ESI) m/z Calculated for $C_{15}H_{19}FO_2$ $[M+H]^+$: 251.1442; found: 251.1438.

■ **4-(1-Fluorocyclopropyl)-3-methylbutyl benzoate (32)**

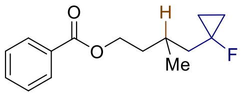

The titled compound was obtained according to general procedure **B** from 3-methylbut-3-en-1-yl benzoate (52.0 mg, 0.20 mmol). Purification was accomplished via flash column chromatography with appropriate eluents (EtOAc : hexane = 1 : 20) to give the product as a colorless gel (12 mg) in 24% yield.

^{1}H NMR (600 MHz, $CDCl_3$): δ 8.04 (dd, J = 8.2, 1.2 Hz, 2H), 7.58 – 7.52 (m, 1H), 7.44 (t, J = 7.8 Hz, 2H), 4.39 (qt, J = 11.0, 6.7 Hz, 2H), 2.16 – 2.05 (m, 1H), 2.02 – 1.93 (m, 1H), 1.89 (ddd, J = 20.8, 14.9, 5.9 Hz, 1H), 1.76 – 1.61 (m, 1H), 1.61 – 1.54 (m, 1H), 1.11 (d, J = 6.7 Hz, 2H), 1.05 – 0.94 (m, 2H), 0.57 – 0.45 (m, 2H).

^{13}C NMR (150 MHz, $CDCl_3$): δ 166.8, 133.0, 130.6, 129.7, 128.5, 78.0 (d, J = 215.0 Hz), 63.4, 42.2 (d, J = 20.5 Hz), 35.7, 28.1, 20.0, 11.3 (d, J = 12.1 Hz), 10.6 (d, J = 12.1 Hz).

^{19}F NMR (564 MHz, $CDCl_3$): δ -180.7–-180.8, -66.4.

HRMS (ESI) m/z Calculated for $C_{15}H_{19}FO_2$ $[M+H]^+$: 251.1442; found: 251.1438.

■ **1-(tert-Butyl)-4-(2-(1-(trifluoromethyl)cyclobutyl)ethyl)benzene (33)**

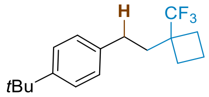

This compound was obtained according to general procedure **B** from 1-(tert-butyl)-4-vinylbenzene (45.0 μL, 0.25 mmol), Isolated as a pale white solid (48 mg, 67% yield) after purification by flash column chromatography (4 g SiO_2, hexane/EA = 20:1).

^{1}H NMR (300 MHz, $CDCl_3$): δ 7.39 – 7.29 (m, 2H), 7.21 – 7.10 (m, 2H), 2.74 – 2.61 (m, 2H), 2.44 – 2.26 (m, 2H), 2.08 – 1.90 (m, 6H), 1.32 (s, 9H).

^{13}C NMR (75 MHz, $CDCl_3$): δ 149.0, 139.1, 128.1, 126.6 (q, J = 280.1 Hz), 125.5, 36.9, 34.5, 31.6, 30.1, 26.1 – 25.6 (m), 15.0.

^{19}F NMR (282 MHz, $CDCl_3$): δ -76.55

HRMS (ESI) m/z Calculated for $C_{17}H_{22}F_3$ $[M-H]^+$: 283.1668; found: 283.1672.

■ **2-(4-(2-(1-(Trifluoromethyl)cyclobutyl)ethyl)benzyl)isoindoline-1,3-dione (34)**

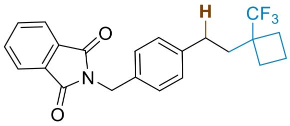

This compound was obtained according to general procedure **B** from 2-(4-vinylbenzyl)isoindoline-1,3-dione (66 mg, 0.25 mmol), Isolated as white solid (69 mg, 71% yield) after purification by flash column chromatography (4 g SiO_2, hexane/EA = 20:1).

^{1}H NMR (300 MHz, $CDCl_3$): δ 7.84 (dd, J = 5.5, 3.1 Hz, 2H), 7.70 (dd, J = 5.5, 3.1 Hz, 2H), 7.38 (d, J = 8.1 Hz, 2H), 7.16 (d, J = 8.1 Hz, 2H), 4.82 (s, 2H), 2.71 – 2.60 (m, 2H), 2.44 – 2.23 (m, 2H), 2.08 – 1.79 (m, 6H).

^{13}C NMR (75 MHz, $CDCl_3$): δ 168.2, 141.8, 134.3, 134.1, 132.3, 129.0, 128.9 (q, J = 279.5 Hz), 123.5, 68.1, 44.8 (q, J = 25.8 Hz), 41.5, 36.9 (d, J = 2.0 Hz), 30.4, 25.9 (q, J = 3.2 Hz), 14.9.

^{19}F NMR (282 MHz, $CDCl_3$): δ -76.53

HRMS (ESI) m/z Calculated for $C_{22}H_{20}O_2NF_3Na$ $[M+Na]^+$: 410.1338; found: 410.1337.

■ **5-(1-(Trifluoromethyl)cyclobutyl)pentyl benzoate (35)**

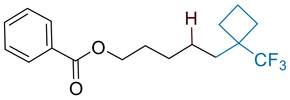

This compound was obtained according to general procedure **B** from pent-4-en-1-yl benzoate (48.0 mg, 0.25 mmol), Isolated as transparent gel (53 mg, 68% yield) after purification by flash column chromatography (4 g SiO_2, hexane/EA = 20:1).

^{1}H NMR (300 MHz, $CDCl_3$): δ 8.32 – 7.90 (m, 2H), 7.69 – 7.51 (m, 1H), 7.49 – 7.38 (m, 2H), 4.34 (t, J = 6.5 Hz, 2H), 2.46 – 2.18 (m, 2H), 2.02 – 1.76 (m, 5H), 1.74 – 1.62 (m, 2H), 1.55 – 1.40 (m, 4H).

^{13}**C NMR** (101 MHz, $CDCl_3$): δ 166.8, 133.0, 130.6, 129.8 (q, J = 280.0 Hz), 129.7, 128.5, 65.1, 44.8 (q, J = 26.3 Hz), 34.6 (q, J = 2.0 Hz), 28.8, 27.0, 25.7 (q, J = 3.3 Hz), 24.0, 14.9.

^{19}**F NMR** (282 MHz, $CDCl_3$): δ -76.89

HRMS (ESI) m/z Calculated for $C_{17}H_{21}O_2F_3Na$ $[M+Na]^+$: 337.1386; found: 337.1386.

■ **5-(1-(Trifluoromethyl)cyclobutyl)pentyl furan-2-carboxylate (36)**

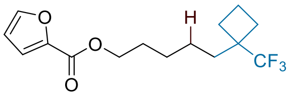

This compound was obtained according to general procedure **B** from pent-4-en-1-yl furan-2-carboxylate (45.0 mg, 0.25 mmol), Isolated as transparent gel (41 mg, 54% yield) after purification by flash column chromatography (4 g SiO_2, hexane/EA = 20:1).

^{1}**H NMR** (300 MHz, $CDCl_3$): δ 7.58 (dd, J = 1.7, 0.9 Hz, 1H), 7.18 (dd, J = 3.5, 0.9 Hz, 1H), 6.51 (dd, J = 3.5, 1.7 Hz, 1H), 4.32 (t, J = 6.7 Hz, 2H), 2.37 – 2.18 (m, 2H), 2.03 – 1.74 (m, 6H), 1.72 – 1.59 (m, 2H), 1.51 – 1.36 (m, 4H).

^{13}**C NMR** (101 MHz, $CDCl_3$): δ 159.0, 146.4, 145.0, 129.2 (q, J = 280.1 Hz), 117.9, 111.9, 65.1, 44.8 (q, J = 25.8 Hz), 28.8, 26.9, 25.8 (q, J = 3.2 Hz), 23.9, 14.9.

^{19}**F NMR** (282 MHz, $CDCl_3$): δ -76.88

HRMS (ESI) m/z Calculated for $C_{15}H_{19}O_3F_3Na$ $[M+Na]^+$: 327.1178; found: 327.1175.

■ **2-(5-(1-(Trifluoromethyl)cyclobutyl)pentyl)isoindoline-1,3-dione (37)**

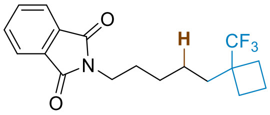

This compound was obtained according to general procedure **B** from 2-(pent-4-en-1-yl)isoindoline-1,3-dione (54.0 mg, 0.25 mmol), Isolated as transperent gel (50 mg, 59% yield) after purification by flash column chromatography (4 g SiO_2, hexane/EA = 20:1).

^{1}**H NMR** (300 MHz, $CDCl_3$): δ 7.91 – 7.77 (m, 2H), 7.77 – 7.65 (m, 2H), 3.70 (t, J = 7.2 Hz, 2H), 2.38 – 2.17 (m, 2H), 1.99 – 1.77 (m, 4H), 1.77 – 1.58 (m, 4H), 1.50 – 1.28 (m, 4H).

^{13}**C NMR** (101 MHz, $CDCl_3$): δ 168.6, 134.0, 132.3, 129.2 (q, J = 278.0 Hz), 123.3, 44.7 (q, J = 26.2 Hz), 38.1, 34.5 (d, J = 2.0 Hz), 28.6, 27.7, 25.7 (t, J = 3.2 Hz), 23.8, 14.9.

^{19}**F NMR** (282 MHz, $CDCl_3$): δ -77.91

HRMS (ESI) m/z Calculated for $C_{18}H_{20}O_2NF_3Na$ $[M+Na]^+$: 362.1338; found: 362.13383.

■ **2-(1-(Trifluoromethyl)cyclobutyl)ethyl benzoate (38)**

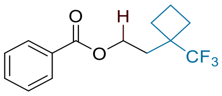

This compound was obtained according to general procedure **B** from vinyl benzoate (37.0 mg, 0.25 mmol), Isolated as transperent gel (50 mg, 74% yield) after purification by flash column chromatography (4 g SiO_2, hexane/EA = 20:1).

^{1}H NMR (300 MHz, $CDCl_3$): δ 8.09 – 7.99 (m, 2H), 7.63 – 7.50 (m, 1H), 7.51 – 7.37 (m, 2H), 4.45 (t, *J* = 7.3 Hz, 2H), 2.52 – 2.31 (m, 1H), 2.20 (t, *J* = 7.3 Hz, 2H), 2.11 – 1.88 (m, 4H).

^{13}C NMR (101 MHz, $CDCl_3$): δ 166.6, 133.2, 130.3, 130.2 (q, *J* = 280.4 Hz), 129.7, 128.6, 61.4 (d, *J* = 2.2 Hz), 43.7 (q, *J* = 27.2 Hz), 33.5 (d, *J* = 2.1 Hz), 26.2 (q, *J* = 3.2 Hz), 15.1.

^{19}F NMR (282 MHz, $CDCl_3$): δ -77.18

HRMS (ESI) m/z Calculated for $C_{14}H_{15}O_2F_3Na$ $[M+Na]^+$: 295.0916; found: 295.0916.

■ 5-(1-(Trifluoromethyl)cyclobutyl)pentyl-4-*d* benzoate (39)

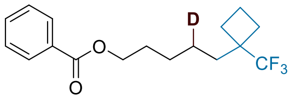

This compound was obtained according to general procedure **B** from pent-4-en-1-yl benzoate (48.0 mg, 0.25 mmol), Isolated as transparent gel (46 mg, 59% yield) after purification by flash column chromatography (4 g SiO_2, hexane/EA = 20:1).

^{1}H NMR (300 MHz, $CDCl_3$): δ 8.10 – 8.00 (m, 2H), 7.55 (m, 1H), 7.48 – 7.38 (m, 2H), 4.34 (t, *J* = 6.6 Hz, 2H), 2.37 – 2.18 (m, 2H), 2.03 – 1.74 (m, 5.34H), 1.67 (d, *J* = 7.0 Hz, 2H), 1.52 – 1.39 (m, 2H), 1.23 (m, 2H).

^{13}C NMR (101 MHz, $CDCl_3$): δ 166.8, 133.0, 130.6, 129.7, 129.5 (d, *J* = 280.1 Hz), 128.5 (t, *J* = 279.9, 279.1 Hz), 65.1, 44.8 (q, *J* = 26.3 Hz), 34.5 (q, *J* = 2.0 Hz), 32.7 – 24.6 (m), 23.7 (dd, *J* = 38.3, 18.9 Hz), 14.9.

^{19}F NMR (376 MHz, $CDCl_3$): δ -77.92

HRMS (ESI) m/z Calculated for $C_{17}H_{20}{}^{2}HO_2F_3Na$ $[M+Na]^+$: 338.1448; found: 338.1447.

■ 6,6,6-Trifluoro-3-methylhexyl benzoate (40)

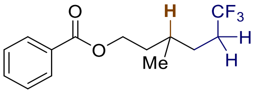

The titled compound was obtained according to general procedure **B** from 3-methylbut-3-en-1-yl benzoate (39.0 mg, 0.20 mmol). Purification was accomplished via flash column chromatography with appropriate eluents (EtOAc : hexane = 1 : 100) to give the product as a colorless gel (27 mg) in 49% yield.

^{1}H NMR (600 MHz, $CDCl_3$): δ 8.03 (d, *J* = 8.0 Hz, 2H), 7.56 (t, *J* = 8.0 Hz, 1H), 7.44 (t, *J* = 8.0 Hz, 2H), 4.41–4.34 (m, 2H), 2.18–2.05 (m, 2H), 1.86–1.61 (m, 4H), 1.50–1.44 (m, 1H), 1.00 (d, *J* = 6.0 Hz, 3H).

^{13}C NMR (150 MHz, $CDCl_3$): δ 166.6, 132.9, 130.3, 129.5, 128.3, 127.3 (q, $^1J_{C\text{–}F}$= 273 Hz), 62.9, 35.2, 31.4 (q, $^2J_{C\text{–}F}$= 28.3 Hz), 29.3, 28.6 (d, $^3J_{C\text{–}F}$= 2.9 Hz), 19.0.

^{19}F NMR (564 MHz, $CDCl_3$): δ -66.43.

HRMS (ESI) m/z Calculated for $C_{14}H_{17}F_3O_2$ $[M+H]^+$: 275.1253; found: 275.1249.

■ 5-Cyano-3-methylpentyl benzoate (41)

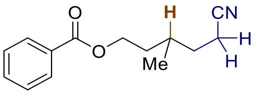

The titled compound was obtained according to general procedure **B** from 3-methylbut-3-en-1-yl benzoate (39.0 mg, 0.20 mmol). Purification was accomplished via flash column chromatography with appropriate eluents (EtOAc : hexane = 1: 20) to give the product as a colorless gel (27 mg) in 58% yield.

^{1}H NMR (600 MHz, $CDCl_3$): δ 8.03 (d, *J* = 7.9 Hz, 2H), 7.55 (t, *J* = 7.4 Hz, 1H), 7.44 (t, *J* = 7.6 Hz, 2H), 4.41–4.33 (m, 2H), 2.44–2.34 (m, 2H), 1.87–1.55 (m, 5H), 1.02 (d, *J* = 6.4 Hz, 3H).

^{13}C NMR (150 MHz, $CDCl_3$): δ 166.5, 132.9, 130.1, 129.5, 128.3, 119.6, 62.6, 34.8, 32.1, 29.3, 18.6, 14.9.

HRMS (ESI) m/z Calculated for $C_{14}H_{17}NO_2$ $[M+Na]^+$: 254.1151; found: 254.1147.

■ 6,6,6-Trifluoro-3,5-dimethylhexyl benzoate (42)

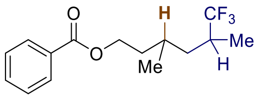

The titled compound was obtained according to general procedure **B** from appropriate olefin (39.0 mg, 0.20 mmol). Purification was accomplished via flash column chromatography with appropriate eluents (EtOAc : hexane = 1 : 100) to give the product as a colorless gel (32 mg) in 55% yield.

^{1}H NMR (600 MHz, $CDCl_3$): δ 8.04–8.02 (m, 2H), 7.56 (d, *J* = 7.4 Hz, 1H), 7.44 (d, *J* = 7.4 Hz, 2H), 4.41–4.43 (m, 2H), 2.30–2.22 (m, 1H), 1.88–1.66 (m, 3H), 1.54–1.48 (m, 1H), 1.45–1.40 (m, 1H), 1.26–1.21 (m, 1H) (diastereomer of **42**), 1.11 (d, *J* = 7.0 Hz, 3H), 1.08 (d, *J* = 6.9 Hz, 3H) (diastereomer of **42**), 1.03 (d, *J* = 6.5 Hz, 3H), 0.97 (d, *J* = 6.5 Hz, 3H) (diastereomer of **42**).

^{13}C NMR (150 MHz, $CDCl_3$): δ 166.6, 132.9, 130.33, 129.5, 128.4, 128.56 (q, $^{1}J_{C–F}$= 277.5 Hz), 62.97, 36.2, 35.58 (q, $^{2}J_{C–F}$= 28 Hz), 34.5, 26.8, 18.6, 12.38(q, $^{3}J_{C–F}$= 3.0 Hz),

(diastereomer of **42**) **^{13}C NMR** (150 MHz, $CDCl_3$): δ 166.6, 132.9, 130.31, 129.5, 128.4, 128.56 (q, $^{1}J_{C–F}$= 277.5 Hz), 62.95, 37.1, 36.3, 35.58 (q, $^{2}J_{C–F}$= 28 Hz), 27.3, 20.1, 13.33(q, $^{3}J_{C–F}$= 3.0 Hz).

^{19}F NMR (564 MHz, $CDCl_3$): δ -73.20 (d, *J* = 8.0 Hz), -73.60 (d, *J* = 8.0 Hz) (diastereomer of **42**).

HRMS (ESI) m/z Calculated for $C_{15}H_{19}F_3O_2$ $[M+Na]^+$: 311.1229; found: 311.1224.

■ 6,6,6-Trifluoro-3,5,5-trimethylhexyl benzoate (43)

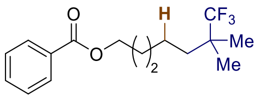

The titled compound was obtained according to general procedure **B** from pent-4-en-1-yl benzoate (38.0 mg, 0.20 mmol). Purification was accomplished via flash column chromatography with appropriate eluents (EtOAc : hexane = 1 : 20) to give the product as a colorless gel (24 mg) in 30% yield.

^{1}H NMR (600 MHz, $CDCl_3$): δ 8.04 (d, *J* = 7.9 Hz, 2H), 7.56 (t, *J* = 7.4 Hz, 1H), 7.44 (t, *J* = 7.6 Hz, 2H), 4.33 (t, *J* = 6.5 Hz, 2H), 1.80 (quint, *J* = 7.3 Hz, 2H), 1.50–1.38 (m, 6H), 1.08 (s, 6H).

^{13}C NMR (150 MHz, $CDCl_3$): δ 166.7, 132.9, 130.4, 129.7 (q, $^{1}J_{C–F}$= 280 Hz), 129.5, 128.3, 64.9, 40.1 (q, $^{2}J_{C–F}$= 24 Hz), 35.7, 28.6, 26.8, 23.4, 20.53(d, $^{3}J_{C–F}$= 3.0 Hz).

^{19}F NMR (564 MHz, $CDCl_3$): δ -78.30.

HRMS (ESI) m/z Calculated for $C_{16}H_{21}F_3O_2$ $[M+H]^+$: 303.1566; found: 303.1561.

■ 3-Methyl-1-tosyl-4-((1-(trifluoromethyl)cyclopropyl)methyl)pyrrolidine (46)

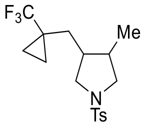

The titled compound was obtained according to general procedure **B** from *N*,*N*-diallyl-4-methylbenzenesulfonamide (50.0 mg, 0.20 mmol). Purification was accomplished via flash column chromatography with appropriate eluents (EtOAc : hexane = 1 : 20) to give the product as a colorless gel in 36% yield.

^{1}H NMR (600 MHz, $CDCl_3$) (major diastereomer): δ 7.71 (d, *J* = 8.0 Hz, 2H), 7.32 (d, *J* = 8.0 Hz, 2H), 3.42 (dd, *J* = 9.7, 7.1 Hz, 1H), 3.29 (dd, *J* = 9.8, 6.5 Hz, 1H), 3.10 (t, *J* = 8.6 Hz, 1H), 3.03 (dd, *J* = 9.8, 3.9 Hz, 1H), 2.43 (s, 3H), 2.27–2.13 (m, 2H), 1.98 (dd, *J* = 14.9, 4.3 Hz, 1H), 1.00–0.93 (m, 3H), 0.73 (d, *J* = 7.0 Hz, 3H), 0.59–0.44 (m, 2H).

^{13}C NMR (150 MHz, $CDCl_3$) (major diastereomer): δ 143.3, 134.1, 129.6, 127.3, 127.2 (q, $^{1}J_{C-F}$= 273 Hz), 54.2, 50.8, 39.6, 35.8, 30.7, 21.5, 20.9 (q, $^{2}J_{C-F}$ = 31 Hz), 12.3, 9.22 (d, $^{3}J_{C-F}$= 3.0 Hz).

^{19}F NMR (564 MHz, $CDCl_3$) (major diastereomer): δ -68.92.

^{1}H NMR (600 MHz, $CDCl_3$) (minor diastereomer): δ 7.71 (d, *J* = 8.0 Hz, 2H), 7.32 (d, *J* = 8.0 Hz, 2H), 3.62 (dd, *J* = 10.0, 7.3 Hz, 1H), 3.49 (dd, *J* = 9.7, 7.6 Hz, 1H), 2.94 (t, *J* = 9.1 Hz, 1H), 2.71 (t, *J* = 9.2 Hz, 1H), 2.43 (s, 3H), 2.04 (dd, *J* = 14.9, 3.7 Hz, 1H), 1.75–1.64 (m, 2H), 1.00–0.93 (m, 3H), 0.90 (d, *J* = 6.5 Hz, 3H), 0.59–0.44 (m, 2H).

^{13}C NMR (150 MHz, $CDCl_3$) (minor diastereomer): δ 143.4, 133.8, 129.6, 127.4, 127.1 (q, $^{1}J_{C-F}$= 273 Hz), 54.1, 43.8, 39.3, 34.8, 29.7, 21.5, 21.1 (q, $^{2}J_{C-F}$ = 33 Hz), 16.0, 9.04 (d, $^{3}J_{C-F}$= 3.0 Hz).

^{19}F NMR (564 MHz, $CDCl_3$) (minor diastereomer): δ -69.05.

HRMS (ESI) m/z Calculated for $C_{17}H_{22}F_3NO_2S$ $[M+Na]^+$: 384.1216; found: 384.1214.

5-(1-(Trifluoromethyl)cyclopropyl)pentyl-4-*d* benzoate (47)

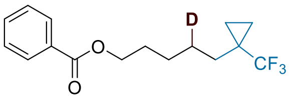

This compound was obtained according to general procedure **B** from pent-4-en-1-yl benzoate (48.0 mg, 0.25 mmol), Isolated as transparent gel (47 mg, 63% yield) after purification by flash column chromatography (4 g SiO_2, hexane/EA = 20:1).

^{1}H NMR (600 MHz, CD_3CN): δ 8.03 – 7.98 (m, 2H), 7.61 (t, *J* = 7.3 Hz, 1H), 7.52 – 7.46 (m, 2H), 4.28 (t, *J* = 6.3 Hz, 2H), 1.79 – 1.71 (m, 2H), 1.62 – 1.56 (m, 2H), 1.51 – 1.38 (m, 3H), 0.92 – 0.89 (m, 2H), 0.66 – 0.63 (m, 2H).

^{13}C NMR (101 MHz, $CDCl_3$): δ 166.8, 130.0 – 129.2 (m), 129.5 (q, *J* = 273.8 Hz), 128.5 (dd, *J* = 275.6, 273.5 Hz), 65.1, 32.0, 28.7, 26.4, 26.2 (dd, *J* = 37.7, 18.4 Hz), 22.4 (d, *J* = 32.2 Hz), 8.9 (p, *J* = 2.6 Hz).

^{19}F NMR (376 MHz, $CDCl_3$): δ -69.67

HRMS (ESI) m/z Calculated for $C_{16}H_{18}$**^{2}H**O_2F_3Na $[M+Na]^+$: 324.1292; found: 324.1289.

8. Computational details

8.1 Computational methods

The DFT calculations have been performed with the Gaussian 9 program package.[58]

The conformational space of all molecules was initially searched using meta-dynamics simulations based on tight-binding quantum chemical calculations as implemented in the software package Conformer-Rotamer Ensemble Sampling Tool CREST.[59]

The structures located with CREST have been subjected to geometry optimization using (U)M06-2X functional[60] with Def2-SVP basis set,[61] including D3 dispersion correction[62] and polarizable continuum model (PCM)[63]with SMD parameters[64] to consider solvent effects (SMD parameters of acetonitrile are available in the used software package). The nature of all stationary points (minima and transition states) was verified through the computation of the vibrational frequencies. Single point (SP) energies from these geometries were calculated at the (U)M06-2X/Def2-TZVP level of theory, including SMD solvation model (SMD parameters of acetonitrile are available in the used software package). The thermal corrections to the Gibbs free energies were combined with the single-point energies to yield Gibbs free energies (ΔG) at 298.15 K. All energies are reported in kcal·mol^{-1} unless otherwise stated.

Visualizations of molecules were prepared with Legault's CYLview20.

8.2 Radical stabilization energy scale

Radical stabilization energy (RSE)[65, 66, 67] approaches have shown to strongly correlate with decarboxylation kinetics in a mechanistically analogous system.[53]

Using the present computational method in the gas phase, a BDE of 103.8 kcal·mol^{-1} was calculated for methane at 298K (**Table S8**), in excellent agreement with the 104.9 kcal·mol^{-1} reported from the ATcT.[68]

---

53. L. M. Denkler, M. A. Shekar, T. S. J. Ngan, L. Wylie, D. Abdullin, M. Engeser, G. Schnakenburg, T. Hett, F. H. Pilz, B. Kirchner, O. Schiemann, P. Kielb, A. Bunescu, *Angew. Chem. Int. Ed.* **2024**, *63*, e202403292; *Angew. Chem.* **2024**, *136*, e202403292.

[58] M. J. Frisch, G. W. Trucks, H. B. Schlegel, G. E. Scuseria, M. A. Robb, J. R. Cheeseman, G. Scalmani, V. Barone, B. Mennucci, G. A. Petersson, H. Nakatsuji, M. Caricato, X. Li, H. P. Hratchian, A. F. Izmaylov, J. Bloino, G. Zheng, J. L. Sonnenberg, M. Hada, M. Ehara, K. Toyota, R. Fukuda, J. Hasegawa, M. Ishida, T. Nakajima, Y. Honda, O. Kitao, H. Nakai, T. Vreven, J. J. A. Montgomery, J. E. Peralta, F. Ogliaro, M. Bearpark, J. J. Heyd, E. Brothers, K. N. Kudin, V. N. Staroverov, T. Keith, R. Kobayashi, J. Normand, K. Raghavachari, A. Rendell, J. C. Burant, S. S. Iyengar, J. Tomasi, M. Cossi, N. Rega, J. M. Millam, M. Klene, J. E. Knox, J. B. Cross, V. Bakken, C. Adamo, J. Jaramillo, R. Gomperts, R. E. Stratmann, O. Yazyev, A. J. Austin, R. Cammi, C. Pomelli, J. W. Ochterski, R. L. Martin, K. Morokuma, V. G. Zakrzewski, G. A. Voth, P. Salvador, J. J. Dannenberg, S. Dapprich, A. D. Daniels, O. Farkas, J. B. Foresman, J. V. Ortiz, J. Cioslowski, D. J. Fox, Wallingford, CT, **2013**

[59] **a.** S. Grimme, *J. Chem. Theory Comput*. **2019**, *15* (5), 2847-2862; **b.** P. Pracht, F. Bohle, S. Grimme, *Phys. Chem. Chem. Phys.* **2020**, *22* (14), 7169-7192.

[60] **a.** Y. Zhao, D. G. Truhlar, *Theor. Chem. Account*. **2008**, *120* (1-3), 215-241; **b.** P. C. St. John, Y. Guan, Y. Kim, B. D. Etz, S. Kim, R. S. Paton, *Sci. Data* **2020**, *7* (1), 244.

[61] **a.** F. Weigend, R. Ahlrichs, *Phys. Chem. Chem. Phys.* **2005**, *7* (18), 3297-3305; **b.** D. Rappoport, F. Furche, *J. Chem. Phys.* **2010**, *133* (13), 134105.

[62] S. Grimme, A. Hansen, J. G. Brandenburg, C. Bannwarth, *Chem. Rev*. **2016**, *116* (9), 5105-5154.

[63] E. Cancès, B. Mennucci, J. Tomasi, *J. Chem. Phys.* **1997**, *107* (8), 3032- 3041.

[64] A. V. Marenich, C. J. Cramer, D. G. Truhlar, *J. Phys. Chem. B* **2009**, *113* (18), 6378-6396.

[65] J. Xu, Y. Liu, Q. Wang, X. Tao, S. Ni, W. Zhang, L. Yu, Y. Pan, Y. Wang, *Nat. Commun.* **2024**, *15* (1), 6116. https://doi.org/10.1038/s41467-024-50596-3.

[66] M. A. J. Dubois, J. J. Rojas, A. J. Sterling, H. C. Broderick, M. A. Smith, A. J. P. White, P. W. Miller, C. Choi, J. J. Mousseau, F. Duarte, J. A. Bull, *J. Org. Chem.* **2023**, *88* (10), 6476–6488.

[67] H. Zipse, *Radicals in Synthesis I* (**2006**) 163–189.

[68] B. Ruscic, *J. Phys. Chem. A* **2015**, *119*, 7810–7837.

Table S8. Computed energies at the (U)M062X-D3/def2-TZVP//(U)M062X-D3/def2-SVP. (Energies in Hartree unless otherwise stated).

Radical	Thermal correction		SP energy	*ΔG*	*ΔH*	BDE
	to *H*	to *G*				*ΔH in kcal·mol⁻¹*
H	0.00236	-0.010654	-0.498138512	-0.508792512	-0.495778512	--
$^{\bullet}CH_3$	0.033619	0.009561	-39.82381786	-39.81425686	-39.79019886	
CH_4	0.048635	0.02514	-40.50009821	-40.47495821	-40.45146321	103.8

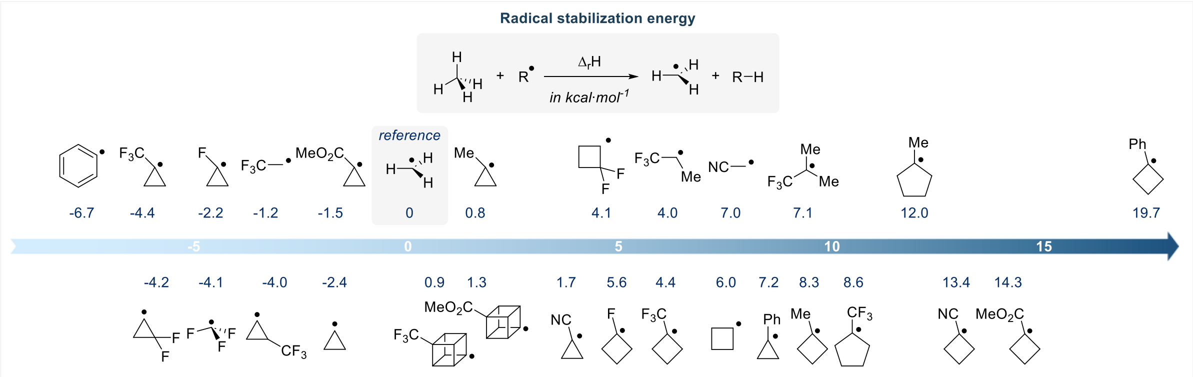

Figure S15. Radical stabilization energy scale computed at the (U)M062X-D3/def2-TZVP,SMD(MeCN)//(U)M062X-D3/def2-SVP,SMD(MeCN) level of theory.

Table S9. Computed energies at the (U)M062X-D3/def2-TZVP,SMD(MeCN)//(U)M062X-D3/def2-SVP,SMD(MeCN). (Energies in Hartree unless otherwise stated)

Radical	Thermal correction		SP energy	*ΔG*	*ΔH*	RSE *ΔΔH (BDE)*
	to *H*	to *G*				*in kcal·mol⁻¹*
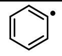	0.093434	0.06013	-231.5470766	-231.4869466	-231.4536426	-6.7 (111.6)
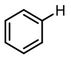	0.106372	0.07359	-232.2331833	-232.1595933	-232.1268113	
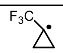	0.081278	0.04189	-454.3012045	-454.2593145	-454.2199265	-4.5 (109.4)
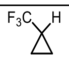	0.094791	0.057145	-454.9844	-454.927255	-454.889609	
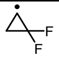	0.057134	0.023661	-315.7080774	-315.6844164	-315.6509434	-4.2 (109.1)
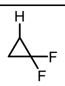	0.071162	0.038422	-316.3913239	-316.3529019	-316.3201619	
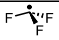	0.016942	-0.014059	-337.5972048	-337.6112638	-337.58	-4.1

Radical	Thermal correction		SP energy	*ΔG*	*ΔH*	RSE *ΔΔH (BDE)* in *kcal·mol*$^{-1}$
	to *H*	to *G*				
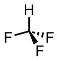	0.030494	0.000063	-338.2798879	-338.2798249	-338.2493939	(109.0)
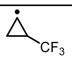	0.057134	0.023661	-315.7080774	-315.6844164	-315.651	-4.0 (108.9)
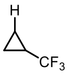	0.094791	0.057145	-454.9844	-454.927255	-454.889609	
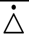	0.071192	0.041959	-117.202297	-117.160338	-117.131105	-2.4 (107.3)
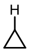	0.085372	0.056756	-117.8828891	-117.8261331	-117.7975171	
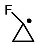	0.065294	0.03378	-216.4543505	-216.4205705	-216.3890565	-2.2 (107.1)
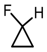	0.078437	0.047558	-217.1334846	-217.0859266	-217.0550476	
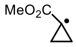	0.119839	0.07787	-345.1003823	-345.0225123	-344.9805433	-1.5 (106.4)
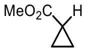	0.133818	0.093912	-345.7793169	-345.6854049	-345.6454989	
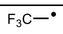	0.04417	0.009612	-376.9217657	-376.9121537	-376.8775957	-1.2 (106.1)
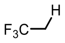	0.058541	0.025184	-377.6005293	-377.5753453	-377.5419883	
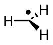	0.033478	0.00949	-39.82217999	-39.81268999	-39.78870199	0 (reference) (BDE = 104.9)
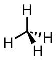	0.048326	0.024824	-40.49958174	-40.47475774	-40.45125574	
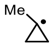	0.100972	0.067387	-156.5176091	-156.4502221	-156.4166371	0.8 (104.1)
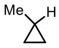	0.11475	0.082607	-157.1926276	-157.1100206	-157.0778776	
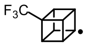	0.136488	0.092854	-645.876829	-645.783975	-645.740341	0.9 (104.0)
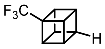	0.149348	0.106284	-646.5507413	-646.4444573	-646.4013933	
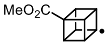	0.175482	0.12969	-536.668726	-536.539036	-536.493244	1.3 (103.6)
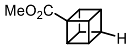	0.188191	0.142952	-537.3419009	-537.1989489	-537.1537099	
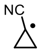	0.072672	0.038031	-209.4678238	-209.4297928	-209.3951518	1.7 (103.2)
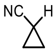	0.086298	0.053118	-210.1413015	-210.0881835	-210.0550035	
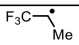	0.074104	0.034386	-416.2378679	-416.2034819	-416.1637639	4.0

Radical	Thermal correction		SP energy	*ΔG*	*ΔH*	RSE *ΔΔH (BDE)* in *kcal·mol*$^{-1}$
	to *H*	to *G*				
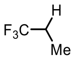	0.088526	0.05176	-416.9084764	-416.8567164	-416.8199504	(100.9)
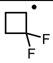	0.087563	0.050626	-355.0462304	-354.9956044	-354.9586674	4.1 (100.8)
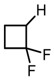	0.101728	0.066499	-355.716436	-355.649937	-355.614708	
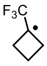	0.111128	0.06773	-493.6218291	-493.5540991	-493.5107011	4.4 (100.5)
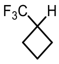	0.125091	0.084584	-494.2912922	-494.2067082	-494.1662012	
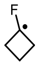	0.09565	0.061365	-255.7814168	-255.7200518	-255.6857668	5.6 (99.3)
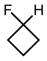	0.109172	0.075954	-256.4485527	-256.3725987	-256.3393807	
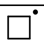	0.101075	0.067115	-156.5193606	-156.4522456	-156.4182856	6.0 (98.9)
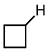	0.11567	0.084467	-157.1869674	-157.1025004	-157.0712974	
	0.035902	0.007067	-132.0918657	-132.0847987	-132.0559637	7.0 (97.9)
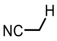	0.04998	0.021464	-132.7573811	-132.7359171	-132.7074011	
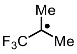	0.103935	0.06097	-455.552414	-455.491444	-455.448479	7.1 (97.8)
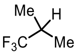	0.117878	0.078105	-456.21761	-456.139505	-456.099732	
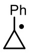	0.157595	0.115037	-348.267038	-348.152001	-348.109443	7.2 (97.7)
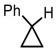	0.171773	0.131323	-348.9323592	-348.8010362	-348.7605862	
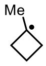	0.130763	0.093651	-195.8344535	-195.7408025	-195.7036905	8.3 (96.6)
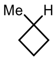	0.144874	0.110348	-196.4978466	-196.3874986	-196.3529726	
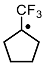	0.141747	0.096664	-532.9674987	-532.8708347	-532.8257517	8.6 (96.3)
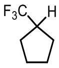	0.155866	0.113682	-533.6304231	-533.5167411	-533.4745571	

Radical	Thermal correction		SP energy	*ΔG*	*ΔH*	RSE *ΔΔH (BDE)* in kcal·mol^{-1}
	to *H*	to *G*				
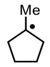	0.161365	0.122511	-235.1774644	-235.0549534	-235.0160994	12.0 (92.9)
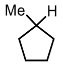	0.175762	0.137092	-235.8353034	-235.6982114	-235.6595414	
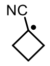	0.103024	0.065432	-248.7923043	-248.7268723	-248.6892803	13.4 (91.5)
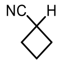	0.116772	0.0811	-249.4472233	-249.3661233	-249.3304513	
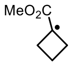	0.150182	0.106054	-384.427455	-384.321401	-384.277273	14.3 (90.6)
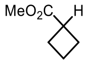	0.163967	0.120216	-385.0810343	-384.9608183	-384.9170673	
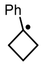	0.188261	0.143803	-387.5907237	-387.4469207	-387.4024627	19.7 (85.2)
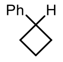	0.202041	0.158491	-388.2357002	-388.0772092	-388.0336592	

8.3 Philicity indices and other parameters

The calculation of the global electrophilicity and nucleophilicity indices was performed following a reported method by De Proft *et al.* (*i.e.* in gas phase using the unrestricted (U)B3LYP functional,[69,70,71] with 6-311+G(d,p) basis set,[72],[73] including D3(BJ) dispersion correction,[74] through the calculation of vertical ionization energy and vertical electron affinity).[75]

Global electrophilicity indices ω were calculated as defined by Parr *et al.*[76] using equation 1,

$$\omega = \frac{\mu^2}{2\eta} \quad (1)$$

where μ, the electronic chemical potential (also defined as $-\chi$, the negative of the electronegativity), and η, the chemical hardness, determined using the finite differences method[77,78] based on vertical ionization energy (IE) and vertical electron affinity (EA), following equation 2 and 3.

$$\mu = -\chi \approx -\frac{IE + EA}{2} \quad (2)$$

[69] C. Lee, W. Yang, R. G. Parr, *Phys. Rev. B* **1988**, *37*, 785–789.
[70] A. D. Becke, *J. Chem. Phys.* **1993**, *98*, 5648–5652.
[71] S. Grimme, A. Hansen, J. G. Brandenburg, C. Bannwarth, *Chem. Rev.* **2016**, *116*, 5105–5154.
[72] R. Krishnan, J. S. Binkley, R. Seeger, J. A. Pople, *J. Chem. Phys.* **1980**, *72*, 650–654.
[73] A. D. McLean, G. S. Chandler, *J. Chem. Phys.* **1980**, *72*, 5639–5648.
[74] S. Grimme, R. Huenerbein, S. Ehrlich, *ChemPhysChem* **2011**, *12*, 1258–1261.
[75] F. De Vleeschouwer, V. Van Speybroeck, M. Waroquier, P. Geerlings, F. De Proft, *Org. Lett.* **2007**, *9*, 2721–2724.
[76] R. G. Parr, L. v. Szentpály, S. Liu, *J. Am. Chem. Soc.* **1999**, *121*, 1922–1924.
[77] R. G. Parr, R. G. Pearson, *J. Am. Chem. Soc.* **1983**, *105*, 7512–7516.
[78] F. De Proft, P. Geerlings, *Chem. Rev.* **2001**, *101*, 1451–1464.

$$\eta \approx IE - EA \tag{3}$$

A nucleophilicity scale, referenced to fluorine radical ($\omega^{-}(F^{\bullet}) = 0$), has also been defined by Jaramillo *et al.*[79] and was utilized for the calculation of the nucleophilicity indices of radicals using equation 4.

$$\omega^{-} = \frac{1}{2}\frac{(\mu_{X\bullet} - \mu_{F\bullet})^2}{(\eta_{X\bullet} + \eta_{F\bullet})^2}\eta_{X\bullet} \tag{4}$$

Table S10. Calculated parameters including, *IE*: vertical ionization energy; *EA*: vertical electron affinity; η: Chemical hardness; μ: Chemical potential (-Electronegativity); ω: Global Electrophilicity index; ω-: Nucleophilicity index. (Negative Vertical Electron Affinity refers to cases where the energy of the anion at the radical's geometry is higher than the energy of the ground state radical plus an electron).

	**Parameters in gas phase (U)B3LYP-D3(BJ)/6-311+G(d,p)**					
**Radical**	***IE* (eV)**	***EA* (eV)**	**η (eV)**	**μ (eV)**	**ω (eV)**	**ω- (eV)**
$F^{\bullet}$	17.77	3.49	14.28	-10.63	3.954	0.000
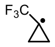	10.76	-1.09	11.85	-4.84	0.99	0.29
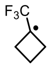	8.71	0.61	8.09	-4.66	1.34	0.29
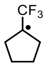	8.42	0.48	7.94	-4.45	1.25	0.31
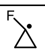	9.00	0.19	8.81	-4.59	1.20	0.30
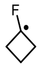	8.08	-0.38	8.45	-3.85	0.88	0.38
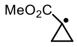	8.70	1.21	7.50	-4.95	1.64	0.25
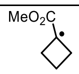	8.19	0.99	7.21	-4.59	1.46	0.28
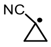	9.10	1.31	7.79	-5.21	1.74	0.24
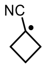	8.55	1.06	7.49	-4.80	1.54	0.27
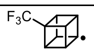	7.89	0.58	7.30	-4.23	1.23	0.32
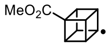	7.50	0.39	7.11	-3.94	1.09	0.35
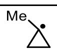	7.95	-0.17	8.12	-3.89	0.93	0.37

[79] P. Jaramillo, P. Pérez, R. Contreras, W. Tiznado, P. Fuentealba, *J. Phys. Chem. A* **2006**, *110*, 8181–8187.

	Parameters in gas phase (U)B3LYP-D3(BJ)/6-311+G(d,p)					
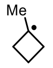	7.08	-0.63	7.70	-3.23	0.68	0.44
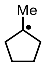	6.79	-0.77	7.56	-3.01	0.60	0.46
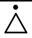	8.73	-0.13	8.86	-4.30	1.04	0.33
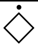	7.69	-0.62	8.31	-3.53	0.75	0.41
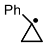	6.64	0.00	6.64	-3.32	0.83	0.41
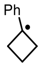	6.45	0.61	5.84	-3.53	1.07	0.36
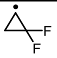	9.90	1.00	8.91	-5.45	1.67	0.22
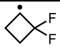	9.29	0.68	8.60	-4.99	1.45	0.26
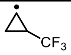	9.47	0.63	8.84	-5.05	1.45	0.26
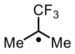	8.75	0.48	8.27	-4.62	1.29	0.29

8.4 Redox potentials

Redox potential were calculated based on previous examples in the literature by Guo, Liu et al.[80] and Nicewicz et al.,[81] using the unrestricted (U)B3LYP functional,[69,70,71] with 6-311+G(d,p) basis set,[72,73] including D3(BJ) dispersion correction[74] and CPCM solvation model [82] This level of theory demonstrated excellent ability to predict redox potentials both for open-shell and close-shell organic molecules. Hence, the geometry of the radicals and their reduced form were optimized at the (U)B3LYPD3(BJ)/6-311+G(d,p),CPCM(MeCN) level of theory. The corresponding "sum of electronic and thermal free energies" was then utilized in the following equation, ultimately leading to the reduction potential values in acetonitrile referenced to the saturated calomel electrode (SCE).

$$E_{(calc),red}\,(SCE) = -\frac{(G_{298}[reduced] - G_{298}[radical])}{nF} - E_{1/2}^{SHE} + E_{1/2}^{SCE}$$

Where G_{298}[radical] and G_{298}[reduced] are the "sum of electronic and thermal energies" calculated for the radical and its reduced form in acetonitrile respectively, n_e is the number of transferred electrons (i.e. n = 1 in all the cases considered here), F is the Faraday constant (F = 23.061 kcal·mol^{-1}V^{-1}), $E_{1/2}^{SHE}$ is the absolute value for the standard hydrogen electrode (SHE) and is equal to 4.281 V and $E_{1/2}^{SCE}$ is the potential of the saturated calomel electrode (SCE) versus SHE in acetonitrile (it is equal to -0.141 V).[81]

[80] Y. Fu, L. Liu, H.-Z. Yu, Y.-M. Wang, Q.-X. Guo, *J. Am. Chem. Soc.* **2005**, *127*, 7227–7234.
[81] H. G. Roth, N. A. Romero, D. A. Nicewicz, *Synlett* **2016**, *27*, 714–723.
[82] V. Barone, M. Cossi, *J. Phys. Chem. A* **1998**, *102*, 1995–2001.

Table S11. Calculated redox potentials.

	Redox potential in solution (MeCN) (U)B3LYP-D3(BJ)/6-311+G(d,p),CPCM(MeCN)	
**Radical**	***ΔG° in kcal·mol^{-1}***	***E(calc),ox (V vs SCE)***
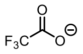	-156.1	2.35
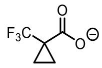	-139.7	1.63
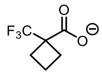	-139.5	1.63
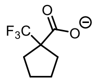	-137.9	1.56
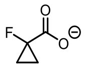	-140.1	1.65
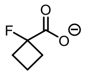	-139.3	1.62
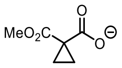	-135.2	1.44
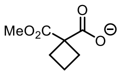	-136.8	1.51
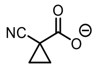	-142.3	1.75
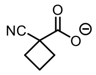	-141.7	1.72
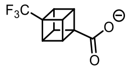	-133.6	1.37
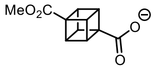	-132.8	1.34
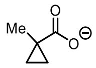	-130.6	1.24
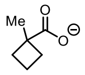	-130.5	1.24
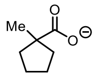	-129.1	1.17

	Redox potential in solution (MeCN) (U)B3LYP-D3(BJ)/6-311+G(d,p),CPCM(MeCN)	
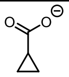	-132.2	1.31
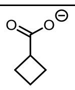	-131.5	1.28
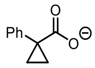	-130.0	1.22
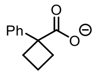	-130.5	1.24
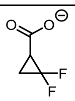	-139.0	1.60
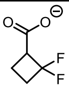	-138.0	1.56
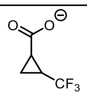	-137.4	1.54
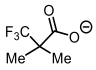	-138.4	1.58

8.5 Computed reaction mechanism

Table S12. Computed energies at the (U)M062X-D3/def2-TZVP,SMD(MeCN)//(U)M062X-D3/def2-SVP,SMD(MeCN). (Energies in Hartree)

Computed reaction mechanism						
**Compound**	**Imaginary frequency**	**Thermal correction**		**SP energy**	***ΔG***	***ΔH***
		**to *H***	**to *G***			
**III_{cp}**	--	0.099241	0.052912	-642.8814676	-642.8285556	-642.7822266
**$TS1_{cp}$**	-281.3533	0.096754	0.051662	-642.8789453	-642.8272833	-642.7821913
**IV_{cp}-CO2-prodC**	--	0.098423	0.047273	-642.9027019	-642.8554289	-642.8042789
**IV_{cp}**	--	0.081269	0.041896	-454.3012729	-454.2593769	-454.2200039
**IV_{cp}-alkene-reactC**	--	0.332935	0.252518	-1070.441323	-1070.188805	-1070.108388
**$TS2_{cp}$**	-334.0878	0.331397	0.255002	-1070.436758	-1070.181756	-1070.105361

Computed reaction mechanism						
**Compound**	**Imaginary frequency**	**Thermal correction**		**SP energy**	***ΔG***	***ΔH***
		**to *H***	**to *G***			
**V$_{cp}$**	--	0.334747	0.258415	-1070.492394	-1070.233979	-1070.157647
**V$_{cp}$-thiol-reactC**	--	0.709519	0.587367	-2054.705385	-2054.118018	-2053.995866
**TS3$_{cp}$**	-706.8277	0.705816	0.586075	-2054.699451	-2054.113376	-2053.993635
**2-thiyl-prodC**	--	0.713959	0.594226	-2054.739677	-2054.145451	-2054.025718
**2**	--	0.348696	0.275209	-1071.154877	-1070.879668	-1070.806181
**III$_{cb}$**	--	0.129001	0.080959	-682.1911154	-682.1101564	-682.0621144
**TS1$_{cb}$**	-531.1121	0.126935	0.079702	-682.1901584	-682.1104564	-682.0632234
**IV$_{cb}$-CO2-prodC**	--	0.128217	0.075671	-682.2239328	-682.1482618	-682.0957158
**IV$_{cb}$**	--	0.111128	0.067737	-493.6218294	-493.5540924	-493.5107014
**IV$_{cb}$-alkene-reactC**	--	0.36267	0.278392	-1109.762785	-1109.484393	-1109.400115
**TS2$_{cb}$**	-446.5221	0.361784	0.284288	-1109.753101	-1109.468813	-1109.391317
**V$_{cb}$**	--	0.364691	0.290544	-1109.802698	-1109.512154	-1109.438007
**V$_{cb}$-thiol-reactC**	--	0.738464	0.619351	-2094.012251	-2093.3929	-2093.273787
**TS3$_{cb}$**	-741.589	0.736518	0.619496	-2094.007777	-2093.388281	-2093.271259
**34-thiyl-prodC**	--	0.744571	0.623896	-2094.046565	-2093.422669	-2093.301994
**34**	--	0.379249	0.304939	-1110.46091	-1110.155971	-1110.081661
**CO_2**	--	0.014423	-0.004968	-188.5971932	-188.6021612	-188.5827702
**H_2O**	--	0.02527	0.003175	-76.4343883	-76.4312133	-76.4091183
**H_2CO_3**	--	0.044741	0.014224	-265.0370477	-265.0228237	-264.9923067
**modelalkene**	--	0.249557	0.192203	-616.1346307	-615.9424277	-615.8850737
**TRIPthiol**	--	0.373195	0.307773	-984.2026926	-983.8949196	-983.8294976
**TRIPthiyl-rad**	--	0.363392	0.298147	-983.5737425	-983.2755955	-983.2103505

8.6 Computed structures

8.6.1 RSE scale

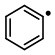

C -3.551897000 -2.032018000 -0.007497000
C -2.155328000 -2.046150000 0.034530000
C -1.438600000 -0.841239000 0.057770000
C -2.180928000 0.319815000 0.037463000
C -3.556634000 0.395095000 -0.003581000
C -4.251445000 -0.822654000 -0.026033000
H -4.102113000 -2.974400000 -0.025668000
H -1.616092000 -2.995894000 0.049092000
H -0.346571000 -0.837543000 0.090298000
H -4.091833000 1.347443000 -0.017946000

H	-5.343205000	-0.821519000	-0.058259000

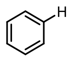

C	-1.191697000	0.797090000	0.500189000
C	-1.190972000	-0.190033000	-0.486642000
C	0.000646000	-0.830903000	-0.830773000
C	1.191498000	-0.485747000	-0.189028000
C	1.190686000	0.501237000	0.797942000
C	-0.000888000	1.142584000	1.142410000
H	-2.122615000	1.299485000	0.769948000
H	-2.120866000	-0.461365000	-0.990336000
H	0.001261000	-1.603126000	-1.602877000
H	2.121994000	-0.988757000	-0.459562000
H	2.121005000	0.771794000	1.301018000
H	-0.001505000	1.915140000	1.914846000

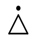

C	0.688493000	-0.408412000	-0.000855000
C	2.210576000	-0.408059000	-0.001397000
C	1.449315000	0.830486000	0.185472000
H	0.195032000	-0.584708000	-0.962904000
H	0.162900000	-0.837639000	0.858643000
H	2.703438000	-0.584124000	-0.963796000
H	2.736986000	-0.837037000	0.857725000
H	1.449442000	1.560246000	0.994880000

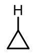

C	0.699352000	-0.367286000	0.008138000
C	2.199708000	-0.366936000	0.007602000
C	1.449309000	0.914386000	0.221392000
H	0.190025000	-0.507264000	-0.947422000
H	0.190245000	-0.809880000	0.866608000
H	2.708424000	-0.506688000	-0.948317000
H	2.709630000	-0.809291000	0.865711000
H	1.449575000	1.344041000	1.225041000
H	1.448843000	1.646035000	-0.588893000

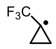

C	0.688930000	-0.397243000	-0.003705000
C	2.211205000	-0.397426000	-0.003566000
C	1.450205000	0.845679000	0.123359000
H	0.196498000	-0.601850000	-0.958562000
H	0.160751000	-0.763558000	0.882309000
H	2.703763000	-0.602157000	-0.958332000
H	2.739129000	-0.763872000	0.882546000
C	1.450220000	1.795959000	1.251775000
F	0.375146000	2.591226000	1.247878000
F	2.525391000	2.591094000	1.247959000
F	1.450135000	1.151199000	2.431887000

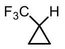

C	0.669896000	-0.415721000	0.124901000
C	2.164635000	-0.394690000	0.047450000
C	1.397305000	0.892619000	0.047560000
H	0.217621000	-0.681014000	1.082634000
H	2.642243000	-0.678652000	-0.890729000
H	2.720763000	-0.645968000	0.952772000
C	1.444572000	1.752984000	1.261504000
F	0.359646000	2.532158000	1.368581000
F	2.502316000	2.576564000	1.256099000
F	1.518923000	1.036872000	2.391873000
H	1.341686000	1.477478000	-0.872714000
H	0.105192000	-0.714294000	-0.758862000

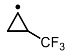

C	0.741216000	-0.376278000	0.101293000
C	2.201925000	-0.383651000	0.039302000
C	1.424520000	0.921426000	0.046199000
H	0.041791000	-0.781400000	0.831630000
H	2.698828000	-0.634808000	-0.902807000
H	2.759069000	-0.659054000	0.941464000
C	1.458875000	1.779374000	1.268376000
F	0.370900000	2.553324000	1.365897000
F	2.514197000	2.603724000	1.277677000
F	1.521749000	1.051667000	2.391073000
H	1.400534000	1.513434000	-0.875061000

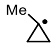

C	0.686705000	-0.401244000	-0.002804000
C	2.212345000	-0.400921000	-0.003297000
C	1.449320000	0.839601000	0.173648000
H	0.189693000	-0.596405000	-0.959118000
H	0.169080000	-0.820192000	0.868333000
H	2.708829000	-0.595874000	-0.959927000
H	2.730710000	-0.819638000	0.867512000
C	1.449478000	1.835734000	1.271482000
H	0.556252000	2.477120000	1.241641000
H	2.342251000	2.477716000	1.240837000
H	1.450115000	1.317263000	2.249379000

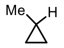

C	0.694670000	-0.371461000	0.013212000
C	2.198314000	-0.367115000	0.006708000
C	1.442796000	0.929337000	0.031640000
H	0.177787000	-0.661884000	-0.903589000
H	0.192786000	-0.670924000	0.936935000
H	2.708765000	-0.654524000	-0.914623000
H	2.709976000	-0.663657000	0.925994000
C	1.446055000	1.773291000	1.282969000
H	0.556632000	2.420005000	1.334520000
H	2.334783000	2.421406000	1.328830000
H	1.449422000	1.134532000	2.179449000
H	1.436958000	1.492009000	-0.906383000

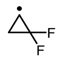

C	0.689139000	-0.421802000	0.003301000
C	2.176377000	-0.400099000	-0.001275000
C	1.459691000	0.836066000	0.203405000
H	0.205425000	-0.585580000	-0.968486000
H	0.174398000	-0.863337000	0.864960000
H	1.457816000	1.603624000	0.976131000
F	2.842611000	-0.634373000	-1.148033000
F	2.861329000	-0.969786000	1.007560000

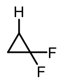

C	0.691661000	-0.378577000	0.008409000
C	2.159592000	-0.344062000	0.013962000
C	1.464955000	0.930768000	0.233864000
H	0.201579000	-0.509974000	-0.958034000
H	0.200401000	-0.826906000	0.873804000
H	1.478151000	1.335130000	1.247638000
F	2.835816000	-0.548101000	-1.131026000
F	2.836890000	-0.917303000	1.025239000
H	1.476988000	1.653793000	-0.583729000

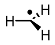

C	-0.000027000	-0.000112000	0.001161000
H	-1.071886000	-0.198399000	0.000900000
H	0.364091000	1.027302000	0.000899000
H	0.708100000	-0.828743000	0.000899000

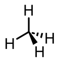

C	-0.031352000	-0.027586000	-0.106605000
H	-1.062503000	-0.296943000	0.157063000
H	0.268623000	0.865196000	0.456922000
H	0.638779000	-0.860603000	0.142650000
H	0.028126000	0.180773000	-1.182578000

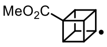

C	0.679422000	-0.294172000	0.104445000
C	1.924353000	-1.036702000	-0.478397000
C	1.608431000	0.873197000	0.571499000
H	-0.279696000	-0.153819000	-0.406116000
H	1.968443000	-1.540222000	-1.449747000
C	2.850680000	0.133408000	-0.017605000
H	3.623599000	0.615948000	-0.625741000
C	3.071335000	-0.533472000	1.379733000
C	2.124287000	-1.653656000	0.917513000
C	1.815285000	0.206504000	1.952746000
H	4.036837000	-0.633692000	1.887036000
H	1.764627000	0.734462000	2.911038000
C	0.879838000	-0.965754000	1.502730000
H	0.084442000	-1.413415000	2.108221000
C	1.314811000	2.312241000	0.341605000
O	1.162304000	3.142867000	1.200803000
O	1.238670000	2.581568000	-0.963510000
C	0.957528000	3.931996000	-1.316098000
H	0.937589000	3.967473000	-2.409993000
H	-0.014875000	4.243431000	-0.910515000
H	1.737200000	4.603868000	-0.931772000

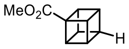

C	0.685090000	-0.290513000	0.103418000
C	1.927727000	-1.048746000	-0.454640000
C	1.608216000	0.887857000	0.561751000
H	-0.272665000	-0.155172000	-0.410438000
H	1.972998000	-1.560647000	-1.421931000
C	2.847550000	0.131487000	-0.017827000
H	3.622812000	0.605751000	-0.629054000
C	3.056699000	-0.542119000	1.374544000
C	2.137342000	-1.723719000	0.936555000
C	1.814327000	0.217406000	1.942483000
H	4.021255000	-0.643318000	1.884012000
H	1.763987000	0.747796000	2.899302000
C	0.894823000	-0.964108000	1.495454000
H	0.099049000	-1.409409000	2.102619000
C	1.313674000	2.326351000	0.333494000
O	1.156837000	3.156571000	1.193559000
O	1.238979000	2.603145000	-0.971723000
C	0.951659000	3.953561000	-1.316206000
H	0.933744000	3.996816000	-2.409969000
H	-0.023258000	4.258044000	-0.910999000
H	1.726164000	4.628188000	-0.925973000
H	2.353286000	-2.785615000	1.098245000

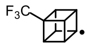

C	0.840880000	-0.411320000	-0.113882000
C	2.222463000	-1.089661000	-0.395970000
C	1.596442000	0.812867000	0.472424000
H	0.001900000	-0.348713000	-0.815558000
H	2.492055000	-1.615303000	-1.317811000
C	1.271117000	2.224846000	0.155824000
F	0.018671000	2.538730000	0.511270000
F	1.383436000	2.481909000	-1.153539000
F	2.083424000	3.077156000	0.793898000
C	2.973451000	0.150375000	0.193585000
H	3.828870000	0.658523000	-0.264874000
C	2.934201000	-0.464138000	1.632478000
C	2.164925000	-1.654422000	1.033703000
C	1.548730000	0.210461000	1.903141000
H	3.775155000	-0.488464000	2.333177000
H	1.273096000	0.768485000	2.804639000
C	0.789552000	-1.028836000	1.323116000
H	-0.085672000	-1.504199000	1.777905000

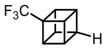

C	0.842451000	-0.401640000	-0.112716000
C	2.219271000	-1.088357000	-0.377808000
C	1.592102000	0.830635000	0.468138000
H	0.003382000	-0.343379000	-0.814136000
H	2.489424000	-1.616166000	-1.298535000

C	1.267205000	2.242255000	0.151983000
F	0.013518000	2.559016000	0.505274000
F	1.380647000	2.503160000	-1.157802000
F	2.078019000	3.097167000	0.791150000
C	2.966644000	0.158126000	0.193025000
H	3.823935000	0.662720000	-0.265076000
C	2.921357000	-0.471611000	1.621272000
C	2.180207000	-1.719482000	1.048422000
C	1.547879000	0.217934000	1.896200000
H	3.762379000	-0.498338000	2.322186000
H	1.272851000	0.772262000	2.799753000
C	0.807365000	-1.028686000	1.316987000
H	-0.066688000	-1.506426000	1.772161000
H	2.420503000	-2.761543000	1.285069000

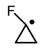

C	0.675951000	-0.390835000	-0.017485000
C	2.223099000	-0.390480000	-0.018036000
C	1.449290000	0.837998000	0.112107000
H	0.177182000	-0.613379000	-0.964376000
H	0.178855000	-0.768426000	0.881224000
H	2.721298000	-0.612790000	-0.965282000
H	2.721018000	-0.767833000	0.880318000
F	1.449508000	1.587068000	1.219702000

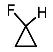

C	0.692839000	-0.374626000	-0.014756000
C	2.206206000	-0.374278000	-0.015297000
C	1.449285000	0.893603000	0.134997000
H	0.162204000	-0.553706000	-0.951313000
H	0.206041000	-0.750718000	0.887514000
H	2.736261000	-0.553116000	-0.952228000
H	2.693824000	-0.750132000	0.886629000
F	1.449594000	1.472972000	1.374412000
H	1.448820000	1.640535000	-0.661936000

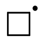

C	0.790314000	-0.465593000	0.192482000
C	2.088765000	-1.191366000	-0.260020000
C	1.640153000	0.720770000	0.531815000
H	0.041226000	-0.309224000	-0.604267000
H	0.266005000	-0.938369000	1.042340000
H	2.113805000	-1.445768000	-1.327690000
H	2.333785000	-2.088116000	0.324088000
H	1.409016000	1.707342000	0.937895000
C	2.922107000	0.066315000	0.115753000
H	3.453016000	0.543678000	-0.727333000
H	3.663789000	-0.089240000	0.919670000

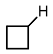

C	0.829094000	-0.497086000	0.312788000
C	2.066822000	-1.114271000	-0.372334000
C	1.612403000	0.828144000	0.424507000
H	-0.121118000	-0.493008000	-0.239850000
H	0.662125000	-0.943334000	1.304390000
H	2.006990000	-1.010173000	-1.465772000
H	2.330214000	-2.153299000	-0.128053000
H	1.493776000	1.424669000	1.340163000
C	2.913662000	0.017539000	0.247781000
H	3.718882000	0.455468000	-0.359040000
H	3.326753000	-0.285714000	1.221171000
H	1.425412000	1.472900000	-0.446862000

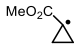

C	0.752803000	-0.227127000	-0.392144000
C	2.026937000	-0.644580000	0.351086000
C	1.476136000	0.694657000	0.465371000
H	0.834262000	-0.110908000	-1.477981000
H	-0.213452000	-0.605612000	-0.041503000
H	2.923845000	-0.792101000	-0.259717000
H	1.944327000	-1.314788000	1.213341000
C	1.201911000	1.728431000	1.440935000
O	0.238793000	2.463498000	1.411541000
O	2.155275000	1.806628000	2.379498000
C	1.973973000	2.819239000	3.360847000
H	2.808634000	2.724587000	4.062930000
H	1.984251000	3.816180000	2.897926000
H	1.020638000	2.684210000	3.890385000

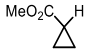

C	0.842715000	0.175488000	-0.464037000
C	1.647976000	-0.869340000	0.218427000
C	1.790413000	0.572718000	0.642319000
H	1.121466000	0.472399000	-1.476000000
H	-0.220875000	0.231446000	-0.223989000
H	2.493507000	-1.308820000	-0.312426000
H	1.133208000	-1.523731000	0.924437000
C	1.168981000	0.938774000	1.939554000
O	0.160930000	0.444392000	2.383365000
O	1.854969000	1.889647000	2.575372000
C	1.335005000	2.302881000	3.834428000
H	2.010790000	3.078978000	4.208030000
H	0.320645000	2.709111000	3.721508000
H	1.307349000	1.459201000	4.537607000
H	2.702789000	1.110124000	0.386292000

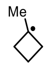

C	0.805490000	-0.485488000	0.224796000
C	2.094718000	-1.177372000	-0.292504000
C	1.615984000	0.769547000	0.399827000
H	-0.049821000	-0.442953000	-0.471736000
H	0.433597000	-0.907111000	1.178241000
H	2.087584000	-1.335662000	-1.379014000
H	2.363750000	-2.119576000	0.202446000
C	1.323693000	2.011836000	1.154618000
H	0.294525000	2.360767000	0.976774000

H	2.015363000	2.824529000	0.882558000
H	1.425517000	1.858160000	2.248003000
C	2.918980000	0.067839000	0.130215000
H	3.583857000	0.507616000	-0.633456000
H	3.526608000	-0.098489000	1.040132000

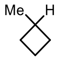

C	0.836042000	-0.554685000	0.296342000
C	2.084518000	-1.138840000	-0.396223000
C	1.584411000	0.793021000	0.411102000
H	-0.120383000	-0.574537000	-0.245984000
H	0.691365000	-0.999351000	1.293796000
H	2.019036000	-1.034355000	-1.489016000
H	2.373757000	-2.171006000	-0.153781000
C	1.394417000	1.642638000	1.651238000
H	0.367259000	2.034484000	1.714914000
H	2.081819000	2.502910000	1.657183000
H	1.586911000	1.050975000	2.560376000
C	2.904018000	0.010241000	0.226571000
H	3.702674000	0.470043000	-0.373474000
H	3.315584000	-0.282890000	1.205468000
H	1.389340000	1.390178000	-0.494668000

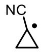

C	0.690296000	-0.436346000	0.027207000
C	2.208980000	-0.435750000	0.026556000
C	1.449336000	0.752400000	0.411625000
H	0.187486000	-0.420370000	-0.945138000
H	0.162239000	-0.977437000	0.818353000
H	2.710936000	-0.419370000	-0.946223000
H	2.738120000	-0.976446000	0.817247000
C	1.449275000	1.832590000	1.272007000
N	1.449154000	2.775475000	1.958252000

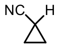

C	0.690352000	-0.439771000	0.042774000
C	2.178506000	-0.405383000	0.051322000
C	1.403101000	0.857776000	0.350233000
H	0.172061000	-0.478690000	-0.915937000
H	0.186031000	-0.935832000	0.873458000
H	2.708821000	-0.419783000	-0.901434000
H	2.695330000	-0.877979000	0.887901000
C	1.384861000	1.362172000	1.700396000
N	1.370676000	1.763001000	2.783912000
H	1.388531000	1.640871000	-0.410442000

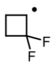

C	0.791573000	-0.464611000	0.187944000
C	2.088761000	-1.200655000	-0.258931000
C	1.631691000	0.726099000	0.538481000
H	0.055330000	-0.297107000	-0.615016000
H	0.265312000	-0.930907000	1.036794000
H	2.148990000	-1.435451000	-1.328694000
H	2.361504000	-2.075414000	0.344058000
H	1.431319000	1.717621000	0.945567000
C	2.886895000	0.049292000	0.117197000
F	3.561877000	0.637009000	-0.911495000
F	3.823433000	-0.129139000	1.091978000

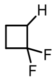

C	0.790999000	-0.402860000	0.078077000
C	2.094955000	-1.211801000	-0.137539000
C	1.630968000	0.737994000	0.706764000
H	0.334759000	-0.099234000	-0.872619000
H	0.031179000	-0.861828000	0.720586000
H	2.255270000	-1.714674000	-1.099854000
H	2.298910000	-1.897381000	0.695052000
H	1.693037000	0.652547000	1.799369000
C	2.847047000	0.093222000	0.063093000
F	3.168099000	0.683851000	-1.121821000
F	3.994647000	0.055675000	0.781692000
H	1.425015000	1.774664000	0.411296000

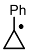

C	0.658416000	-0.261112000	-0.222304000
C	2.118260000	-0.576352000	0.128278000
C	1.437441000	0.592738000	0.658180000
H	0.444490000	0.078573000	-1.242739000
H	-0.119943000	-0.900906000	0.210836000
H	2.868982000	-0.445064000	-0.660121000
H	2.311217000	-1.425220000	0.795266000
C	1.462397000	1.697798000	1.521639000
C	0.314332000	2.522296000	1.683183000
C	0.343739000	3.616442000	2.534851000
C	1.507016000	3.930242000	3.250656000
C	2.647545000	3.129875000	3.103708000
C	2.634378000	2.031689000	2.256369000
H	-0.591994000	2.277836000	1.124887000
H	-0.547322000	4.237536000	2.646724000
H	1.524285000	4.793305000	3.918078000
H	3.555571000	3.371701000	3.659954000
H	3.524447000	1.409060000	2.142326000

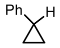

C	1.233087000	-0.877658000	0.063040000
C	2.291871000	0.171947000	-0.013091000
C	0.928561000	0.523679000	0.529345000
H	0.766459000	-1.211222000	-0.865690000
H	1.326961000	-1.645495000	0.834949000
H	2.560104000	0.566691000	-0.994780000
H	3.111524000	0.126271000	0.708235000
C	0.744213000	0.821768000	1.978674000
C	-0.165122000	1.812123000	2.374062000
C	-0.354482000	2.117226000	3.722561000

C	0.363812000	1.434627000	4.704163000
C	1.272253000	0.444926000	4.323191000
C	1.460662000	0.141218000	2.975908000
H	-0.730496000	2.351167000	1.610102000
H	-1.067288000	2.894085000	4.006535000
H	0.217631000	1.670775000	5.759678000
H	1.839696000	-0.096801000	5.082635000
H	2.176861000	-0.637559000	2.700723000
H	0.291965000	1.120184000	-0.127687000

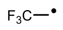

C	1.333397000	0.863408000	0.069590000
C	1.369683000	1.701950000	1.281686000
F	0.214633000	2.348268000	1.485080000
F	2.324692000	2.649220000	1.220791000
F	1.622201000	0.985959000	2.384443000
H	0.800819000	1.239633000	-0.803018000
H	2.019919000	0.020436000	0.003460000

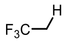

C	1.197499000	0.754895000	0.110809000
C	1.343137000	1.673410000	1.278189000
F	0.227597000	2.379268000	1.511591000
F	2.329710000	2.564082000	1.102808000
F	1.621049000	1.013080000	2.411591000
H	0.973097000	1.345850000	-0.785246000
H	2.135285000	0.204708000	-0.030999000
H	0.379559000	0.051562000	0.306687000

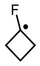

C	0.784292000	-0.459209000	0.185718000
C	2.079256000	-1.159975000	-0.310778000
C	1.613653000	0.783682000	0.232401000
H	-0.097788000	-0.479194000	-0.471811000
H	0.486317000	-0.788557000	1.196504000
H	2.079650000	-1.317537000	-1.396230000
H	2.331852000	-2.098369000	0.196185000
C	2.923799000	0.075073000	0.108488000
H	3.664622000	0.460373000	-0.608177000
H	3.409329000	-0.057791000	1.091061000
F	1.391042000	1.800621000	1.079563000

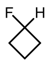

C	0.812003000	-0.454529000	0.220218000
C	2.073472000	-1.143706000	-0.347813000
C	1.590694000	0.854825000	0.128828000
H	-0.139151000	-0.540565000	-0.321163000
H	0.668617000	-0.716497000	1.278877000
H	2.045982000	-1.192830000	-1.444919000
H	2.335861000	-2.131487000	0.050670000
C	2.898031000	0.067528000	0.143896000
H	3.738185000	0.429247000	-0.463154000
H	3.220149000	-0.078290000	1.185494000
F	1.400134000	1.766993000	1.146487000
H	1.425128000	1.376589000	-0.825793000

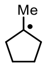

C	2.415436000	0.727586000	-0.211088000
C	2.386146000	-0.734774000	0.249471000
C	0.985374000	-1.226507000	-0.158066000
C	0.141913000	0.018179000	-0.139451000
C	0.997512000	1.226532000	0.103078000
C	-1.341044000	0.008963000	-0.034275000
H	-1.781087000	0.952923000	-0.392641000
H	-1.681527000	-0.122137000	1.013165000
H	-1.784821000	-0.817479000	-0.612164000
H	3.205669000	1.317806000	0.274260000
H	2.584804000	0.769307000	-1.299358000
H	3.200724000	-1.339513000	-0.173599000
H	2.480448000	-0.770692000	1.346836000
H	0.597087000	-2.011888000	0.514421000
H	1.004791000	-1.678412000	-1.169967000
H	0.694799000	2.098799000	-0.500968000
H	0.933024000	1.550434000	1.163584000

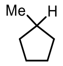

C	2.417920000	0.784279000	-0.152526000
C	2.446435000	-0.756894000	-0.003358000
C	0.970264000	-1.187620000	0.162063000
C	0.133547000	0.044427000	-0.215837000
C	1.009790000	1.194862000	0.287504000
C	-1.284696000	0.029923000	0.331664000
H	-1.840304000	0.933110000	0.036455000
H	-1.273995000	-0.014361000	1.432877000
H	-1.847105000	-0.843183000	-0.033404000
H	3.211157000	1.283408000	0.421844000
H	2.560097000	1.061127000	-1.208445000
H	2.909027000	-1.227672000	-0.882479000
H	3.042977000	-1.062731000	0.868093000
H	0.762567000	-1.437440000	1.215965000
H	0.709385000	-2.072831000	-0.436629000
H	0.700272000	2.178360000	-0.097576000
H	0.948080000	1.232057000	1.389741000
H	0.094767000	0.112051000	-1.318348000

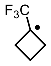

C	0.783488000	-0.446788000	0.158671000
C	2.086900000	-1.186832000	-0.253040000
C	1.625356000	0.772086000	0.372708000
H	0.004838000	-0.363123000	-0.616260000
H	0.310878000	-0.825810000	1.080566000
H	2.118915000	-1.465242000	-1.313493000
H	2.325111000	-2.065531000	0.358306000
C	1.360338000	1.949044000	1.218328000
F	0.110503000	2.405844000	1.062562000

F	2.198485000	2.960623000	0.954436000
F	1.499638000	1.683302000	2.534481000
C	2.921290000	0.080505000	0.084931000
H	3.519736000	0.502731000	-0.738019000
H	3.574754000	-0.019037000	0.968007000

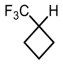

C	0.822805000	-0.556785000	0.251710000
C	2.089512000	-1.136092000	-0.413854000
C	1.591324000	0.766350000	0.435192000
H	-0.105349000	-0.523220000	-0.332675000
H	0.626895000	-1.030751000	1.224332000
H	2.054824000	-1.018464000	-1.505598000
H	2.361894000	-2.171262000	-0.170622000
C	1.397765000	1.506362000	1.722102000
F	0.154556000	1.995239000	1.834614000
F	2.239034000	2.543663000	1.835069000
F	1.602257000	0.730869000	2.796098000
C	2.910844000	-0.006765000	0.245244000
H	3.696798000	0.480204000	-0.345635000
H	3.322796000	-0.321407000	1.214916000
H	1.403511000	1.475942000	-0.382421000

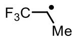

C	2.763464000	0.616911000	-0.534701000
C	1.460285000	0.827853000	0.138798000
H	3.463534000	1.436188000	-0.313319000
H	3.239954000	-0.319411000	-0.188070000
C	1.431087000	1.279399000	1.546097000
F	0.200511000	1.613623000	1.947964000
F	2.219799000	2.345264000	1.756192000
F	1.868410000	0.329724000	2.399660000
H	2.635999000	0.538743000	-1.621709000
H	0.528474000	0.412656000	-0.247770000

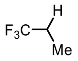

C	2.797182000	0.617579000	-0.512768000
C	1.432159000	1.024972000	0.021179000
H	3.539563000	1.409356000	-0.341196000
H	3.151136000	-0.302255000	-0.026761000
C	1.454731000	1.293670000	1.496845000
F	0.249327000	1.648461000	1.960789000
F	2.299114000	2.283904000	1.821479000
F	1.838726000	0.220388000	2.203829000
H	2.738569000	0.432736000	-1.592776000
H	0.682200000	0.238225000	-0.143003000
H	1.066615000	1.943579000	-0.459503000

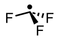

C	-0.757685000	0.274854000	0.678249000
F	0.073163000	0.956821000	1.427421000
F	-1.657133000	1.054400000	0.132844000
F	-1.317686000	-0.695670000	1.356169000

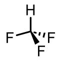

C	-0.729574000	0.253277000	0.635156000
F	0.055037000	0.964833000	1.441840000
F	-1.670525000	1.064437000	0.159247000
F	-1.329588000	-0.679951000	1.371203000
H	-0.150571000	-0.199965000	-0.179135000

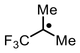

C	0.235857000	-0.173203000	0.101329000
C	2.803703000	0.093157000	-0.109758000
C	1.476657000	0.623698000	0.304418000
H	0.196934000	-0.569795000	-0.923680000
H	-0.669096000	0.421426000	0.286113000
H	3.552838000	0.890891000	-0.205597000
H	3.187694000	-0.634284000	0.631048000
C	1.441607000	1.662177000	1.364502000
F	0.351531000	2.440266000	1.287934000
F	2.505576000	2.474448000	1.330728000
F	1.420408000	1.124821000	2.604769000
H	0.211775000	-1.043476000	0.784442000
H	2.719740000	-0.435643000	-1.069436000

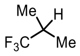

C	0.258001000	-0.165488000	0.096060000
C	2.776315000	0.080845000	-0.080491000
C	1.439529000	0.799100000	0.074170000
H	0.228666000	-0.735655000	-0.841956000
H	-0.696198000	0.368207000	0.203933000
H	3.613969000	0.790876000	-0.107180000
H	2.938311000	-0.620787000	0.751351000
C	1.443950000	1.640280000	1.327230000
F	0.309168000	2.337965000	1.472156000
F	2.445465000	2.530662000	1.336028000
F	1.581701000	0.899445000	2.436875000
H	0.355250000	-0.880032000	0.927055000
H	2.781914000	-0.492262000	-1.017187000
H	1.311156000	1.521840000	-0.747056000

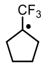

C	-1.430495000	0.790682000	0.596119000
C	-1.567303000	-0.697719000	0.252902000
C	-0.117163000	-1.212927000	0.230546000
C	0.659703000	-0.000139000	-0.177218000
C	-0.183769000	1.234369000	-0.186284000
C	1.980670000	-0.084155000	-0.824820000
F	2.672841000	1.059844000	-0.727358000
F	1.896946000	-0.353718000	-2.146798000
F	2.741682000	-1.057205000	-0.301818000
H	-2.324071000	1.377277000	0.344046000
H	-1.242511000	0.907228000	1.675112000
H	-2.205312000	-1.248646000	0.956758000

H	-2.004343000	-0.803908000	-0.752633000
H	0.036331000	-2.065652000	-0.450306000
H	0.200382000	-1.550309000	1.235143000
H	0.327077000	2.109033000	0.245404000
H	-0.448571000	1.502395000	-1.227904000

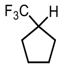

C	-1.416590000	0.770966000	0.543772000
C	-1.521431000	-0.701343000	0.147385000
C	-0.091128000	-1.209896000	0.349026000
C	0.812634000	-0.021357000	-0.062115000
C	-0.111732000	1.220838000	-0.123237000
C	1.477227000	-0.272005000	-1.386720000
F	2.198023000	0.780641000	-1.798005000
F	0.591132000	-0.533235000	-2.361106000
F	2.314215000	-1.318207000	-1.345146000
H	-2.280989000	1.374738000	0.234106000
H	-1.325657000	0.850432000	1.639135000
H	-2.259834000	-1.264266000	0.735077000
H	-1.802937000	-0.779006000	-0.915245000
H	0.134584000	-2.126637000	-0.214467000
H	0.077794000	-1.431632000	1.411954000
H	0.343609000	2.096874000	0.356080000
H	-0.311399000	1.486832000	-1.173093000
H	1.634637000	0.119598000	0.653203000

C	1.365939000	0.907250000	0.124047000
H	0.661269000	1.173558000	-0.664014000
H	2.095817000	0.110654000	-0.021544000
C	1.337547000	1.599775000	1.335949000
N	1.312679000	2.178915000	2.345804000

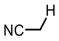

C	1.255258000	0.830751000	-0.051707000
H	0.612690000	1.391899000	-0.741967000
H	2.262461000	0.747470000	-0.479310000
C	1.323056000	1.527509000	1.226769000
N	1.376167000	2.079201000	2.238453000
H	0.839197000	-0.173595000	0.097921000

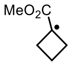

C	1.018646000	-0.555082000	0.716684000
C	2.008919000	-1.146225000	-0.326775000
C	1.864438000	0.675979000	0.729111000
H	-0.015898000	-0.402908000	0.367329000
H	0.972679000	-1.088112000	1.681040000
H	1.562409000	-1.322413000	-1.313090000
H	2.527784000	-2.055176000	0.000969000
C	2.865249000	0.150248000	-0.245366000
H	2.952056000	0.729093000	-1.179646000
H	3.880248000	0.021706000	0.166323000
C	1.772960000	1.949565000	1.400233000
O	2.570653000	2.858177000	1.270660000
O	0.692817000	2.030374000	2.197499000
C	0.513745000	3.261764000	2.880033000
H	-0.407289000	3.162126000	3.464165000
H	1.360657000	3.467821000	3.549894000
H	0.416274000	4.093282000	2.167529000

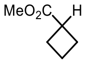

C	0.928573000	-0.582325000	0.359855000
C	2.226342000	-1.226121000	-0.170178000
C	1.775572000	0.654710000	0.764481000
H	0.237806000	-0.332189000	-0.458697000
H	0.376930000	-1.096320000	1.157820000
H	2.168000000	-1.787556000	-1.112208000
H	2.696431000	-1.857889000	0.596463000
C	2.863812000	0.178673000	-0.209239000
H	2.729197000	0.662730000	-1.186785000
H	3.910185000	0.283089000	0.107547000
C	1.148768000	1.997506000	0.523380000
O	1.397594000	2.731772000	-0.399322000
O	0.226319000	2.279265000	1.447203000
C	-0.467027000	3.511891000	1.291020000
H	-1.163561000	3.584080000	2.132738000
H	0.235224000	4.356468000	1.309911000
H	-1.021645000	3.529917000	0.342582000
H	2.084281000	0.582084000	1.816968000

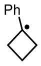

C	0.823516000	-0.166398000	-0.245092000
C	2.029651000	-1.143475000	-0.315082000
C	1.649320000	0.705464000	0.656632000
H	0.538527000	0.295658000	-1.205921000
H	-0.093451000	-0.576959000	0.211213000
H	2.449018000	-1.275641000	-1.320365000
H	1.833465000	-2.130663000	0.121955000
C	2.849889000	-0.195629000	0.603181000
H	3.744890000	0.249725000	0.135603000
H	3.159586000	-0.623836000	1.571828000
C	1.383707000	1.921263000	1.326395000
C	0.123148000	2.565518000	1.198959000
C	-0.133917000	3.758744000	1.859774000
C	0.845964000	4.350591000	2.667429000
C	2.094373000	3.729578000	2.805228000
C	2.365397000	2.536710000	2.149346000
H	-0.645711000	2.109117000	0.570908000
H	-1.108625000	4.238529000	1.748026000
H	0.638571000	5.287942000	3.186161000
H	2.861371000	4.186335000	3.434248000
H	3.341051000	2.057807000	2.262176000

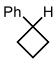

C	0.808535000	-0.324407000	-0.149073000

C	2.135851000	-1.019218000	-0.517107000
C	1.635664000	0.721996000	0.632566000
H	0.159955000	0.028363000	-0.962880000
H	0.215019000	-0.940279000	0.543978000
H	2.528749000	-0.645962000	-1.473793000
H	2.153715000	-2.117317000	-0.526006000
C	2.794570000	-0.298511000	0.677243000
H	3.820616000	0.076833000	0.560146000
H	2.735792000	-0.908487000	1.591787000
C	1.093056000	1.299591000	1.911143000
C	-0.267326000	1.612762000	2.031899000
C	-0.772246000	2.165063000	3.209272000
C	0.077557000	2.410787000	4.289343000
C	1.433595000	2.099250000	4.181919000
C	1.935395000	1.546153000	3.002850000
H	-0.939808000	1.418774000	1.192162000
H	-1.835583000	2.401325000	3.285165000
H	-0.317046000	2.839277000	5.212557000
H	2.105648000	2.284645000	5.022339000
H	2.997921000	1.300644000	2.928283000
H	1.908728000	1.544214000	-0.050918000

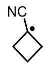

C	0.787806000	-0.453999000	0.219116000
C	2.082127000	-1.164977000	-0.261578000
C	1.644460000	0.714273000	0.612835000
H	0.050859000	-0.228055000	-0.568046000
H	0.258916000	-0.935079000	1.057043000
H	2.100347000	-1.392171000	-1.333950000
H	2.328723000	-2.070843000	0.304849000
C	2.920192000	0.078473000	0.142233000
H	3.408753000	0.611400000	-0.689169000
H	3.669254000	-0.081758000	0.933765000
C	1.361129000	1.942387000	1.209907000
N	1.125154000	2.969910000	1.708739000

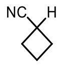

C	0.793960000	-0.401525000	0.147164000
C	2.091224000	-1.177798000	-0.154287000
C	1.666258000	0.662506000	0.860688000
H	0.348644000	0.012082000	-0.767755000
H	0.017014000	-0.887414000	0.750406000
H	2.175287000	-1.664305000	-1.134267000
H	2.307301000	-1.910756000	0.635122000
C	2.882644000	0.127147000	0.062978000
H	3.007060000	0.684636000	-0.875239000
H	3.840097000	0.081232000	0.596784000
C	1.304405000	2.068419000	0.715399000
N	1.019497000	3.178003000	0.569237000
H	1.766758000	0.436894000	1.931455000

8.6.2 Reaction mechanism

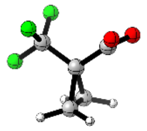

IIIcp

C	0.697626000	-0.388105000	0.008444000
C	2.187640000	-0.369911000	-0.019344000
C	1.430035000	0.930248000	-0.016226000
H	0.167818000	-0.670565000	-0.902101000
H	0.196607000	-0.642404000	0.943502000
H	2.695238000	-0.629840000	-0.949571000
H	2.727645000	-0.607019000	0.899561000
C	1.446324000	1.766383000	1.232789000
F	0.395358000	2.591337000	1.277232000
F	2.540348000	2.527568000	1.302705000
F	1.413282000	1.010235000	2.329151000
C	1.463003000	1.775017000	-1.265907000
O	2.424118000	2.292864000	-1.762246000
O	0.254174000	1.906671000	-1.764535000

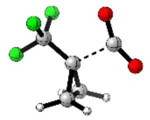

TS1cp

C	0.806151000	-0.426294000	-0.036150000
C	2.320781000	-0.257756000	0.037876000
C	1.420795000	0.903995000	0.082445000
H	0.425566000	-0.725462000	-1.014839000
H	0.248743000	-0.791114000	0.830398000
H	2.850881000	-0.455138000	-0.896081000
H	2.859446000	-0.500078000	0.957628000
C	1.266373000	1.762457000	1.302051000
F	0.113038000	2.424656000	1.313968000
F	2.248652000	2.650283000	1.426254000
F	1.290947000	0.987904000	2.395487000
C	1.399800000	1.854411000	-1.527348000
O	1.517285000	1.086076000	-2.421991000
O	1.270760000	2.978540000	-1.136247000

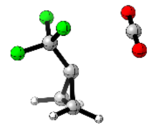

IVcp-CO_2-prodC

C	0.685357000	-0.634545000	0.150606000
C	2.146677000	-0.365235000	-0.182025000
C	1.187089000	0.727580000	-0.025409000
H	0.069219000	-1.059844000	-0.646660000
H	0.422650000	-0.950846000	1.164929000
H	2.472817000	-0.614128000	-1.196063000
H	2.901749000	-0.498172000	0.599195000
C	1.209055000	1.836868000	0.946521000

F	0.013625000	2.412943000	1.089303000
F	2.067540000	2.802437000	0.581891000
F	1.591736000	1.420388000	2.164020000
C	1.757329000	2.839145000	-2.154190000
O	2.847760000	2.500862000	-2.335563000
O	0.664909000	3.180051000	-1.982733000

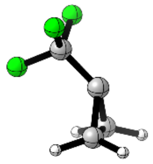

IVcp

C	0.688936000	-0.397537000	-0.003243000
C	2.211126000	-0.397747000	-0.003143000
C	1.450212000	0.845380000	0.123974000
H	0.197719000	-0.601903000	-0.958835000
H	0.160040000	-0.764247000	0.882171000
H	2.702673000	-0.602162000	-0.958546000
H	2.739647000	-0.764526000	0.882461000
C	1.450259000	1.796399000	1.251765000
F	0.375123000	2.591414000	1.247324000
F	2.525415000	2.591465000	1.247296000
F	1.450224000	1.152516000	2.432324000

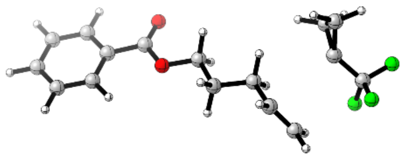

IVcp-alkene-reactC

C	-1.615121000	-0.875352000	-0.573217000
C	-0.240704000	-0.912216000	-0.815449000
C	0.550861000	0.196270000	-0.520456000
C	-0.038674000	1.345162000	0.018775000
C	-1.415906000	1.381136000	0.261269000
C	-2.203110000	0.270993000	-0.033991000
H	-2.231547000	-1.745419000	-0.807352000
H	0.216134000	-1.808639000	-1.237909000
H	1.624683000	0.175571000	-0.707372000
H	-1.855140000	2.286918000	0.682294000
H	-3.277395000	0.298106000	0.155306000
C	0.761854000	2.563229000	0.349038000
O	0.291411000	3.577884000	0.800754000
O	2.059427000	2.413375000	0.091029000
C	2.902861000	3.534074000	0.369660000
C	4.325308000	3.152100000	0.026324000
H	2.803432000	3.802733000	1.432794000
H	2.563217000	4.396160000	-0.225299000
C	5.282887000	4.321198000	0.263019000
H	4.625196000	2.286823000	0.639137000
H	4.371813000	2.838747000	-1.028559000
H	5.001107000	5.174169000	-0.375815000
H	5.178579000	4.657105000	1.310793000
C	6.715662000	3.955767000	0.008698000
C	7.505172000	4.554546000	-0.884308000
H	7.115962000	3.130953000	0.611510000
H	8.544824000	4.248496000	-1.026020000
H	7.134336000	5.379069000	-1.502077000
C	5.346351000	7.786054000	1.607135000
C	6.503236000	7.250135000	2.437552000
C	6.545497000	7.233407000	0.975178000
H	4.432942000	7.183955000	1.582816000
H	5.194098000	8.868697000	1.556509000
H	6.345047000	6.298344000	2.953893000
H	7.149866000	7.962740000	2.959286000
C	7.501030000	7.908139000	0.078709000
F	7.717345000	9.179856000	0.460668000
F	7.060736000	7.957709000	-1.185294000
F	8.694288000	7.306619000	0.054681000

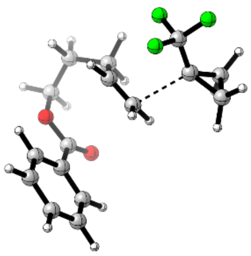

TS2cp

C	-3.715126000	-3.034794000	0.340700000
C	-3.629557000	-2.081816000	1.357592000
C	-3.255624000	-0.773010000	1.058726000
C	-2.965764000	-0.418982000	-0.263442000
C	-3.049604000	-1.374648000	-1.281331000
C	-3.426159000	-2.680981000	-0.979074000
H	-4.006191000	-4.059779000	0.578456000
H	-3.853358000	-2.360382000	2.388734000
H	-3.180229000	-0.023681000	1.846938000
H	-2.814246000	-1.079711000	-2.305166000
H	-3.493535000	-3.427052000	-1.772663000
C	-2.529743000	0.962589000	-0.632562000
O	-2.203902000	1.283492000	-1.748630000
O	-2.542302000	1.792399000	0.410962000
C	-2.045306000	3.125945000	0.239357000
C	-0.656647000	3.266411000	0.834138000
H	-2.756754000	3.780399000	0.759695000
H	-2.047214000	3.374845000	-0.829532000
C	0.401702000	2.360782000	0.195461000
H	-0.363109000	4.320136000	0.712284000
H	-0.702548000	3.072248000	1.918266000
H	0.365796000	2.441770000	-0.903377000
H	1.395030000	2.727622000	0.510104000
C	0.312561000	0.921375000	0.604965000
C	0.445671000	-0.126573000	-0.235252000
H	0.222515000	0.728666000	1.681299000
H	0.358056000	-1.152441000	0.130958000
H	0.448341000	0.020350000	-1.319722000
C	3.197098000	-1.437210000	-1.433352000
C	3.458242000	0.040442000	-1.671008000

C	2.806355000	-0.427638000	-0.442609000
H	2.400895000	-1.907219000	-2.019174000
H	4.049717000	-2.084620000	-1.204880000
H	2.837462000	0.547177000	-2.416645000
H	4.489874000	0.401342000	-1.604093000
C	3.320907000	-0.284015000	0.928172000
F	4.625595000	-0.610434000	1.020929000
F	2.673520000	-1.063866000	1.803905000
F	3.216671000	0.974123000	1.381120000

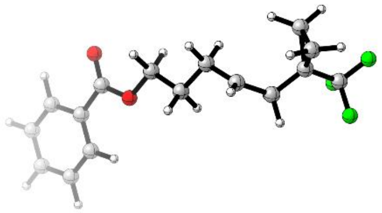

Vcp

C	-1.025296000	-1.248175000	-1.386432000
C	0.342521000	-0.968298000	-1.396272000
C	0.821653000	0.198225000	-0.802788000
C	-0.074510000	1.086951000	-0.198446000
C	-1.444896000	0.805518000	-0.189191000
C	-1.919293000	-0.361331000	-0.782372000
H	-1.397417000	-2.163390000	-1.850839000
H	1.039818000	-1.663143000	-1.867173000
H	1.888403000	0.423554000	-0.806152000
H	-2.127081000	1.512078000	0.286100000
H	-2.988121000	-0.581072000	-0.774887000
C	0.381742000	2.355023000	0.447831000
O	-0.361775000	3.154224000	0.961085000
O	1.702562000	2.515229000	0.398452000
C	2.225218000	3.705430000	0.996549000
C	3.733269000	3.667043000	0.892054000
H	1.896630000	3.752650000	2.046091000
H	1.806646000	4.581196000	0.476320000
C	4.364920000	4.923039000	1.515051000
H	4.110445000	2.767522000	1.403454000
H	4.024242000	3.590175000	-0.167861000
H	3.974283000	5.809019000	0.984194000
H	4.031223000	5.001152000	2.562786000
C	5.855700000	4.912301000	1.449274000
C	6.584900000	5.373834000	0.230423000
H	6.417495000	4.414553000	2.243265000
H	7.410758000	4.683094000	-0.007672000
H	5.900116000	5.373724000	-0.634505000
C	6.489167000	7.862570000	1.121326000
C	7.769024000	7.274345000	1.636354000
C	7.182113000	6.779726000	0.347903000
H	5.559841000	7.584526000	1.621853000
H	6.522233000	8.877159000	0.719981000
H	7.713530000	6.591130000	2.485423000
H	8.668767000	7.890397000	1.586102000
C	7.883294000	7.208570000	-0.903072000
F	8.404561000	8.438607000	-0.814027000
F	7.058294000	7.217070000	-1.961488000
F	8.893337000	6.384533000	-1.219890000

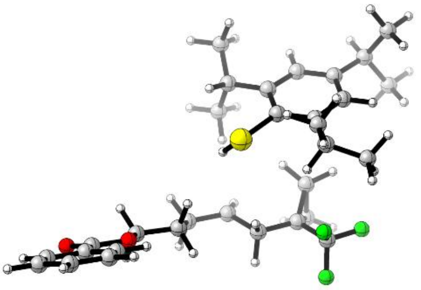

Vcp-thiol-reactC

C	-1.344187000	0.185977000	-3.463662000
C	-0.067096000	0.719754000	-3.280524000
C	0.242339000	1.404453000	-2.106646000
C	-0.732743000	1.554338000	-1.113974000
C	-2.012678000	1.020635000	-1.299184000
C	-2.317399000	0.336865000	-2.473245000
H	-1.582425000	-0.352035000	-4.383137000
H	0.691867000	0.600794000	-4.055499000
H	1.237857000	1.823146000	-1.956656000
H	-2.758303000	1.148363000	-0.512901000
H	-3.315002000	-0.081483000	-2.617078000
C	-0.453359000	2.269265000	0.168321000
O	-1.264406000	2.414112000	1.049388000
O	0.793022000	2.731540000	0.242267000
C	1.154291000	3.405007000	1.451233000
C	2.595369000	3.846561000	1.332714000
H	1.010937000	2.717001000	2.298865000
H	0.480827000	4.263114000	1.600495000
C	3.045277000	4.584879000	2.592262000
H	3.226827000	2.960517000	1.156866000
H	2.697517000	4.496268000	0.449602000
H	2.405458000	5.484184000	2.712538000
H	2.836441000	3.964051000	3.480576000
C	4.482194000	4.996750000	2.605646000
C	5.170080000	5.413228000	1.349318000
H	4.882431000	5.333776000	3.564888000
H	5.134911000	4.584324000	0.618894000
H	4.617349000	6.250173000	0.874359000
C	7.079371000	6.645371000	2.697492000
C	7.508279000	5.214779000	2.561312000
C	6.621997000	5.835827000	1.518907000
H	6.323593000	6.896718000	3.443636000
H	7.830804000	7.417156000	2.519247000
H	7.040163000	4.467760000	3.206763000
H	8.550572000	5.016747000	2.300777000
C	7.266640000	6.193922000	0.214987000
F	8.568020000	6.484233000	0.340190000
F	6.688321000	7.265246000	-0.349586000
F	7.176581000	5.204565000	-0.687787000
C	9.824971000	2.278530000	2.173403000
C	9.983067000	2.097846000	3.551227000
C	8.863512000	1.740230000	4.300822000

C	7.599245000	1.568389000	3.721033000
C	7.463827000	1.806900000	2.338975000
C	8.583812000	2.148248000	1.546034000
H	10.696125000	2.551030000	1.574006000
H	8.982461000	1.584361000	5.375519000
C	6.418784000	1.191160000	4.601859000
H	5.686446000	0.679570000	3.959696000
C	5.754126000	2.450574000	5.170783000
H	5.429853000	3.141923000	4.377382000
H	4.870975000	2.188929000	5.773720000
H	6.461886000	2.995268000	5.816025000
C	6.787355000	0.227977000	5.730364000
H	7.409287000	0.712697000	6.497838000
H	5.873021000	-0.126482000	6.228793000
H	7.331056000	-0.649940000	5.350949000
C	8.451490000	2.352011000	0.045951000
H	7.500040000	2.880835000	-0.114930000
C	9.560963000	3.203487000	-0.564453000
H	9.677306000	4.161487000	-0.036141000
H	10.529741000	2.681093000	-0.551105000
H	9.321950000	3.424569000	-1.615166000
C	8.362105000	0.998206000	-0.669333000
H	9.301304000	0.437148000	-0.541514000
H	7.543487000	0.377779000	-0.273936000
H	8.190328000	1.139165000	-1.747136000
C	11.332999000	2.304354000	4.211480000
H	11.204103000	2.083561000	5.283052000
C	11.792074000	3.759421000	4.079237000
H	12.740830000	3.917070000	4.614413000
H	11.954889000	4.023655000	3.022204000
H	11.045393000	4.454371000	4.492170000
C	12.389043000	1.347891000	3.652653000
H	13.345653000	1.476668000	4.181239000
H	12.076243000	0.298758000	3.760059000
H	12.569020000	1.542289000	2.583736000
S	5.863878000	1.663860000	1.536239000
H	5.164082000	2.393145000	2.432381000

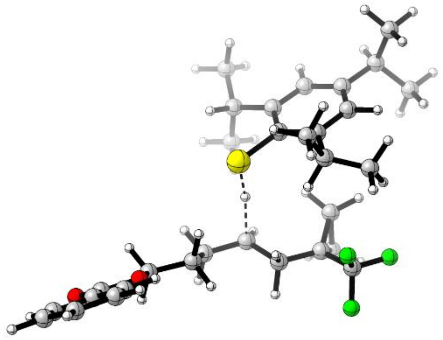

TS3cp

C	-1.345655000	0.394120000	-3.849845000
C	-0.039566000	0.819672000	-3.600818000
C	0.285321000	1.398640000	-2.375504000
C	-0.703540000	1.549970000	-1.396580000
C	-2.011650000	1.122150000	-1.646648000
C	-2.332269000	0.545473000	-2.872778000
H	-1.595929000	-0.059378000	-4.810814000
H	0.729758000	0.699524000	-4.365372000
H	1.303348000	1.733043000	-2.174349000
H	-2.767128000	1.248827000	-0.869615000
H	-3.352996000	0.212896000	-3.068531000
C	-0.411141000	2.162865000	-0.065237000
O	-1.228194000	2.291224000	0.812648000
O	0.854749000	2.558338000	0.053805000
C	1.228933000	3.146992000	1.301947000
C	2.670258000	3.590727000	1.192953000
H	1.093424000	2.404021000	2.103357000
H	0.560937000	3.995325000	1.515771000
C	3.155946000	4.214483000	2.499225000
H	3.293544000	2.720684000	0.926856000
H	2.755809000	4.314330000	0.367766000
H	2.530575000	5.100604000	2.729293000
H	2.993951000	3.512328000	3.334494000
C	4.593188000	4.644245000	2.490524000
C	5.145040000	5.317128000	1.273885000
H	4.986309000	4.963592000	3.462672000
H	5.121609000	4.612956000	0.423222000
H	4.472908000	6.150084000	0.985362000
C	6.955486000	6.692961000	2.605473000
C	7.541382000	5.326960000	2.416549000
C	6.555506000	5.866593000	1.417808000
H	6.198551000	6.829589000	3.380902000
H	7.601889000	7.555022000	2.429122000
H	7.193971000	4.522183000	3.067716000
H	8.588523000	5.254020000	2.114433000
C	7.110520000	6.326087000	0.103923000
F	8.371940000	6.763625000	0.195970000
F	6.387550000	7.332099000	-0.410475000
F	7.103844000	5.352344000	-0.818464000
C	9.725352000	2.276891000	2.261266000
C	10.050633000	2.073525000	3.606564000
C	9.024139000	1.750617000	4.492612000
C	7.689712000	1.643188000	4.079075000
C	7.385418000	1.881149000	2.720495000
C	8.412676000	2.188429000	1.793984000
H	10.525026000	2.523834000	1.560716000
H	9.275576000	1.581172000	5.541725000
C	6.608651000	1.337117000	5.104727000
H	5.824228000	0.769841000	4.583690000
C	5.977510000	2.641534000	5.603876000
H	5.553016000	3.222705000	4.772532000
H	5.168654000	2.434921000	6.321534000
H	6.732653000	3.265853000	6.108039000
C	7.095045000	0.495124000	6.283199000
H	7.768176000	1.062567000	6.943351000
H	6.234297000	0.180010000	6.891459000
H	7.624958000	-0.409056000	5.948365000
C	8.117291000	2.406017000	0.316793000
H	7.171860000	2.968275000	0.259921000
C	9.185814000	3.214892000	-0.416018000
H	9.391441000	4.174071000	0.083033000

H	10.132399000	2.658916000	-0.497323000
H	8.845841000	3.433562000	-1.438427000
C	7.909826000	1.058342000	-0.385299000
H	8.836228000	0.463487000	-0.346572000
H	7.107645000	0.476140000	0.089389000
H	7.645740000	1.209951000	-1.443067000
C	11.484800000	2.194843000	4.084949000
H	11.474157000	2.046783000	5.176480000
C	12.053363000	3.587457000	3.801373000
H	13.071457000	3.680634000	4.208898000
H	12.107303000	3.777800000	2.718044000
H	11.430480000	4.373564000	4.254013000
C	12.368457000	1.107174000	3.468336000
H	13.392443000	1.167306000	3.867354000
H	11.974333000	0.102900000	3.683513000
H	12.425268000	1.223362000	2.374524000
S	5.685291000	1.781720000	2.159739000
H	5.298675000	3.155512000	2.407247000

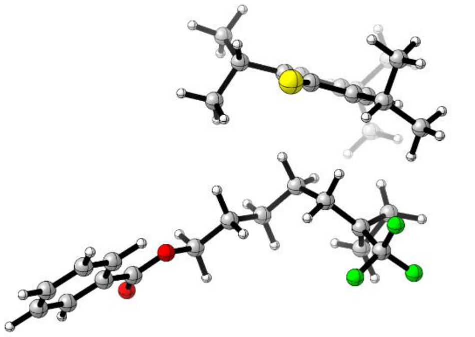

2-thiyl-prodC

C	-2.664551000	1.309779000	-1.598596000
C	-1.294620000	1.086039000	-1.748590000
C	-0.384455000	1.744259000	-0.923599000
C	-0.849913000	2.629499000	0.055227000
C	-2.222760000	2.852925000	0.204683000
C	-3.128809000	2.193103000	-0.621433000
H	-3.374699000	0.793016000	-2.246898000
H	-0.933043000	0.395208000	-2.511983000
H	0.686710000	1.575044000	-1.035628000
H	-2.565363000	3.548047000	0.972719000
H	-4.199659000	2.367678000	-0.504998000
C	0.081837000	3.366177000	0.962737000
O	-0.278982000	4.146583000	1.808788000
O	1.360086000	3.071886000	0.737856000
C	2.329718000	3.747032000	1.545462000
C	3.699882000	3.243552000	1.151026000
H	2.114427000	3.547672000	2.606639000
H	2.235056000	4.832251000	1.383447000
C	4.804420000	3.958299000	1.925366000
H	3.750584000	2.159190000	1.339534000
H	3.827301000	3.391748000	0.067063000
H	4.751201000	5.038379000	1.709997000
H	4.611176000	3.847947000	3.005351000
C	6.204964000	3.419693000	1.620877000
C	6.715959000	3.711586000	0.208836000
H	6.923431000	3.803307000	2.365395000
H	7.439004000	2.936259000	-0.091238000
H	5.878809000	3.641843000	-0.504045000
C	7.242640000	6.214746000	0.981637000
C	8.544395000	5.479683000	0.895703000
C	7.393360000	5.065147000	0.024297000
H	6.623823000	6.050165000	1.864108000
H	7.196644000	7.221850000	0.562998000
H	8.800570000	4.792582000	1.705378000
H	9.391087000	5.981846000	0.423477000
C	7.521574000	5.436086000	-1.420692000
F	8.170565000	6.591512000	-1.611882000
F	6.327162000	5.565456000	-2.017696000
F	8.189429000	4.500918000	-2.116406000
C	9.604580000	1.931796000	2.244205000
C	9.117175000	1.809537000	3.556497000
C	8.083911000	0.900097000	3.811124000
C	7.516180000	0.124102000	2.805633000
C	8.003100000	0.270847000	1.461104000
C	9.087388000	1.182435000	1.195999000
H	10.421204000	2.630594000	2.052275000
H	7.719002000	0.809012000	4.835701000
C	6.355623000	-0.799407000	3.127307000
H	6.437794000	-1.662520000	2.451374000
C	5.026157000	-0.099221000	2.814136000
H	4.970242000	0.188107000	1.754201000
H	4.179861000	-0.767558000	3.034563000
H	4.914733000	0.809074000	3.428528000
C	6.357980000	-1.314040000	4.565820000
H	6.115773000	-0.518967000	5.287161000
H	5.593434000	-2.096778000	4.676210000
H	7.330289000	-1.746421000	4.844842000
C	9.699776000	1.279338000	-0.189949000
H	8.873548000	1.194012000	-0.911441000
C	10.431220000	2.592749000	-0.454875000
H	9.800770000	3.463252000	-0.224609000
H	11.357107000	2.670382000	0.135280000
H	10.712985000	2.648847000	-1.516545000
C	10.642268000	0.090746000	-0.425362000
H	11.479182000	0.118221000	0.290213000
H	10.118172000	-0.868286000	-0.305935000
H	11.059694000	0.130775000	-1.442758000
C	9.709701000	2.630935000	4.680432000
H	9.139185000	2.388797000	5.590405000
C	9.569177000	4.131789000	4.413776000
H	9.913027000	4.706440000	5.286621000
H	10.180321000	4.437309000	3.550401000
H	8.522642000	4.407055000	4.212737000
C	11.174757000	2.254502000	4.922532000
H	11.575843000	2.819002000	5.777549000
H	11.283502000	1.181430000	5.137466000
H	11.791243000	2.492270000	4.041575000
S	7.273969000	-0.602787000	0.178616000
H	6.189311000	2.327589000	1.762063000

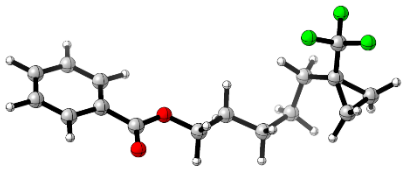

2

C	-2.451865000	0.960973000	-3.060175000
C	-1.089605000	0.701408000	-2.898588000
C	-0.360418000	1.378362000	-1.922723000
C	-0.999841000	2.317949000	-1.106646000
C	-2.364681000	2.578023000	-1.269647000
C	-3.089787000	1.899223000	-2.246045000
H	-3.019366000	0.428857000	-3.825997000
H	-0.593283000	-0.032081000	-3.536070000
H	0.703550000	1.181168000	-1.790184000
H	-2.843401000	3.315041000	-0.622903000
H	-4.154467000	2.101468000	-2.373707000
C	-0.263157000	3.068608000	-0.044192000
O	-0.772737000	3.884695000	0.683282000
O	1.025064000	2.739846000	0.016528000
C	1.819031000	3.406077000	1.003140000
C	3.234272000	2.884843000	0.893435000
H	1.390477000	3.212226000	1.998872000
H	1.773003000	4.492024000	0.826437000
C	4.150447000	3.503742000	1.945668000
H	3.221967000	1.789178000	1.009509000
H	3.610228000	3.098174000	-0.120262000
H	4.076946000	4.603189000	1.889095000
H	3.787977000	3.218071000	2.946224000
C	5.611676000	3.073021000	1.785423000
C	6.338103000	3.792759000	0.647528000
H	6.158772000	3.222283000	2.730243000
H	7.172040000	3.176524000	0.278766000
H	5.642586000	3.914776000	-0.198879000
C	6.549155000	5.906664000	2.270580000
C	7.911321000	5.304815000	2.115403000
C	6.903304000	5.162897000	1.012225000
H	5.848964000	5.426497000	2.955038000
H	6.463680000	6.993481000	2.207350000
H	8.134729000	4.393127000	2.674254000
H	8.756783000	5.977167000	1.959062000
C	7.134758000	6.018356000	-0.194733000
F	7.727976000	7.183506000	0.092822000
F	5.988112000	6.318558000	-0.824557000
F	7.910099000	5.403193000	-1.100241000
H	5.639158000	1.989073000	1.593542000

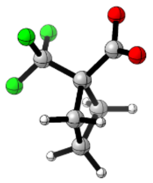

IIIcb

C	0.810310000	-0.359780000	-0.165394000
C	2.112166000	-1.119325000	0.163602000
C	1.600547000	0.977763000	-0.069656000
H	0.462985000	-0.533926000	-1.191810000
H	-0.037524000	-0.456798000	0.525426000
H	2.344826000	-1.978349000	-0.476888000
H	2.157871000	-1.425513000	1.215897000
C	1.384635000	1.682965000	1.251264000
F	0.122245000	2.098231000	1.399126000
F	2.171094000	2.756651000	1.365196000
F	1.655252000	0.878500000	2.280056000
C	1.407987000	1.926842000	-1.245795000
O	2.088385000	1.958568000	-2.237363000
O	0.360245000	2.710091000	-1.127232000
C	2.917301000	0.169005000	-0.108835000
H	3.343178000	0.200034000	-1.118661000
H	3.680021000	0.471365000	0.620564000

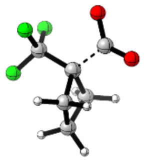

TS1cb

C	-0.862565000	-0.944440000	-0.246725000
C	0.458555000	-1.668620000	0.095148000
C	-0.106020000	0.400231000	-0.119813000
H	-1.180178000	-1.121477000	-1.281288000
H	-1.717979000	-1.069991000	0.429454000
H	0.720184000	-2.513021000	-0.553764000
H	0.498783000	-1.977513000	1.146403000
C	-0.342866000	1.084743000	1.211935000
F	-1.604681000	1.486540000	1.366566000
F	0.445958000	2.147463000	1.374382000
F	-0.078512000	0.242030000	2.216411000
C	-0.299473000	1.317585000	-1.395060000
O	0.061563000	0.958145000	-2.480896000
O	-0.892547000	2.384734000	-1.024045000
C	1.241317000	-0.361784000	-0.158571000
H	1.675037000	-0.323373000	-1.163892000
H	1.983424000	-0.041252000	0.583755000

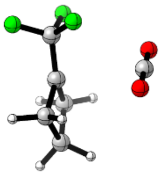

IVcb-CO_2-prodC

C	0.755151000	-0.375423000	0.061531000
C	2.053236000	-1.036561000	-0.483642000
C	1.609529000	0.789449000	0.441622000
H	-0.053601000	-0.182655000	-0.661145000
H	0.320838000	-0.899824000	0.930278000
H	2.097551000	-1.023117000	-1.580197000
H	2.261627000	-2.050456000	-0.122183000

C	1.310876000	2.051302000	1.142818000
F	-0.002733000	2.272241000	1.233319000
F	1.849025000	3.118372000	0.514458000
F	1.804253000	2.083741000	2.393477000
C	1.380526000	2.358833000	-2.152408000
O	2.505019000	2.242957000	-2.397141000
O	0.252402000	2.476650000	-1.924176000
C	2.902898000	0.098193000	0.154785000
H	3.613908000	0.626729000	-0.498831000
H	3.437014000	-0.208576000	1.070678000

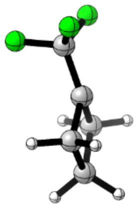

IVcb

C	0.783488000	-0.446788000	0.158671000
C	2.086900000	-1.186832000	-0.253040000
C	1.625356000	0.772086000	0.372708000
H	0.004838000	-0.363123000	-0.616260000
H	0.310878000	-0.825810000	1.080566000
H	2.118915000	-1.465242000	-1.313493000
H	2.325111000	-2.065531000	0.358306000
C	1.360338000	1.949044000	1.218328000
F	0.110503000	2.405844000	1.062562000
F	2.198485000	2.960623000	0.954436000
F	1.499638000	1.683302000	2.534481000
C	2.921290000	0.080505000	0.084931000
H	3.519736000	0.502731000	-0.738019000
H	3.574754000	-0.019037000	0.968007000

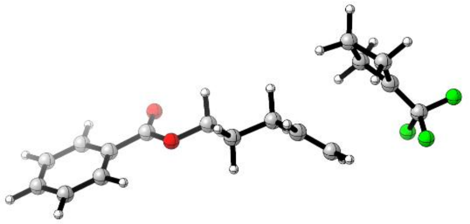

IVcb-alkene-reactC

C	-1.461595000	-1.418905000	-0.404038000
C	-0.076951000	-1.311233000	-0.545820000
C	0.551455000	-0.081411000	-0.358267000
C	-0.212144000	1.044194000	-0.028884000
C	-1.599531000	0.935258000	0.112934000
C	-2.223350000	-0.295541000	-0.074553000
H	-1.950342000	-2.383889000	-0.551369000
H	0.516366000	-2.190049000	-0.803755000
H	1.632353000	0.010616000	-0.466725000
H	-2.176714000	1.824889000	0.369955000
H	-3.305614000	-0.380680000	0.036048000
C	0.409223000	2.387426000	0.179761000
O	-0.214059000	3.383500000	0.452241000
O	1.732357000	2.372893000	0.032664000
C	2.411332000	3.619584000	0.208939000
C	3.891310000	3.383303000	0.008474000
H	2.198758000	4.005072000	1.218233000
H	2.016344000	4.347870000	-0.516213000
C	4.686903000	4.679485000	0.174201000
H	4.239242000	2.633328000	0.736754000
H	4.059434000	2.965893000	-0.996826000
H	4.345836000	5.426406000	-0.560952000
H	4.478765000	5.094381000	1.176939000
C	6.165425000	4.467310000	0.030566000
C	6.938957000	5.071627000	-0.873857000
H	6.617838000	3.751979000	0.729484000
H	8.012288000	4.873514000	-0.932269000
H	6.520409000	5.787020000	-1.589747000
C	6.175756000	7.921191000	1.136256000
C	6.774553000	7.133013000	2.336195000
C	7.527669000	7.836869000	0.509561000
H	5.360350000	7.427082000	0.582279000
H	5.853031000	8.946902000	1.383628000
H	6.396047000	6.104573000	2.402917000
H	6.673042000	7.621372000	3.312885000
C	8.054537000	8.347251000	-0.765200000
C	8.190109000	7.217409000	1.696308000
H	8.705700000	6.259987000	1.515755000
H	8.886586000	7.889673000	2.225742000
F	8.597747000	9.579127000	-0.664696000
F	7.094735000	8.442020000	-1.697237000
F	9.022389000	7.561759000	-1.262792000

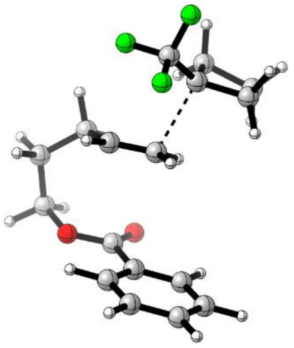

TS2cb

C	-3.523259000	-3.029983000	0.398203000
C	-3.552721000	-2.032312000	1.374035000
C	-3.223812000	-0.719533000	1.041838000
C	-2.865493000	-0.406667000	-0.273359000
C	-2.835798000	-1.406688000	-1.250304000
C	-3.165281000	-2.717105000	-0.914670000
H	-3.779009000	-4.057832000	0.662639000
H	-3.829633000	-2.279648000	2.400239000
H	-3.236678000	0.064830000	1.798813000
H	-2.548970000	-1.139664000	-2.268666000
H	-3.141304000	-3.497799000	-1.676807000
C	-2.465831000	0.975311000	-0.678489000
O	-2.105539000	1.265242000	-1.792084000

O	-2.552392000	1.845500000	0.329317000
C	-2.082676000	3.182096000	0.119828000
C	-0.696515000	3.376875000	0.707627000
H	-2.808727000	3.835895000	0.620609000
H	-2.090735000	3.400241000	-0.955937000
C	0.383147000	2.465048000	0.110793000
H	-0.426685000	4.430448000	0.536841000
H	-0.737714000	3.233303000	1.799775000
H	0.322922000	2.474274000	-0.989878000
H	1.367887000	2.888076000	0.381019000
C	0.342917000	1.053702000	0.614513000
C	0.474819000	-0.050403000	-0.159828000
H	0.295471000	0.930007000	1.703226000
H	0.375902000	-1.046049000	0.279178000
H	0.392232000	0.024410000	-1.248976000
C	2.723690000	-1.694866000	-1.360878000
C	2.893481000	-0.747866000	-2.577500000
C	2.718632000	-0.464718000	-0.488761000
H	1.822915000	-2.328211000	-1.338430000
H	3.604320000	-2.334184000	-1.179804000
H	1.960082000	-0.616394000	-3.140435000
H	3.700053000	-1.000779000	-3.276468000
C	3.229268000	-0.392380000	0.895953000
C	3.174922000	0.431551000	-1.610389000
H	2.608003000	1.362451000	-1.768396000
H	4.246523000	0.684934000	-1.537459000
F	4.513167000	-0.805222000	0.988641000
F	2.537077000	-1.159595000	1.749998000
F	3.208963000	0.859478000	1.374645000

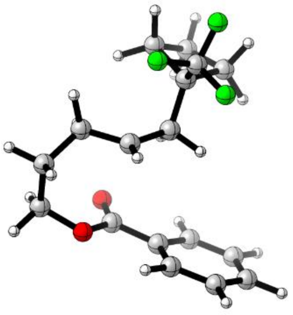

Vcb

C	-3.510431000	-2.947394000	0.531466000
C	-3.549302000	-1.929120000	1.486129000
C	-3.277059000	-0.613302000	1.115743000
C	-2.963755000	-0.318866000	-0.215648000
C	-2.924555000	-1.339421000	-1.171293000
C	-3.199402000	-2.652555000	-0.797882000
H	-3.722671000	-3.977214000	0.825000000
H	-3.789726000	-2.162196000	2.524817000
H	-3.296908000	0.187733000	1.855262000
H	-2.672078000	-1.088577000	-2.202932000
H	-3.168402000	-3.449386000	-1.542858000
C	-2.607277000	1.064414000	-0.653660000
O	-2.210510000	1.327733000	-1.763233000
O	-2.771819000	1.966301000	0.314057000
C	-2.240121000	3.282017000	0.110848000
C	-0.846779000	3.406081000	0.704271000
H	-2.938018000	3.963730000	0.613247000
H	-2.235696000	3.505289000	-0.964044000
C	0.184438000	2.439134000	0.106855000
H	-0.524093000	4.445347000	0.538211000
H	-0.900501000	3.258773000	1.795355000
H	0.122076000	2.464325000	-0.994670000
H	1.195286000	2.814889000	0.363692000
C	0.046872000	1.031287000	0.590232000
C	0.432768000	-0.150897000	-0.235880000
H	-0.120374000	0.880222000	1.661315000
H	0.001068000	-1.066783000	0.199975000
H	0.009948000	-0.047656000	-1.251726000
C	2.205369000	-1.588832000	-1.382414000
C	2.452852000	-0.569969000	-2.515151000
C	1.943703000	-0.402669000	-0.422432000
H	1.370590000	-2.293909000	-1.499486000
H	3.116414000	-2.142243000	-1.118032000
H	1.554710000	-0.407349000	-3.126014000
H	3.305230000	-0.762225000	-3.179006000
C	2.646086000	-0.476530000	0.909764000
C	2.632864000	0.530766000	-1.448541000
H	2.153309000	1.502374000	-1.625128000
H	3.692154000	0.689736000	-1.201129000
F	3.913017000	-0.900816000	0.796922000
F	2.037077000	-1.311075000	1.763608000
F	2.707502000	0.720886000	1.513309000

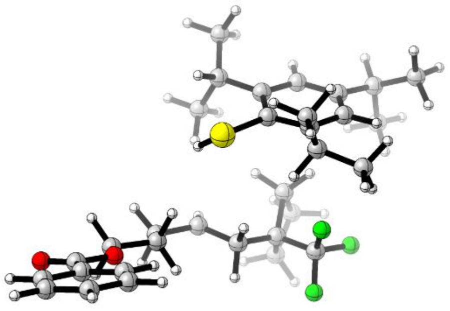

Vcb-thiol-reactC

C	-0.818599000	0.490978000	-3.952949000
C	0.462934000	0.927449000	-3.611688000
C	0.693775000	1.513873000	-2.368725000
C	-0.364553000	1.661629000	-1.465123000
C	-1.648313000	1.224076000	-1.808088000
C	-1.874789000	0.639039000	-3.051201000
H	-0.994570000	0.031722000	-4.927623000
H	1.285740000	0.810331000	-4.318791000
H	1.691673000	1.856317000	-2.094011000
H	-2.458929000	1.350181000	-1.088772000
H	-2.876205000	0.298749000	-3.319763000
C	-0.173967000	2.285394000	-0.120345000
O	-1.056529000	2.425221000	0.689986000
O	1.080654000	2.675809000	0.093756000
C	1.355793000	3.294692000	1.353545000

C	2.820987000	3.665068000	1.385777000
H	1.097942000	2.593544000	2.162255000
H	0.713364000	4.182196000	1.464641000
C	3.178619000	4.381581000	2.686824000
H	3.427628000	2.752757000	1.269269000
H	3.043307000	4.310855000	0.522028000
H	2.577410000	5.312773000	2.745699000
H	2.849758000	3.774882000	3.548910000
C	4.628095000	4.714939000	2.840716000
C	5.495325000	4.966397000	1.652417000
H	4.950083000	5.082391000	3.818514000
H	5.582400000	4.036644000	1.057608000
H	5.015773000	5.702269000	0.975532000
C	9.630990000	1.706035000	1.878476000
C	9.987400000	1.825045000	3.224502000
C	9.006489000	1.586163000	4.188038000
C	7.682242000	1.286466000	3.849636000
C	7.334407000	1.276837000	2.482488000
C	8.319281000	1.430325000	1.479638000
H	10.394515000	1.848408000	1.113397000
H	9.287465000	1.637452000	5.243152000
C	6.673303000	0.998295000	4.952928000
H	5.887851000	0.367027000	4.510604000
C	6.010418000	2.281663000	5.466085000
H	5.489089000	2.831361000	4.667254000
H	5.273621000	2.046638000	6.248973000
H	6.765512000	2.957207000	5.899295000
C	7.277879000	0.215920000	6.119885000
H	7.975673000	0.830270000	6.708345000
H	6.477340000	-0.109815000	6.800377000
H	7.816132000	-0.677978000	5.771880000
C	7.979542000	1.243717000	0.007764000
H	7.051946000	1.808995000	-0.181473000
C	9.051233000	1.755487000	-0.952121000
H	9.325784000	2.798251000	-0.746382000
H	9.963102000	1.140756000	-0.899828000
H	8.676667000	1.698124000	-1.984649000
C	7.702160000	-0.237860000	-0.287948000
H	8.616637000	-0.830120000	-0.126441000
H	6.915379000	-0.658019000	0.355330000
H	7.388463000	-0.370545000	-1.334308000
C	11.369708000	2.281940000	3.651976000
H	11.548727000	1.864559000	4.656335000
C	11.382600000	3.811501000	3.773517000
H	12.366420000	4.169226000	4.114564000
H	11.170152000	4.275511000	2.796132000
H	10.623010000	4.161342000	4.488959000
C	12.489354000	1.805816000	2.729174000
H	13.468730000	2.062340000	3.159138000
H	12.456553000	0.716415000	2.578963000
H	12.428486000	2.289018000	1.742069000
S	5.632237000	1.024906000	1.970864000
H	5.062239000	1.814839000	2.904852000
C	7.022176000	6.790795000	2.801013000
C	8.053926000	6.113878000	3.731512000
C	6.897162000	5.473118000	1.992930000
H	6.078491000	7.032964000	3.306908000
H	7.354927000	7.672888000	2.235637000
H	7.865986000	6.209354000	4.808992000
H	9.084997000	6.426043000	3.520059000
C	7.710762000	5.552338000	0.726603000
C	7.702678000	4.747532000	3.102075000
H	7.043807000	4.138911000	3.732245000
H	8.535694000	4.114954000	2.761593000
F	8.967509000	5.968412000	0.945591000
F	7.180419000	6.397418000	-0.168702000
F	7.809202000	4.360305000	0.117404000

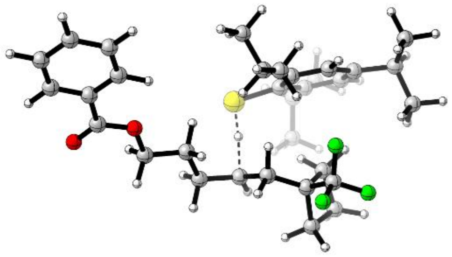

TS3cb

C	0.329655000	-0.027467000	-4.488019000
C	1.522696000	0.484216000	-3.973785000
C	1.510745000	1.246435000	-2.807034000
C	0.297961000	1.497627000	-2.155407000
C	-0.896825000	0.984347000	-2.671259000
C	-0.880226000	0.222036000	-3.836483000
H	0.342396000	-0.624650000	-5.401719000
H	2.466935000	0.288591000	-4.484627000
H	2.438204000	1.649693000	-2.399171000
H	-1.831135000	1.192428000	-2.147510000
H	-1.811988000	-0.178749000	-4.239095000
C	0.227332000	2.316571000	-0.907324000
O	-0.797738000	2.578162000	-0.328262000
O	1.423751000	2.732427000	-0.497744000
C	1.461493000	3.536929000	0.684141000
C	2.912894000	3.755976000	1.046186000
H	0.919762000	3.019581000	1.490204000
H	0.941704000	4.487975000	0.487384000
C	3.052275000	4.615585000	2.300095000
H	3.384977000	2.774693000	1.214740000
H	3.427281000	4.229348000	0.194991000
H	2.577802000	5.601280000	2.117928000
H	2.487329000	4.160099000	3.130615000
C	4.470468000	4.855104000	2.732644000
C	5.482737000	5.263488000	1.700293000
H	4.583775000	5.282579000	3.736428000
H	5.802326000	4.383857000	1.110769000
H	4.992588000	5.939036000	0.972381000
C	8.650467000	2.076359000	1.855625000
C	9.523724000	2.188131000	2.940415000
C	8.991216000	2.101383000	4.229189000
C	7.618011000	1.974780000	4.462034000
C	6.752814000	1.934474000	3.346084000

C	7.270082000	1.927447000	2.029682000
H	9.052226000	2.107598000	0.842405000
H	9.676911000	2.152909000	5.078361000
C	7.092681000	1.924687000	5.891792000
H	6.193295000	1.291774000	5.880675000
C	6.666242000	3.315977000	6.373140000
H	5.864369000	3.729040000	5.744024000
H	6.293001000	3.264221000	7.407502000
H	7.520939000	4.011337000	6.352979000
C	8.082424000	1.305870000	6.878703000
H	8.944167000	1.965063000	7.062891000
H	7.586113000	1.142845000	7.846693000
H	8.461904000	0.336652000	6.522112000
C	6.367122000	1.690497000	0.826441000
H	5.438754000	2.252189000	0.999399000
C	6.947979000	2.157862000	-0.505524000
H	7.269659000	3.207973000	-0.467928000
H	7.811077000	1.546823000	-0.811879000
H	6.184309000	2.061755000	-1.291925000
C	5.997298000	0.204041000	0.747056000
H	6.899849000	-0.404444000	0.577659000
H	5.517814000	-0.138438000	1.674998000
H	5.300963000	0.023332000	-0.086438000
C	11.003938000	2.466898000	2.756336000
H	11.522040000	2.022779000	3.622000000
C	11.247889000	3.981089000	2.794884000
H	12.320166000	4.206632000	2.688543000
H	10.709830000	4.478828000	1.971726000
H	10.898585000	4.417551000	3.742750000
C	11.592961000	1.859071000	1.485139000
H	12.685089000	1.989244000	1.476108000
H	11.375745000	0.783040000	1.410930000
H	11.197314000	2.351326000	0.583560000
S	4.978250000	1.933091000	3.606619000
H	4.801317000	3.321481000	3.207701000
C	6.506804000	7.259417000	3.096702000
C	7.478505000	6.713306000	4.166914000
C	6.718208000	5.970612000	2.258232000
H	5.471060000	7.336982000	3.452064000
H	6.780115000	8.202455000	2.602797000
H	7.112411000	6.731178000	5.201944000
H	8.465719000	7.191180000	4.128394000
C	7.690539000	6.234306000	1.135281000
C	7.450285000	5.337024000	3.467403000
H	6.803820000	4.614505000	3.977695000
H	8.413320000	4.849128000	3.253921000
F	8.787273000	6.885118000	1.551253000
F	7.151846000	6.977961000	0.160117000
F	8.124744000	5.100748000	0.561046000

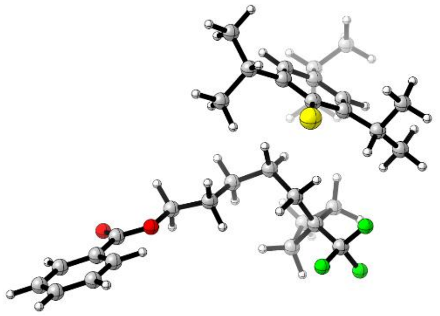

34-thiyl-prodC

C	-2.796422000	1.927591000	-1.340340000
C	-1.448919000	1.695140000	-1.622659000
C	-0.461370000	2.180600000	-0.767345000
C	-0.827469000	2.901791000	0.374841000
C	-2.177936000	3.133765000	0.656922000
C	-3.161406000	2.647117000	-0.200234000
H	-3.566174000	1.545090000	-2.013247000
H	-1.166871000	1.131093000	-2.513257000
H	0.593041000	2.002601000	-0.980442000
H	-2.441016000	3.699393000	1.552113000
H	-4.214855000	2.828550000	0.019099000
C	0.187513000	3.445928000	1.327740000
O	-0.089574000	4.063162000	2.326554000
O	1.438141000	3.178107000	0.959835000
C	2.477410000	3.659284000	1.818189000
C	3.808053000	3.273210000	1.213776000
H	2.344095000	3.220800000	2.819448000
H	2.382203000	4.751861000	1.918108000
C	4.970872000	3.761122000	2.072556000
H	3.852995000	2.176201000	1.108186000
H	3.869217000	3.699319000	0.199722000
H	4.948510000	4.860657000	2.126351000
H	4.820653000	3.408479000	3.107200000
C	6.337741000	3.247489000	1.608483000
C	6.732538000	3.458385000	0.144393000
H	7.123724000	3.650138000	2.268451000
H	7.562713000	2.765541000	-0.072187000
H	5.901819000	3.158540000	-0.515258000
C	9.711242000	1.916926000	2.395950000
C	9.085943000	1.704149000	3.637012000
C	8.082162000	0.733925000	3.730290000
C	7.663750000	-0.003383000	2.626129000
C	8.278381000	0.248358000	1.351773000
C	9.349254000	1.208817000	1.258967000
H	10.506272000	2.662467000	2.333767000
H	7.612317000	0.566073000	4.701482000
C	6.519870000	-0.989980000	2.768250000
H	6.696553000	-1.790632000	2.036132000
C	5.191763000	-0.315673000	2.396713000
H	5.217194000	0.072897000	1.368088000
H	4.364078000	-1.037317000	2.471828000
H	4.977823000	0.520958000	3.081599000
C	6.424192000	-1.620061000	4.156771000
H	6.092120000	-0.894092000	4.914164000

H	5.684387000	-2.433502000	4.139581000
H	7.387144000	-2.040445000	4.482594000
C	10.107882000	1.404121000	-0.041407000
H	9.367458000	1.361006000	-0.854537000
C	10.846042000	2.736146000	-0.136698000
H	10.177189000	3.586176000	0.058989000
H	11.690482000	2.787602000	0.567632000
H	11.255880000	2.856749000	-1.150052000
C	11.088064000	0.240448000	-0.247145000
H	11.838649000	0.226031000	0.558641000
H	10.567435000	-0.727782000	-0.252257000
H	11.616912000	0.352457000	-1.205676000
C	9.494918000	2.516223000	4.846279000
H	8.880032000	2.172586000	5.692578000
C	9.212057000	4.005761000	4.625036000
H	9.472596000	4.581851000	5.525608000
H	9.811747000	4.399452000	3.789343000
H	8.149155000	4.184245000	4.402447000
C	10.967104000	2.283994000	5.198441000
H	11.233614000	2.842187000	6.108320000
H	11.172022000	1.218276000	5.376957000
H	11.626640000	2.628284000	4.386865000
S	7.701755000	-0.541340000	-0.056655000
H	6.345292000	2.159138000	1.774392000
C	6.256171000	6.041575000	0.050692000
C	7.166514000	6.418841000	1.239163000
C	7.205840000	4.855842000	-0.266688000
H	5.209508000	5.782979000	0.256838000
H	6.283071000	6.802476000	-0.741108000
H	6.777511000	6.057593000	2.201168000
H	7.424143000	7.481071000	1.338180000
C	7.619832000	4.811832000	-1.717767000
C	8.263838000	5.493070000	0.671153000
H	8.751488000	4.801950000	1.373810000
H	9.035642000	6.042486000	0.115113000
F	8.086212000	5.989921000	-2.155605000
F	6.594412000	4.487959000	-2.519971000
F	8.589755000	3.913119000	-1.948569000

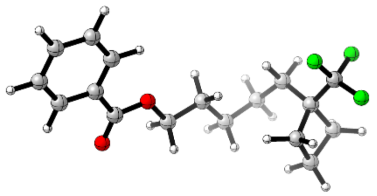

34

C	-2.515971000	1.948429000	-1.773308000
C	-1.148752000	1.882968000	-2.048211000
C	-0.222454000	2.280990000	-1.086142000
C	-0.669243000	2.747445000	0.155394000
C	-2.039452000	2.813444000	0.429477000
C	-2.961643000	2.413324000	-0.533930000
H	-3.238085000	1.633649000	-2.529054000
H	-0.802231000	1.520055000	-3.017293000
H	0.846728000	2.232972000	-1.293847000
H	-2.367049000	3.179703000	1.403702000
H	-4.030475000	2.462738000	-0.319669000
C	0.279067000	3.191015000	1.222333000
O	-0.067168000	3.621319000	2.294771000
O	1.554593000	3.061508000	0.865367000
C	2.536884000	3.469235000	1.822766000
C	3.904319000	3.183551000	1.244545000
H	2.369448000	2.921809000	2.762996000
H	2.405452000	4.542233000	2.034195000
C	5.015165000	3.644284000	2.183673000
H	3.995819000	2.101396000	1.052110000
H	3.984697000	3.692763000	0.270828000
H	4.962607000	4.737963000	2.300386000
H	4.827644000	3.228313000	3.187478000
C	6.415892000	3.194089000	1.752148000
C	6.838659000	3.464998000	0.304089000
H	7.163641000	3.610639000	2.445893000
H	7.711467000	2.827420000	0.091209000
H	6.042141000	3.134245000	-0.381542000
H	6.462851000	2.101530000	1.887551000
C	6.243300000	6.031178000	0.274200000
C	7.132717000	6.406736000	1.479066000
C	7.242060000	4.895325000	-0.071989000
H	5.204862000	5.730273000	0.462512000
H	6.249329000	6.816239000	-0.494135000
H	6.758965000	5.988326000	2.423702000
H	7.337187000	7.475584000	1.622463000
C	7.663830000	4.903221000	-1.522075000
C	8.272968000	5.558817000	0.875951000
H	8.813656000	4.877725000	1.547510000
H	9.000323000	6.171211000	0.326001000
F	8.054204000	6.116695000	-1.938407000
F	6.667298000	4.525925000	-2.337097000
F	8.692768000	4.076013000	-1.756823000

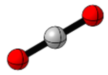

CO_2

C	-0.104872000	-0.281161000	0.242050000
O	-0.313352000	0.028985000	1.336590000
O	0.103399000	-0.591291000	-0.852608000

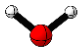

H_2O

H	-0.107980000	-0.423988000	-1.528490000
O	-0.107980000	0.344562000	-0.944018000
H	-0.107980000	-0.055409000	-0.065232000

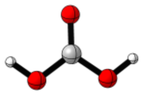

H_2CO_3

H	-0.086793000	-0.391251000	-1.607444000
O	-0.105937000	0.251178000	-0.878287000

O	-0.123981000	-1.622605000	0.360818000
C	-0.125635000	-0.425557000	0.261687000
O	-0.146950000	0.429377000	1.274979000
H	-0.165759000	-0.081899000	2.101789000

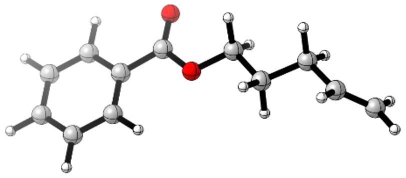

modelalkene

C	-1.382649000	-1.452915000	-0.403768000
C	0.004896000	-1.325368000	-0.489563000
C	0.603778000	-0.078157000	-0.320613000
C	-0.192041000	1.043923000	-0.064084000
C	-1.582203000	0.914912000	0.022410000
C	-2.176642000	-0.332791000	-0.148083000
H	-1.848442000	-2.431257000	-0.536894000
H	0.623115000	-2.201895000	-0.690470000
H	1.686633000	0.030400000	-0.385940000
H	-2.184194000	1.802272000	0.224342000
H	-3.261122000	-0.433910000	-0.081819000
C	0.397375000	2.404382000	0.125549000
O	-0.251454000	3.393825000	0.359744000
O	1.723579000	2.412618000	0.009887000
C	2.376943000	3.674126000	0.179108000
C	3.866217000	3.451673000	0.042470000
H	2.120263000	4.081879000	1.169174000
H	2.000065000	4.377636000	-0.579495000
C	4.646134000	4.756158000	0.217308000
H	4.189458000	2.717753000	0.798086000
H	4.078592000	3.017219000	-0.947217000
H	4.333233000	5.487636000	-0.544825000
H	4.393956000	5.186699000	1.202095000
C	6.129538000	4.543800000	0.139344000
C	6.940373000	5.121531000	-0.747563000
H	6.551135000	3.844903000	0.872972000
H	8.014472000	4.919953000	-0.753867000
H	6.555276000	5.821480000	-1.496318000

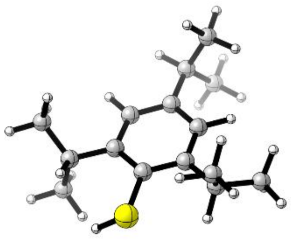

TRIPthiol

C	1.428703000	-0.467037000	-0.054907000
C	1.456288000	0.926382000	0.056917000
C	0.239463000	1.602001000	0.137290000
C	-0.991747000	0.934529000	0.112082000
C	-0.986091000	-0.469387000	-0.025774000
C	0.231705000	-1.185692000	-0.098741000
H	2.373401000	-1.010731000	-0.117421000
H	0.251791000	2.690197000	0.230365000
C	-2.282687000	1.734913000	0.182744000
H	-3.035228000	1.095262000	0.668535000
C	-2.777452000	2.072512000	-1.230289000
H	-2.875744000	1.177990000	-1.864379000
H	-3.755593000	2.575313000	-1.191975000
H	-2.062732000	2.748948000	-1.724929000
C	-2.171950000	3.009231000	1.019062000
H	-1.543852000	3.767940000	0.529003000
H	-3.170541000	3.450574000	1.153543000
H	-1.751422000	2.806105000	2.015060000
C	0.249275000	-2.705156000	-0.180554000
H	-0.577127000	-2.998211000	-0.847403000
C	1.537764000	-3.280982000	-0.763621000
H	1.792290000	-2.816433000	-1.727583000
H	2.390375000	-3.145405000	-0.081121000
H	1.418045000	-4.361865000	-0.927197000
C	-0.025160000	-3.316915000	1.200348000
H	0.786873000	-3.056906000	1.897654000
H	-0.969199000	-2.954083000	1.633886000
H	-0.082503000	-4.413966000	1.134537000
C	2.773878000	1.679227000	0.074524000
H	2.535695000	2.743425000	0.231458000
C	3.496402000	1.548701000	-1.269094000
H	4.428030000	2.134591000	-1.269790000
H	3.758061000	0.497624000	-1.469792000
H	2.866192000	1.905332000	-2.097291000
C	3.674165000	1.220706000	1.224054000
H	4.598036000	1.818179000	1.252902000
H	3.168778000	1.325615000	2.195511000
H	3.963100000	0.165083000	1.102344000
S	-2.506103000	-1.419999000	-0.056400000
H	-3.272588000	-0.522516000	-0.702811000

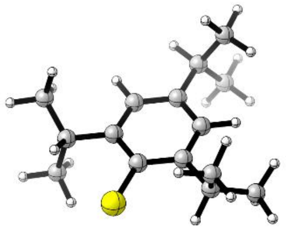

TRIPthiyl-rad

C	1.417280000	-0.476388000	-0.041586000
C	1.447084000	0.920014000	0.099494000
C	0.234795000	1.615021000	0.190175000
C	-0.994502000	0.965009000	0.153686000
C	-1.017444000	-0.466163000	0.015542000
C	0.225214000	-1.186156000	-0.087789000
H	2.363365000	-1.015582000	-0.116036000
H	0.269557000	2.700776000	0.294027000
C	-2.281195000	1.768832000	0.198797000
H	-3.014952000	1.164094000	0.750594000
C	-2.824768000	1.966256000	-1.222865000

H	-2.992058000	1.002443000	-1.724887000
H	-3.780614000	2.511136000	-1.194699000
H	-2.114968000	2.552568000	-1.827538000
C	-2.145253000	3.116180000	0.905562000
H	-1.537660000	3.826124000	0.323961000
H	-3.140845000	3.566383000	1.031100000
H	-1.691826000	3.011194000	1.902472000
C	0.246982000	-2.700633000	-0.191293000
H	-0.632337000	-2.990948000	-0.783796000
C	1.491864000	-3.253010000	-0.883535000
H	1.667249000	-2.767640000	-1.855209000
H	2.395960000	-3.126923000	-0.268758000
H	1.365791000	-4.331189000	-1.060016000
C	0.088901000	-3.324723000	1.201669000
H	0.938777000	-3.048568000	1.845846000
H	-0.837153000	-2.985830000	1.687964000
H	0.057862000	-4.422389000	1.128443000
C	2.766824000	1.659569000	0.135417000
H	2.539037000	2.723369000	0.305098000
C	3.493177000	1.535975000	-1.207807000
H	4.426456000	2.118402000	-1.193438000
H	3.751838000	0.486339000	-1.417598000
H	2.869642000	1.906323000	-2.034709000
C	3.653212000	1.171731000	1.284665000
H	4.578667000	1.765269000	1.328051000
H	3.140832000	1.264236000	2.253450000
H	3.937062000	0.117260000	1.145531000
S	-2.511266000	-1.309206000	-0.0223050

9. NMR spectra

^{1}H NMR of **1**

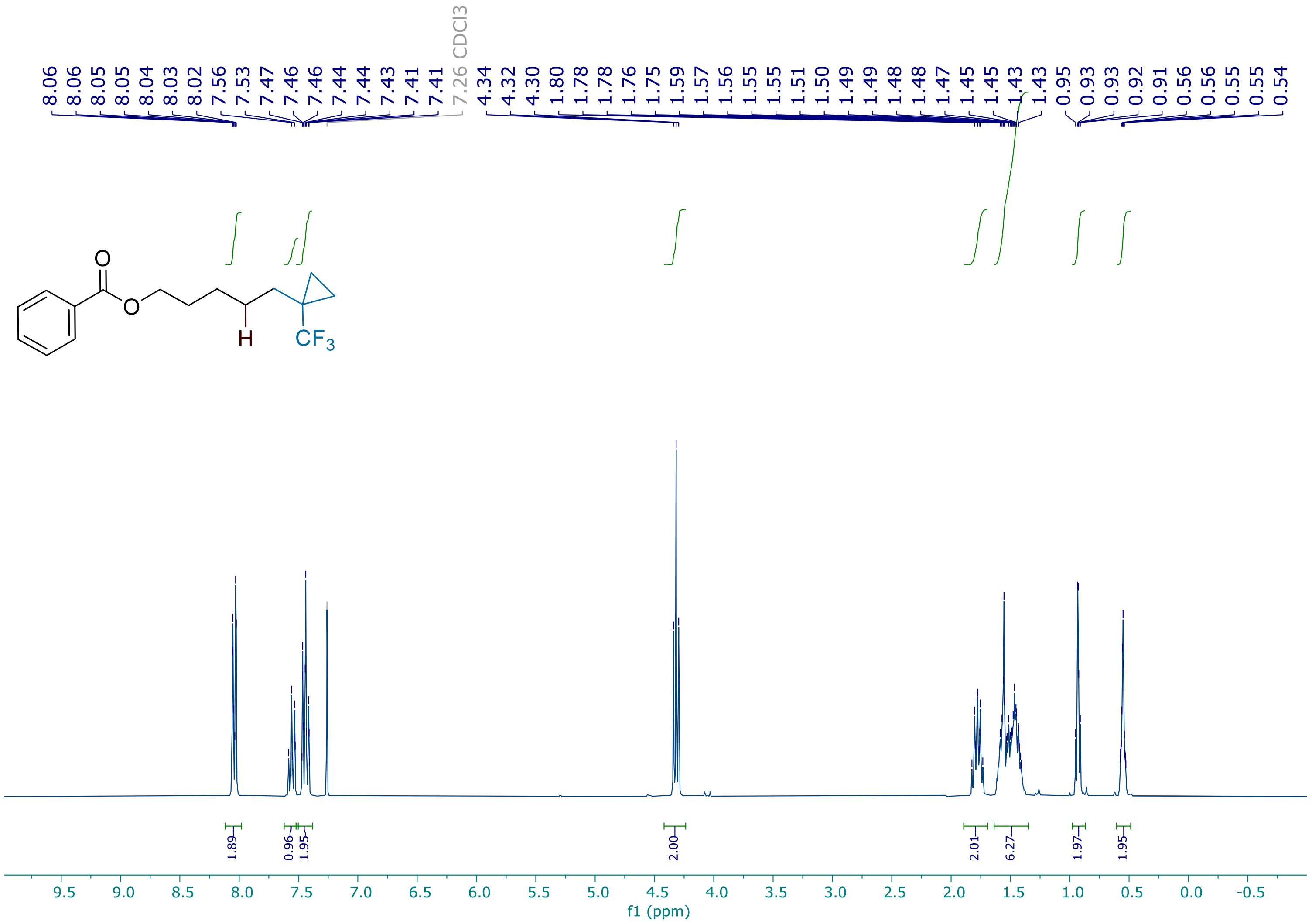

^{13}C NMR of **1**

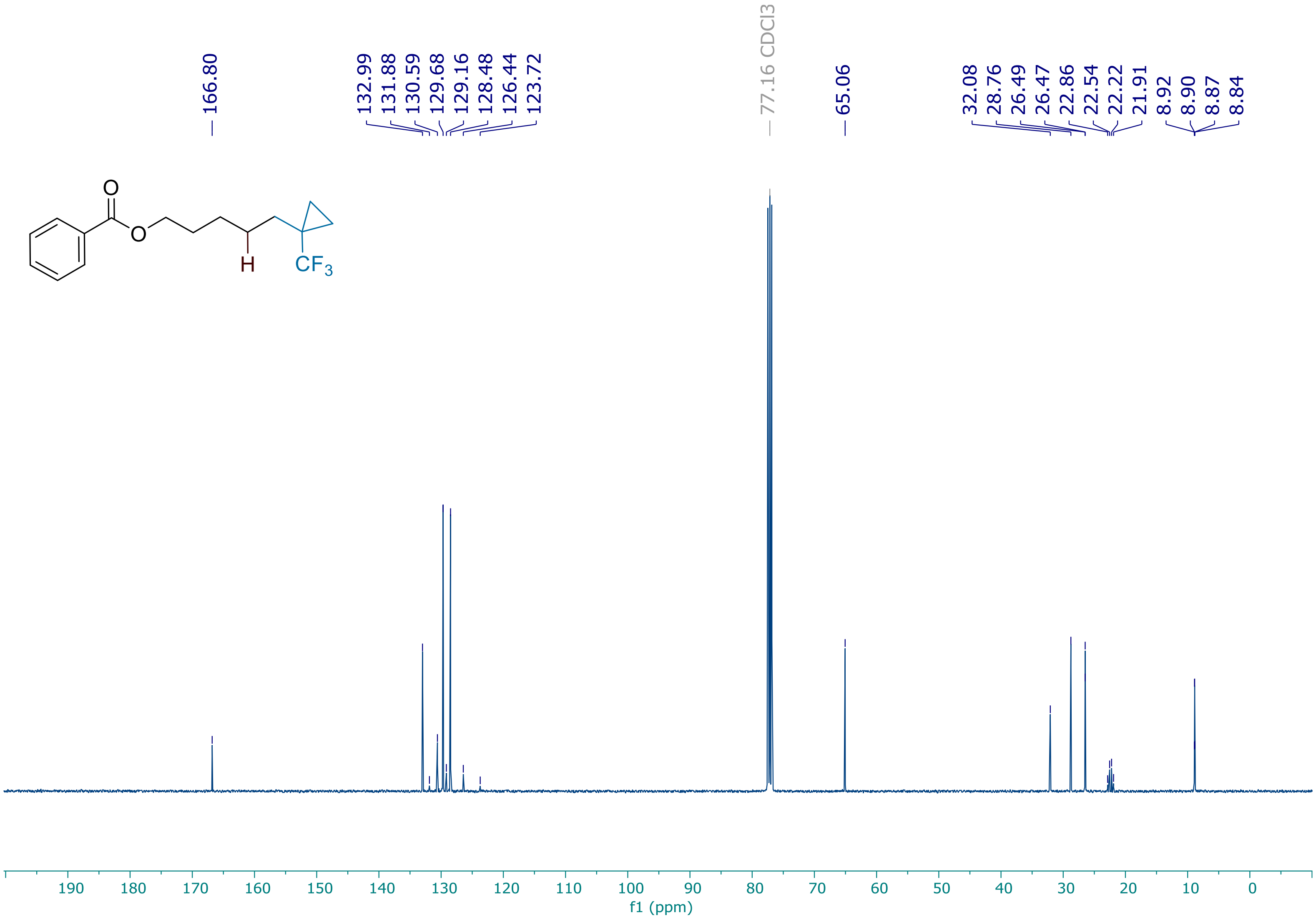
166.80
132.99
131.88
130.59
129.68
129.16
128.48
126.44
123.72
77.16 CDCl3
65.06
32.08
28.76
26.49
26.47
22.86
22.54
22.22
21.91
8.92
8.90
8.87
8.84
O
O
H
CF3
190 180 170 160 150 140 130 120 110 100 90 80 70 60 50 40 30 20 10 0
f1 (ppm)

^{19}F NMR of **1**

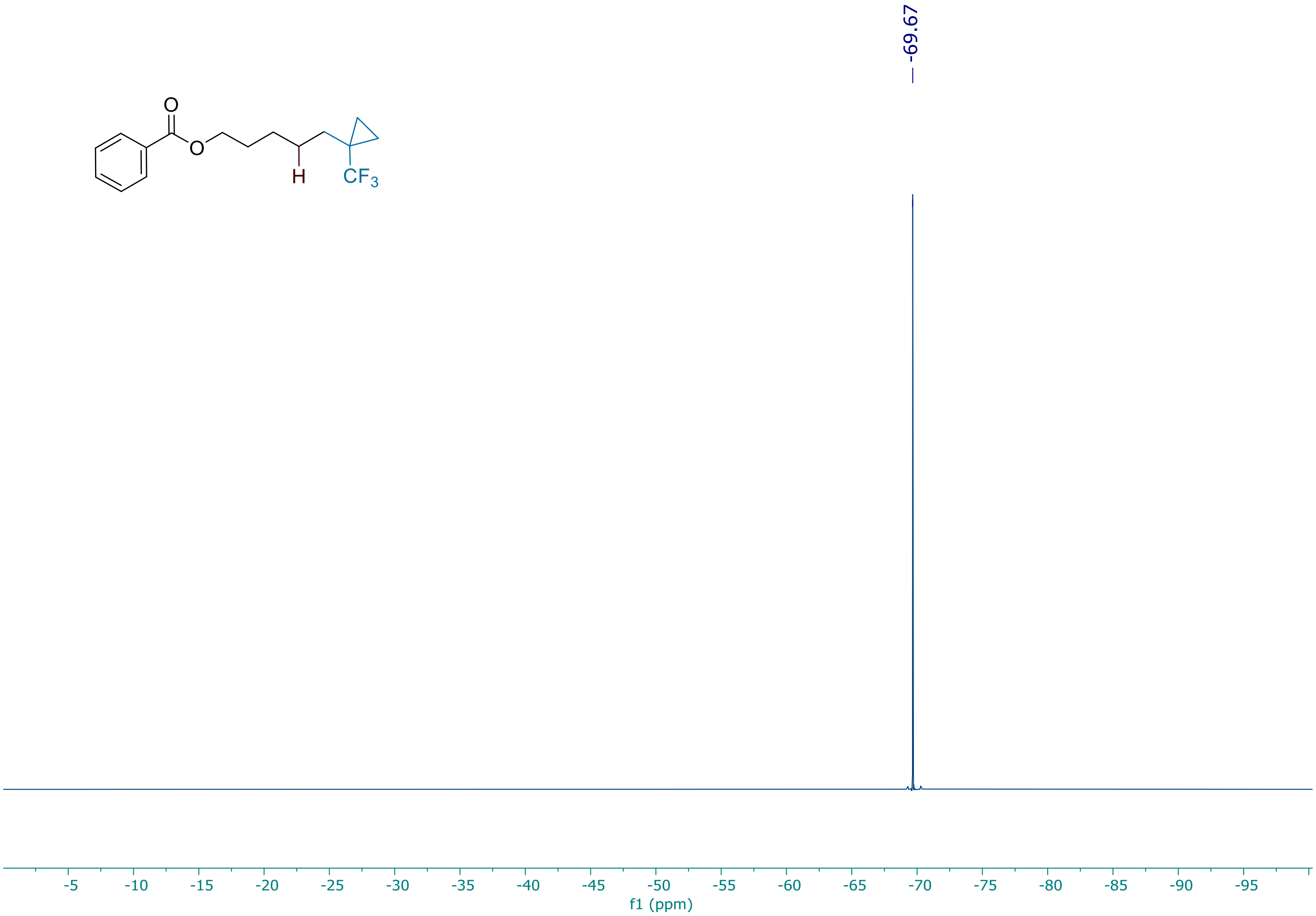

O
O
H
CF3
-69.67
-5
-10
-15
-20
-25
-30
-35
-40
-45
-50
-55
-60
-65
-70
-75
-80
-85
-90
-95
f1 (ppm)

^{1}H NMR of **2**

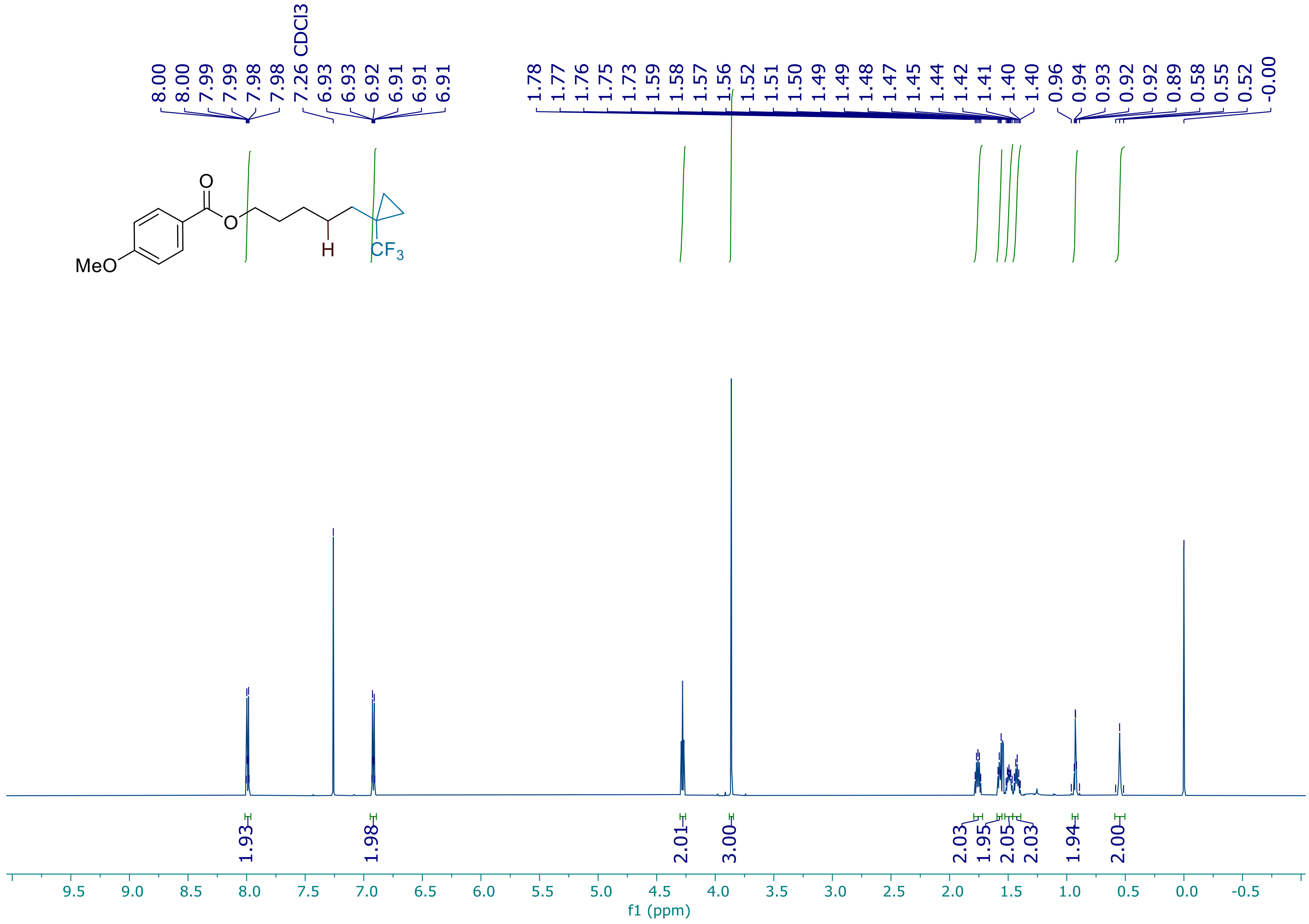
8.00
8.00
7.99
7.99
7.98
7.98
7.26 CDCl3
6.93
6.93
6.92
6.91
6.91
6.91
1.78
1.77
1.76
1.75
1.73
1.59
1.58
1.57
1.56
1.52
1.51
1.50
1.49
1.49
1.48
1.47
1.45
1.44
1.42
1.41
1.40
1.40
0.96
0.94
0.93
0.92
0.92
0.89
0.58
0.55
0.52
-0.00
MeO
O
O
H
CF3
1.93
1.98
2.01
3.00
2.03
1.95
2.05
2.03
1.94
2.00
9.5
9.0
8.5
8.0
7.5
7.0
6.5
6.0
5.5
5.0
4.5
4.0
3.5
3.0
2.5
2.0
1.5
1.0
0.5
0.0
-0.5
f1 (ppm)

^{13}C NMR of **2**

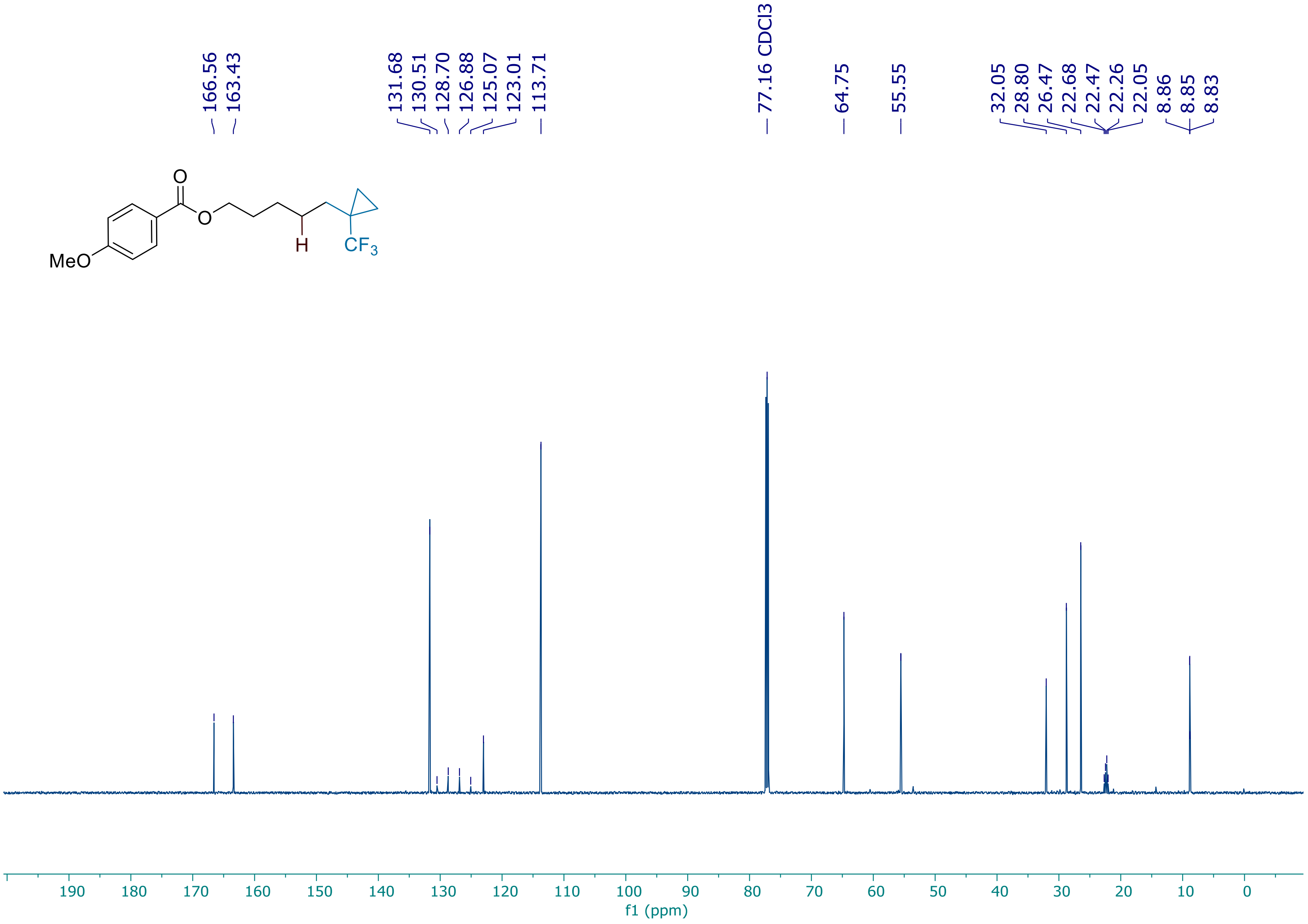

166.56
163.43
131.68
130.51
128.70
126.88
125.07
123.01
113.71
77.16 CDCl3
64.75
55.55
32.05
28.80
26.47
22.68
22.47
22.26
22.05
8.86
8.85
8.83
MeO
O
O
H
CF3
190
180
170
160
150
140
130
120
110
100
90
80
70
60
50
40
30
20
10
0
f1 (ppm)

^{19}F NMR of **2**

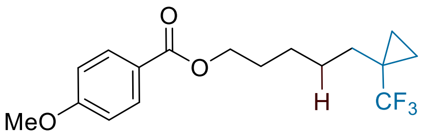
MeO
O
O
H
CF3

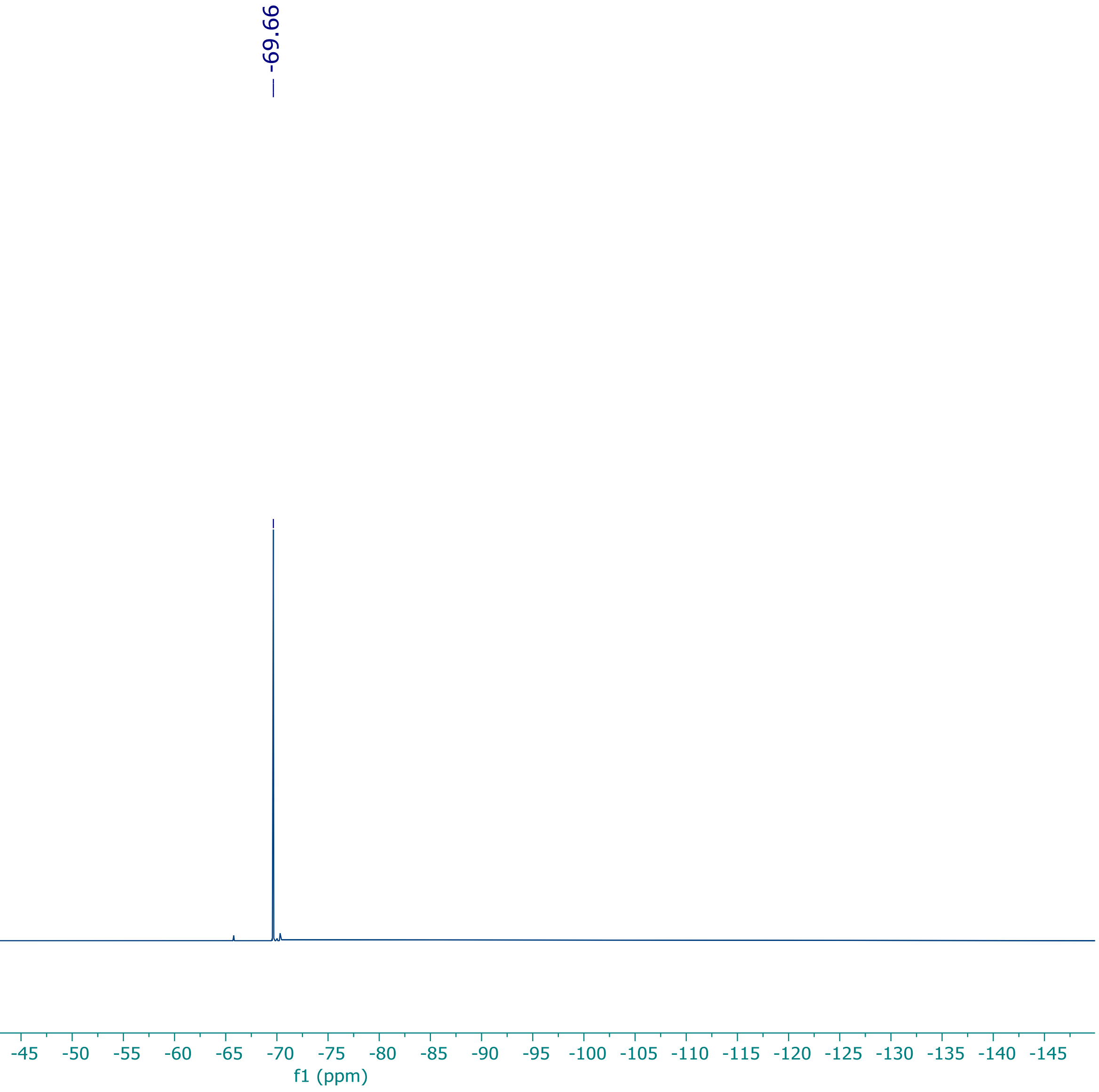
-69.66
-5 -10 -15 -20 -25 -30 -35 -40 -45 -50 -55 -60 -65 -70 -75 -80 -85 -90 -95 -100 -105 -110 -115 -120 -125 -130 -135 -140 -145
f1 (ppm)

^{1}H NMR of **3**

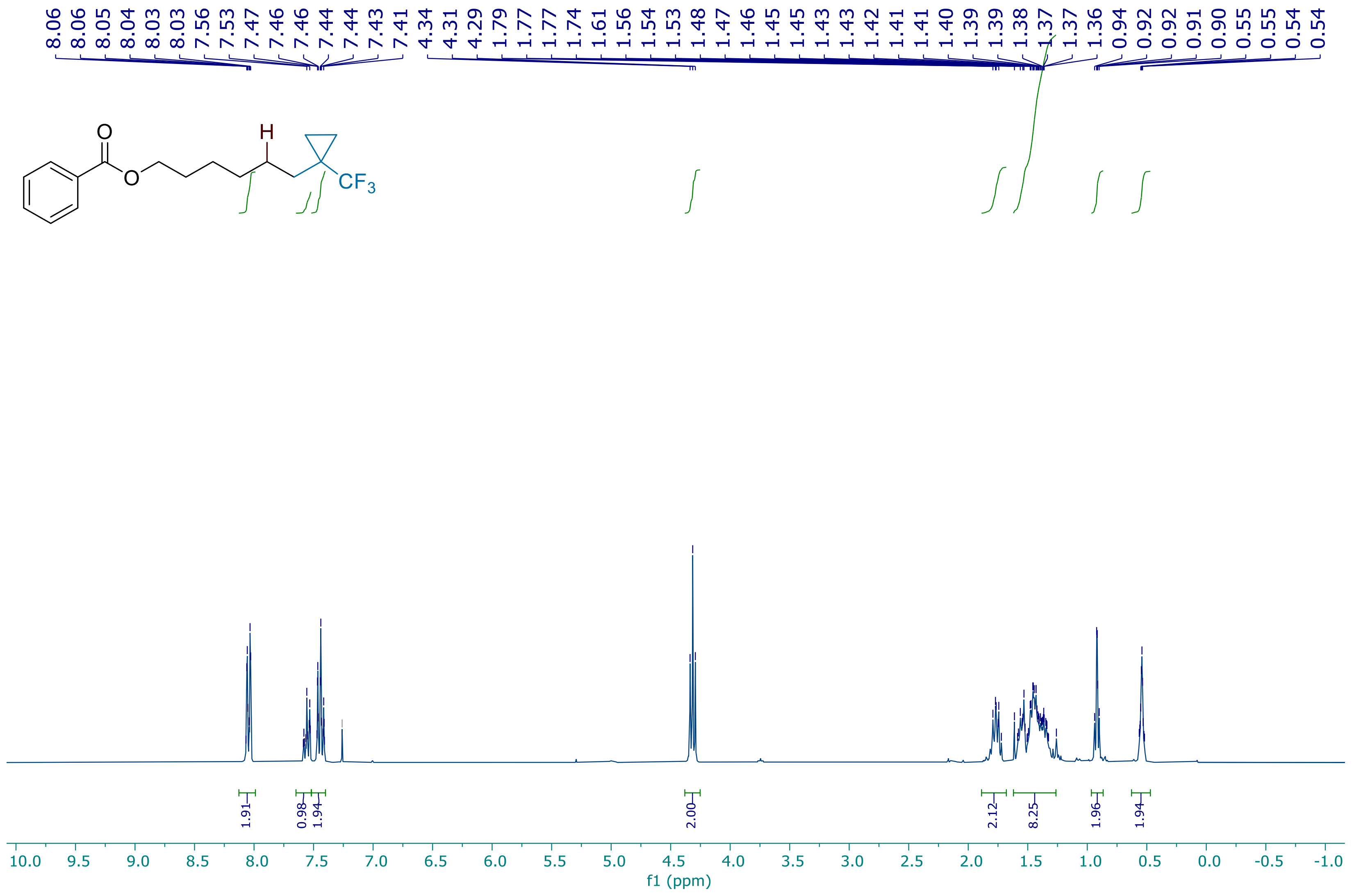

^{13}C NMR of **3**

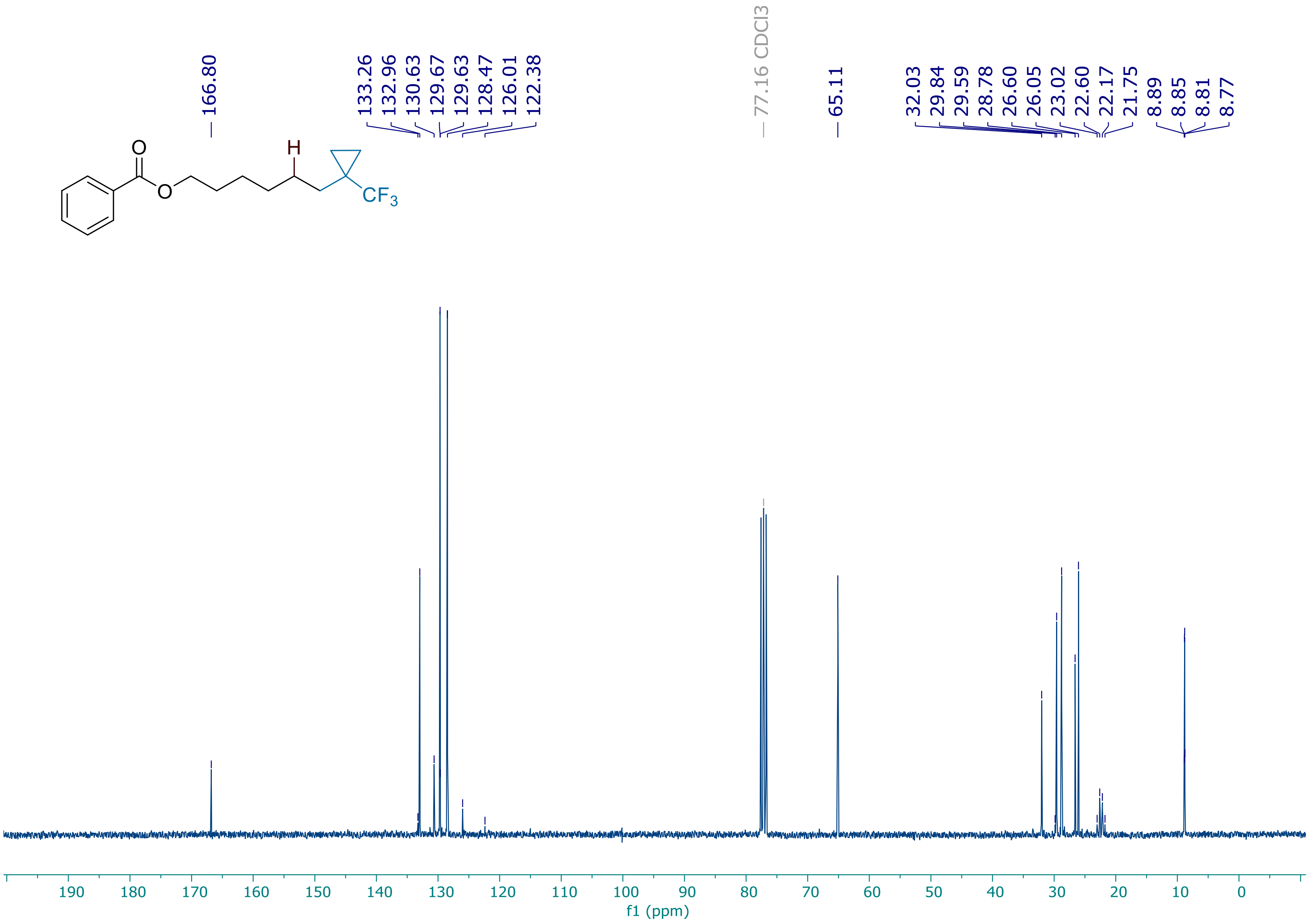

166.80
133.26
132.96
130.63
129.67
129.63
128.47
126.01
122.38
77.16 CDCl3
65.11
32.03
29.84
29.59
28.78
26.60
26.05
23.02
22.60
22.17
21.75
8.89
8.85
8.81
8.77
O
O
H
CF3
190
180
170
160
150
140
130
120
110
100
90
80
70
60
50
40
30
20
10
0
f1 (ppm)

^{19}F NMR of **3**

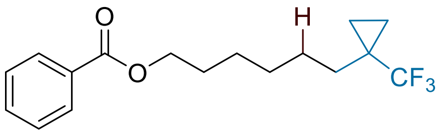
O
O
H
CF3

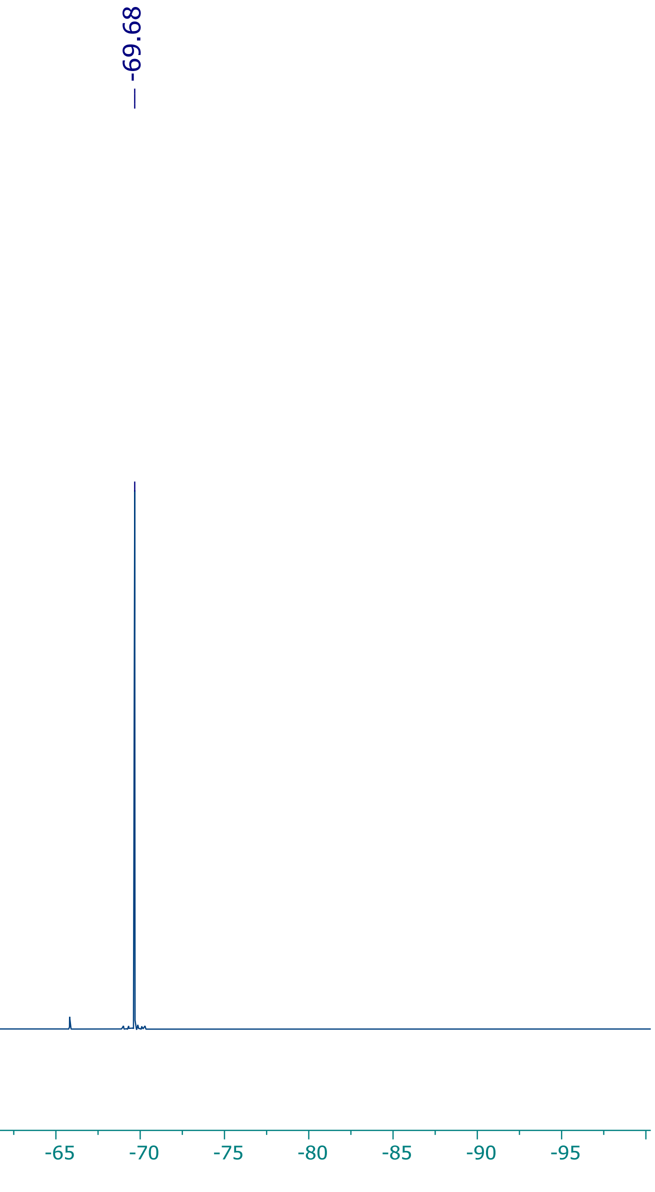
-69.68
0
-5
-10
-15
-20
-25
-30
-35
-40
-45
-50
-55
-60
-65
-70
-75
-80
-85
-90
-95
f1 (ppm)

^{1}H NMR of **4**

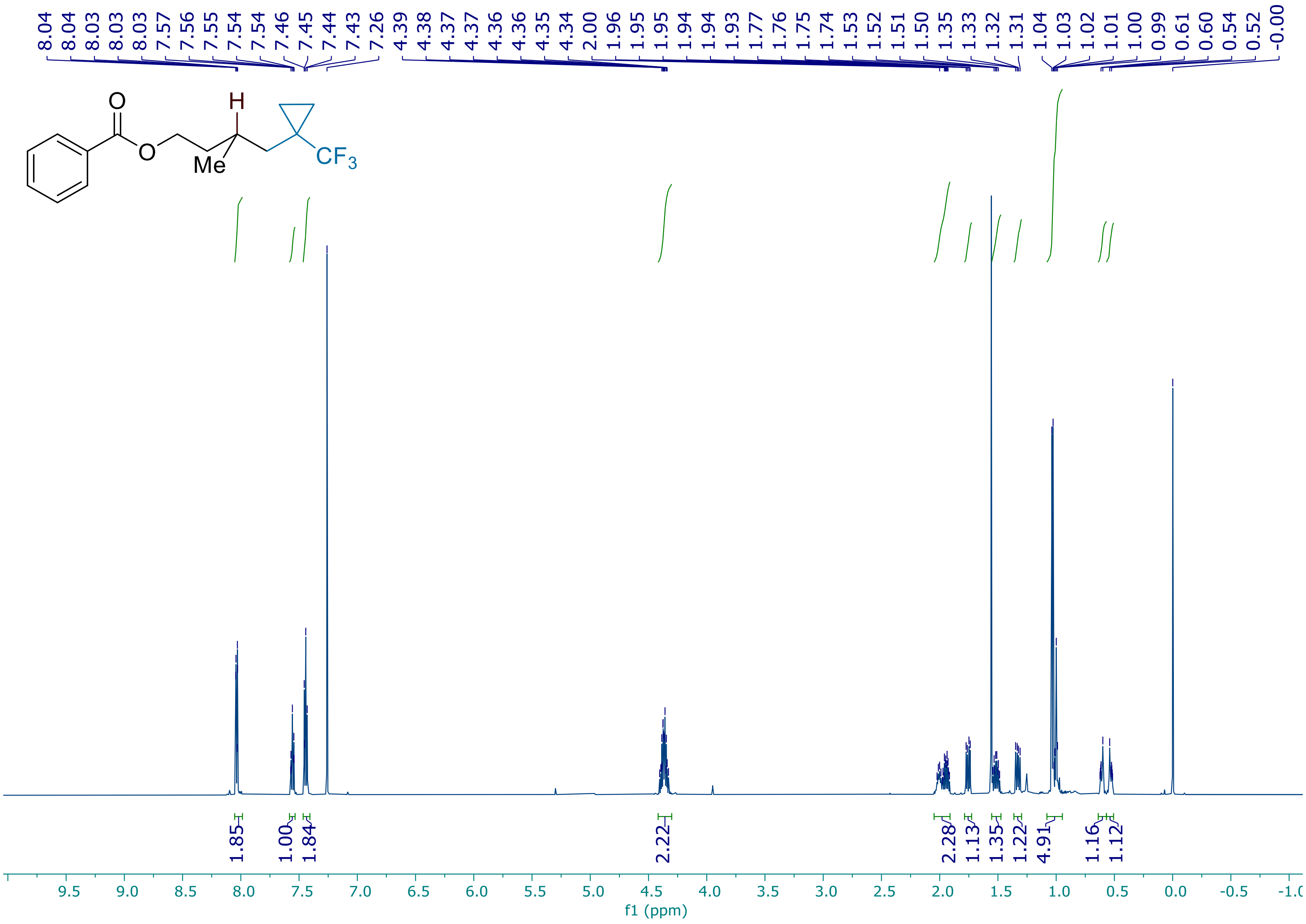
8.04 8.04 8.03 8.03 8.03 7.57 7.56 7.55 7.54 7.54 7.46 7.45 7.44 7.43 7.26 4.39 4.38 4.37 4.37 4.36 4.36 4.35 4.34 2.00 1.96 1.95 1.95 1.94 1.94 1.93 1.77 1.76 1.75 1.74 1.53 1.52 1.51 1.50 1.35 1.33 1.32 1.31 1.04 1.03 1.02 1.01 1.00 0.99 0.61 0.60 0.54 0.52 -0.00
O
H
O
Me
CF3
1.85 1.00 1.84 2.22 2.28 1.13 1.35 1.22 4.91 1.16 1.12
9.5 9.0 8.5 8.0 7.5 7.0 6.5 6.0 5.5 5.0 4.5 4.0 3.5 3.0 2.5 2.0 1.5 1.0 0.5 0.0 -0.5
f1 (ppm)

^{13}C NMR of **4**

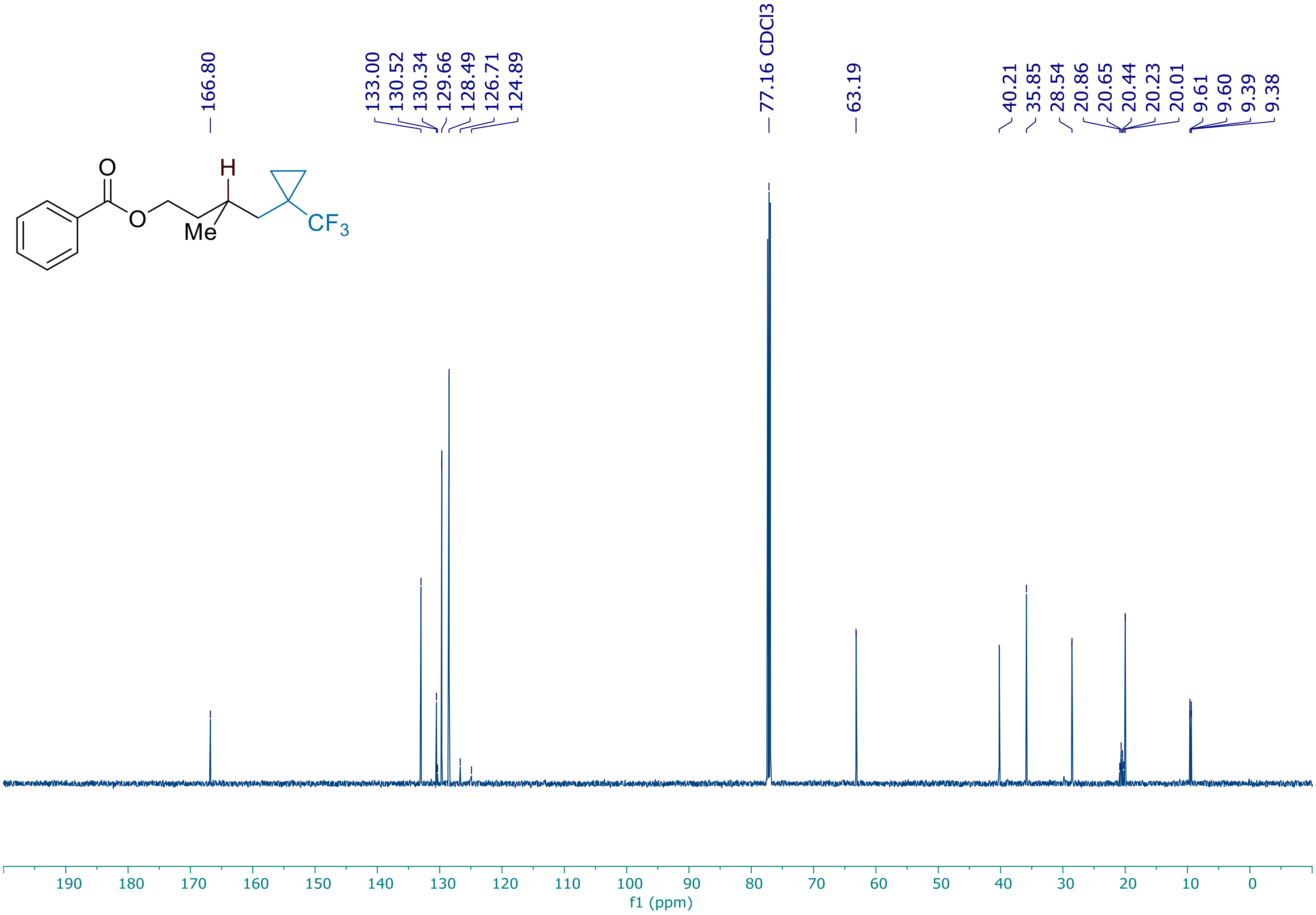

166.80
133.00
130.52
130.34
129.66
128.49
126.71
124.89
77.16 CDCl3
63.19
40.21
35.85
28.54
20.86
20.65
20.44
20.23
20.01
9.61
9.60
9.39
9.38
O
H
O
Me
CF3
190
180
170
160
150
140
130
120
110
100
90
80
70
60
50
40
30
20
10
0
f1 (ppm)

^{19}F NMR of **4**

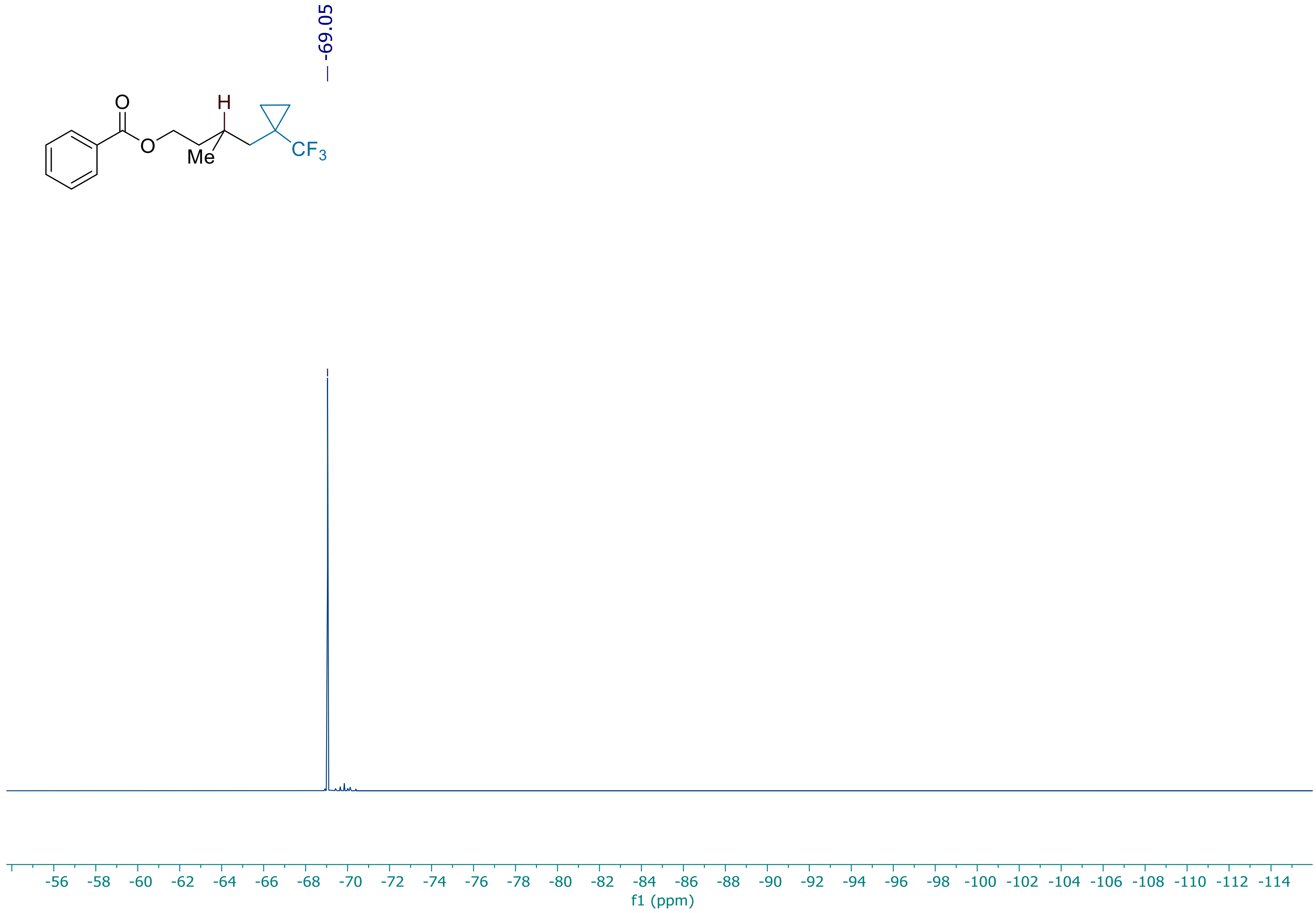

O
O
H
Me
CF3
-69.05
-56
-58
-60
-62
-64
-66
-68
-70
-72
-74
-76
-78
-80
-82
-84
-86
-88
-90
-92
-94
-96
-98
-100
-102
-104
-106
-108
-110
-112
-114
f1 (ppm)

^{1}H NMR of **5**

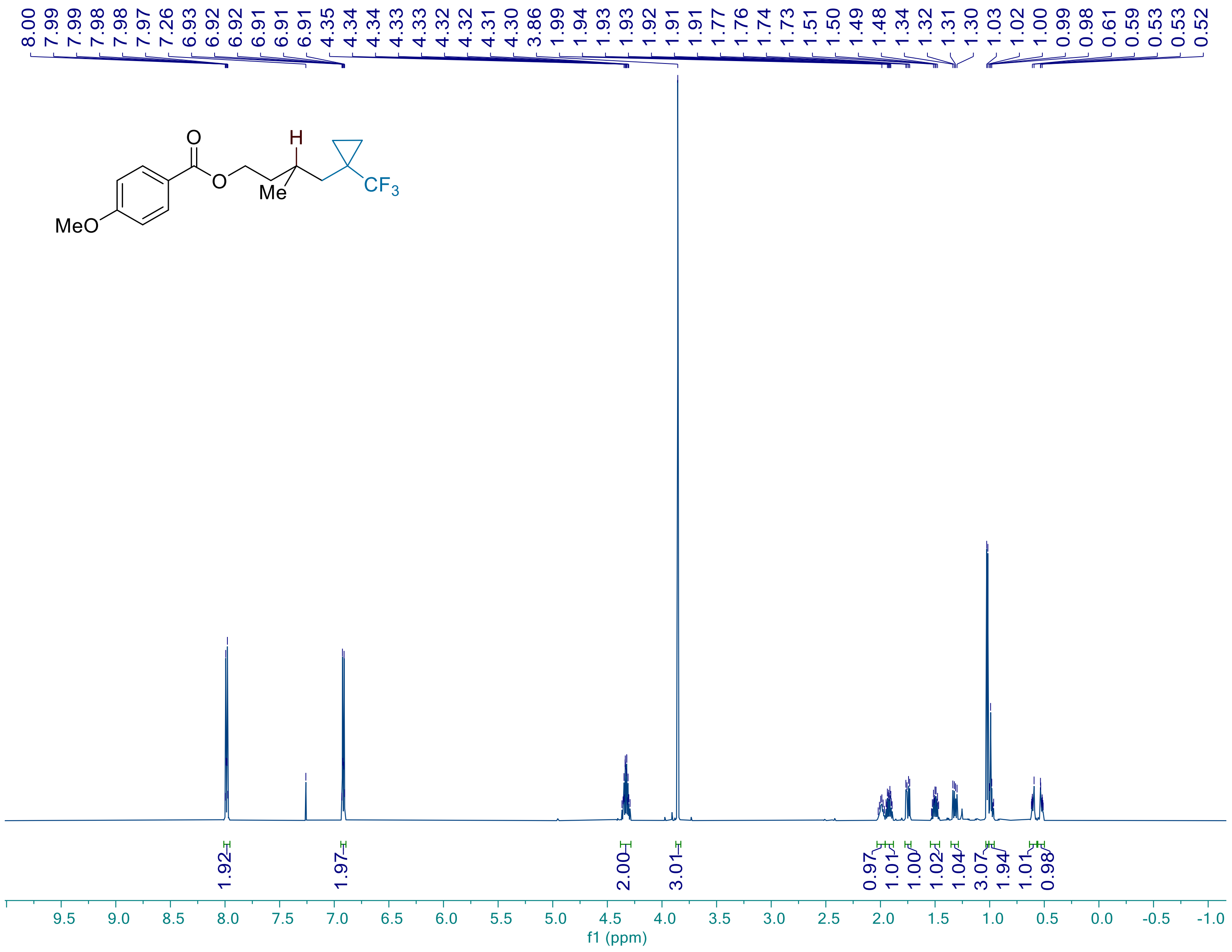

8.00
7.99
7.99
7.98
7.98
7.97
7.26
6.93
6.92
6.92
6.91
6.91
6.91
4.35
4.34
4.34
4.33
4.33
4.32
4.32
4.31
4.30
3.86
1.99
1.94
1.93
1.93
1.92
1.91
1.91
1.77
1.76
1.74
1.73
1.51
1.50
1.49
1.48
1.34
1.32
1.31
1.30
1.03
1.02
1.00
0.99
0.98
0.61
0.59
0.53
0.53
0.52
O
H
O
Me
CF3
MeO
1.92
1.97
2.00
3.01
0.97
1.01
1.00
1.02
1.04
3.07
1.94
1.01
0.98
9.5
9.0
8.5
8.0
7.5
7.0
6.5
6.0
5.5
5.0
4.5
4.0
3.5
3.0
2.5
2.0
1.5
1.0
0.5
0.0
-0.5
-1.0
f1 (ppm)

^{13}C NMR of **5**

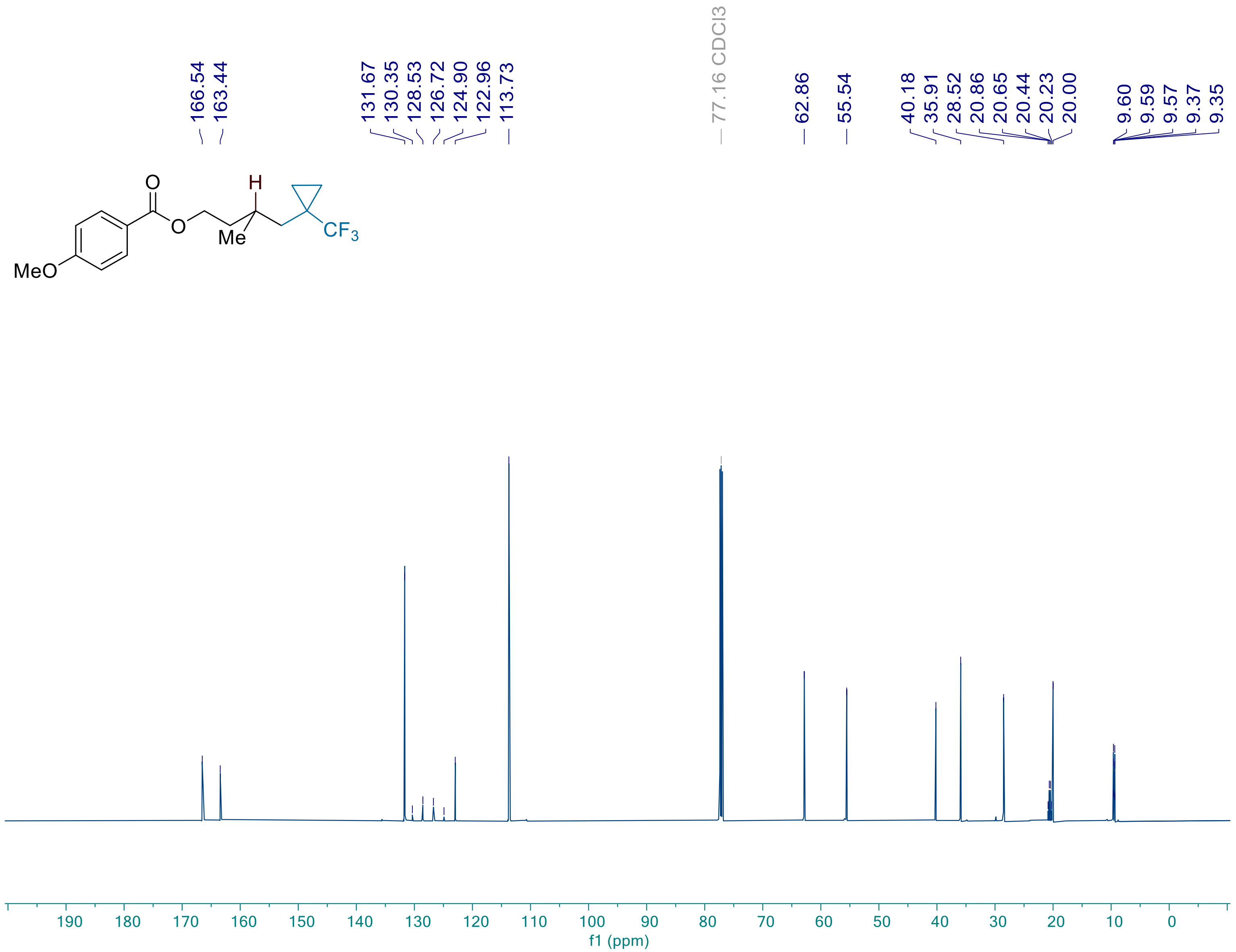

166.54
163.44
131.67
130.35
128.53
126.72
124.90
122.96
113.73
77.16 CDCl3
62.86
55.54
40.18
35.91
28.52
20.86
20.65
20.44
20.23
20.00
9.60
9.59
9.57
9.37
9.35
O
H
O
Me
CF3
MeO
190
180
170
160
150
140
130
120
110
100
90
80
70
60
50
40
30
20
10
0
f1 (ppm)

^{19}F NMR of **5**

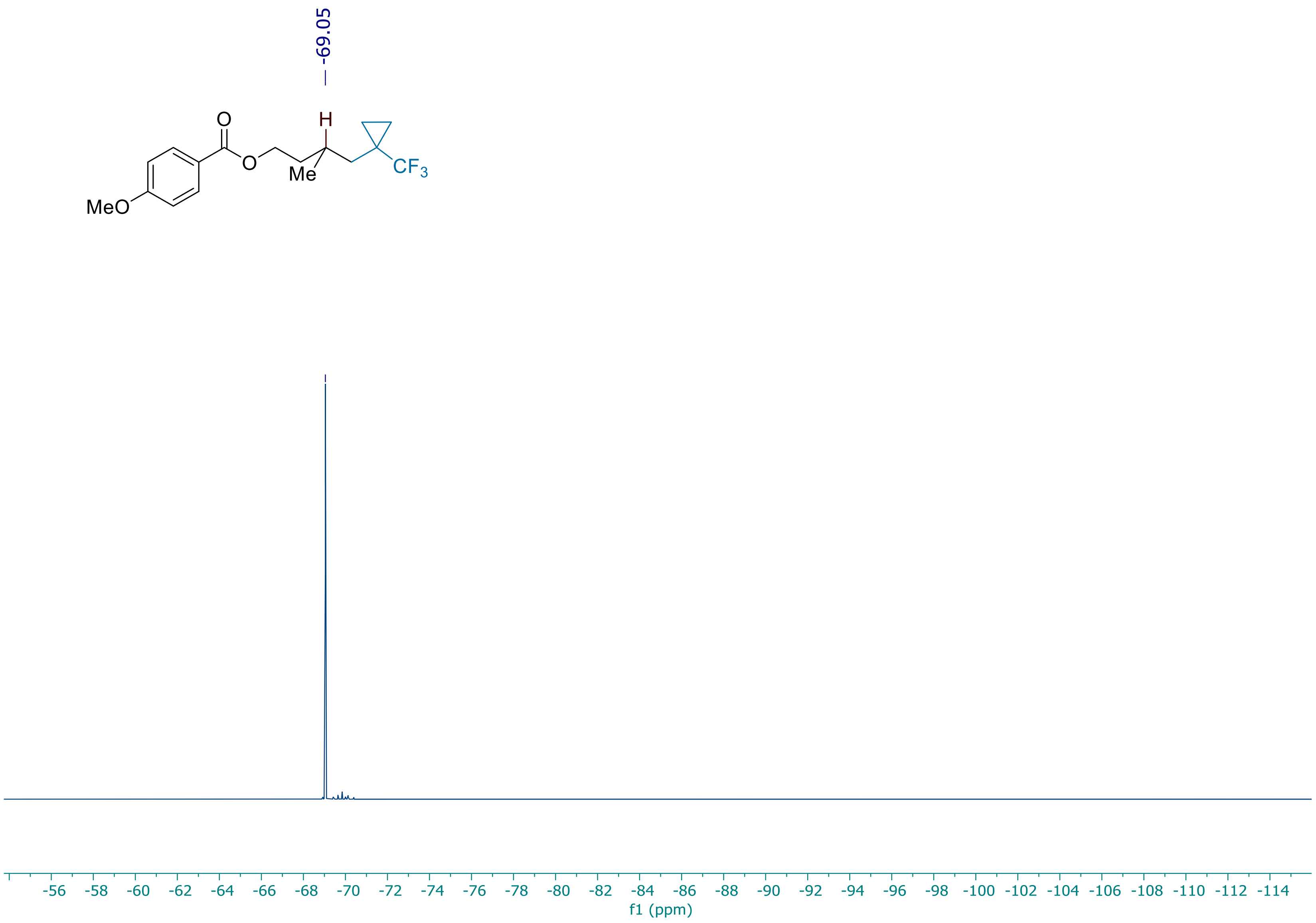

-69.05
O
O
H
Me
MeO
CF3
-56 -58 -60 -62 -64 -66 -68 -70 -72 -74 -76 -78 -80 -82 -84 -86 -88 -90 -92 -94 -96 -98 -100 -102 -104 -106 -108 -110 -112 -114
f1 (ppm)

^{1}H NMR of **6**

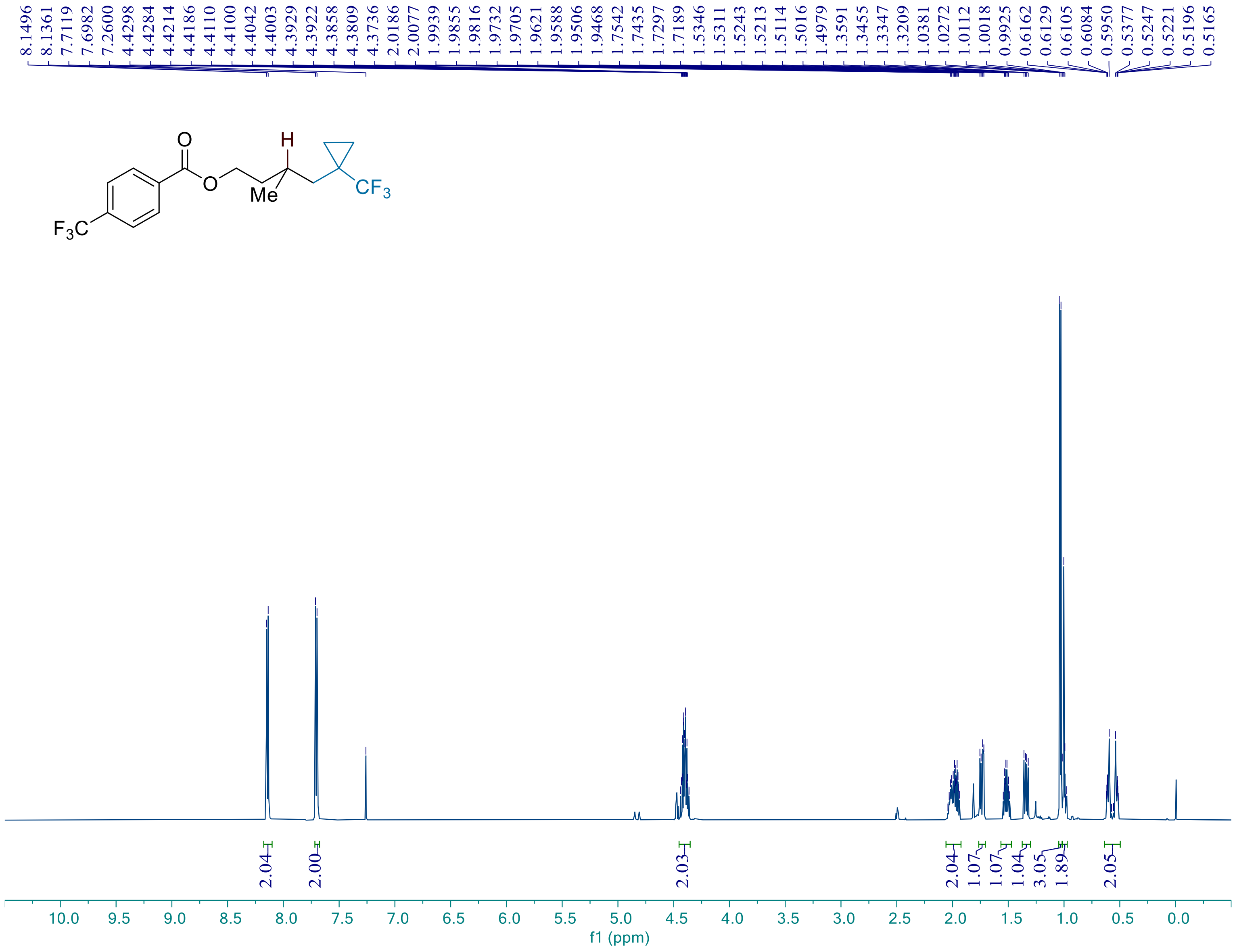

8.1496
8.1361
7.7119
7.6982
7.2600
4.4298
4.4284
4.4214
4.4186
4.4110
4.4100
4.4042
4.4003
4.3929
4.3922
4.3858
4.3809
4.3736
2.0186
2.0077
1.9939
1.9855
1.9816
1.9732
1.9705
1.9621
1.9588
1.9506
1.9468
1.7542
1.7435
1.7297
1.7189
1.5346
1.5311
1.5243
1.5213
1.5114
1.5016
1.4979
1.3591
1.3455
1.3347
1.3209
1.0381
1.0272
1.0112
1.0018
0.9925
0.6162
0.6129
0.6105
0.6084
0.5950
0.5377
0.5247
0.5221
0.5196
0.5165
O
H
O
Me
CF3
F3C
2.04
2.00
2.03
2.04
1.07
1.07
1.04
3.05
1.89
2.05
10.0
9.5
9.0
8.5
8.0
7.5
7.0
6.5
6.0
5.5
5.0
4.5
4.0
3.5
3.0
2.5
2.0
1.5
1.0
0.5
0.0
f1 (ppm)

^{13}C NMR of **6**

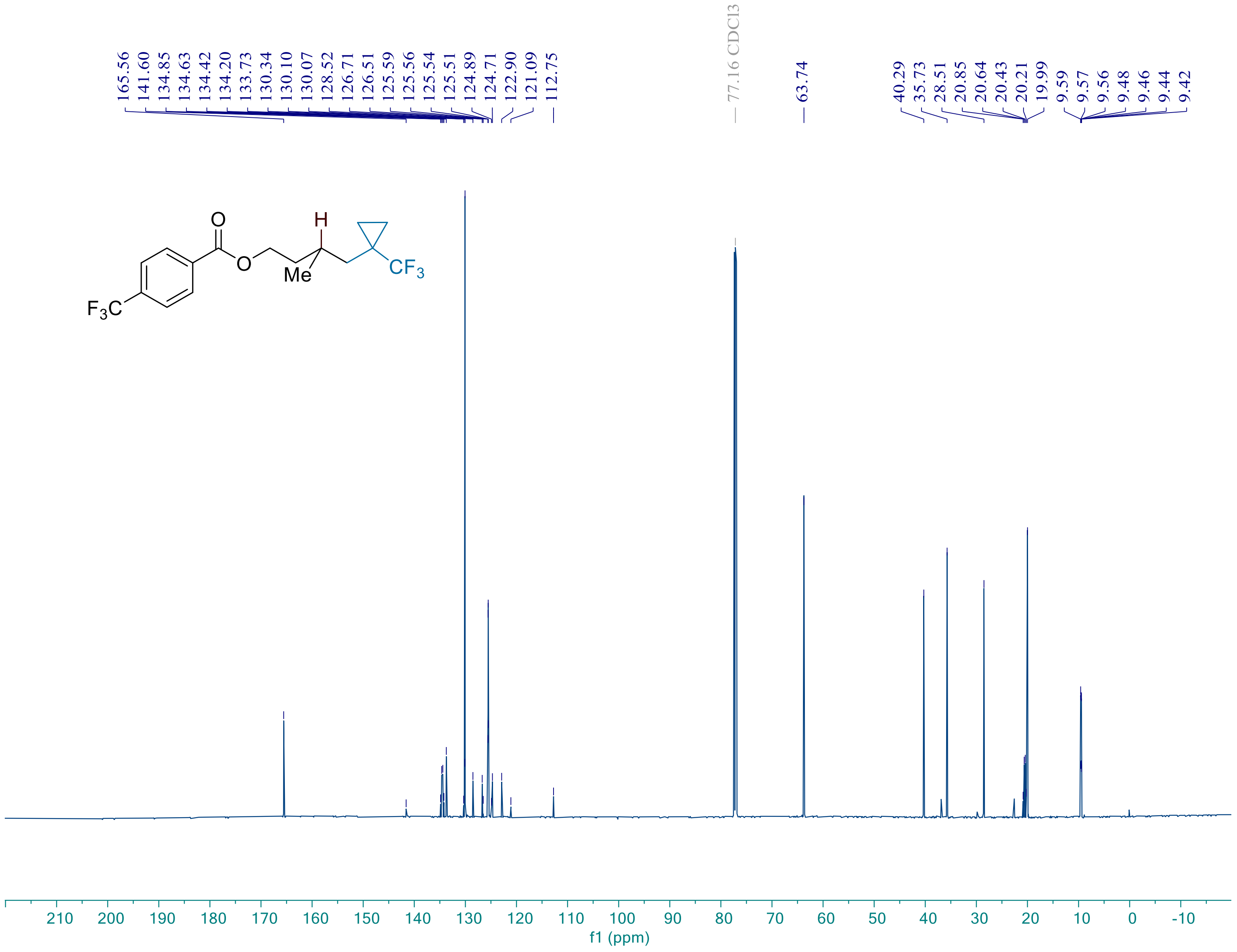
165.56
141.60
134.85
134.63
134.42
134.20
133.73
130.34
130.10
130.07
128.52
126.71
126.51
125.59
125.56
125.54
125.51
124.89
124.71
122.90
121.09
112.75
77.16 CDCl3
63.74
40.29
35.73
28.51
20.85
20.64
20.43
20.21
19.99
9.59
9.57
9.56
9.48
9.46
9.44
9.42
O
H
O
Me
CF3
F3C
210 200 190 180 170 160 150 140 130 120 110 100 90 80 70 60 50 40 30 20 10 0 -10
f1 (ppm)

^{19}F NMR of **6**

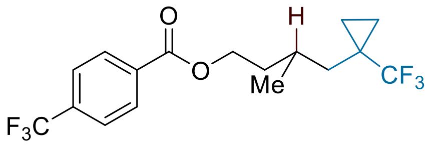

O
O
H
Me
CF3
F3C

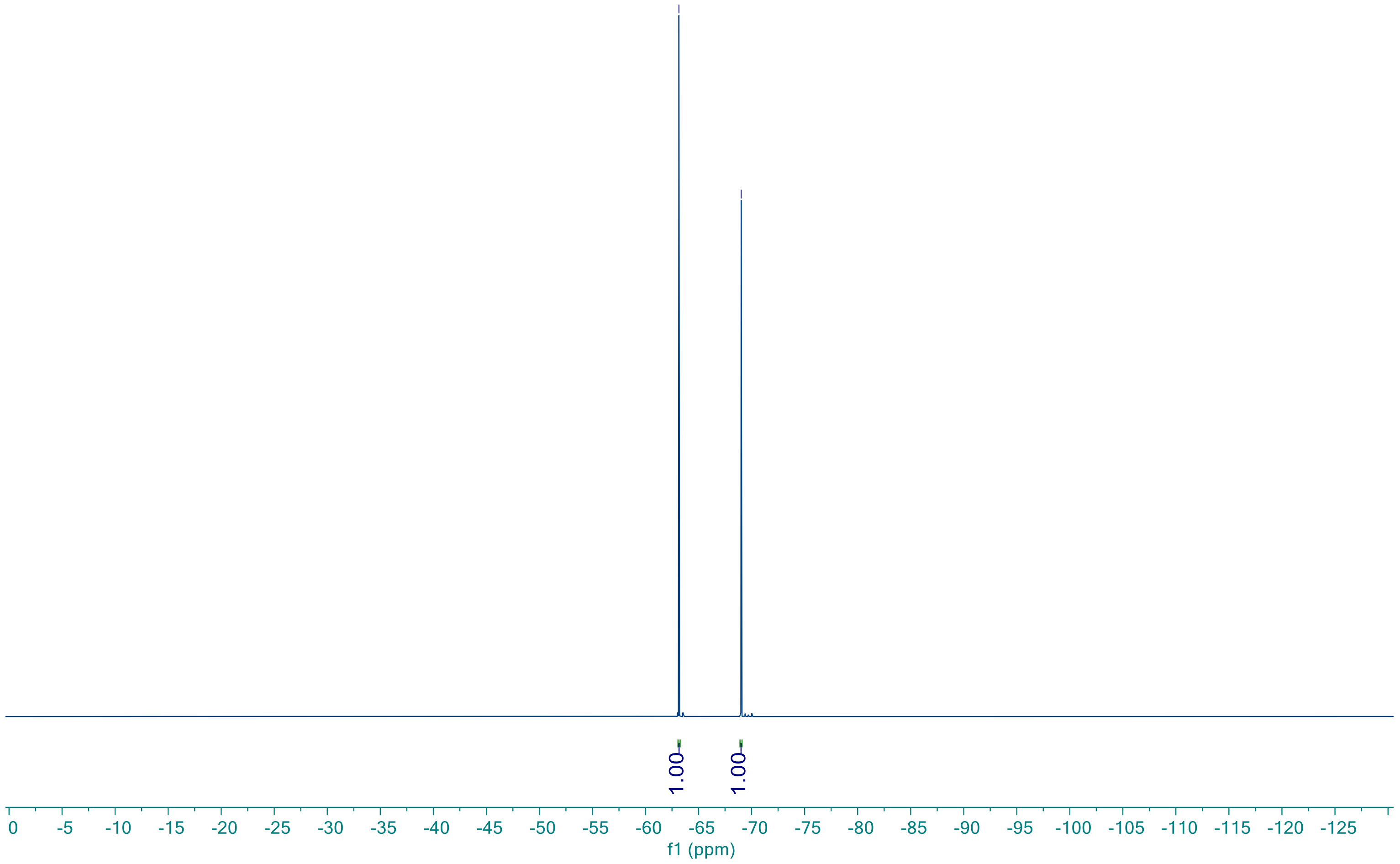

-63.15
-69.02
1.00
1.00
0
-5
-10
-15
-20
-25
-30
-35
-40
-45
-50
-55
-60
-65
-70
-75
-80
-85
-90
-95
-100
-105
-110
-115
-120
-125
f1 (ppm)

^{1}H NMR of **7**

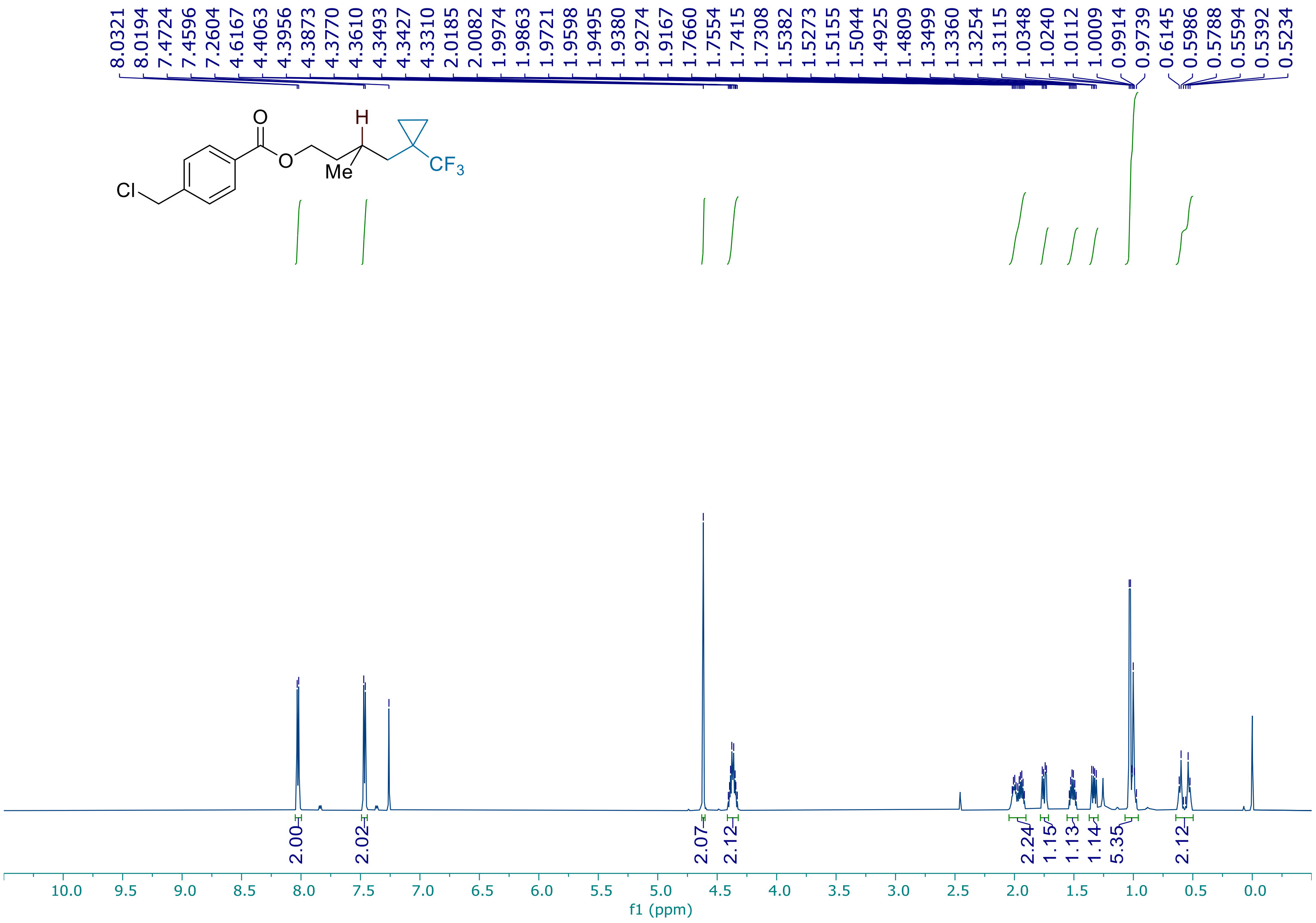

^{13}C NMR of **7**

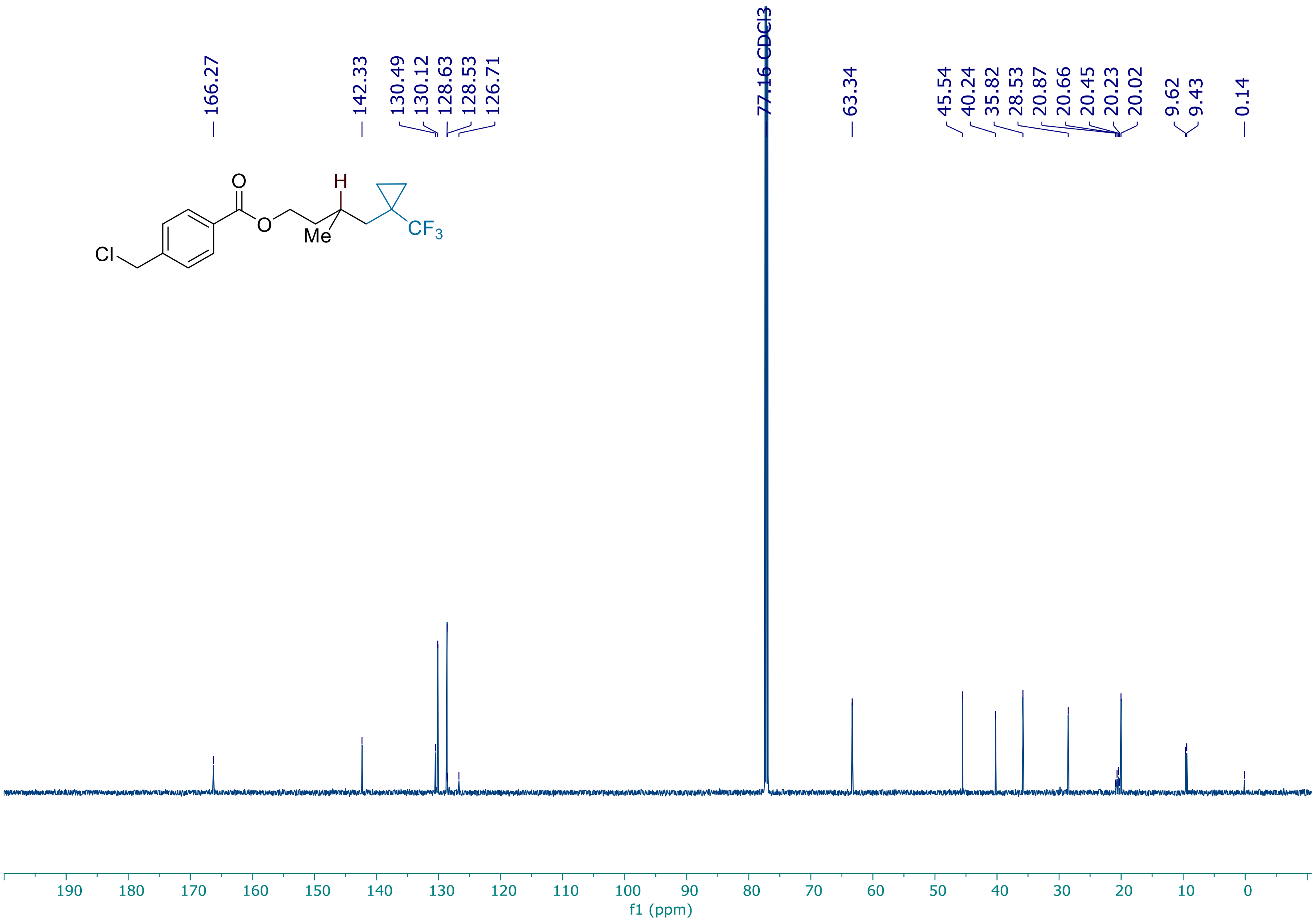

166.27
142.33
130.49
130.12
128.63
128.53
126.71
77.16 CDCl3
63.34
45.54
40.24
35.82
28.53
20.87
20.66
20.45
20.23
20.02
9.62
9.43
0.14
O
H
O
Me
CF3
Cl
190
180
170
160
150
140
130
120
110
100
90
80
70
60
50
40
30
20
10
0
f1 (ppm)

^{19}F NMR of **7**

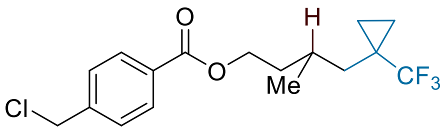

O
H
O
Me
CF3
Cl

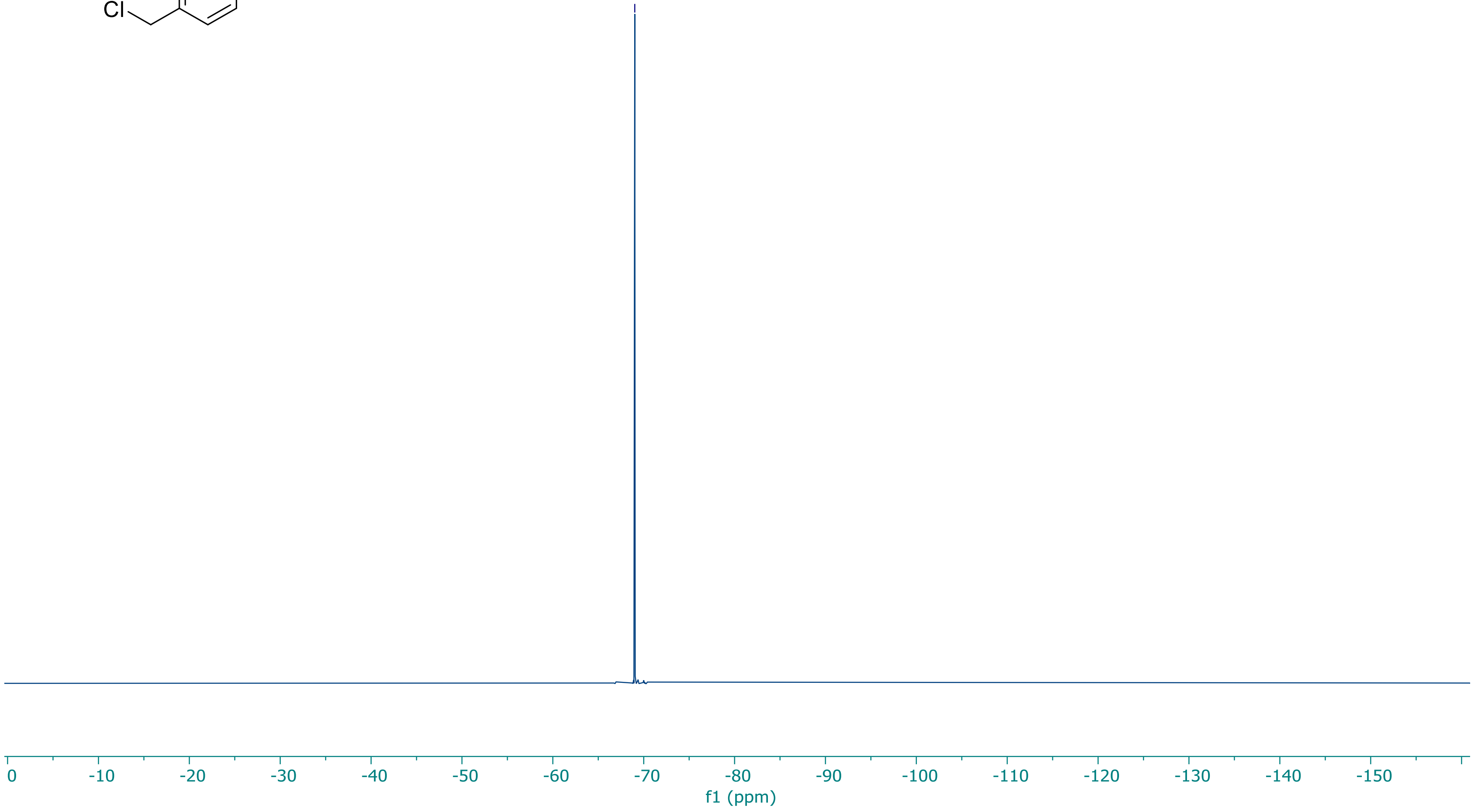

-69.02
0
-10
-20
-30
-40
-50
-60
-70
-80
-90
-100
-110
-120
-130
-140
-150
f1 (ppm)

^{1}H NMR of **8**

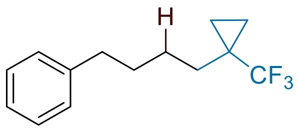

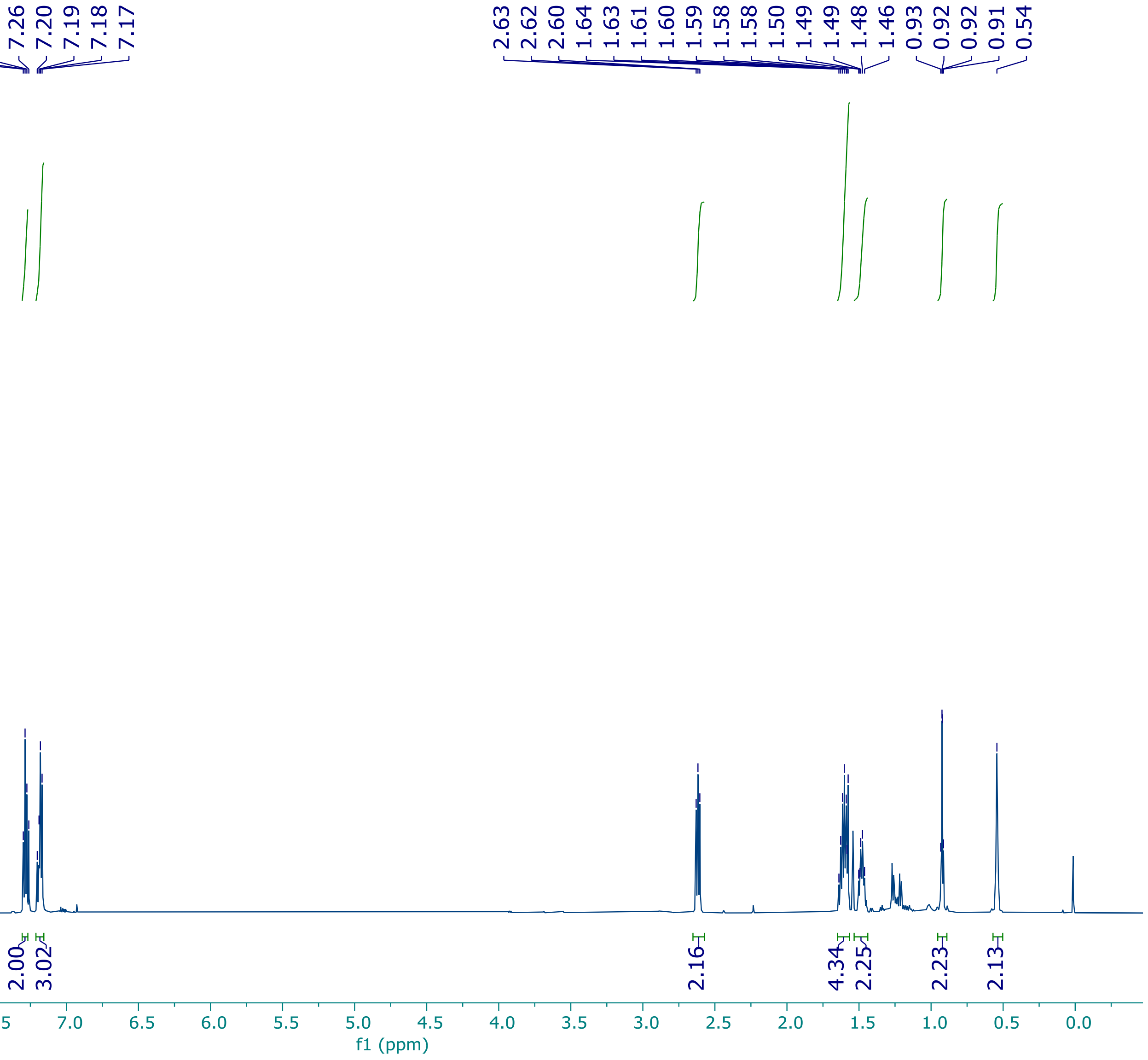
7.30
7.29
7.27
7.26
7.20
7.19
7.18
7.17
2.63
2.62
2.60
1.64
1.63
1.61
1.60
1.59
1.58
1.58
1.50
1.49
1.49
1.48
1.46
0.93
0.92
0.92
0.91
0.54
2.00
3.02
2.16
4.34
2.25
2.23
2.13
9.5
9.0
8.5
8.0
7.5
7.0
6.5
6.0
5.5
5.0
4.5
4.0
3.5
3.0
2.5
2.0
1.5
1.0
0.5
0.0
f1 (ppm)

^{13}C NMR of **8**

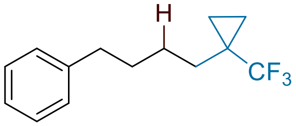
H
CF3

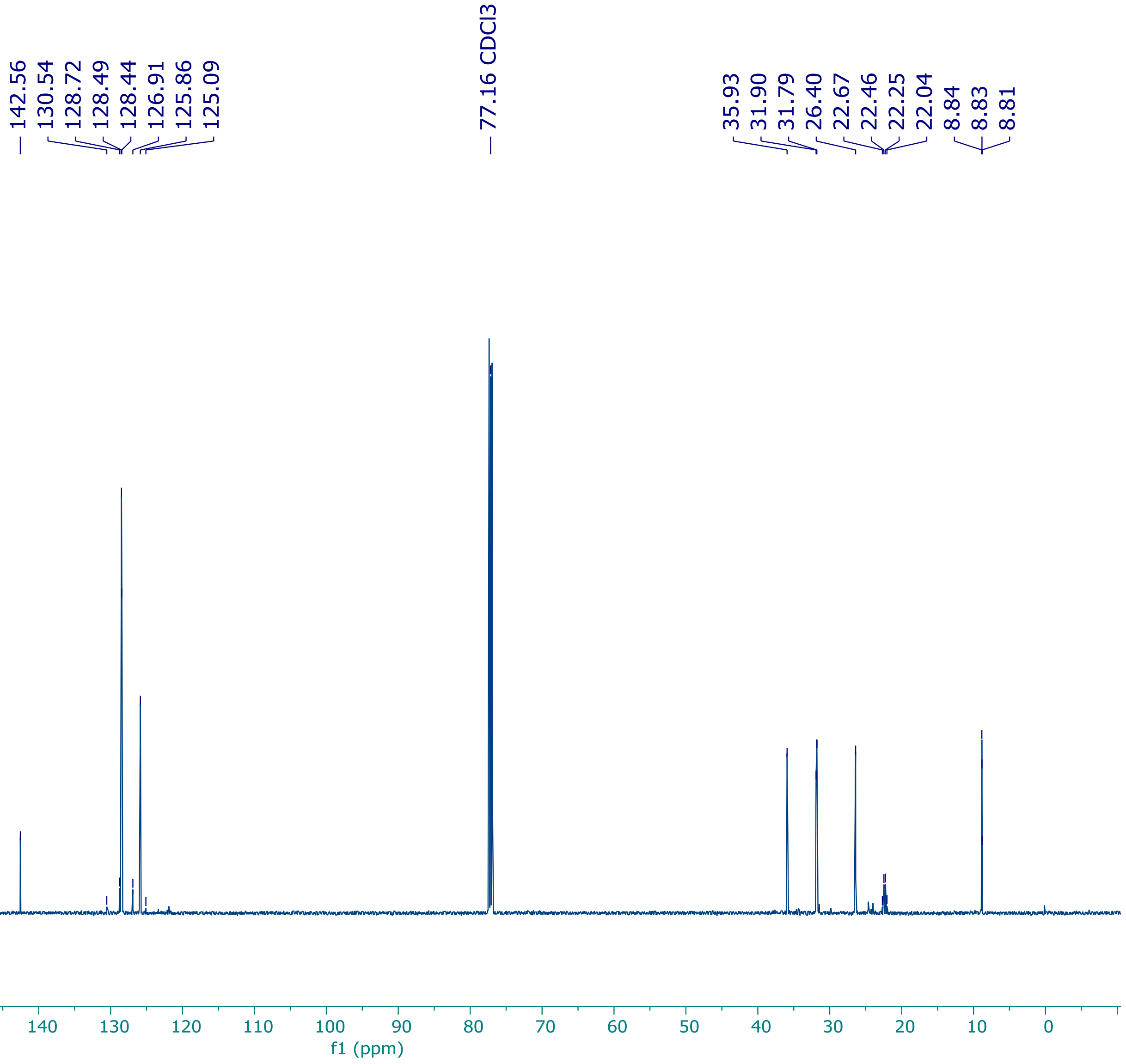
142.56
130.54
128.72
128.49
128.44
126.91
125.86
125.09
77.16 CDCl3
35.93
31.90
31.79
26.40
22.67
22.46
22.25
22.04
8.84
8.83
8.81
190
180
170
160
150
140
130
120
110
100
90
80
70
60
50
40
30
20
10
0
f1 (ppm)

^{19}F NMR of **8**

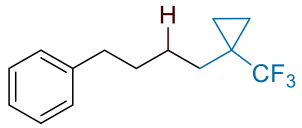

H
CF3

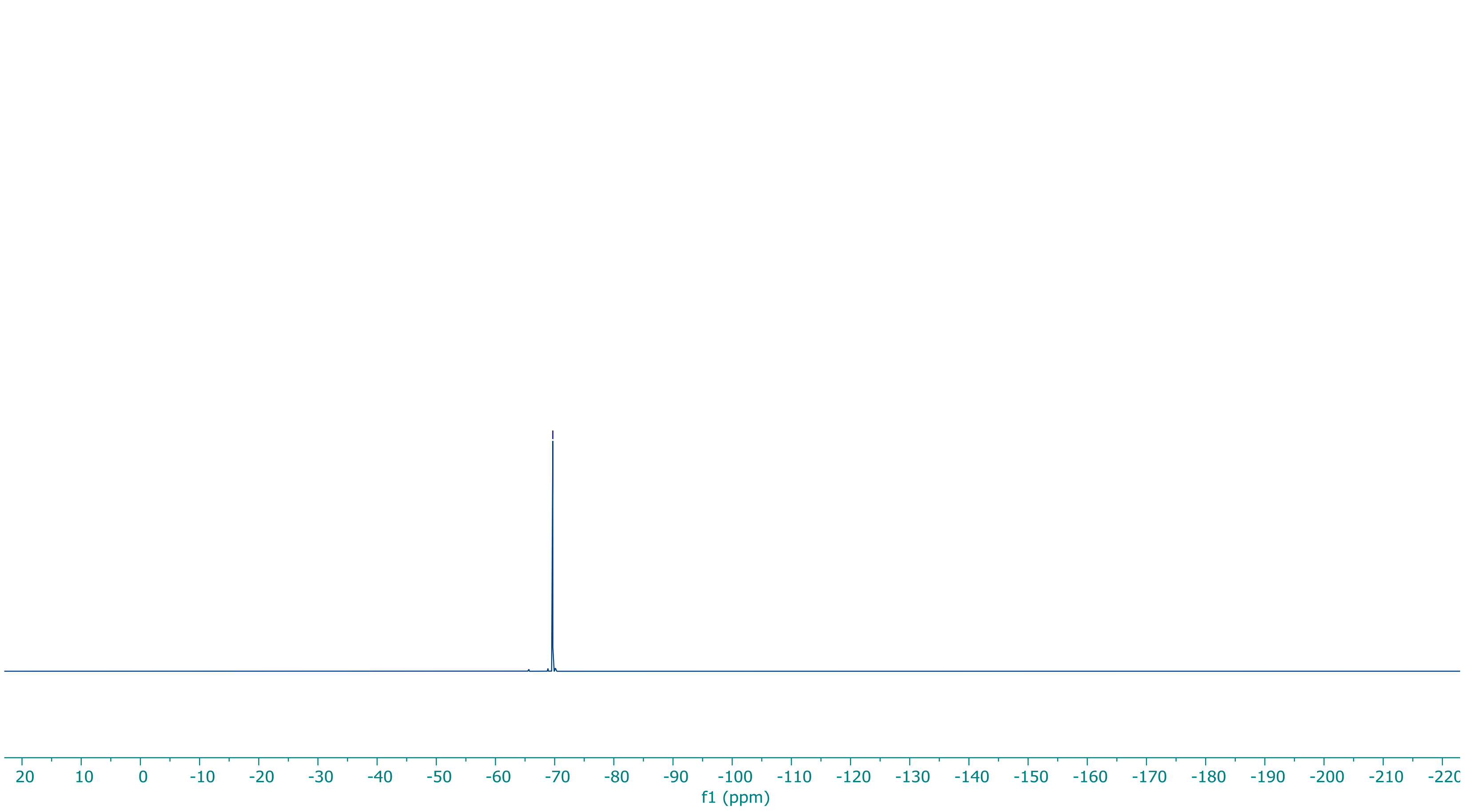

-69.69
20
10
0
-10
-20
-30
-40
-50
-60
-70
-80
-90
-100
-110
-120
-130
-140
-150
-160
-170
-180
-190
-200
-210
-220
f1 (ppm)

^{1}H NMR of **9**

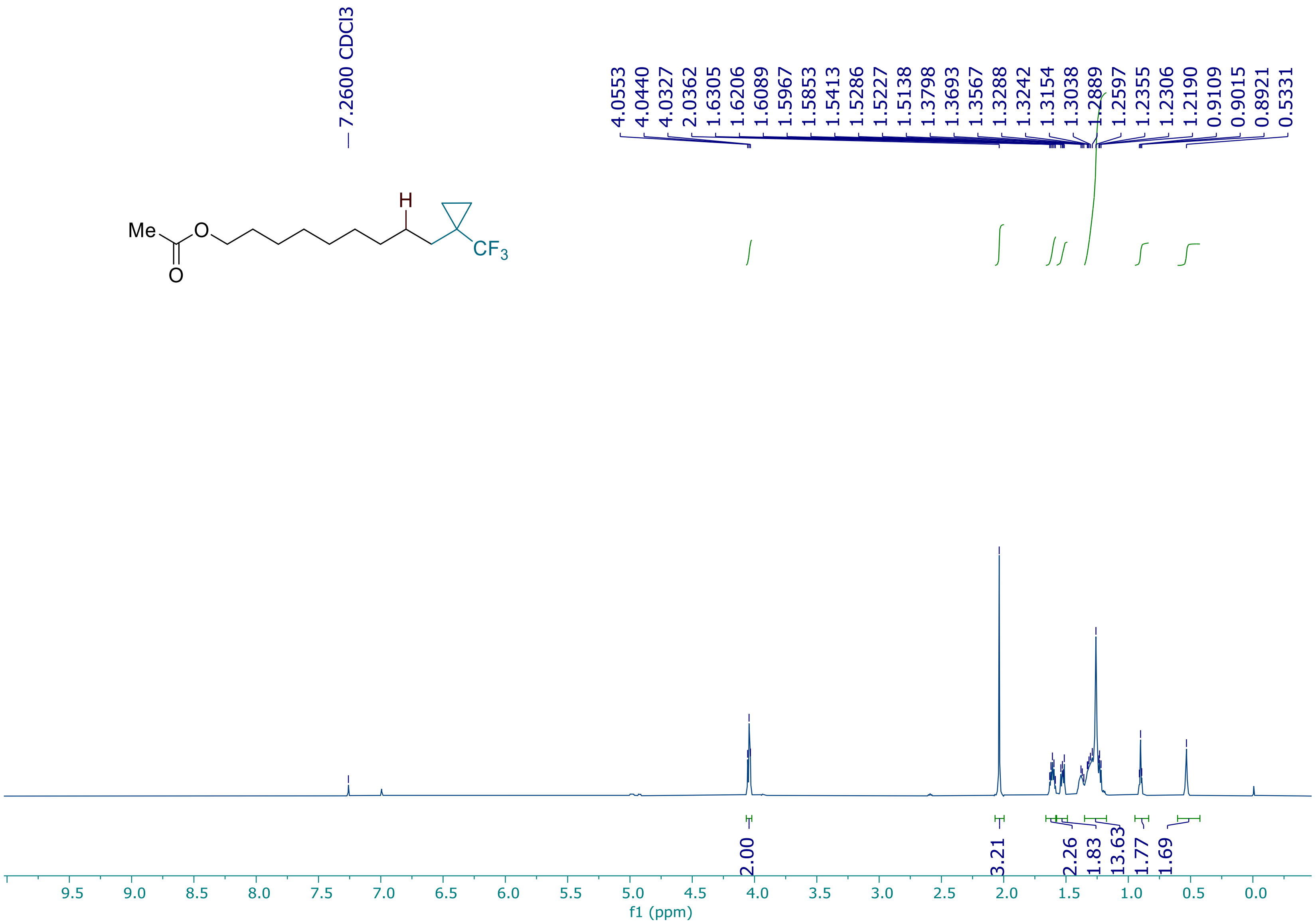

7.2600 CDCl3
4.0553
4.0440
4.0327
2.0362
1.6305
1.6206
1.6089
1.5967
1.5853
1.5413
1.5286
1.5227
1.5138
1.3798
1.3693
1.3567
1.3288
1.3242
1.3154
1.3038
1.2889
1.2597
1.2355
1.2306
1.2190
0.9109
0.9015
0.8921
0.5331
Me
O
O
H
CF3
2.00
3.21
2.26
1.83
13.63
1.77
1.69
9.5
9.0
8.5
8.0
7.5
7.0
6.5
6.0
5.5
5.0
4.5
4.0
3.5
3.0
2.5
2.0
1.5
1.0
0.5
0.0
f1 (ppm)

^{13}C NMR of **9**

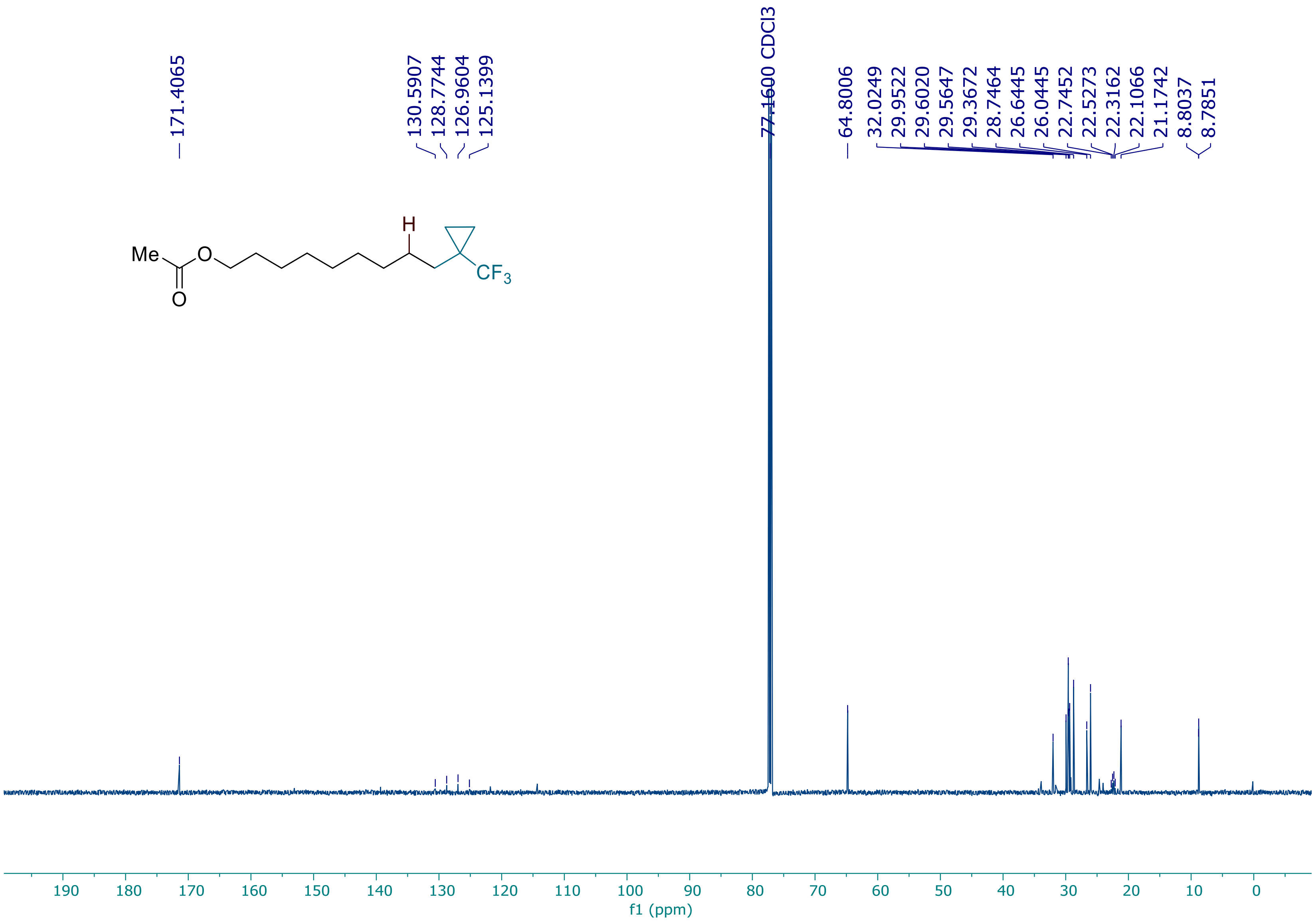

171.4065
130.5907
128.7744
126.9604
125.1399
77.1600 CDCl3
64.8006
32.0249
29.9522
29.6020
29.5647
29.3672
28.7464
26.6445
26.0445
22.7452
22.5273
22.3162
22.1066
21.1742
8.8037
8.7851
Me
O
O
H
CF3
190
180
170
160
150
140
130
120
110
100
90
80
70
60
50
40
30
20
10
0
f1 (ppm)

^{19}F NMR of **9**

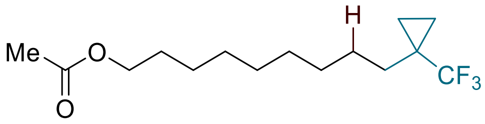

Me
O
O
H
CF3

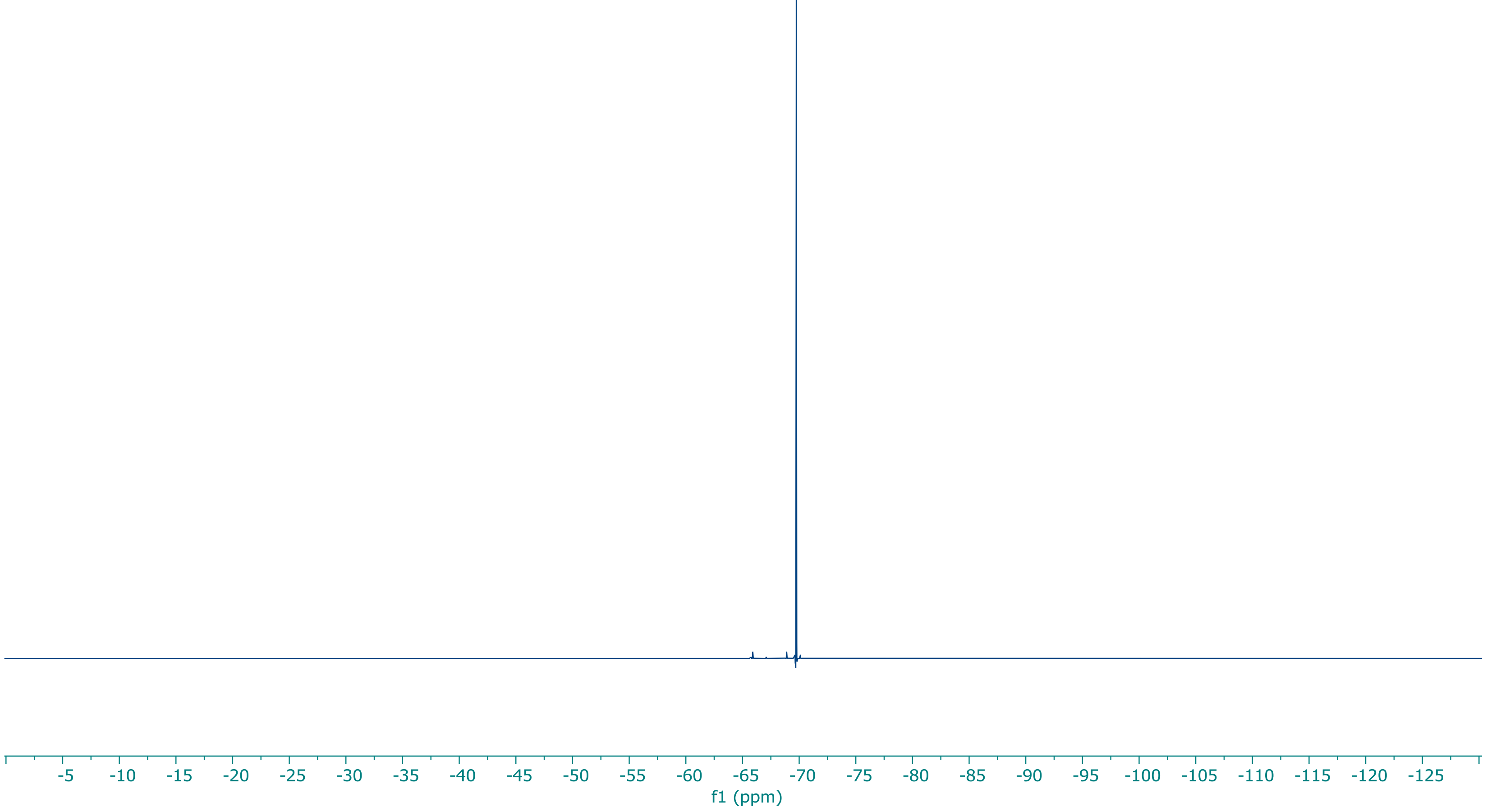

-69.75
-5
-10
-15
-20
-25
-30
-35
-40
-45
-50
-55
-60
-65
-70
-75
-80
-85
-90
-95
-100
-105
-110
-115
-120
-125
f1 (ppm)

^{1}H NMR of **10**

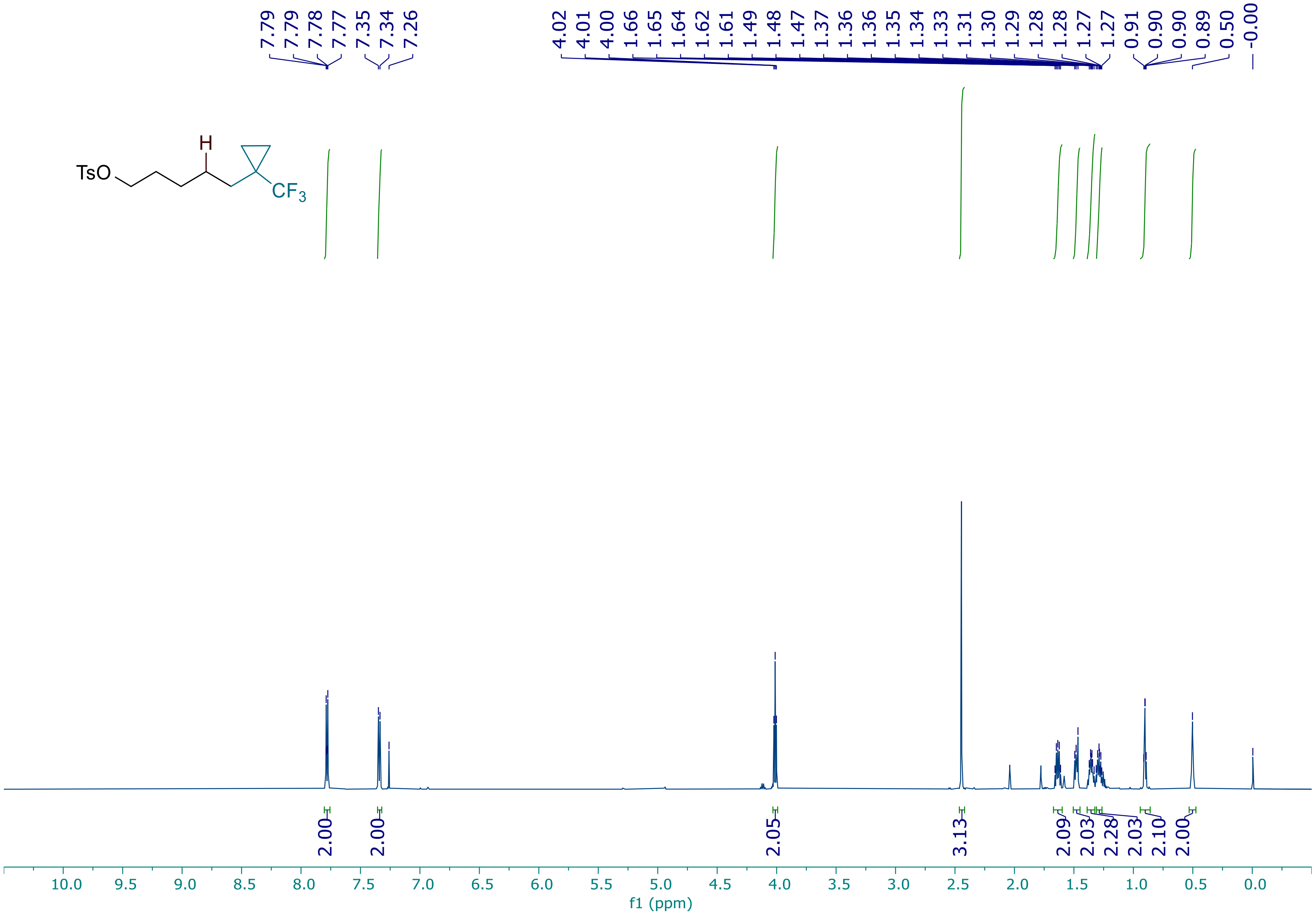
7.79
7.79
7.78
7.77
7.35
7.34
7.26
4.02
4.01
4.00
1.66
1.65
1.64
1.62
1.61
1.49
1.48
1.47
1.37
1.36
1.36
1.35
1.34
1.33
1.31
1.30
1.29
1.28
1.28
1.27
1.27
0.91
0.90
0.90
0.89
0.50
-0.00
TsO
H
CF3
2.00
2.00
2.05
3.13
2.09
2.03
2.28
2.03
2.10
2.00
10.0
9.5
9.0
8.5
8.0
7.5
7.0
6.5
6.0
5.5
5.0
4.5
4.0
3.5
3.0
2.5
2.0
1.5
1.0
0.5
0.0
f1 (ppm)

^{13}C NMR of **10**

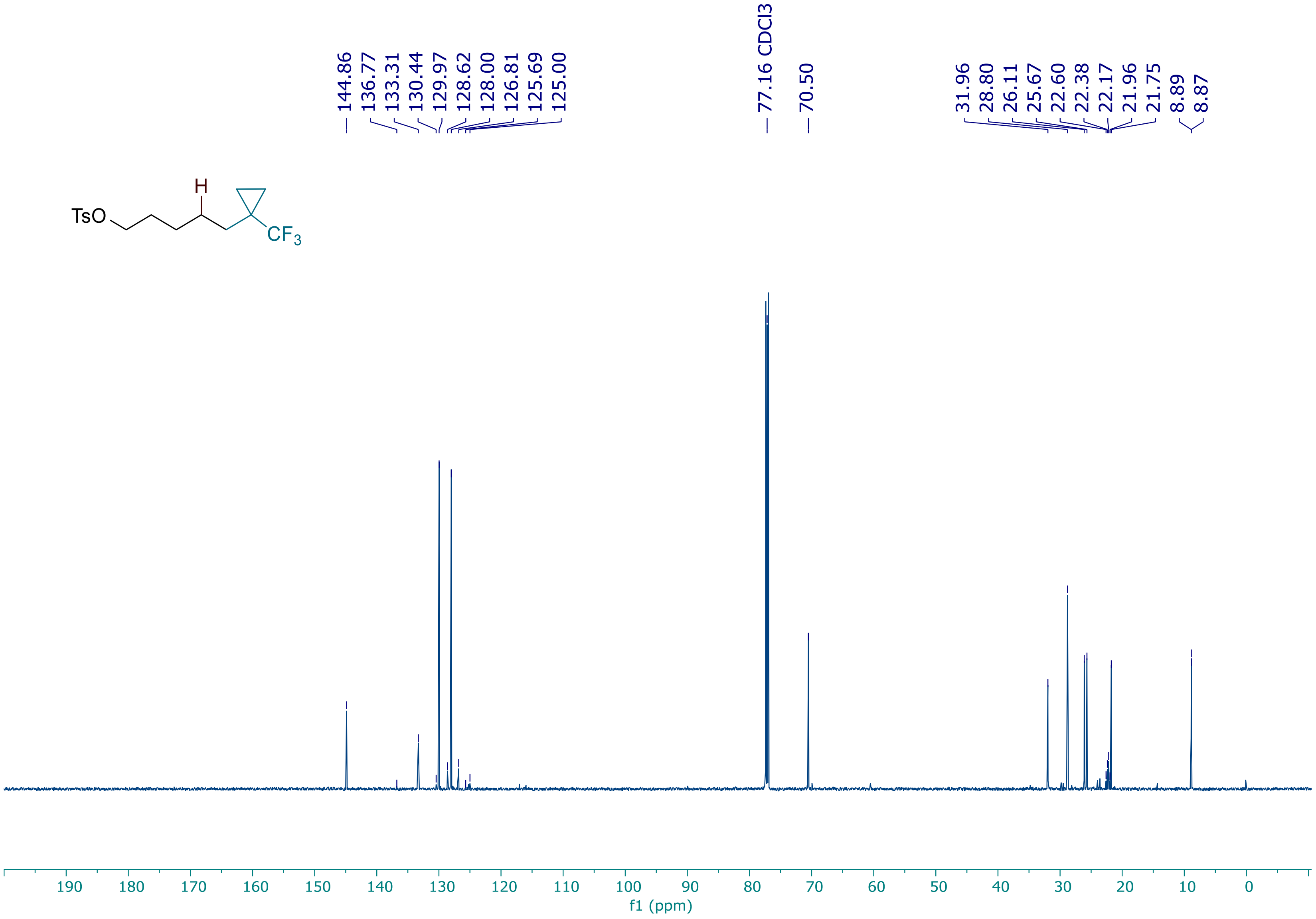

TsO
H
CF3
144.86
136.77
133.31
130.44
129.97
128.62
128.00
126.81
125.69
125.00
77.16 CDCl3
70.50
31.96
28.80
26.11
25.67
22.60
22.38
22.17
21.96
21.75
8.89
8.87
190
180
170
160
150
140
130
120
110
100
90
80
70
60
50
40
30
20
10
0
f1 (ppm)

^{19}F NMR of **10**

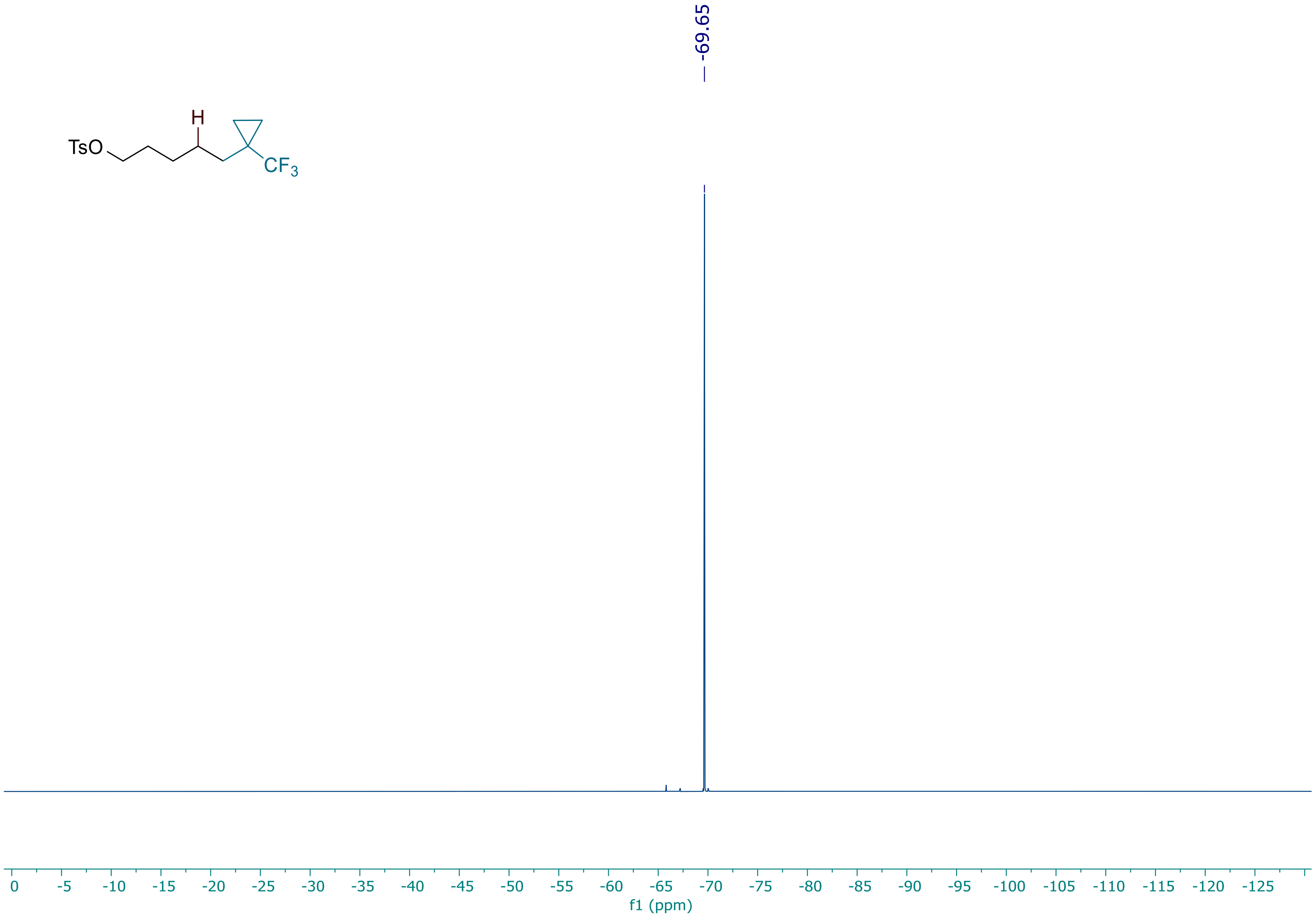

TsO
H
CF3
-69.65
0
-5
-10
-15
-20
-25
-30
-35
-40
-45
-50
-55
-60
-65
-70
-75
-80
-85
-90
-95
-100
-105
-110
-115
-120
-125
f1 (ppm)

^{1}H NMR of **11**

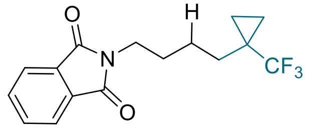

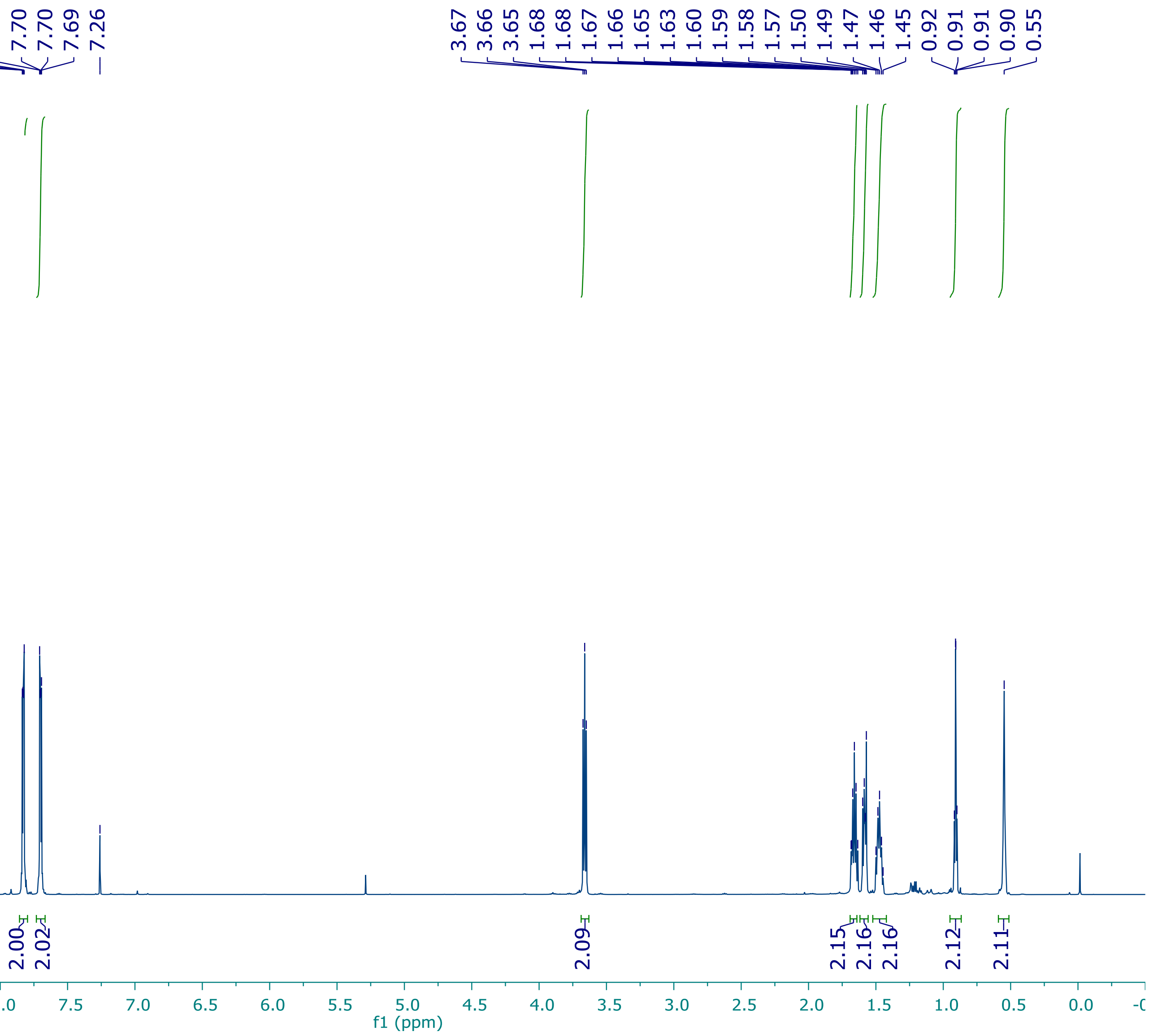
7.84
7.83
7.83
7.82
7.71
7.70
7.70
7.69
7.26
3.67
3.66
3.65
1.68
1.68
1.67
1.66
1.65
1.63
1.60
1.60
1.59
1.58
1.57
1.50
1.49
1.47
1.46
1.45
0.92
0.91
0.91
0.90
0.55
2.00
2.02
2.09
2.15
2.16
2.16
2.12
2.11
f1 (ppm)

^{13}C NMR of **11**

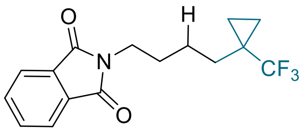

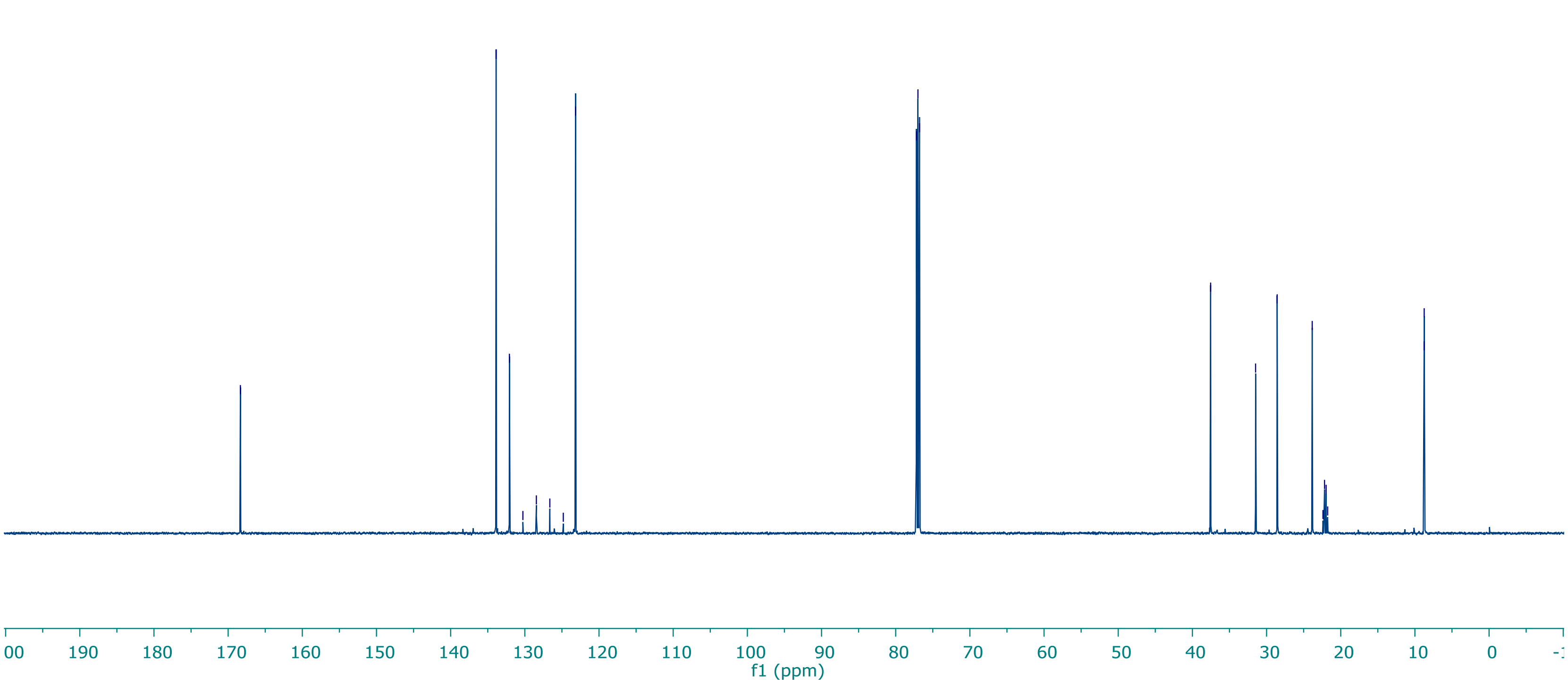
168.35
133.87
132.08
130.26
128.45
126.63
124.81
123.17
77.21 CDCl3
77.00 CDCl3
76.79 CDCl3
37.54
31.48
28.57
23.85
22.40
22.18
21.97
21.76
8.76
8.75
00 190 180 170 160 150 140 130 120 110 100 90 80 70 60 50 40 30 20 10 0
f1 (ppm)

^{19}F NMR of **11**

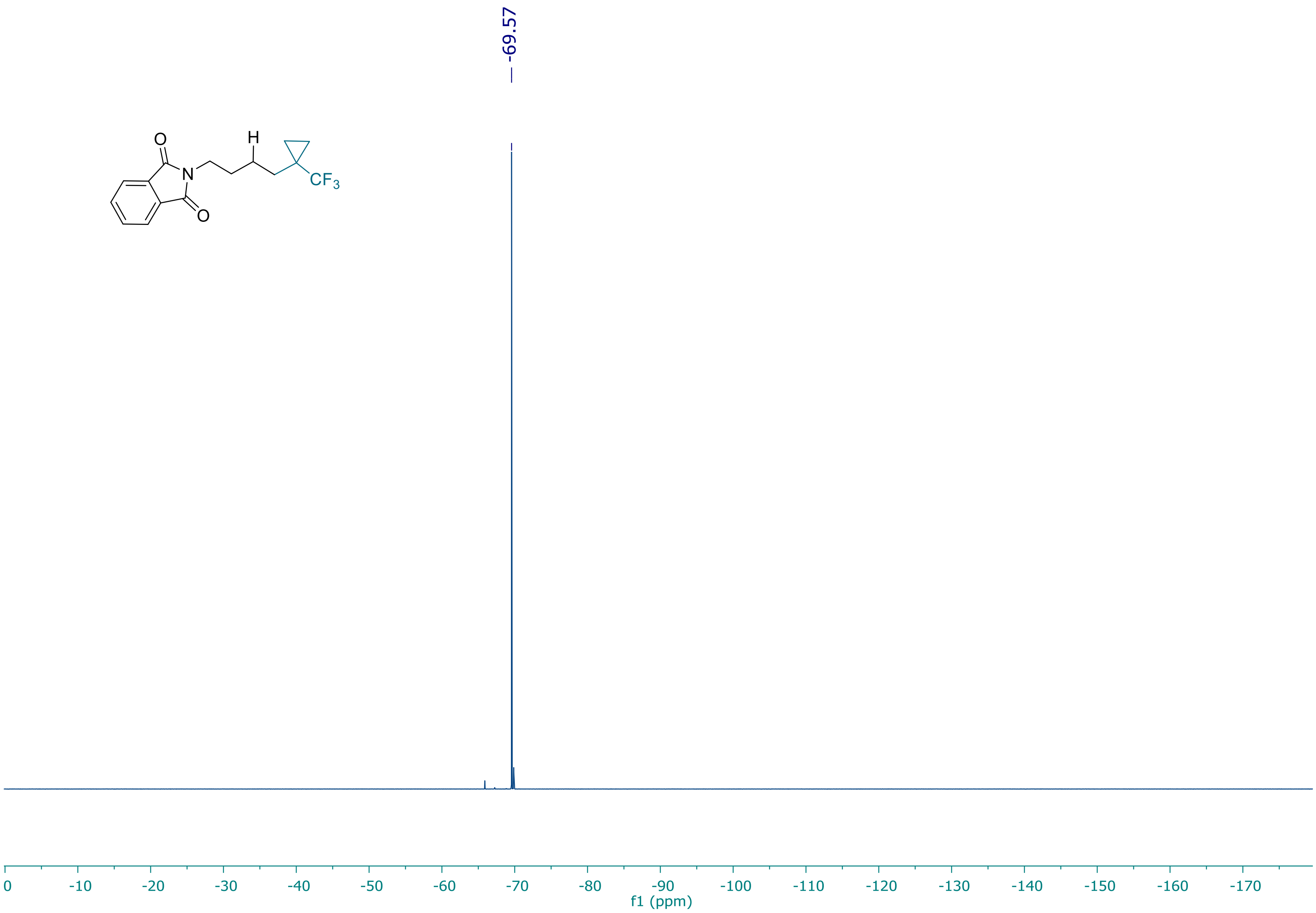

-69.57
O
H
N
CF3
O
0
-10
-20
-30
-40
-50
-60
-70
-80
-90
-100
-110
-120
-130
-140
-150
-160
-170
f1 (ppm)

^{1}H NMR of **12**

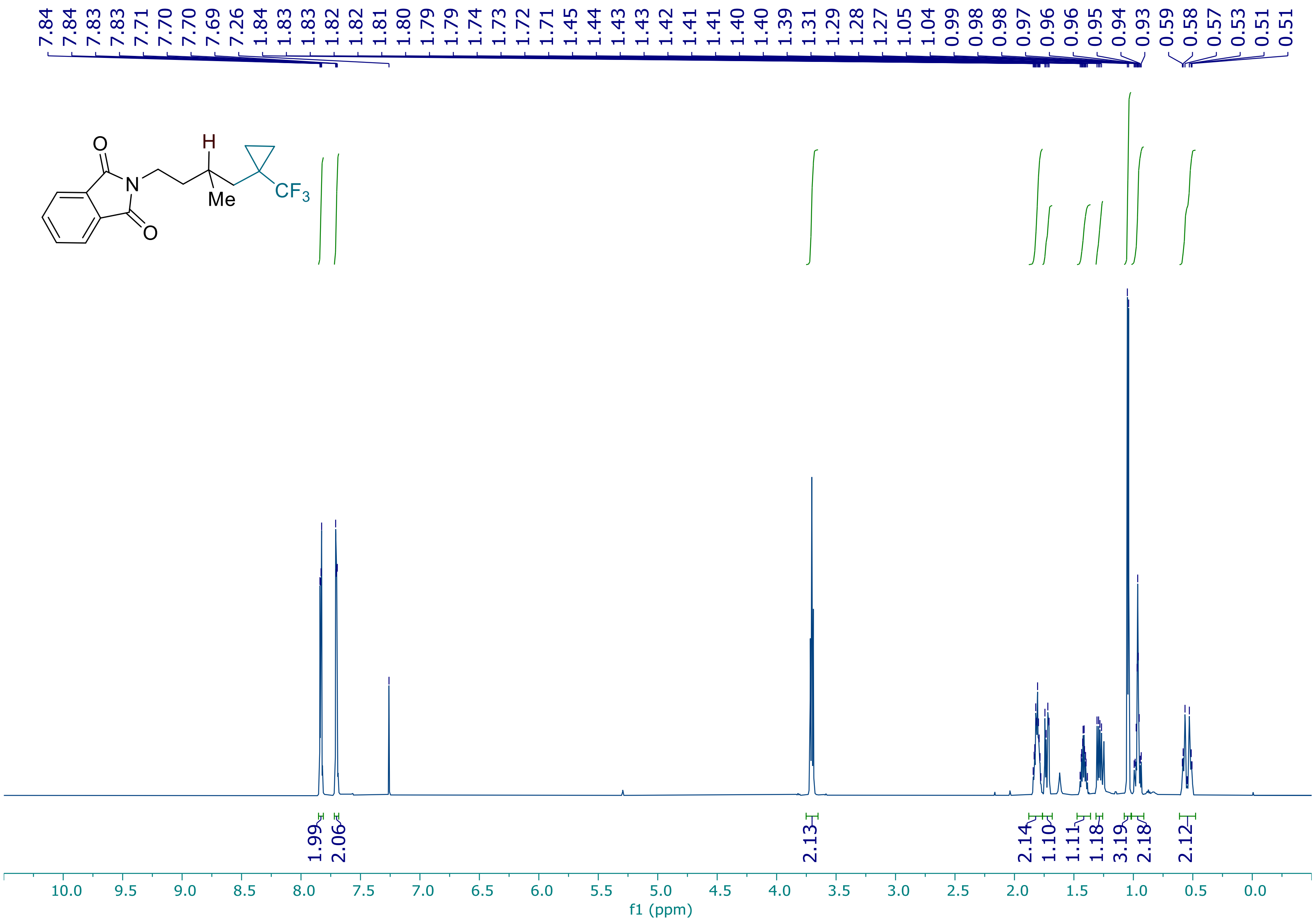

7.84 7.84 7.83 7.83 7.71 7.70 7.70 7.69 7.26 1.84 1.83 1.83 1.82 1.82 1.81 1.80 1.79 1.79 1.74 1.73 1.72 1.71 1.45 1.44 1.43 1.43 1.42 1.41 1.41 1.40 1.40 1.39 1.31 1.29 1.28 1.27 1.05 1.04 0.99 0.98 0.98 0.97 0.96 0.96 0.95 0.94 0.93 0.59 0.58 0.57 0.53 0.51 0.51
O
N
H
Me
CF_3
1.99
2.06
2.13
2.14
1.10
1.11
1.18
3.19
2.18
2.12
10.0 9.5 9.0 8.5 8.0 7.5 7.0 6.5 6.0 5.5 5.0 4.5 4.0 3.5 3.0 2.5 2.0 1.5 1.0 0.5 0.0
f1 (ppm)

^{13}C NMR of **12**

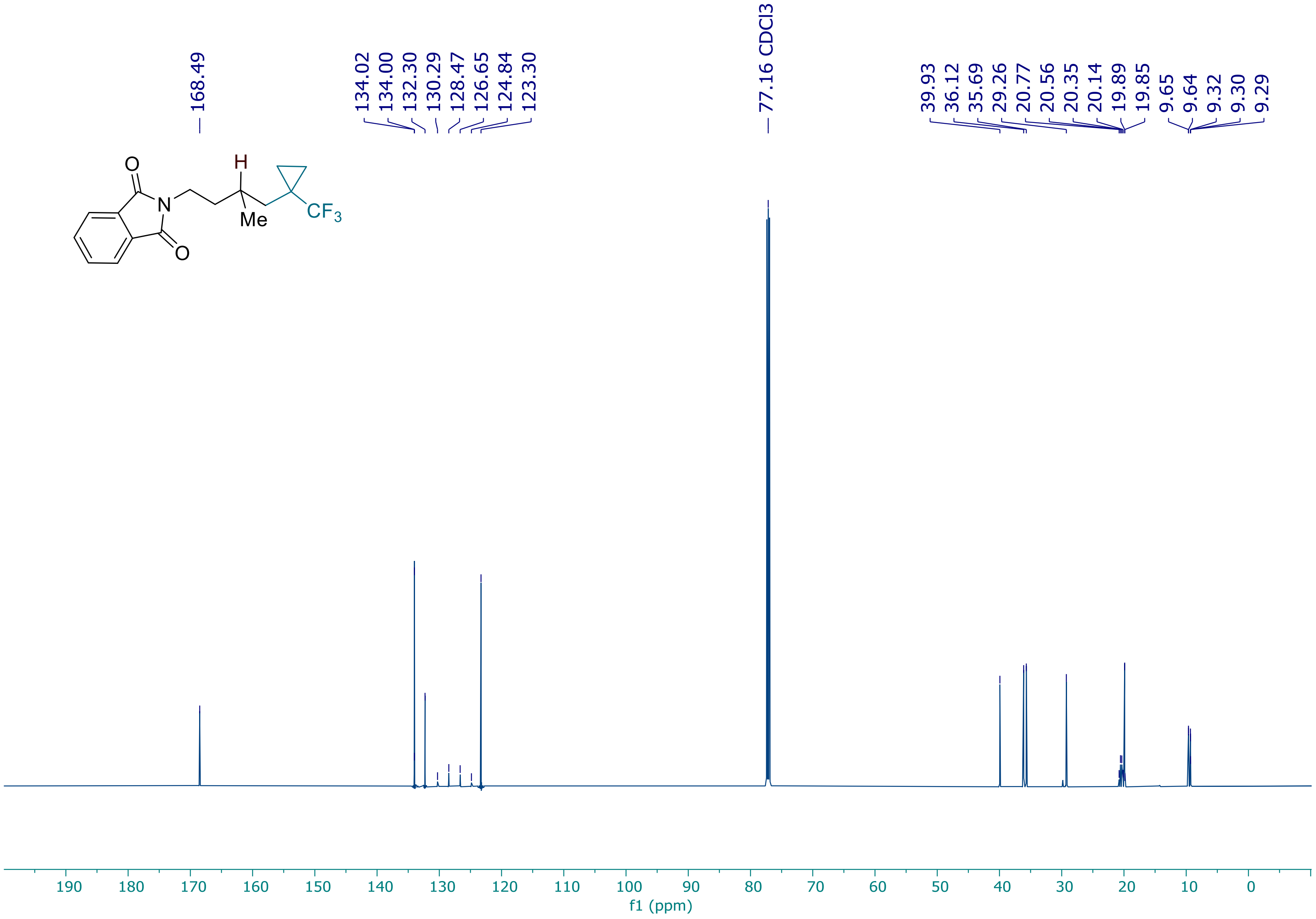

168.49
134.02
134.00
132.30
130.29
128.47
126.65
124.84
123.30
77.16 CDCl3
39.93
36.12
35.69
29.26
20.77
20.56
20.35
20.14
19.89
19.85
9.65
9.64
9.32
9.30
9.29
O
N
H
Me
CF3
190
180
170
160
150
140
130
120
110
100
90
80
70
60
50
40
30
20
10
0
f1 (ppm)

^{19}F NMR of **12**

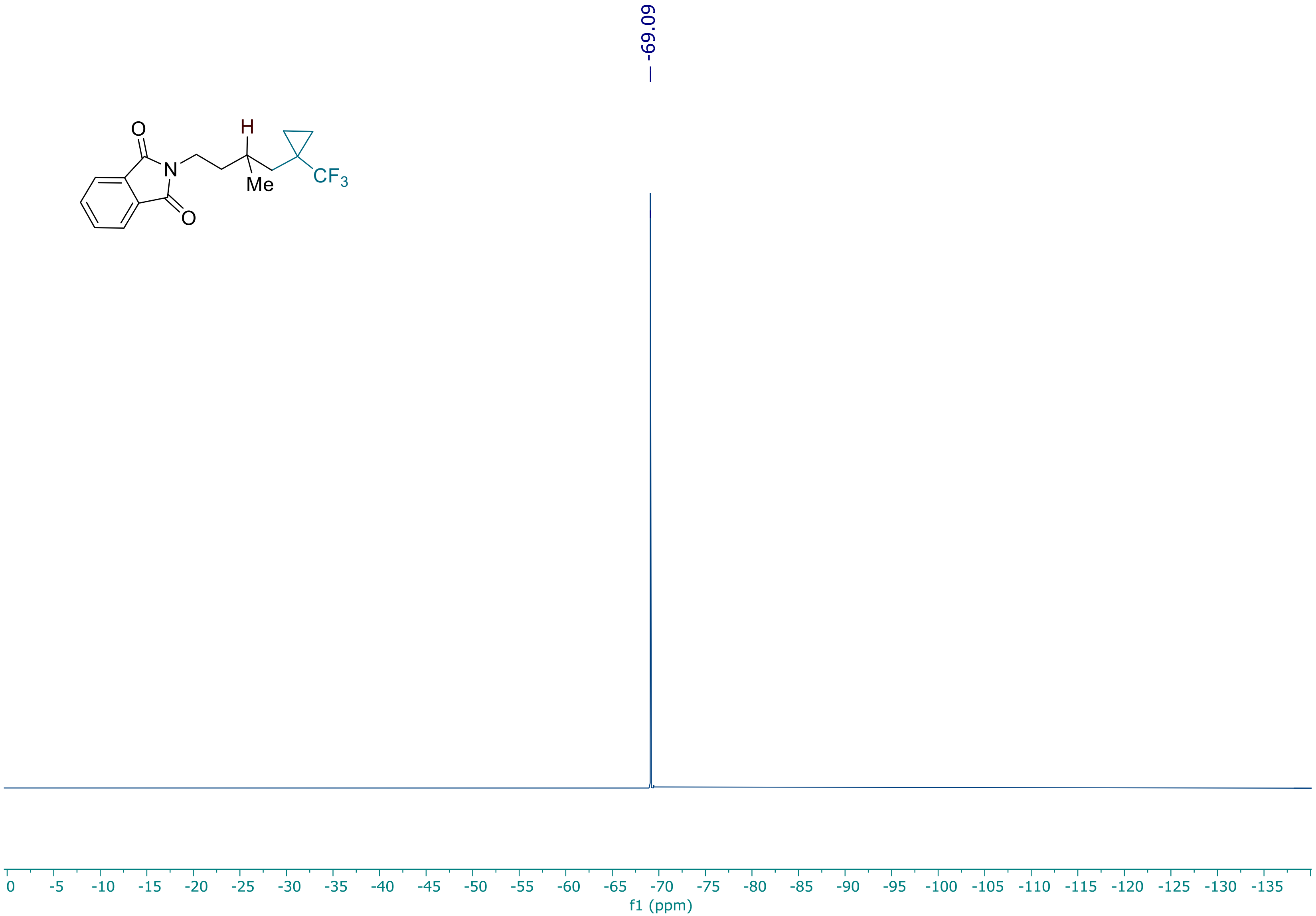
O
N
H
Me
CF3
O
-69.09
0
-5
-10
-15
-20
-25
-30
-35
-40
-45
-50
-55
-60
-65
-70
-75
-80
-85
-90
-95
-100
-105
-110
-115
-120
-125
-130
-135
f1 (ppm)

^{1}H NMR of **13**

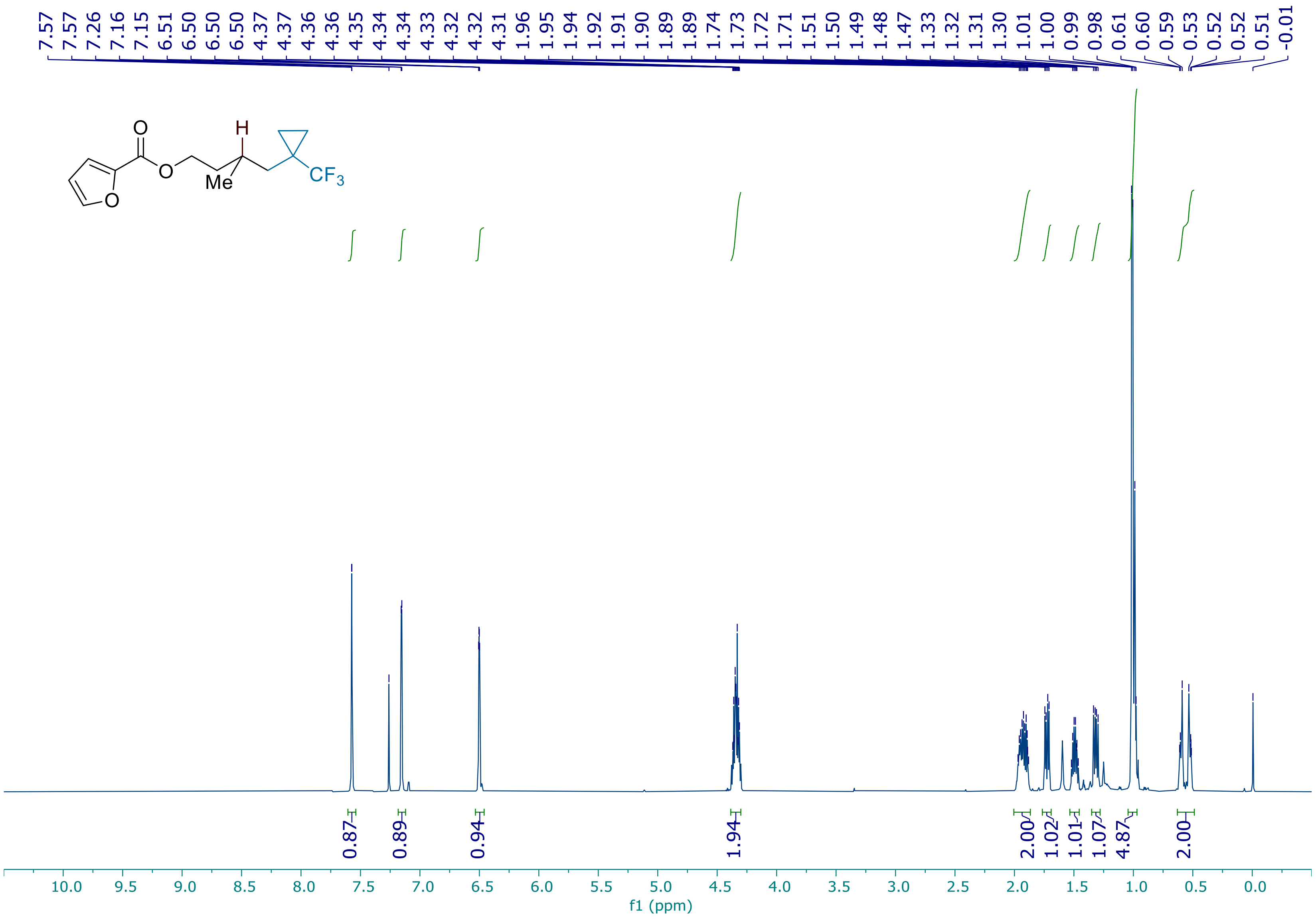

^{13}C NMR of **13**

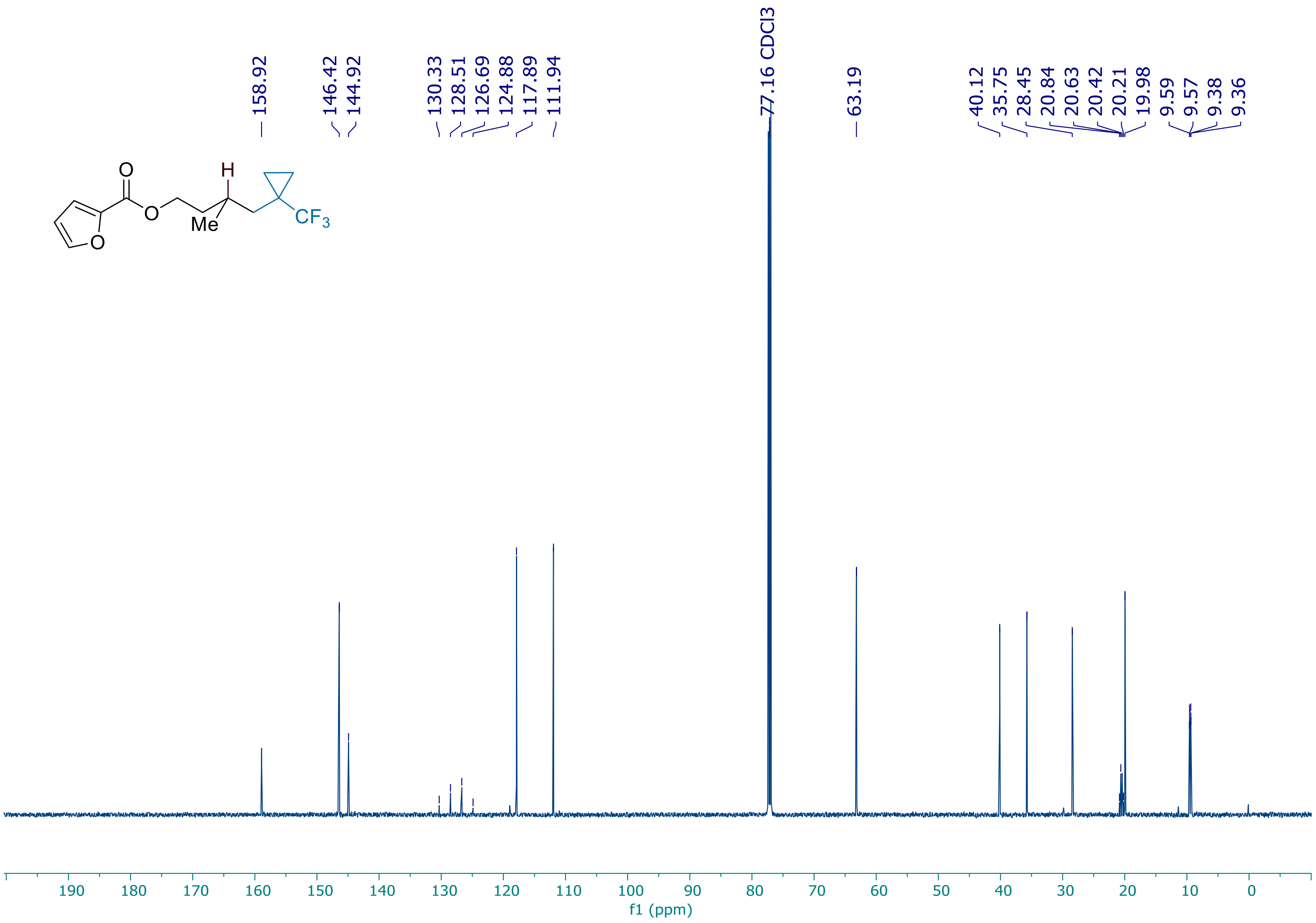

158.92
146.42
144.92
130.33
128.51
126.69
124.88
117.89
111.94
77.16 CDCl3
63.19
40.12
35.75
28.45
20.84
20.63
20.42
20.21
19.98
9.59
9.57
9.38
9.36
190 180 170 160 150 140 130 120 110 100 90 80 70 60 50 40 30 20 10 0
f1 (ppm)

^{19}F NMR of **13**

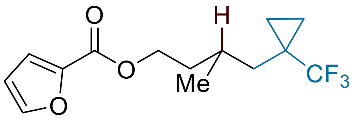
O
O
H
Me
CF3
O

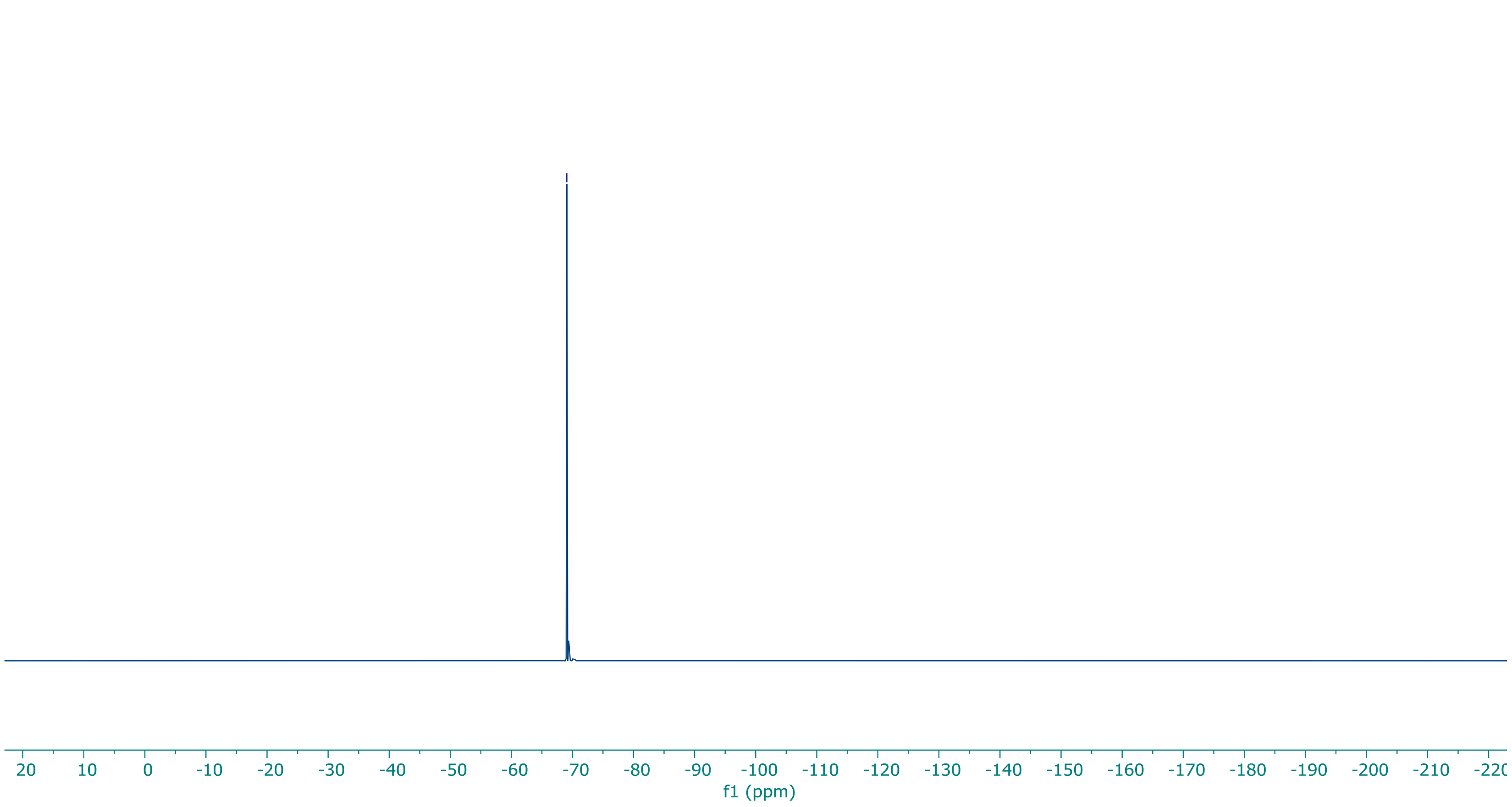
-69.08
20
10
0
-10
-20
-30
-40
-50
-60
-70
-80
-90
-100
-110
-120
-130
-140
-150
-160
-170
-180
-190
-200
-210
f1 (ppm)

^{1}H NMR of **14**

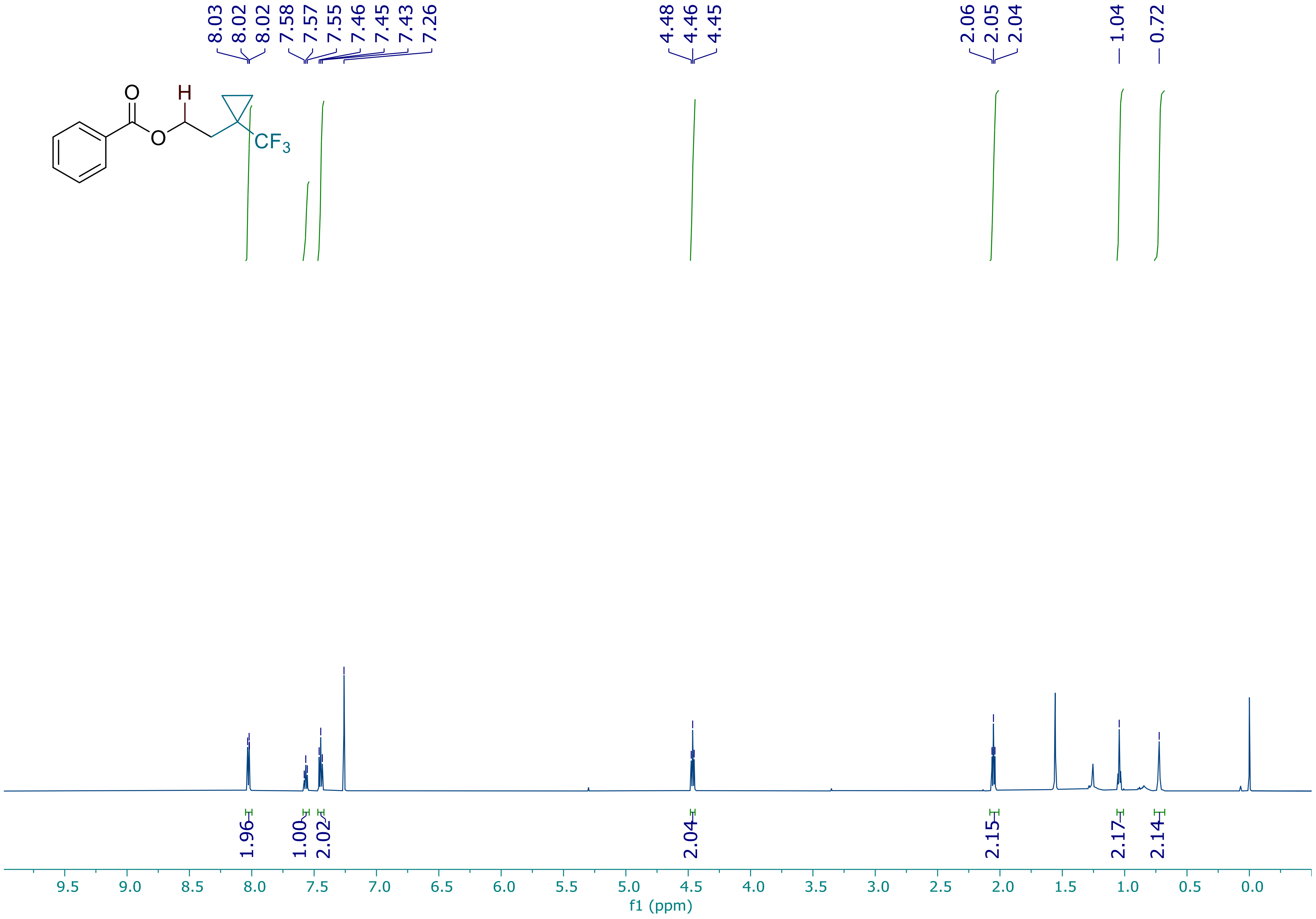
8.03
8.02
8.02
7.58
7.57
7.55
7.46
7.45
7.43
7.26
4.48
4.46
4.45
2.06
2.05
2.04
1.04
0.72
O
H
O
CF3
1.96
1.00
2.02
2.04
2.15
2.17
2.14
9.5
9.0
8.5
8.0
7.5
7.0
6.5
6.0
5.5
5.0
4.5
4.0
3.5
3.0
2.5
2.0
1.5
1.0
0.5
0.0
f1 (ppm)

^{13}C NMR of **14**

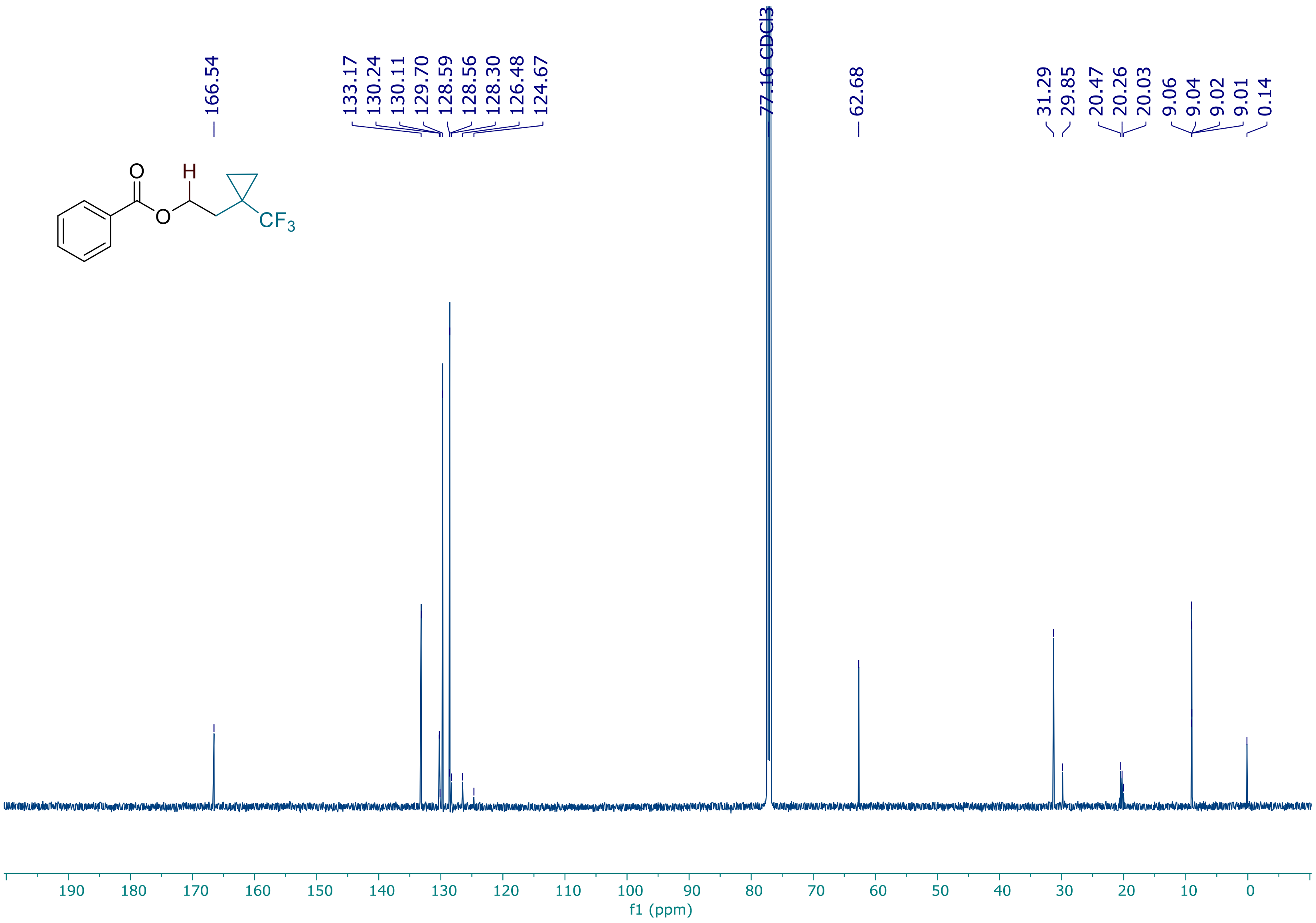

166.54
133.17
130.24
130.11
129.70
128.59
128.56
128.30
126.48
124.67
77.16 CDCl3
62.68
31.29
29.85
20.47
20.26
20.03
9.06
9.04
9.02
9.01
0.14
O
H
O
CF3
190
180
170
160
150
140
130
120
110
100
90
80
70
60
50
40
30
20
10
0
f1 (ppm)

^{19}F NMR of **14**

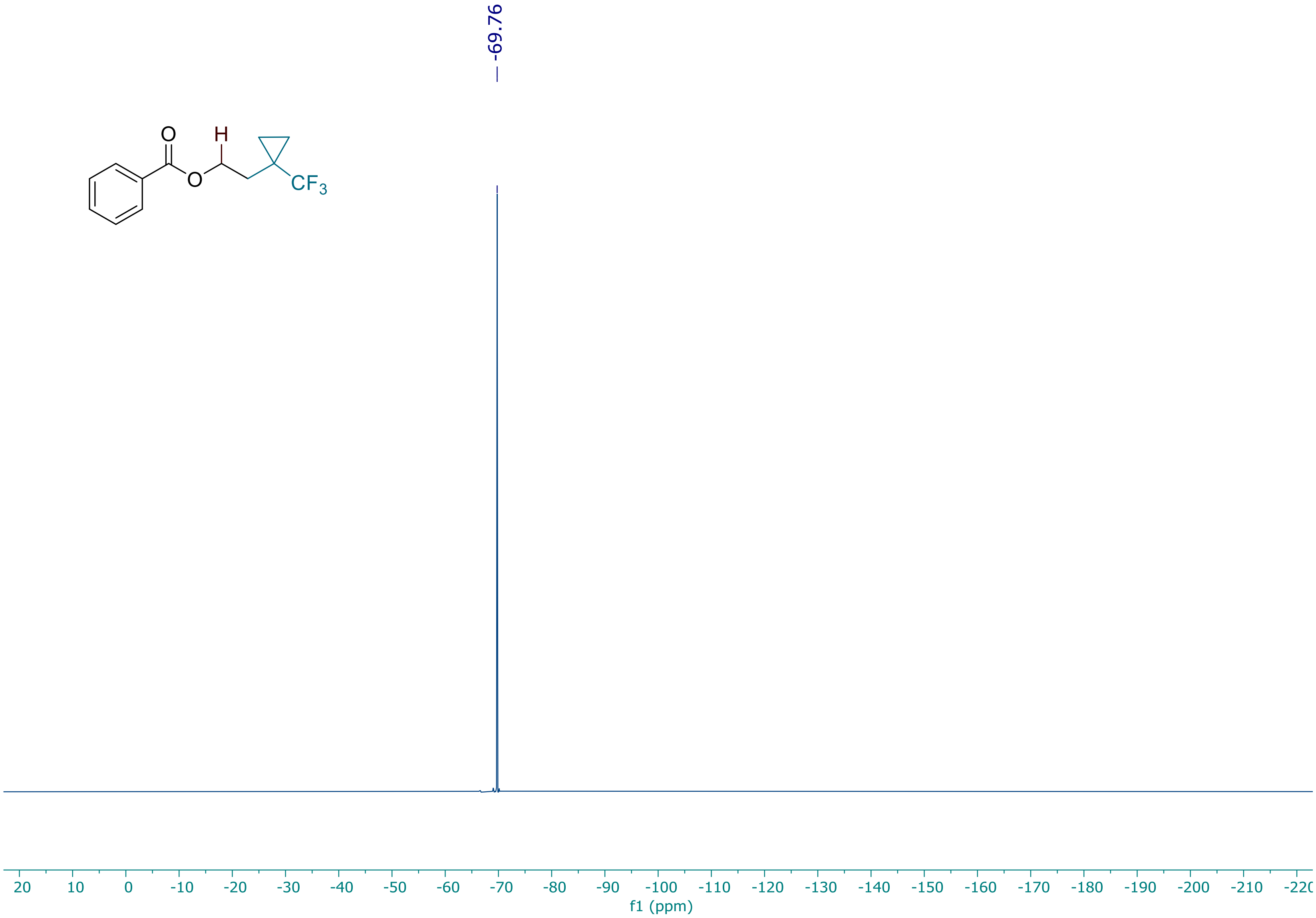

-69.76
O
H
O
CF3
20
10
0
-10
-20
-30
-40
-50
-60
-70
-80
-90
-100
-110
-120
-130
-140
-150
-160
-170
-180
-190
-200
-210
-220
f1 (ppm)

^1H NMR of **15**

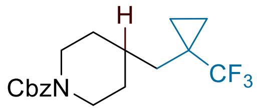

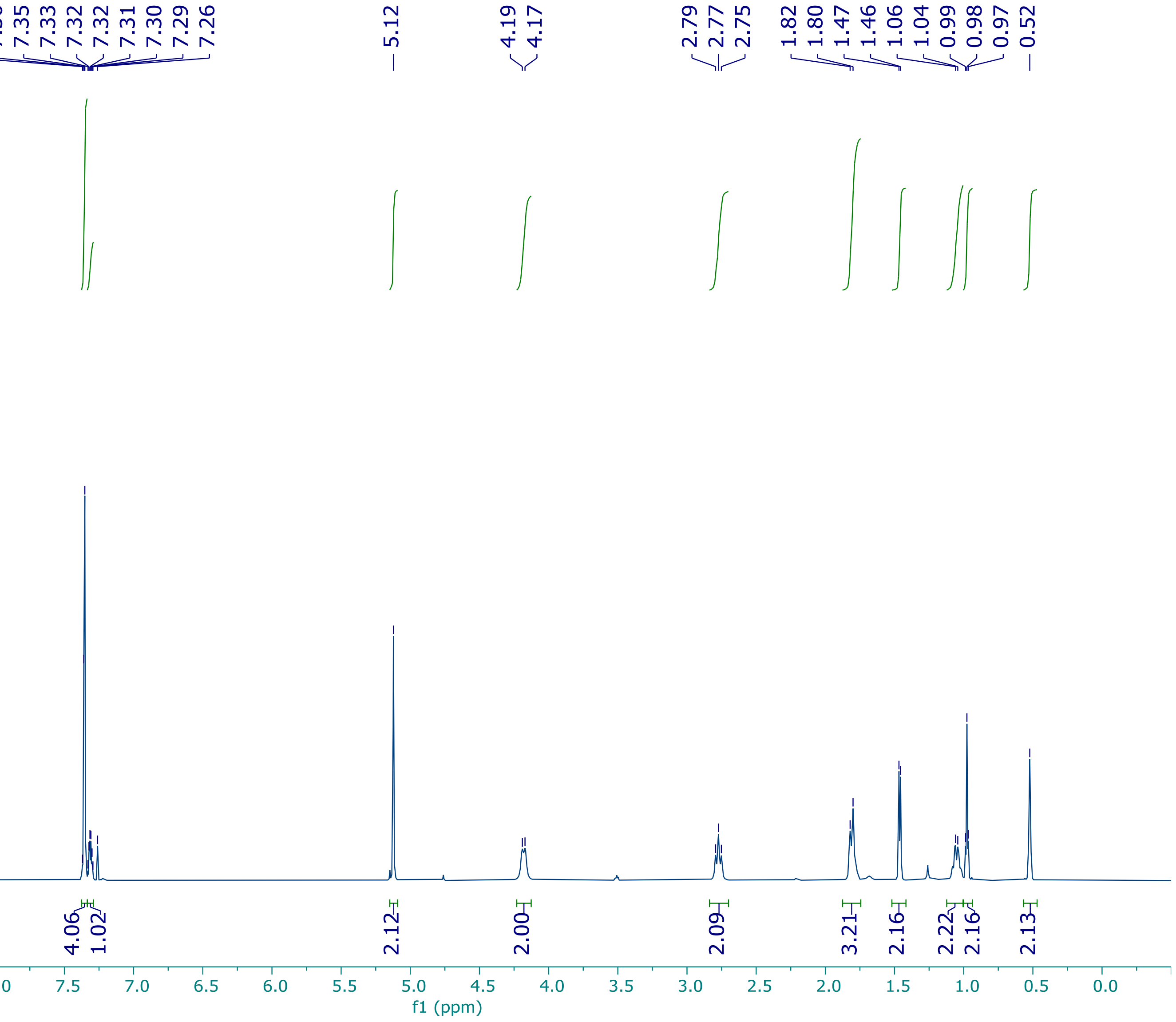
7.37
7.36
7.35
7.33
7.32
7.32
7.31
7.30
7.29
7.26
5.12
4.19
4.17
2.79
2.77
2.75
1.82
1.80
1.47
1.46
1.06
1.04
0.99
0.98
0.97
0.52
4.06
1.02
2.12
2.00
2.09
3.21
2.16
2.22
2.16
2.13
9.5
9.0
8.5
8.0
7.5
7.0
6.5
6.0
5.5
5.0
4.5
4.0
3.5
3.0
2.5
2.0
1.5
1.0
0.5
0.0
f1 (ppm)

^{13}C NMR of **15**

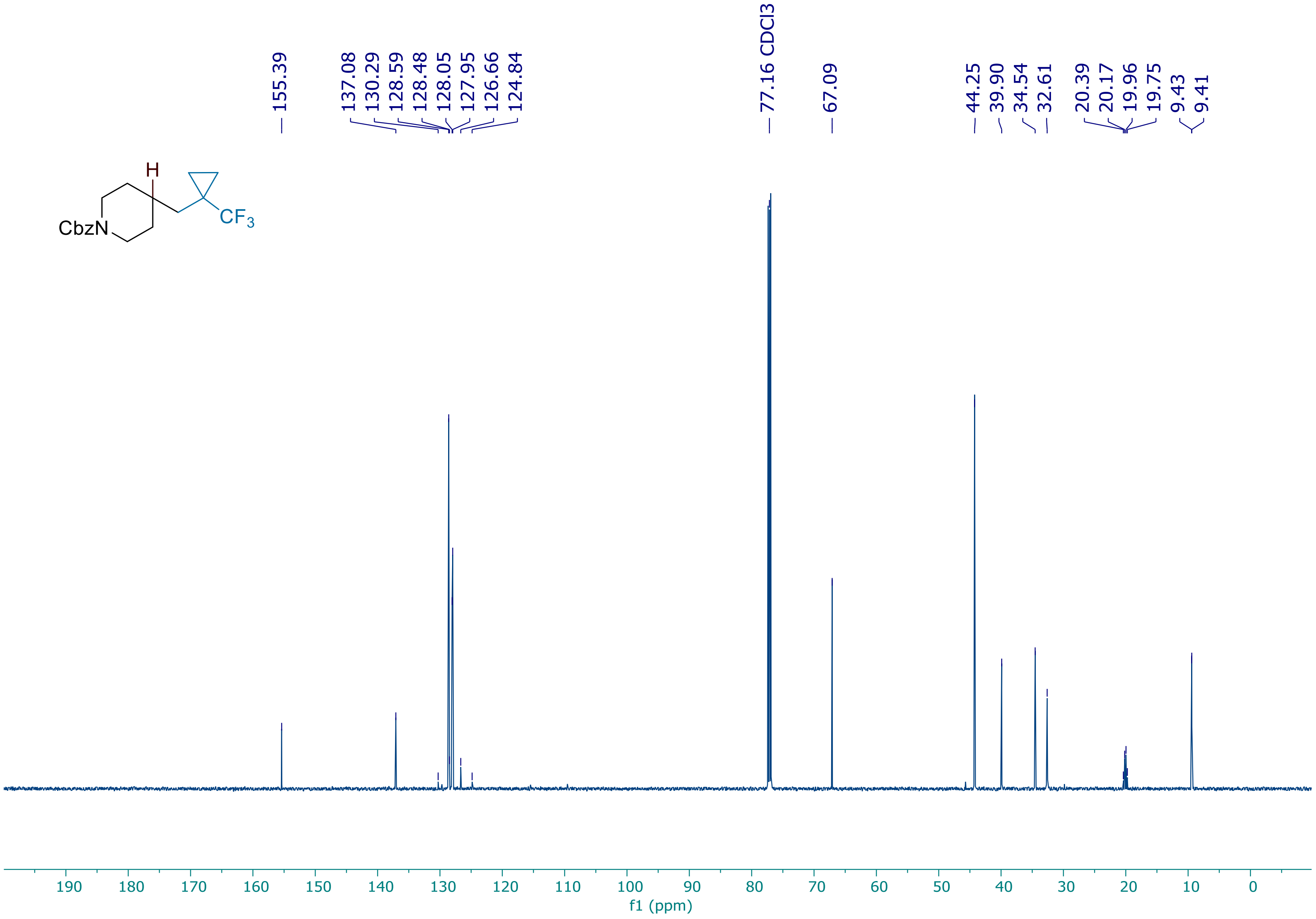

155.39
137.08
130.29
128.59
128.48
128.05
127.95
126.66
124.84
77.16 CDCl3
67.09
44.25
39.90
34.54
32.61
20.39
20.17
19.96
19.75
9.43
9.41
H
CbzN
CF3
190
180
170
160
150
140
130
120
110
100
90
80
70
60
50
40
30
20
10
0
f1 (ppm)

^{19}F NMR of **15**

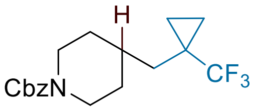

H
CbzN
CF3

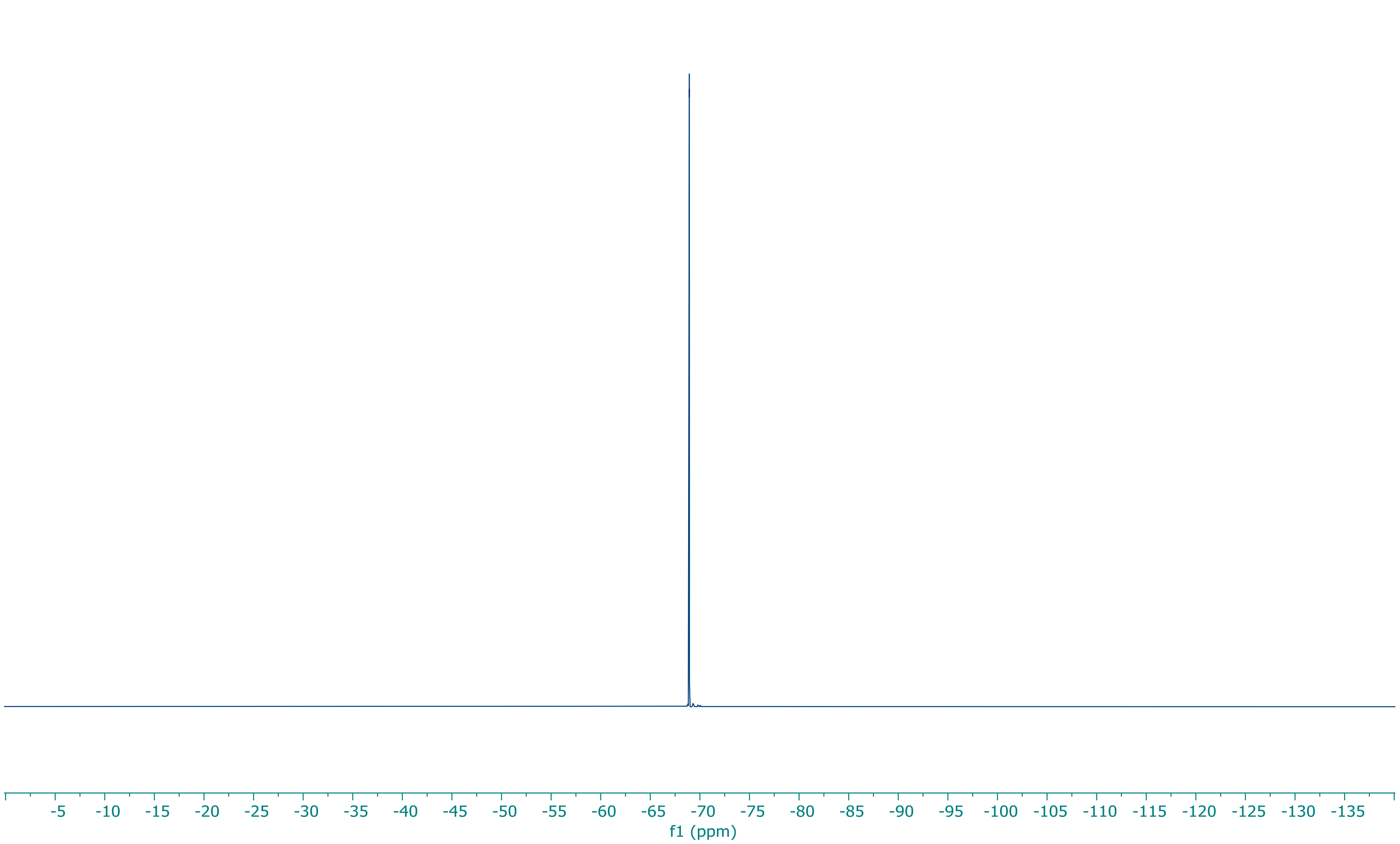

-68.94
-5 -10 -15 -20 -25 -30 -35 -40 -45 -50 -55 -60 -65 -70 -75 -80 -85 -90 -95 -100 -105 -110 -115 -120 -125 -130 -135
f1 (ppm)

^{1}H NMR of **16**

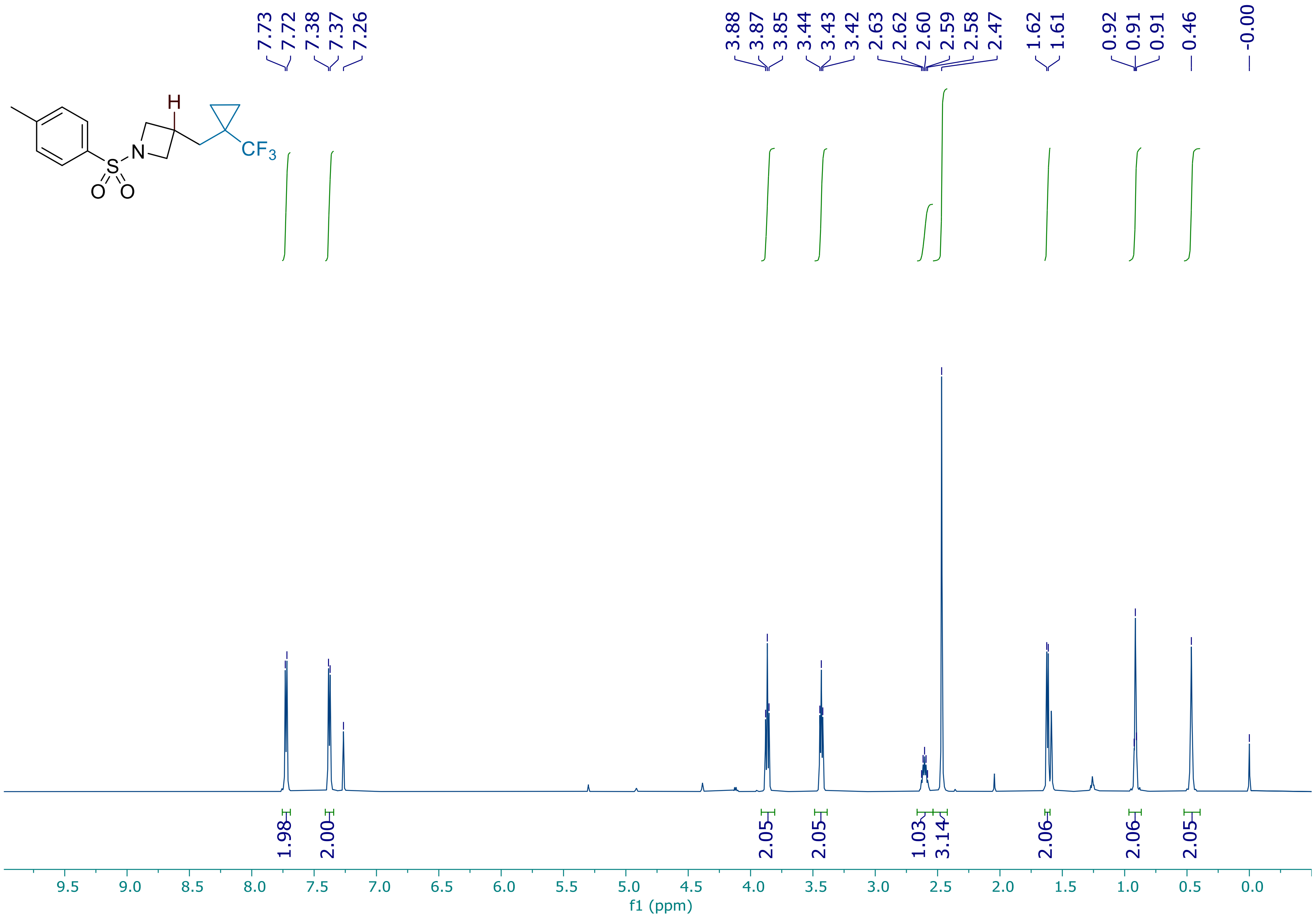

7.73
7.72
7.38
7.37
7.26
3.88
3.87
3.85
3.44
3.43
3.42
2.63
2.62
2.60
2.59
2.58
2.47
1.62
1.61
0.92
0.91
0.91
0.46
-0.00
1.98
2.00
2.05
2.05
1.03
3.14
2.06
2.06
2.05
f1 (ppm)

^{13}C NMR of **16**

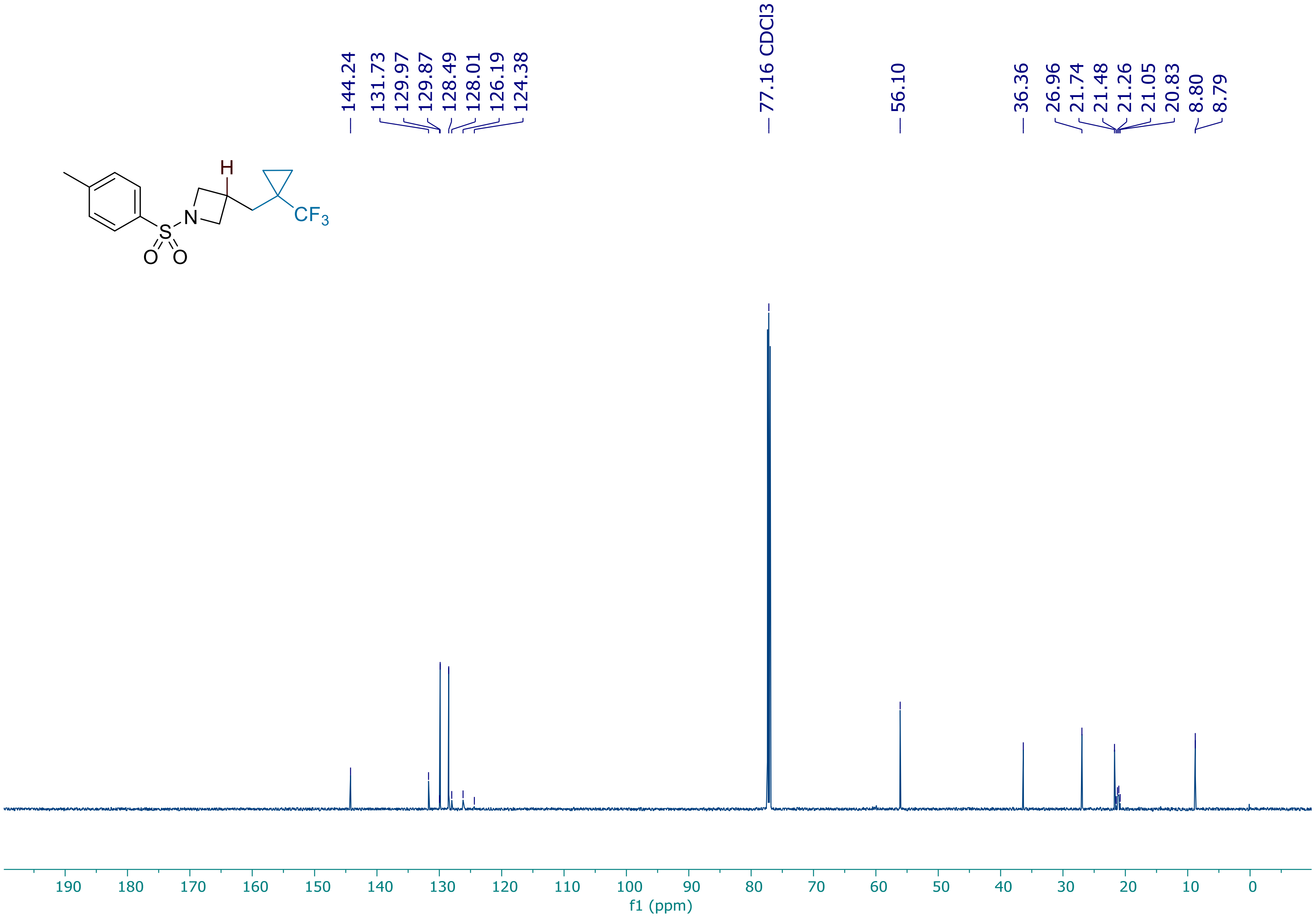

144.24
131.73
129.97
129.87
128.49
128.01
126.19
124.38
77.16 CDCl3
56.10
36.36
26.96
21.74
21.48
21.26
21.05
20.83
8.80
8.79
H
N
S
O
O
CF3
190
180
170
160
150
140
130
120
110
100
90
80
70
60
50
40
30
20
10
0
f1 (ppm)

^{19}F NMR of **16**

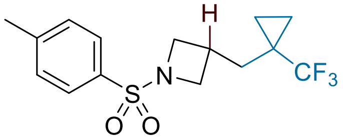

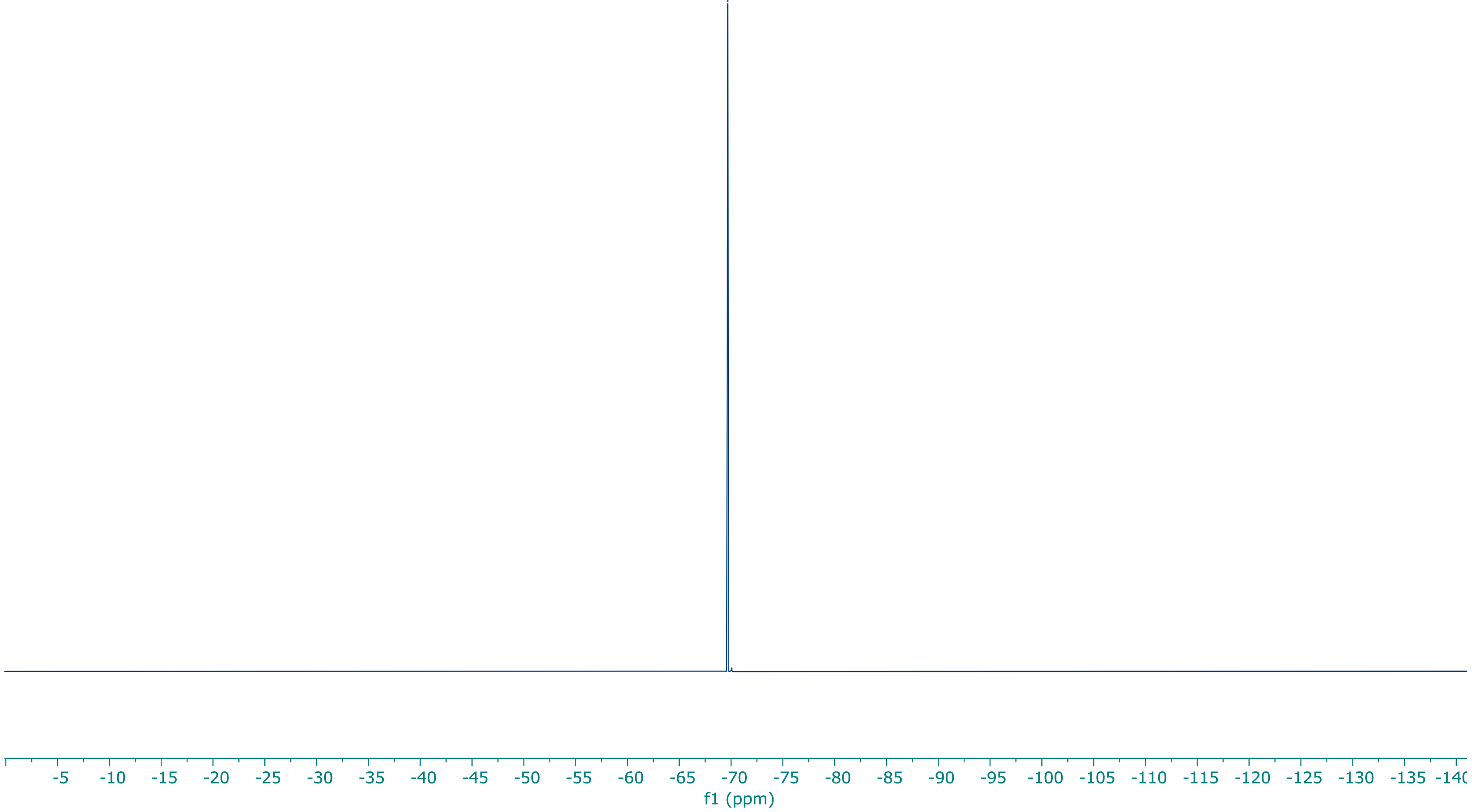
-69.69
-5
-10
-15
-20
-25
-30
-35
-40
-45
-50
-55
-60
-65
-70
-75
-80
-85
-90
-95
-100
-105
-110
-115
-120
-125
-130
-135
f1 (ppm)

^{1}H NMR of **17**

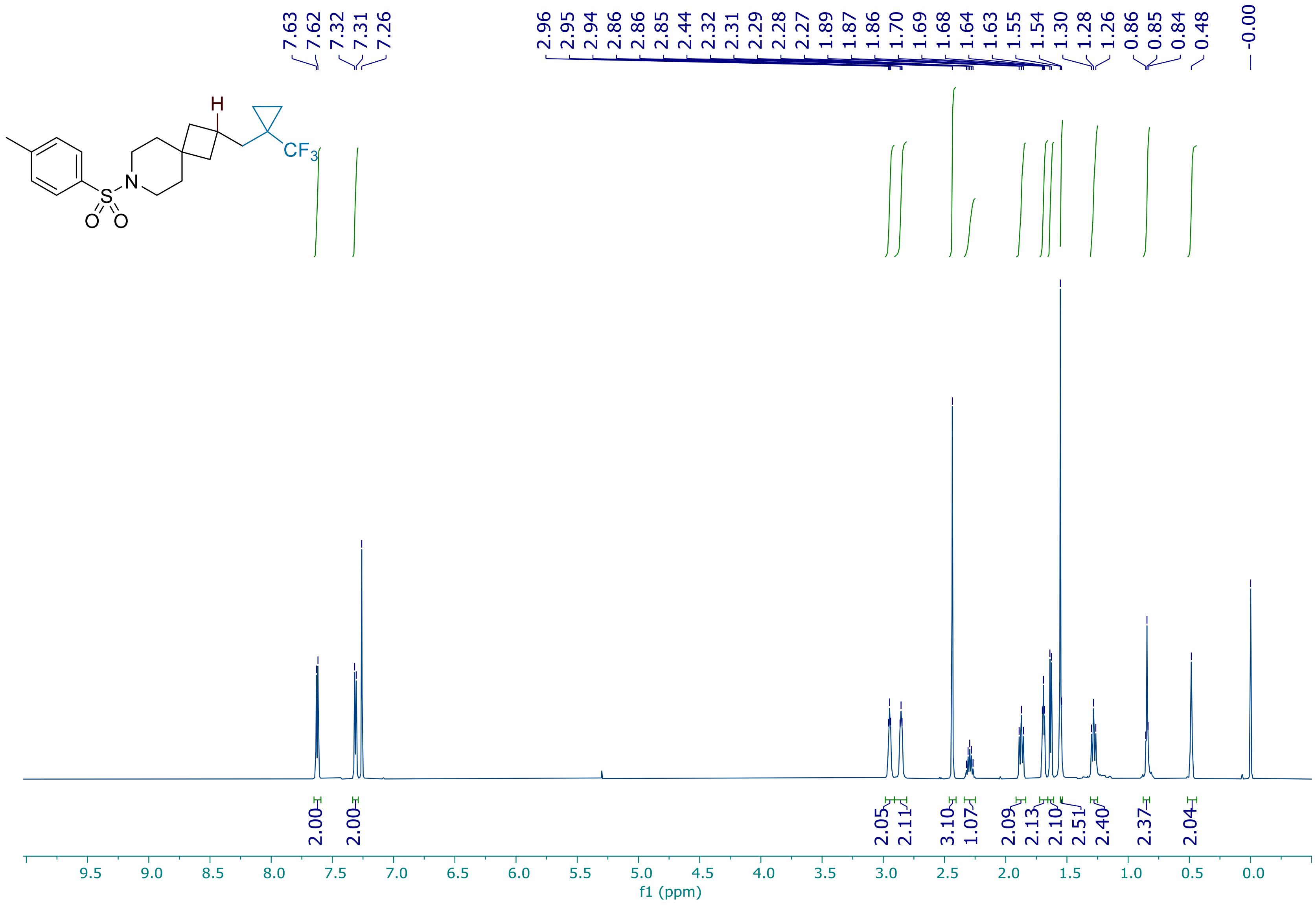

^{13}C NMR of **17**

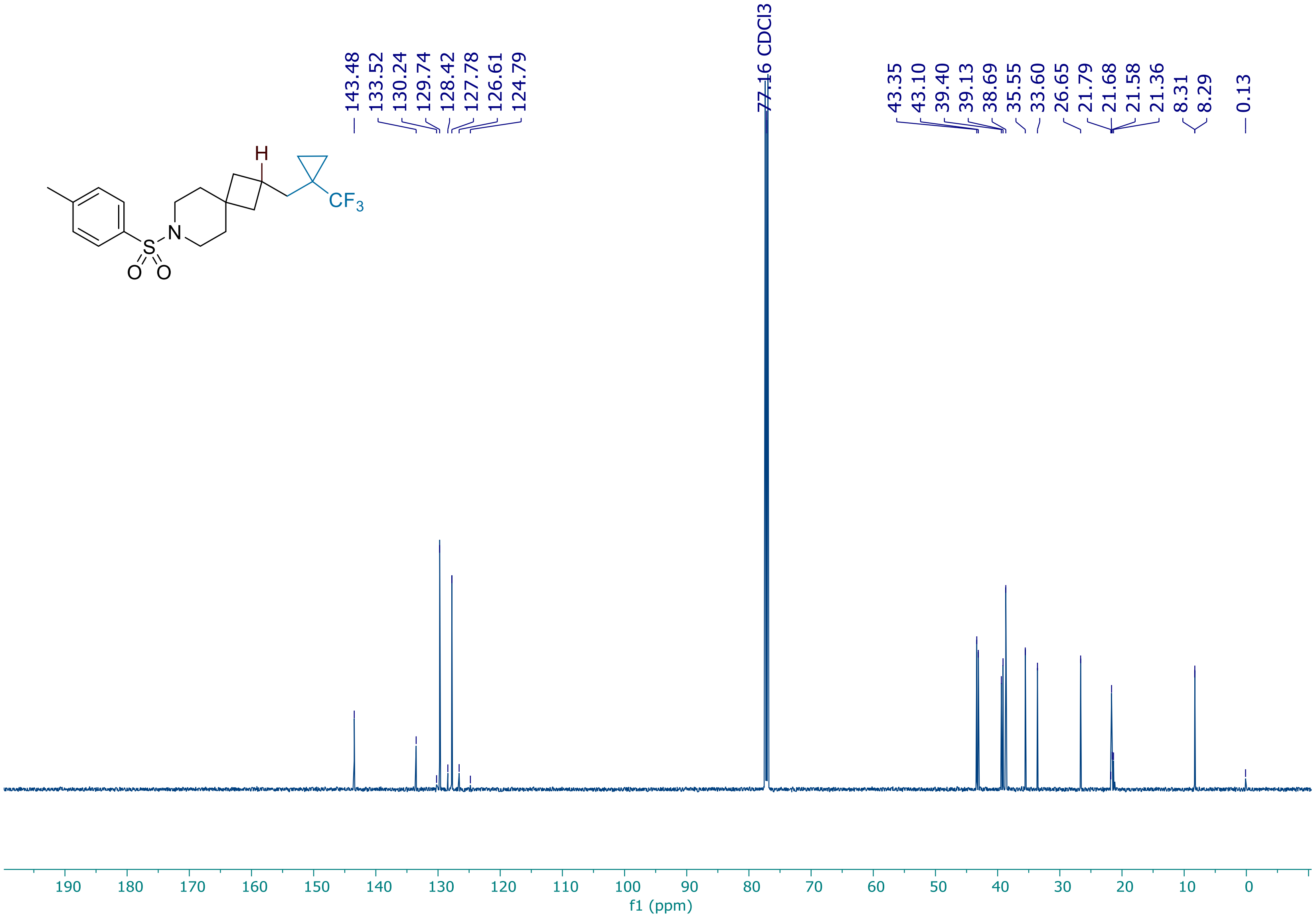

143.48
133.52
130.24
129.74
128.42
127.78
126.61
124.79
77.16 CDCl3
43.35
43.10
39.40
39.13
38.69
35.55
33.60
26.65
21.79
21.68
21.58
21.36
8.31
8.29
0.13
190 180 170 160 150 140 130 120 110 100 90 80 70 60 50 40 30 20 10 0
f1 (ppm)

^{19}F NMR of **17**

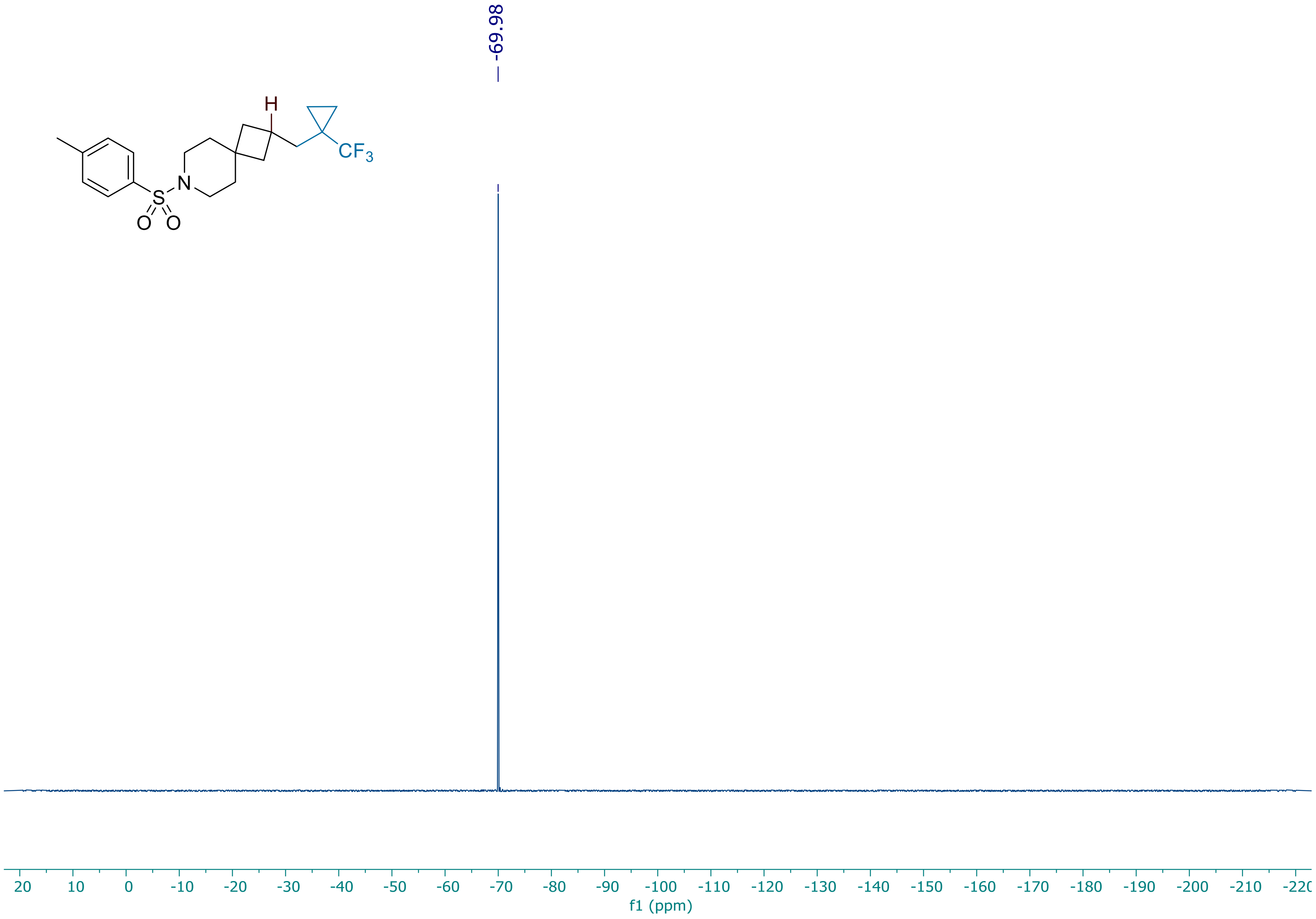
-69.98
H
CF3
N
S
O
O
20 10 0 -10 -20 -30 -40 -50 -60 -70 -80 -90 -100 -110 -120 -130 -140 -150 -160 -170 -180 -190 -200 -210 -220
f1 (ppm)

^{1}H NMR of **18**

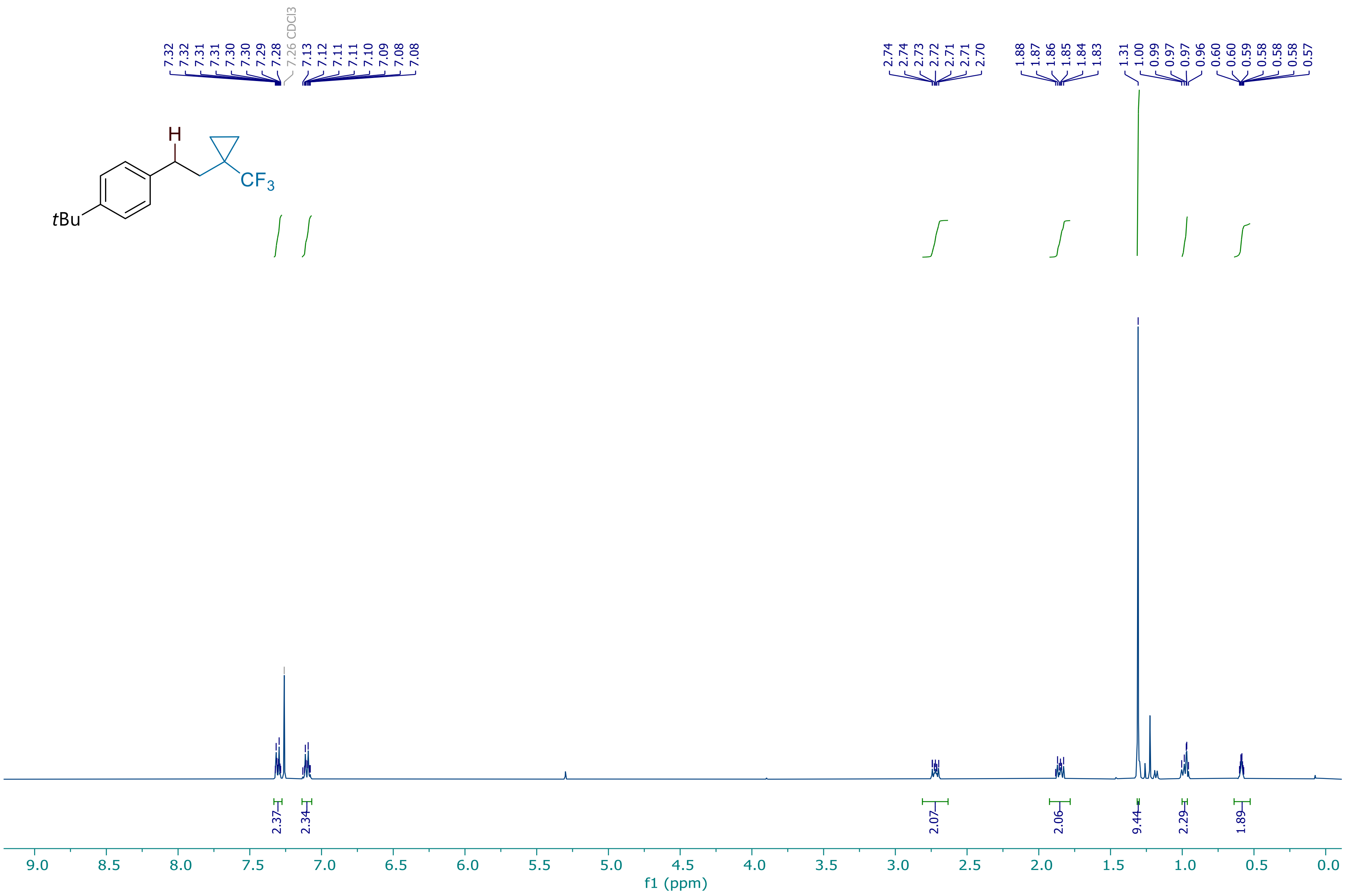

7.32
7.32
7.31
7.31
7.30
7.30
7.29
7.28
7.26 CDCl3
7.13
7.12
7.11
7.11
7.10
7.09
7.08
7.08
2.74
2.74
2.73
2.72
2.71
2.71
2.70
1.88
1.87
1.86
1.85
1.84
1.83
1.31
1.00
0.99
0.97
0.97
0.96
0.60
0.60
0.59
0.58
0.58
0.57
H
CF3
tBu
2.37
2.34
2.07
2.06
9.44
2.29
1.89
9.0
8.5
8.0
7.5
7.0
6.5
6.0
5.5
5.0
4.5
4.0
3.5
3.0
2.5
2.0
1.5
1.0
0.5
0.0
f1 (ppm)

^{13}C NMR of **18**

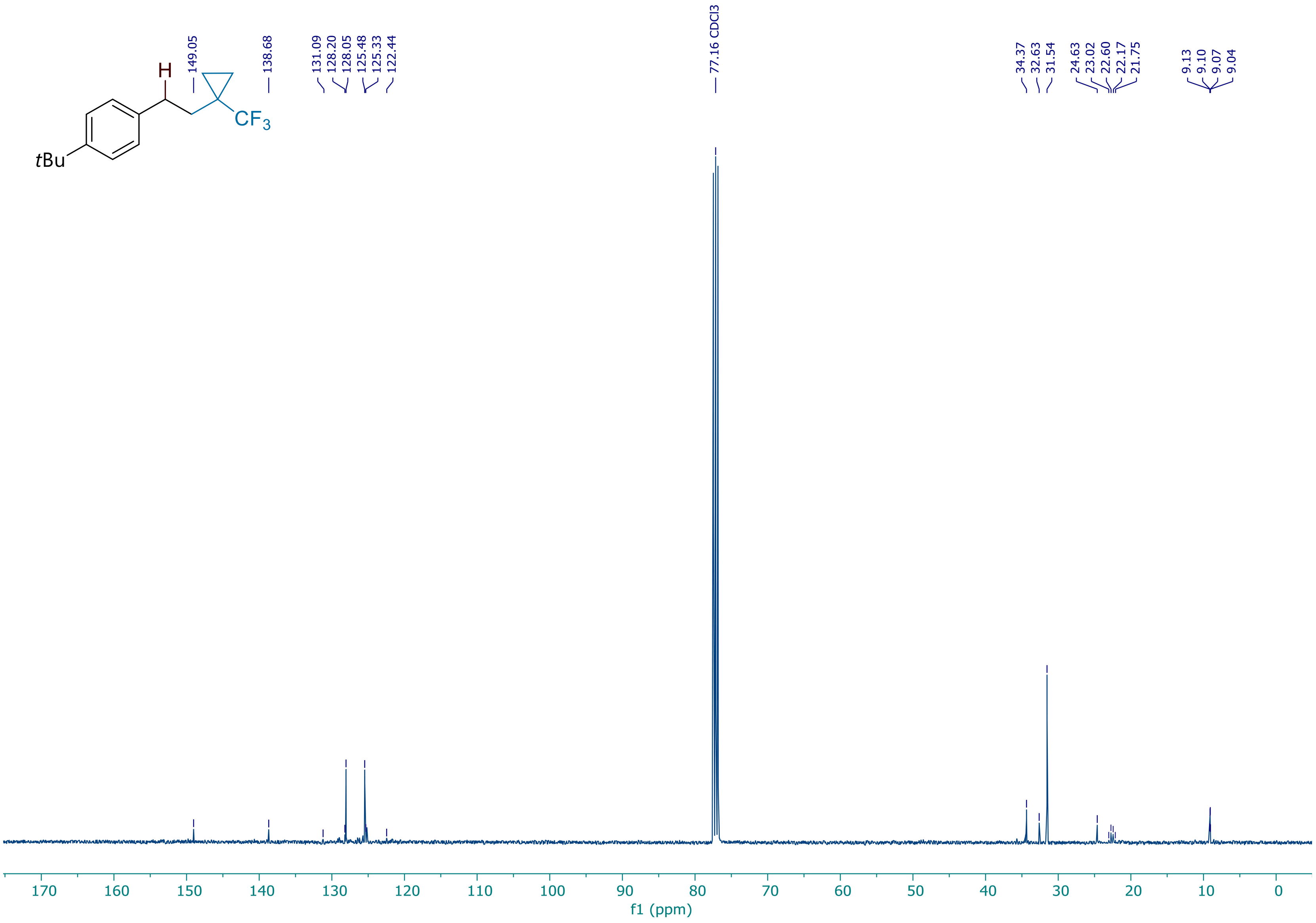
H
CF3
tBu
149.05
138.68
131.09
128.20
128.05
125.48
125.33
122.44
77.16 CDCl3
34.37
32.63
31.54
24.63
23.02
22.60
22.17
21.75
9.13
9.10
9.07
9.04
170 160 150 140 130 120 110 100 90 80 70 60 50 40 30 20 10 0
f1 (ppm)

^{19}F NMR of **18**

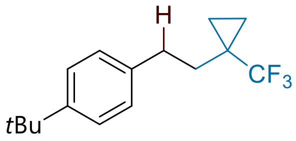

H
CF3
tBu

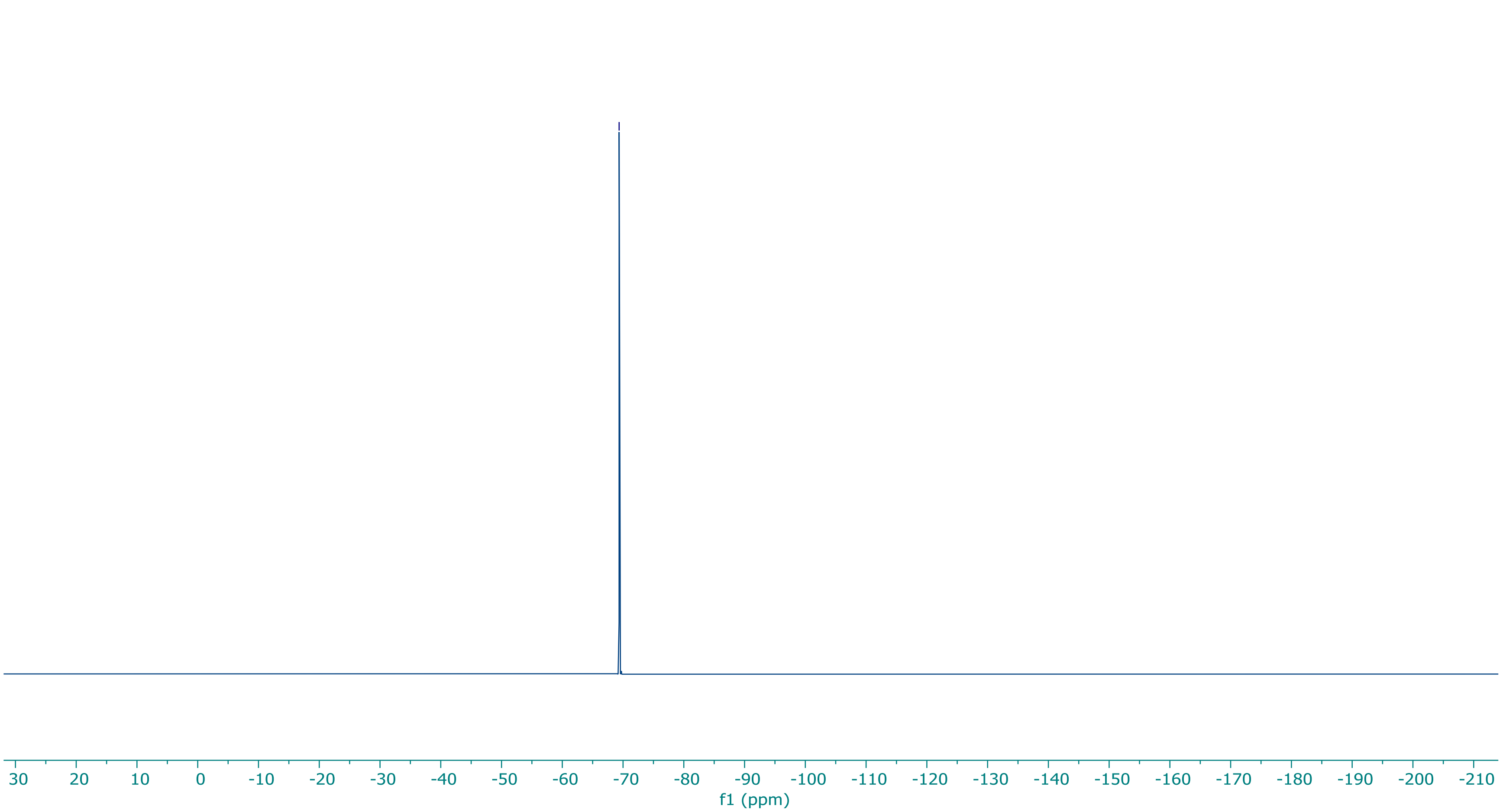

-69.35
30
20
10
0
-10
-20
-30
-40
-50
-60
-70
-80
-90
-100
-110
-120
-130
-140
-150
-160
-170
-180
-190
-200
-210
f1 (ppm)

^{1}H NMR of **19**

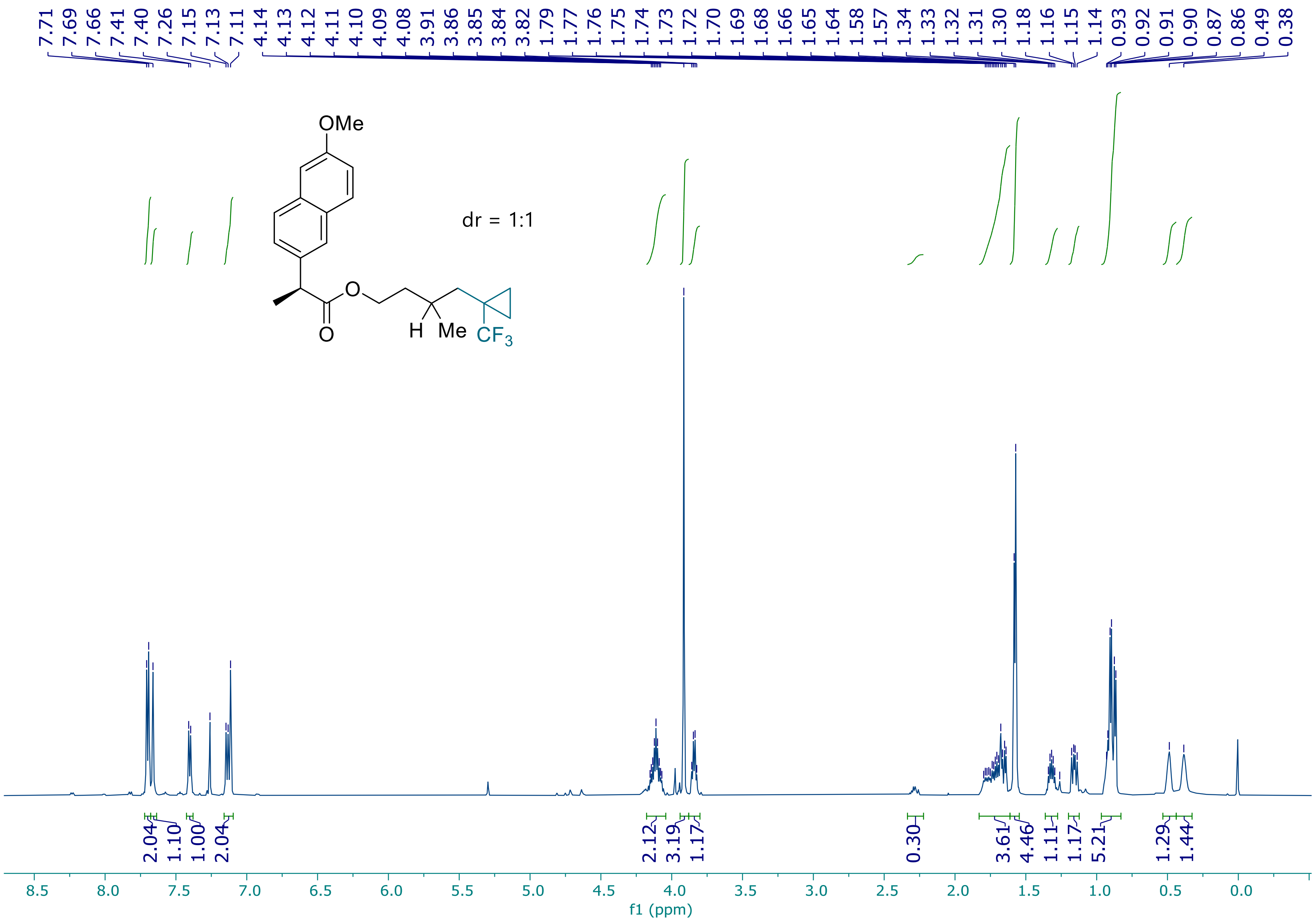

^{13}C NMR of **19**

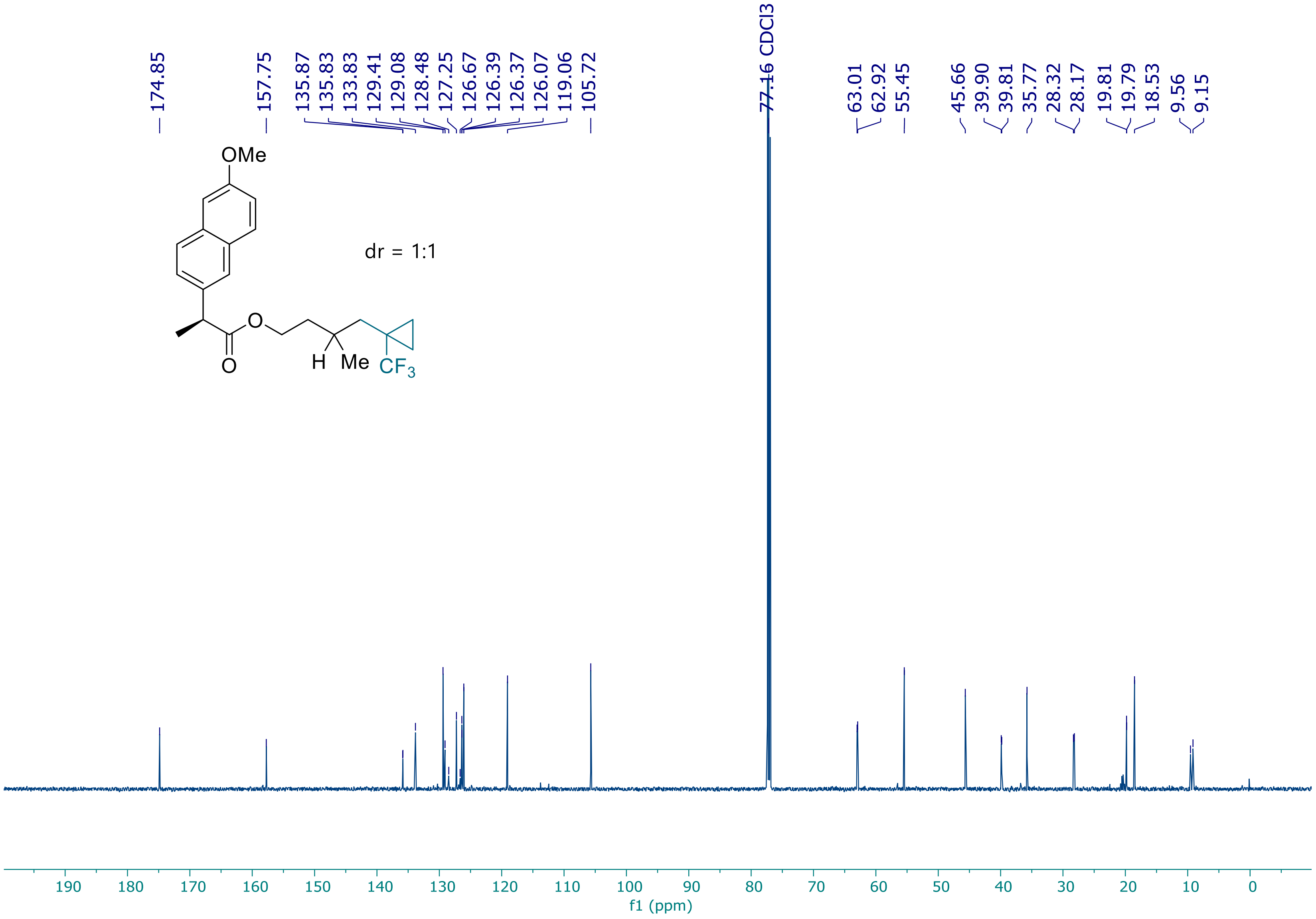

174.85
157.75
135.87
135.83
133.83
129.41
129.08
128.48
127.25
126.67
126.39
126.37
126.07
119.06
105.72
77.16 CDCl3
63.01
62.92
55.45
45.66
39.90
39.81
35.77
28.32
28.17
19.81
19.79
18.53
9.56
9.15
OMe
O
O
H
Me
CF3
dr = 1:1
190
180
170
160
150
140
130
120
110
100
90
80
70
60
50
40
30
20
10
0
f1 (ppm)

^{19}F NMR of **19**

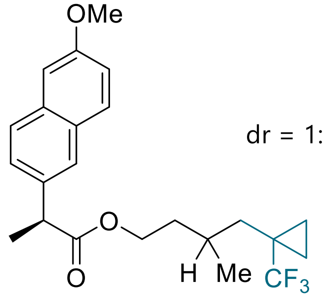

dr = 1:1

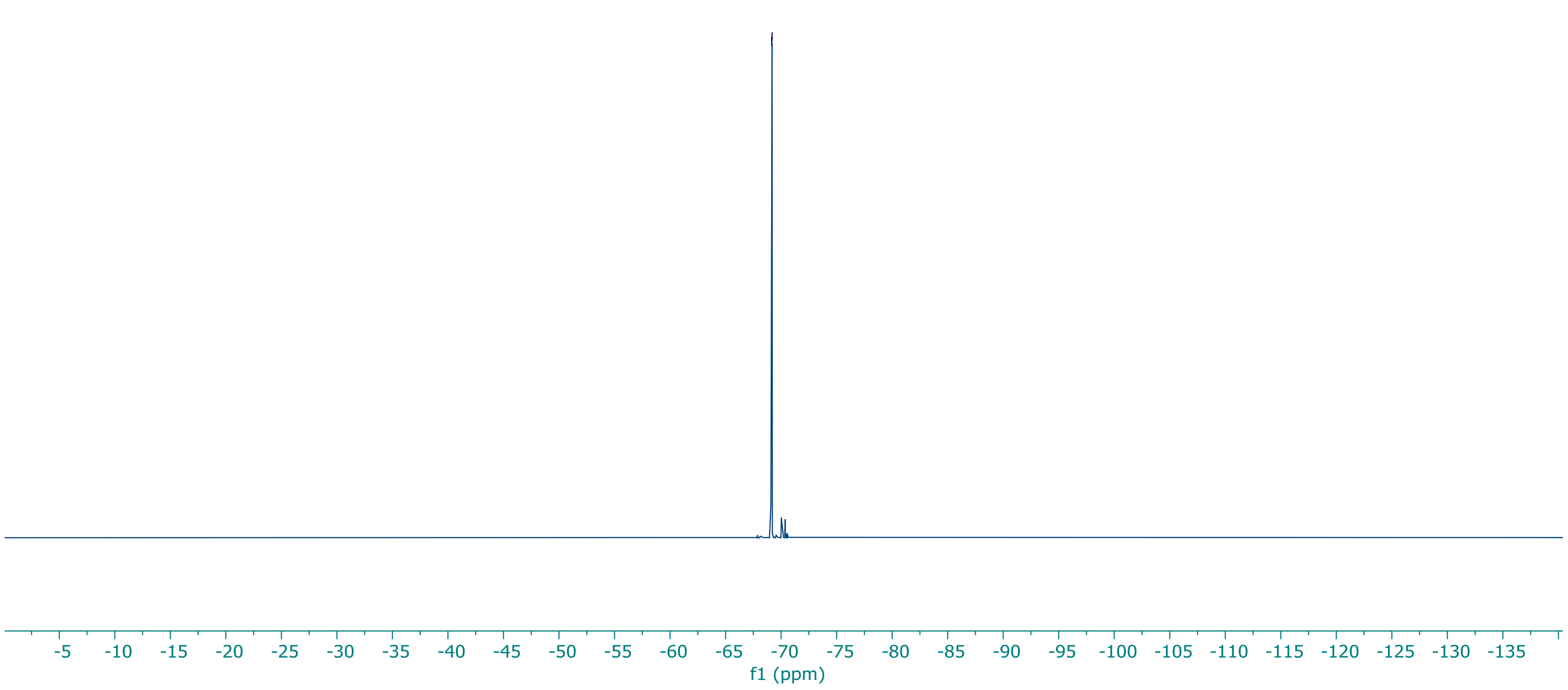

^{1}H NMR of **20**

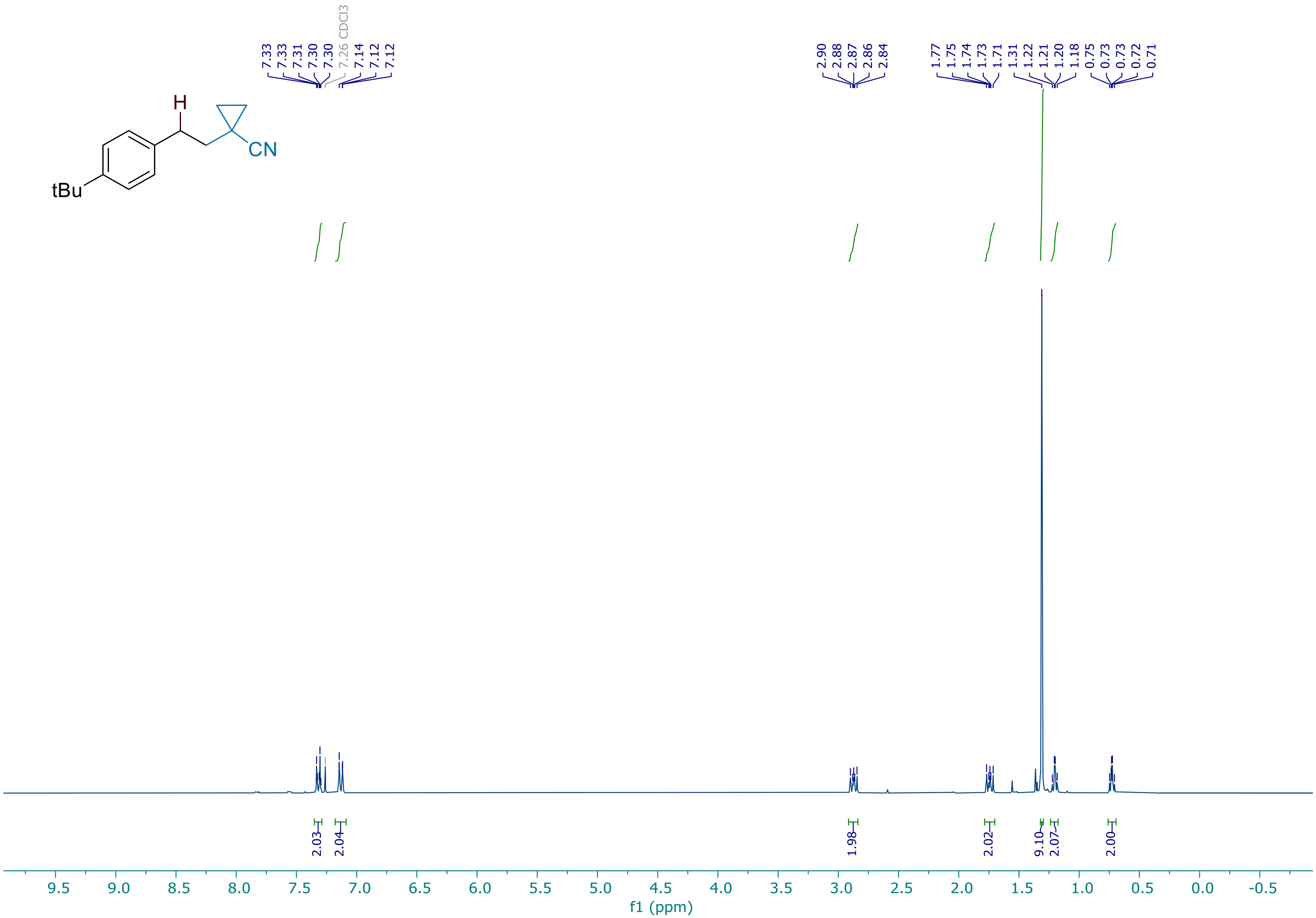

tBu
H
CN
7.33
7.33
7.31
7.30
7.30
7.26 CDCl3
7.14
7.12
7.12
2.90
2.88
2.87
2.86
2.84
1.77
1.75
1.74
1.73
1.71
1.31
1.22
1.21
1.20
1.18
0.75
0.73
0.73
0.72
0.71
2.03
2.04
1.98
2.02
9.10
2.07
2.00
9.5
9.0
8.5
8.0
7.5
7.0
6.5
6.0
5.5
5.0
4.5
4.0
3.5
3.0
2.5
2.0
1.5
1.0
0.5
0.0
-0.5
f1 (ppm)

^{13}C NMR of **20**

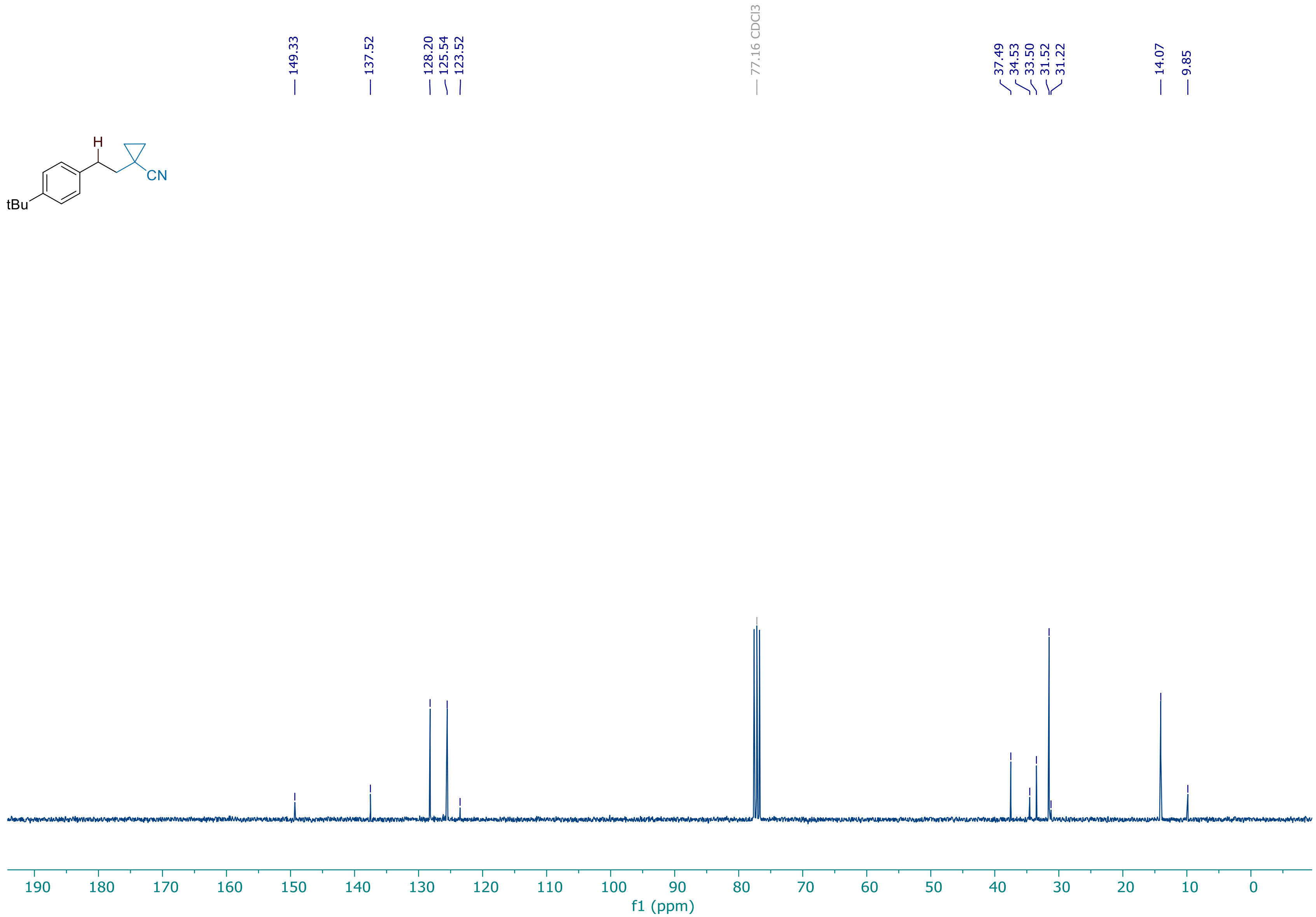

149.33
137.52
128.20
125.54
123.52
77.16 CDCl3
37.49
34.53
33.50
31.52
31.22
14.07
9.85
H
CN
tBu
190
180
170
160
150
140
130
120
110
100
90
80
70
60
50
40
30
20
10
0
f1 (ppm)

^{1}H NMR of **21**

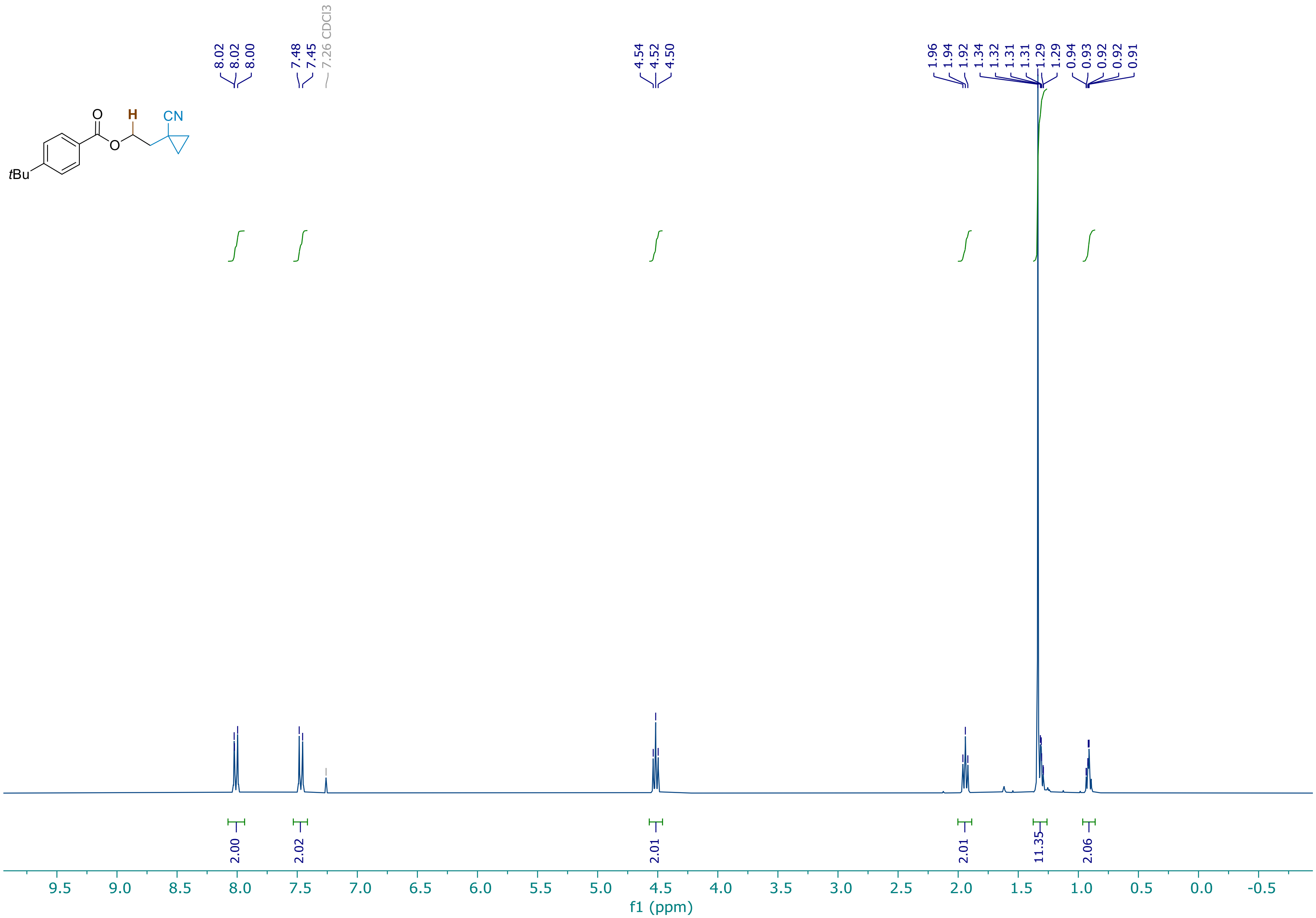
tBu
O
O
H
CN
8.02
8.02
8.00
7.48
7.45
7.26 CDCl3
4.54
4.52
4.50
1.96
1.94
1.92
1.34
1.32
1.31
1.31
1.29
1.29
0.94
0.93
0.92
0.92
0.91
2.00
2.02
2.01
2.01
11.35
2.06
9.5
9.0
8.5
8.0
7.5
7.0
6.5
6.0
5.5
5.0
4.5
4.0
3.5
3.0
2.5
2.0
1.5
1.0
0.5
0.0
-0.5
f1 (ppm)

^{13}C NMR of **21**

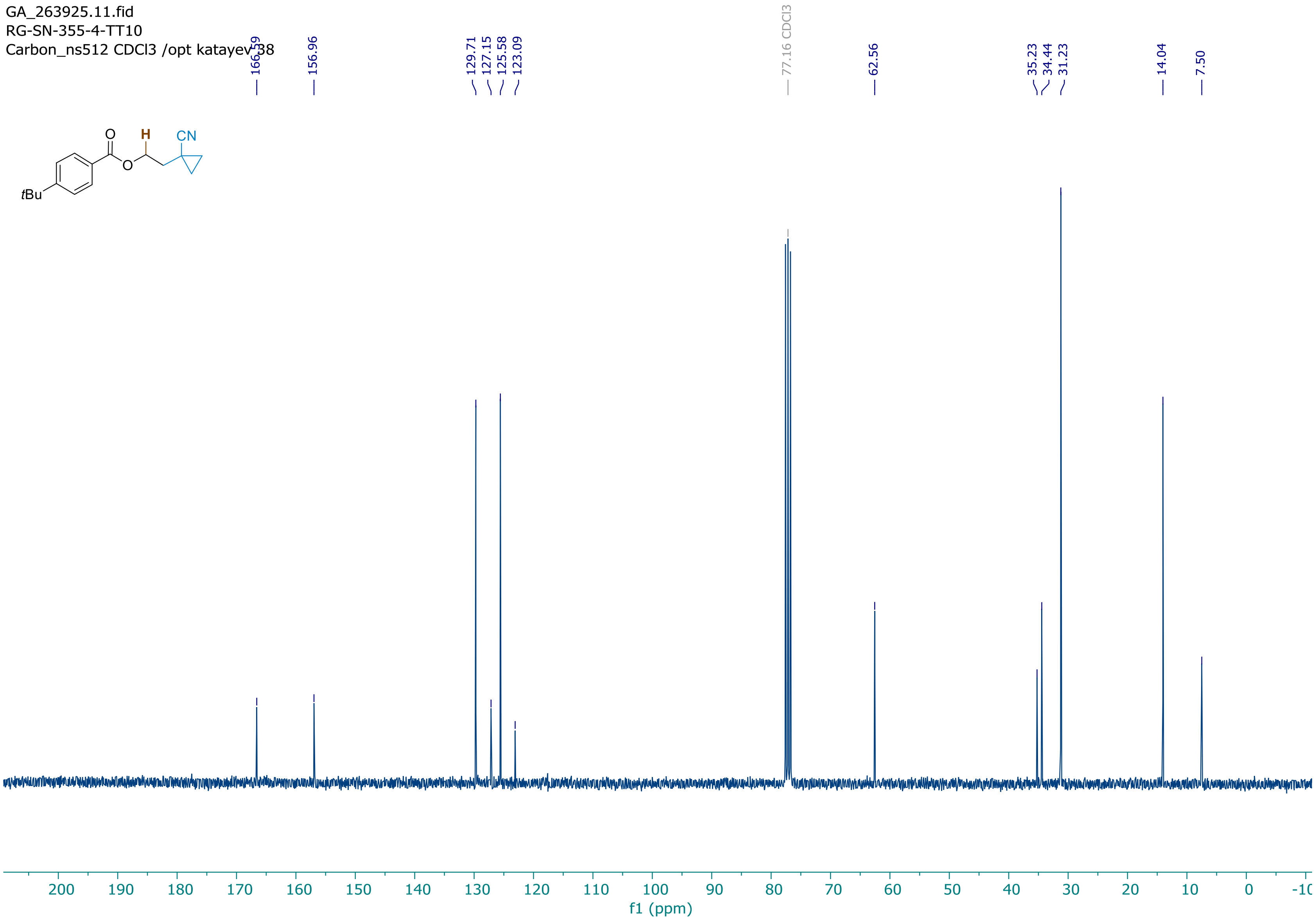

GA_263925.11.fid
RG-SN-355-4-TT10
Carbon_ns512 CDCl3 /opt katayev 38
166.59
156.96
129.71
127.15
125.58
123.09
77.16 CDCl3
62.56
35.23
34.44
31.23
14.04
7.50
O
H
CN
O
tBu
200 190 180 170 160 150 140 130 120 110 100 90 80 70 60 50 40 30 20 10 0 -10
f1 (ppm)

¹H NMR of **22**

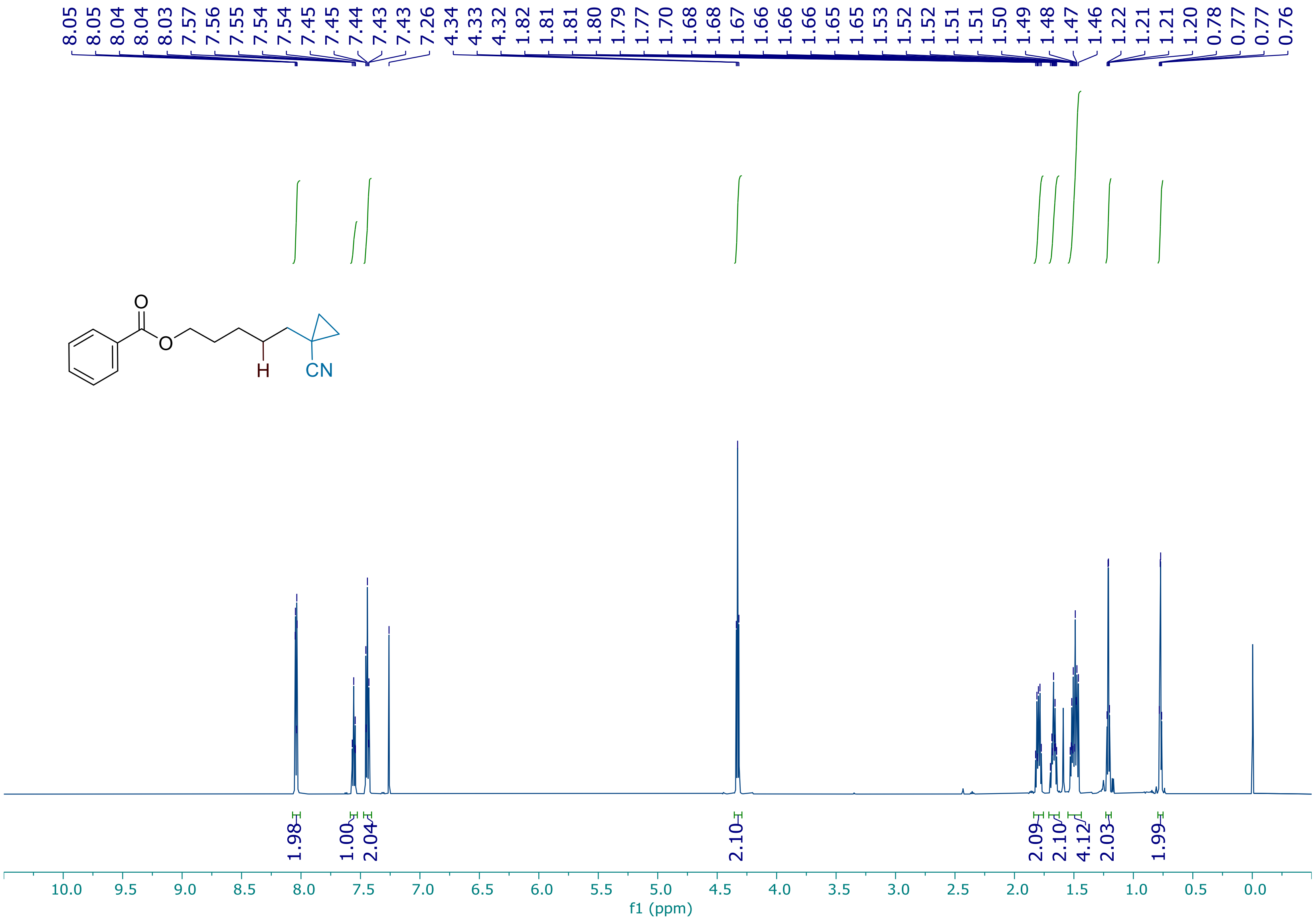

^{13}C NMR of **22**

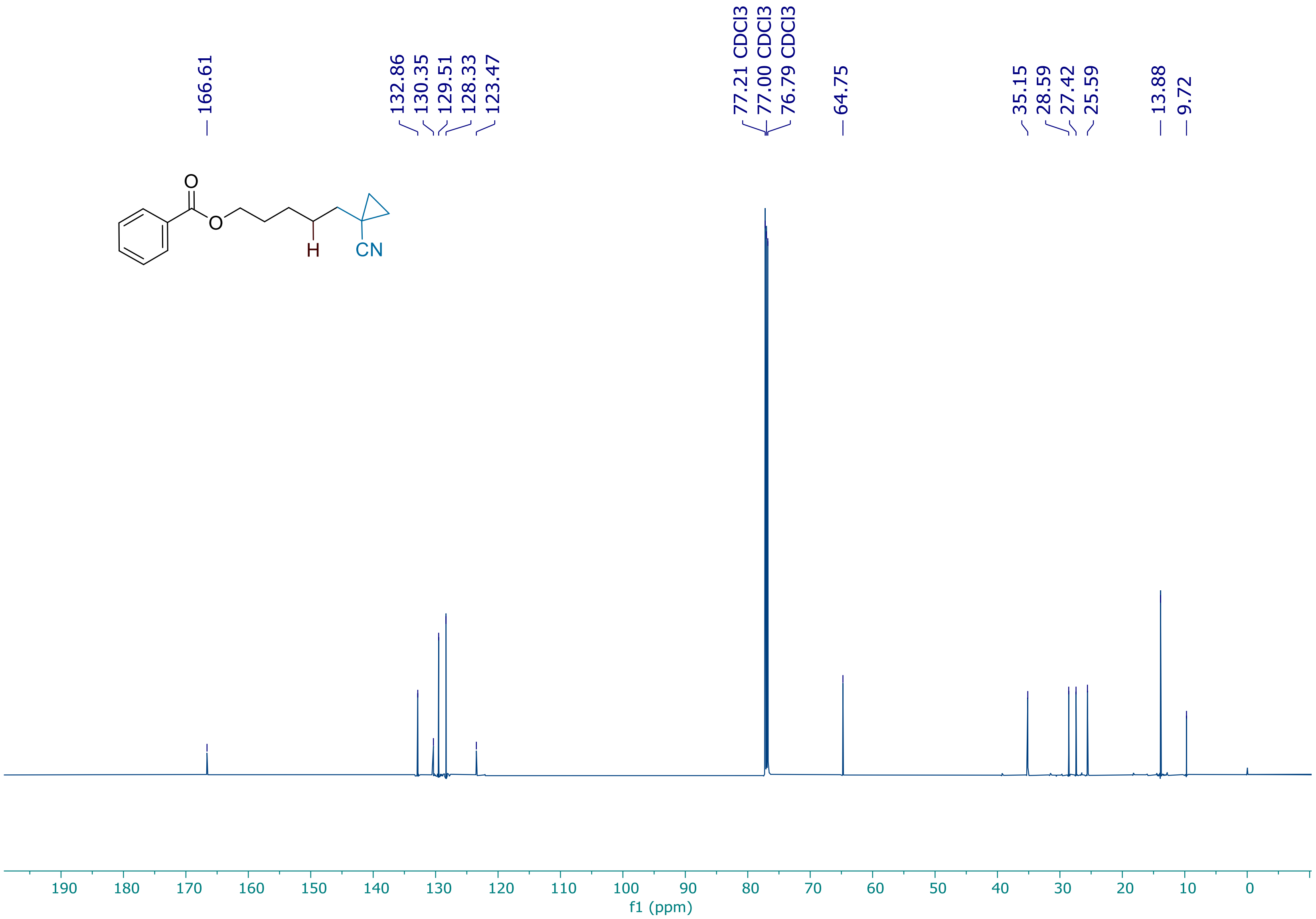

166.61
132.86
130.35
129.51
128.33
123.47
77.21 CDCl3
77.00 CDCl3
76.79 CDCl3
64.75
35.15
28.59
27.42
25.59
13.88
9.72
O
O
H
CN
190 180 170 160 150 140 130 120 110 100 90 80 70 60 50 40 30 20 10 0
f1 (ppm)

^{1}H NMR of **23**

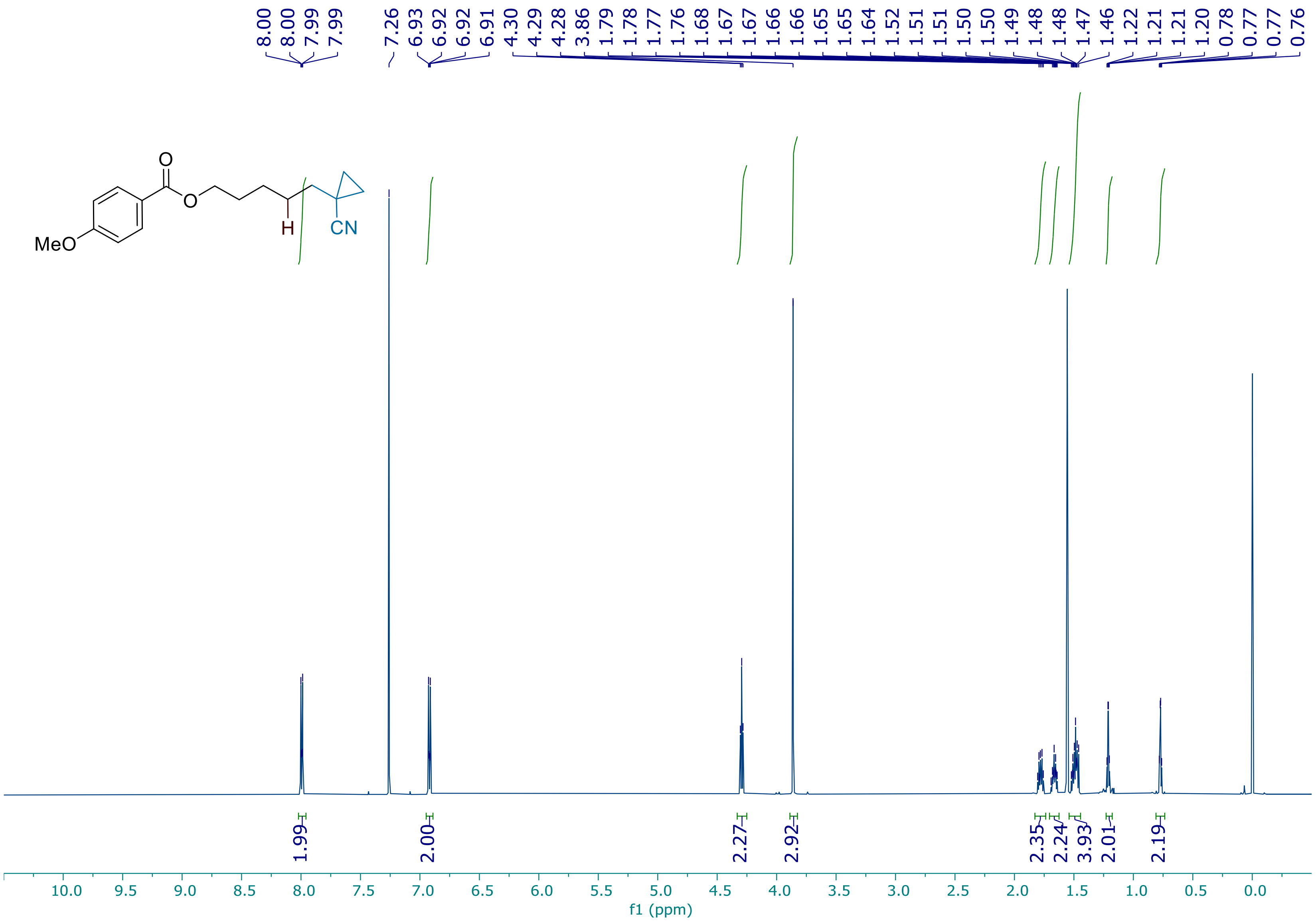

^{13}C NMR of **23**

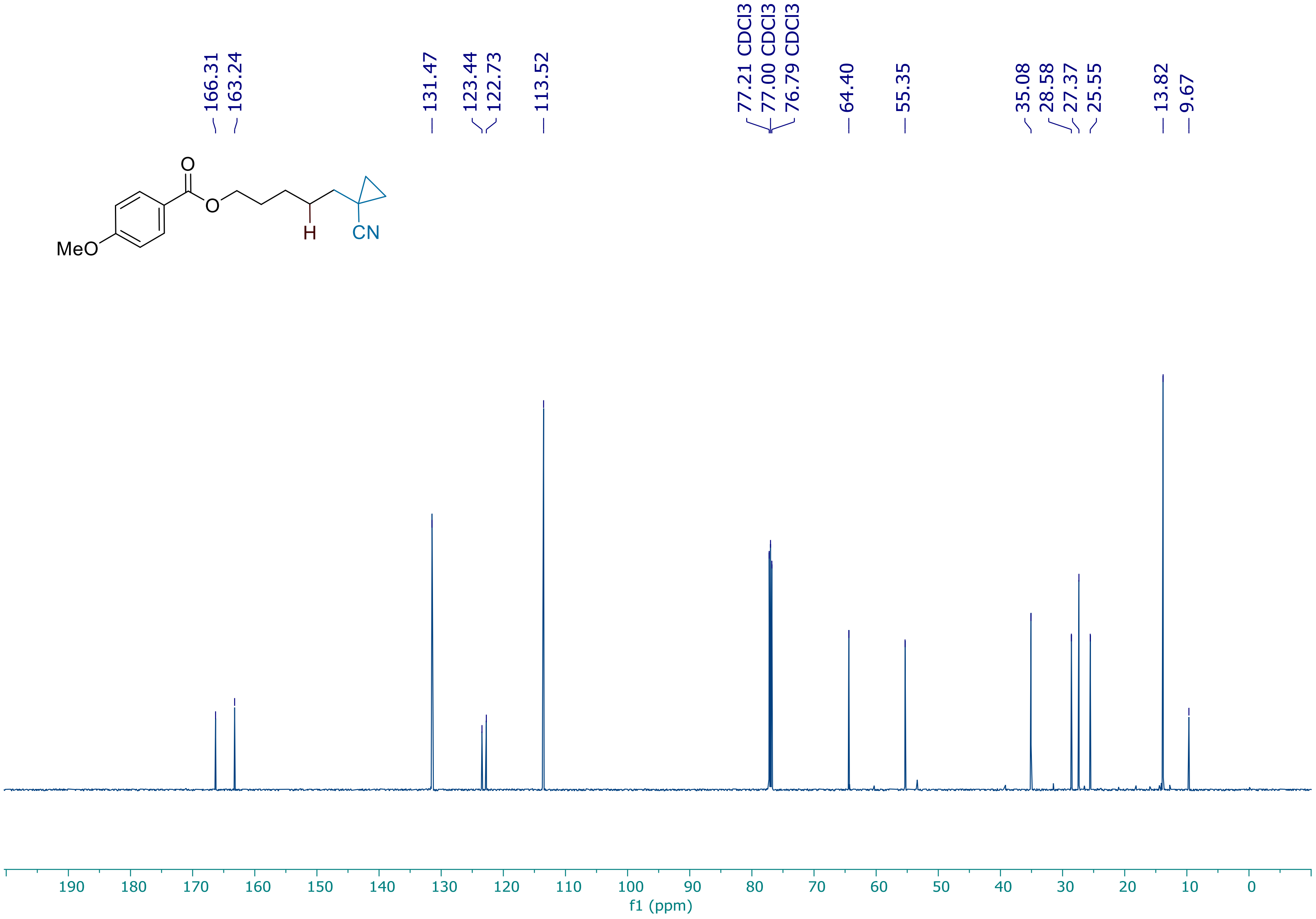

MeO
O
O
H
CN
166.31
163.24
131.47
123.44
122.73
113.52
77.21 CDCl3
77.00 CDCl3
76.79 CDCl3
64.40
55.35
35.08
28.58
27.37
25.55
13.82
9.67
190 180 170 160 150 140 130 120 110 100 90 80 70 60 50 40 30 20 10 0
f1 (ppm)

^{1}H NMR of **24**

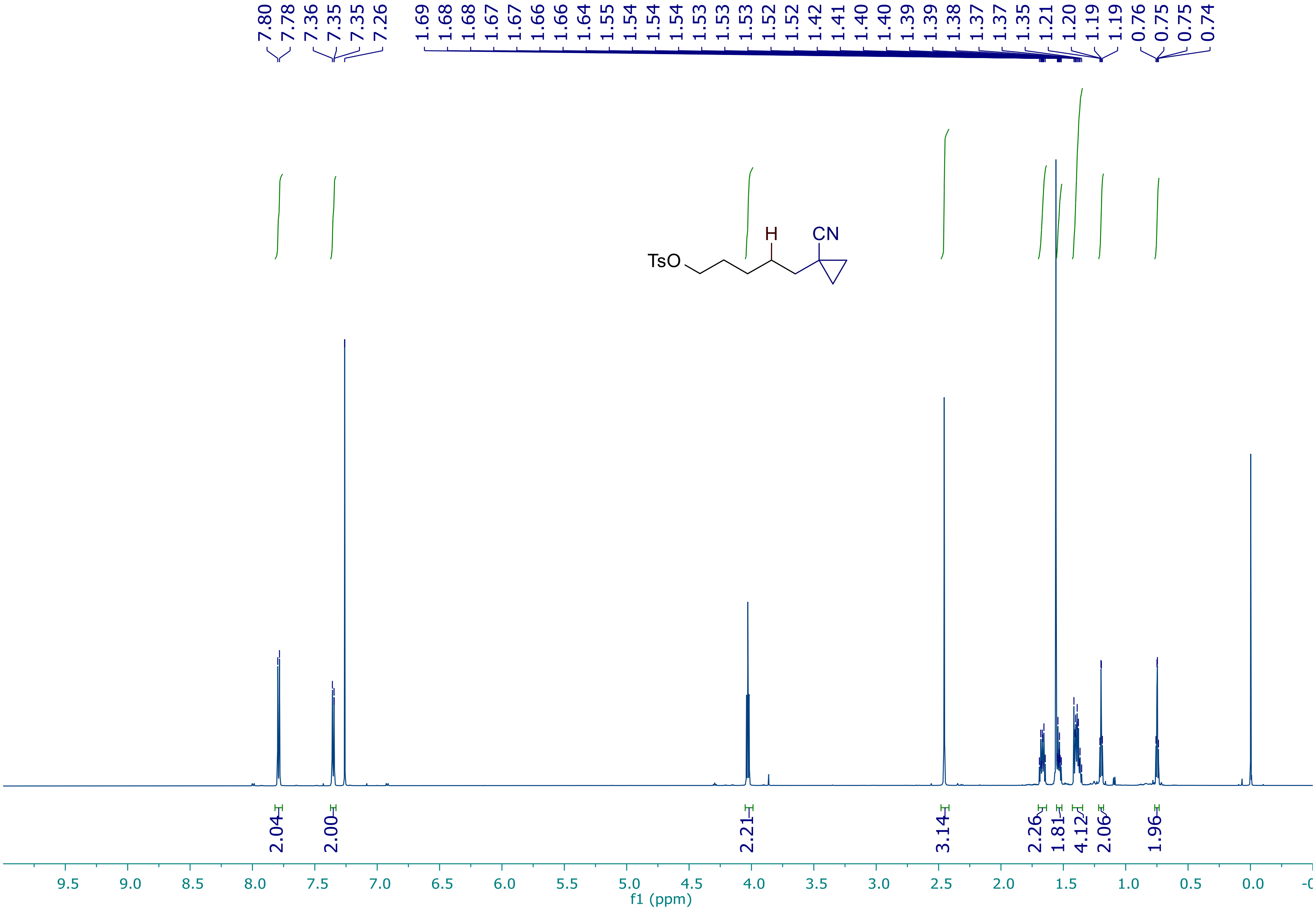

^{13}C NMR of **24**

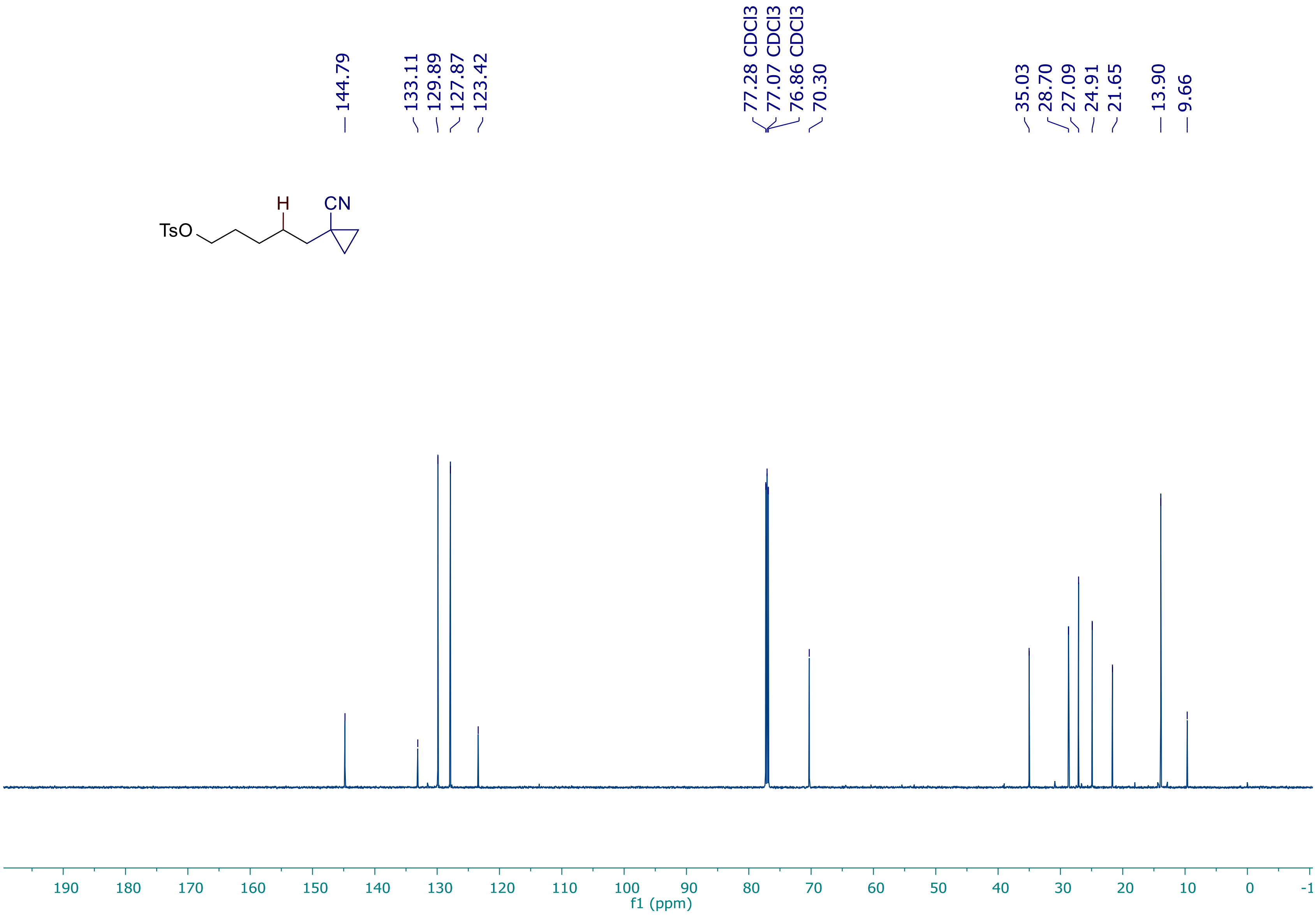

TsO
H
CN
144.79
133.11
129.89
127.87
123.42
77.28 CDCl3
77.07 CDCl3
76.86 CDCl3
70.30
35.03
28.70
27.09
24.91
21.65
13.90
9.66
190 180 170 160 150 140 130 120 110 100 90 80 70 60 50 40 30 20 10 0 -1
f1 (ppm)

^{1}H NMR of **25**

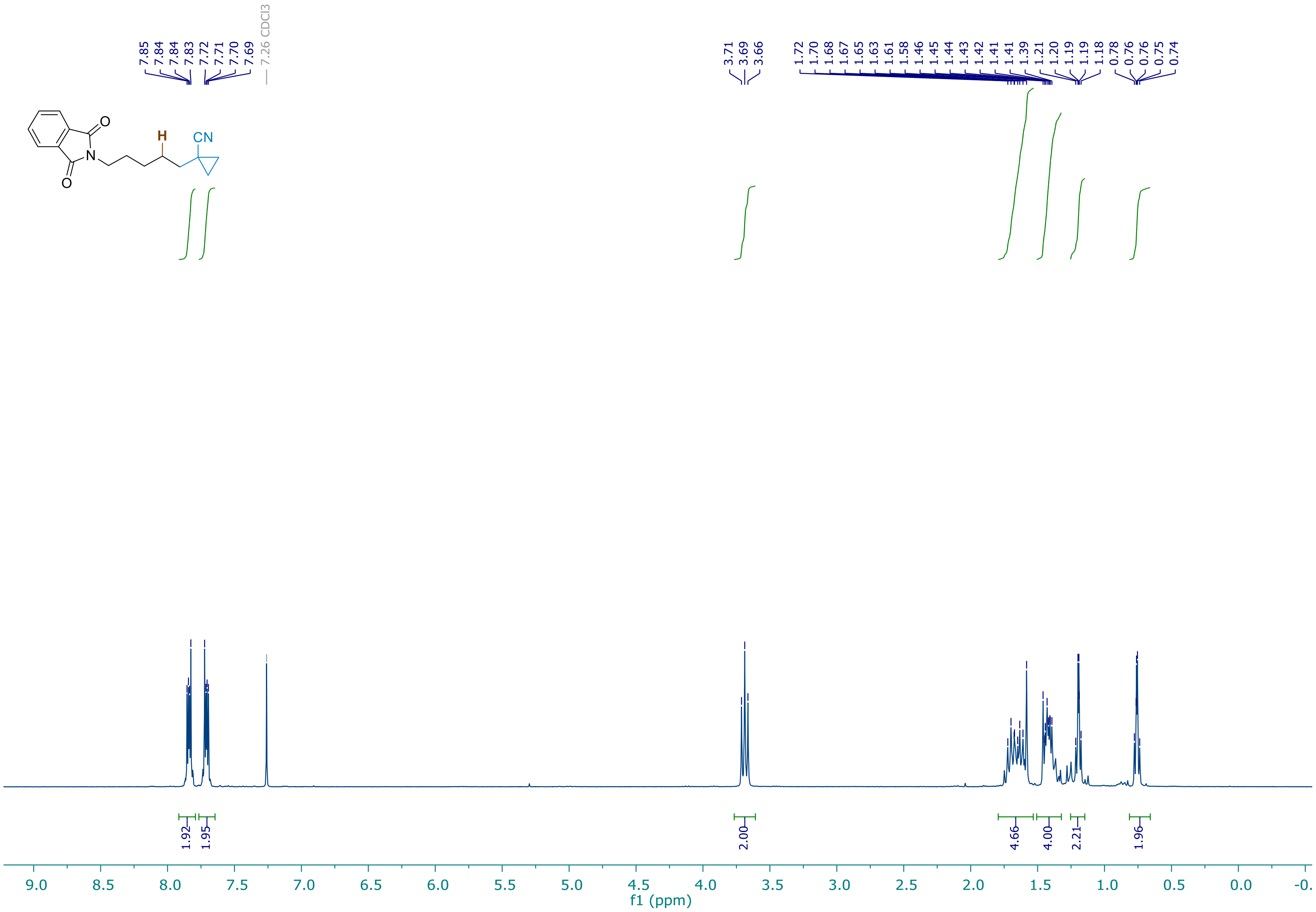

^{13}C NMR of **25**

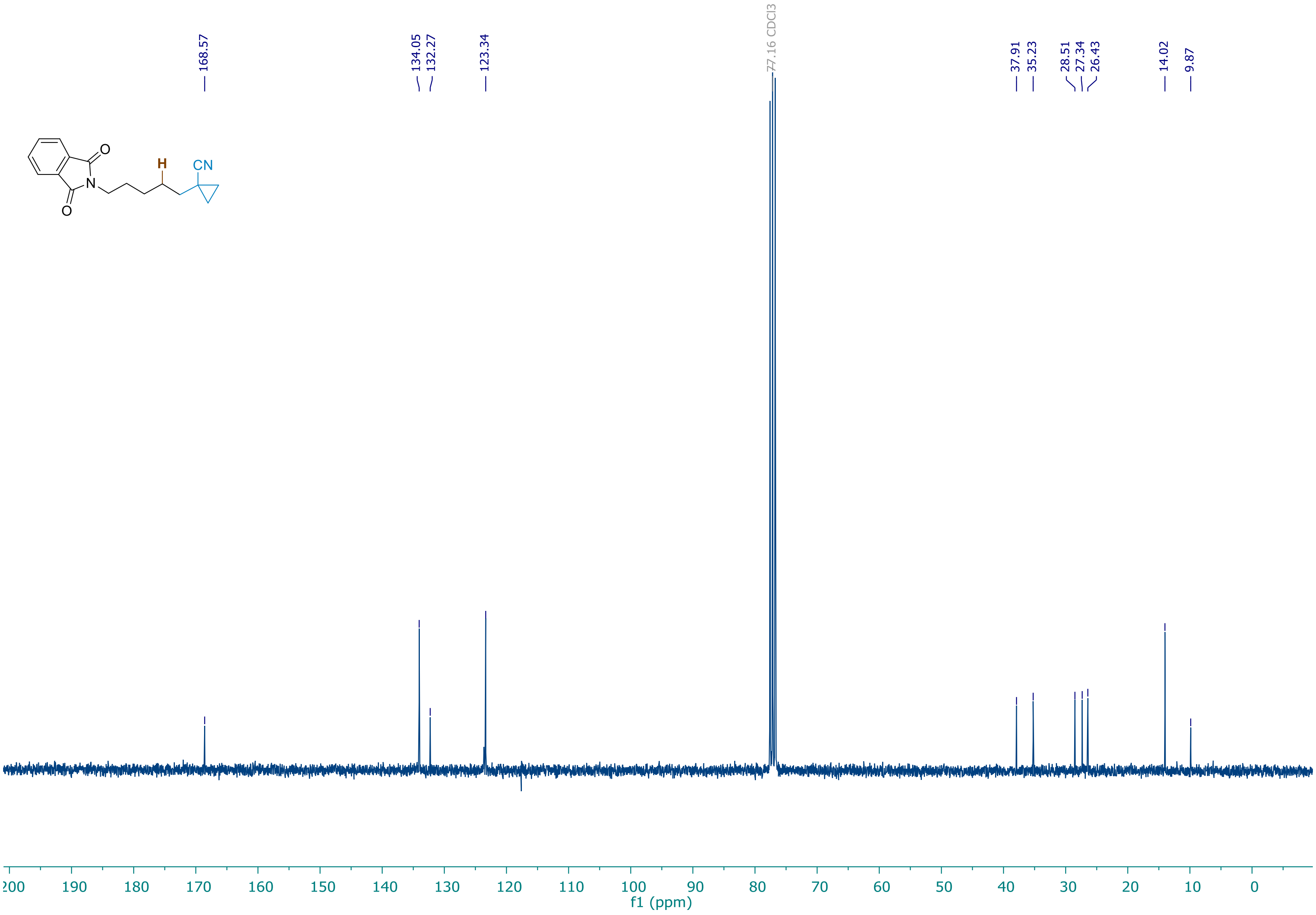

168.57
134.05
132.27
123.34
77.16 CDCl3
37.91
35.23
28.51
27.34
26.43
14.02
9.87
H
CN
O
N
O
200 190 180 170 160 150 140 130 120 110 100 90 80 70 60 50 40 30 20 10 0
f1 (ppm)

^{1}H NMR of **26**

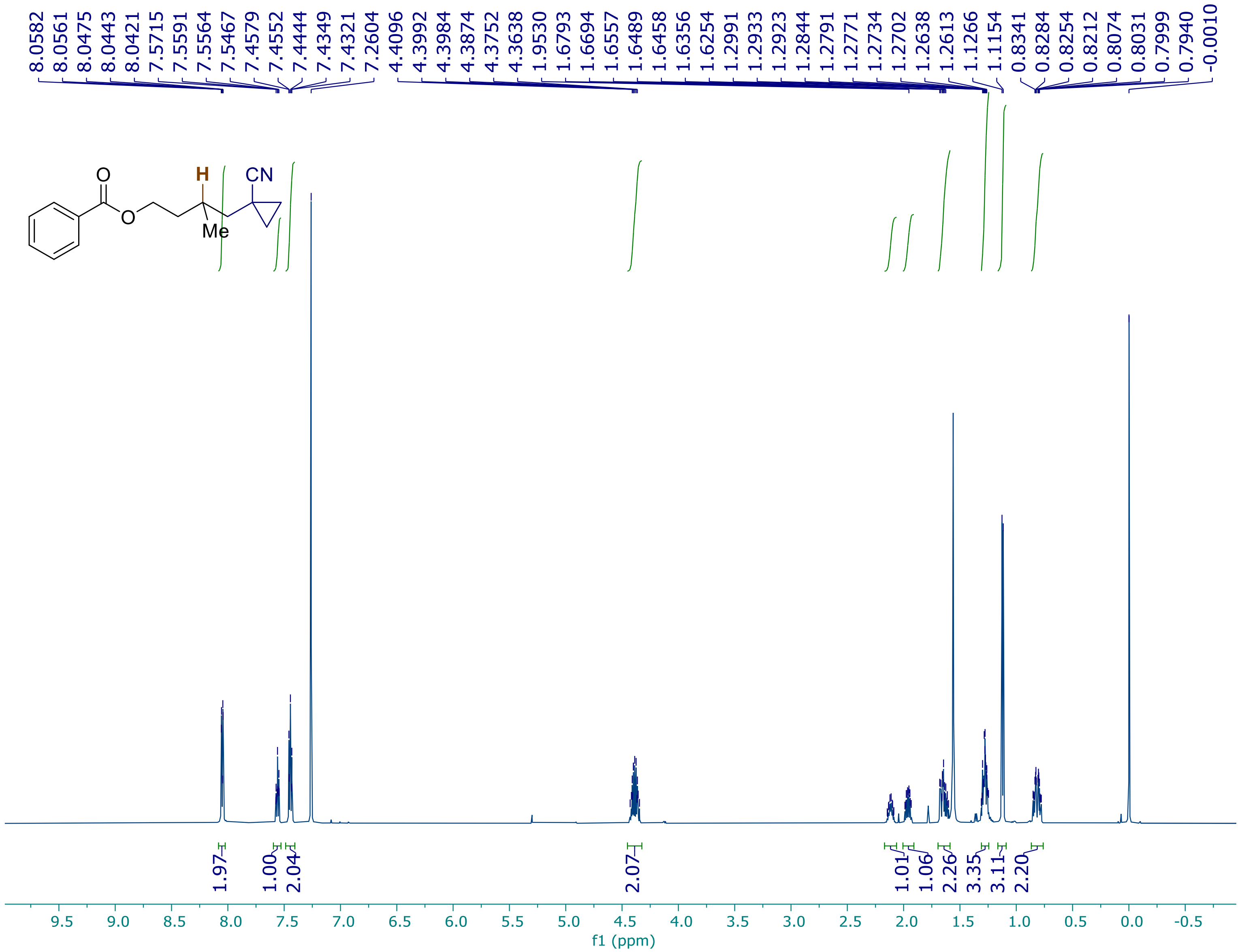

8.0582
8.0561
8.0475
8.0443
8.0421
7.5715
7.5591
7.5564
7.5467
7.4579
7.4552
7.4444
7.4349
7.4321
7.2604
4.4096
4.3992
4.3984
4.3874
4.3752
4.3638
1.9530
1.6793
1.6694
1.6557
1.6489
1.6458
1.6356
1.6254
1.2991
1.2933
1.2923
1.2844
1.2791
1.2771
1.2734
1.2702
1.2638
1.2613
1.1266
1.1154
0.8341
0.8284
0.8254
0.8212
0.8074
0.8031
0.7999
0.7940
-0.0010
O
O
H
CN
Me
1.97
1.00
2.04
2.07
1.01
1.06
2.26
3.35
3.11
2.20
9.5 9.0 8.5 8.0 7.5 7.0 6.5 6.0 5.5 5.0 4.5 4.0 3.5 3.0 2.5 2.0 1.5 1.0 0.5 0.0 -0.5
f1 (ppm)

^{13}C NMR of **26**

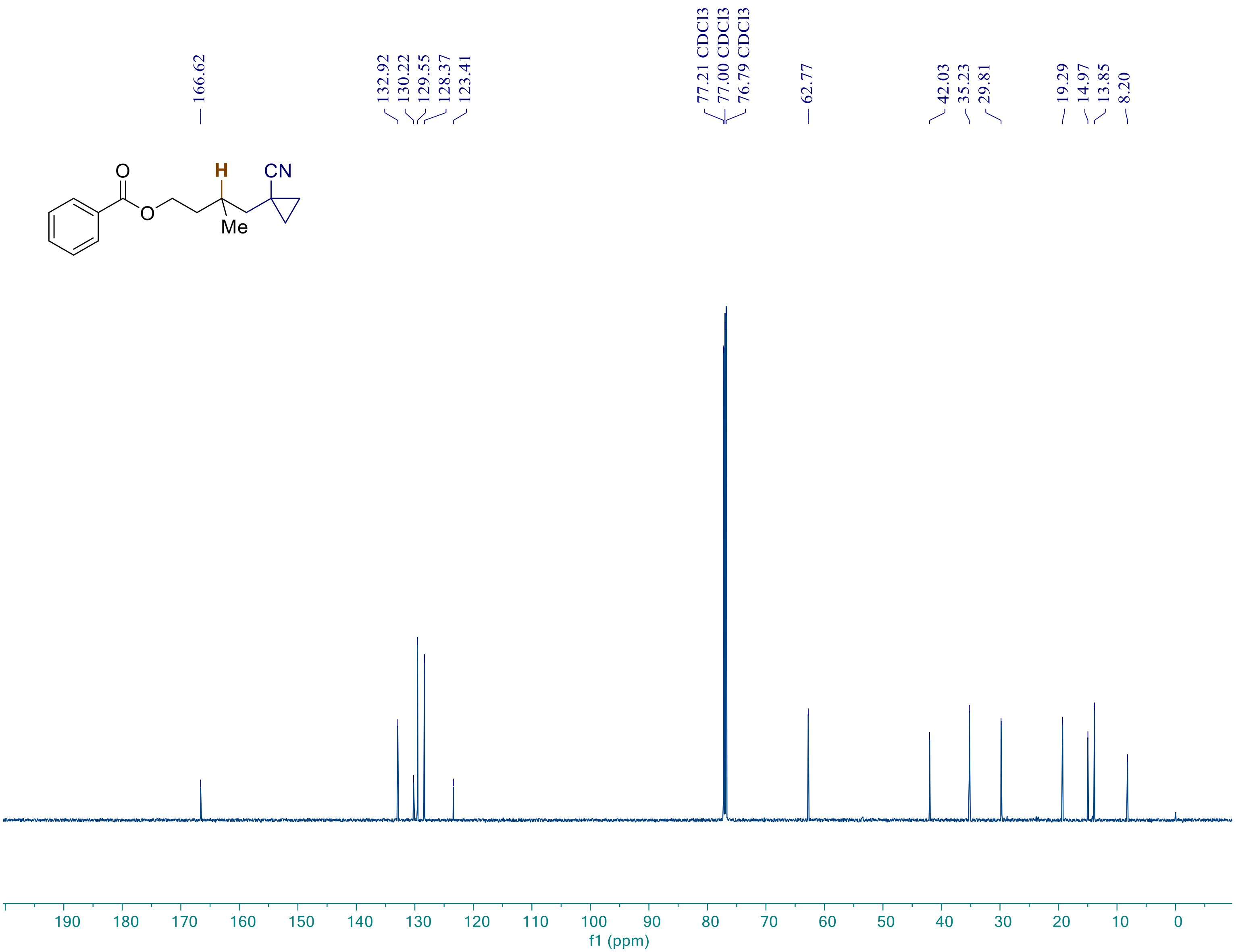
166.62
132.92
130.22
129.55
128.37
123.41
77.21 CDCl3
77.00 CDCl3
76.79 CDCl3
62.77
42.03
35.23
29.81
19.29
14.97
13.85
8.20
O
O
H
CN
Me
190
180
170
160
150
140
130
120
110
100
90
80
70
60
50
40
30
20
10
0
f1 (ppm)

^{1}H NMR of **27**

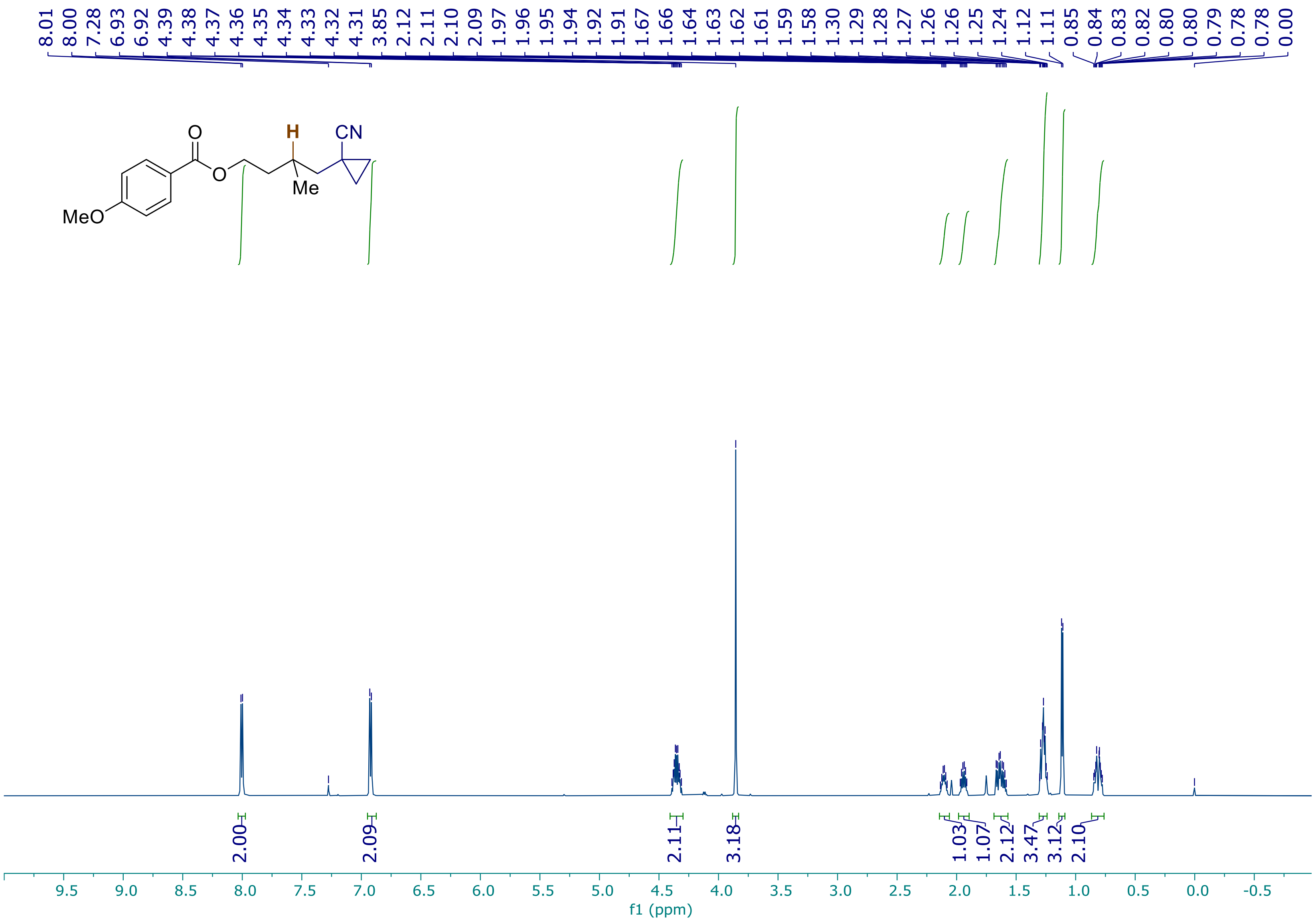

8.01 8.00 7.28 6.93 6.92 4.39 4.38 4.37 4.36 4.35 4.34 4.33 4.32 4.31 3.85 2.12 2.11 2.10 2.09 1.97 1.96 1.95 1.94 1.92 1.91 1.67 1.66 1.64 1.63 1.62 1.61 1.59 1.58 1.30 1.29 1.28 1.27 1.26 1.26 1.25 1.24 1.12 1.11 0.85 0.84 0.83 0.82 0.80 0.80 0.79 0.78 0.78 0.00
O
H
CN
O
Me
MeO
2.00
2.09
2.11
3.18
1.03
1.07
2.12
3.47
3.12
2.10
9.5 9.0 8.5 8.0 7.5 7.0 6.5 6.0 5.5 5.0 4.5 4.0 3.5 3.0 2.5 2.0 1.5 1.0 0.5 0.0 -0.5
f1 (ppm)

^{13}C NMR of **27**

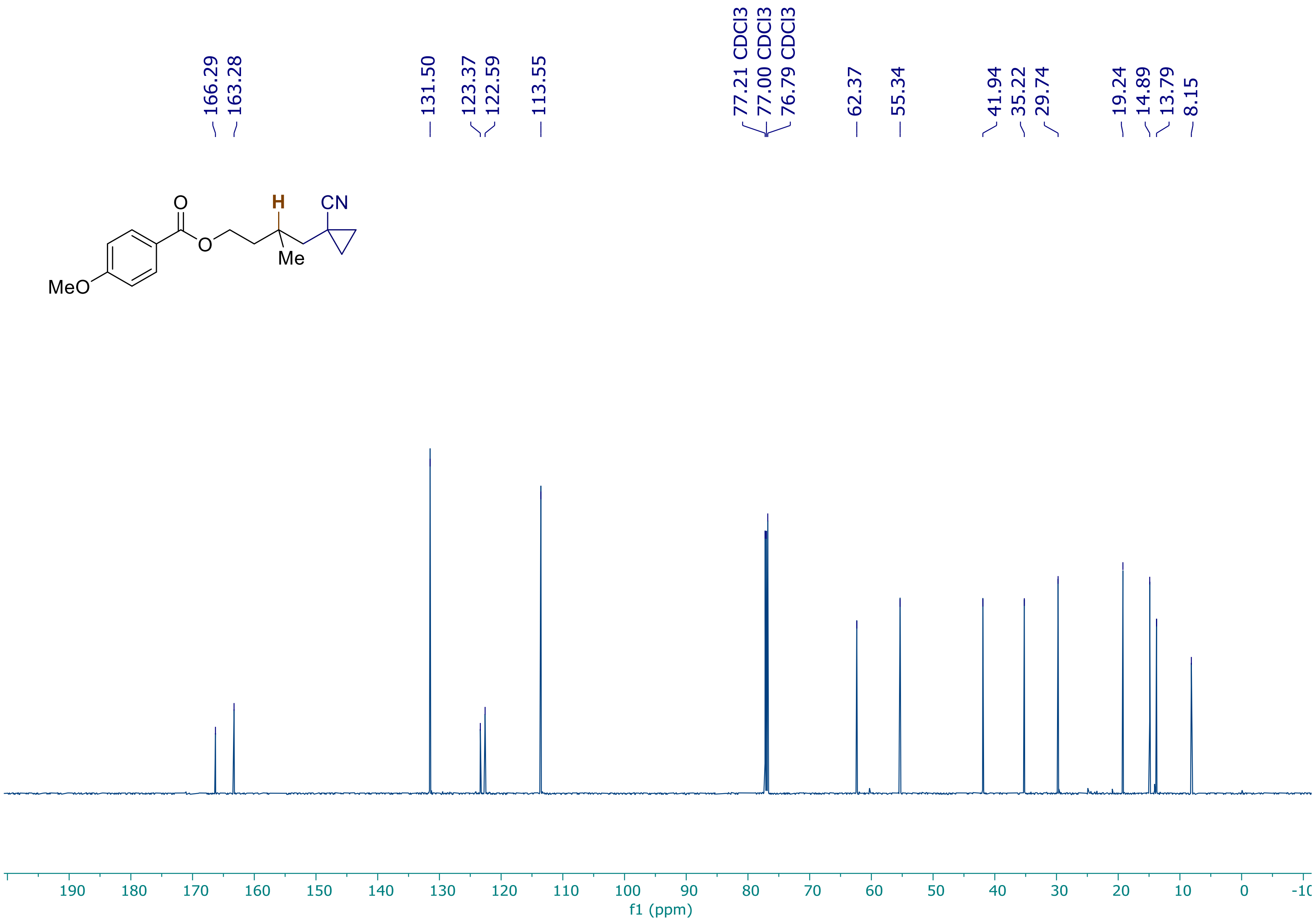

166.29
163.28
131.50
123.37
122.59
113.55
77.21 CDCl3
77.00 CDCl3
76.79 CDCl3
62.37
55.34
41.94
35.22
29.74
19.24
14.89
13.79
8.15
O
H
CN
O
Me
MeO
190
180
170
160
150
140
130
120
110
100
90
80
70
60
50
40
30
20
10
0
f1 (ppm)

^{1}H NMR of **28**

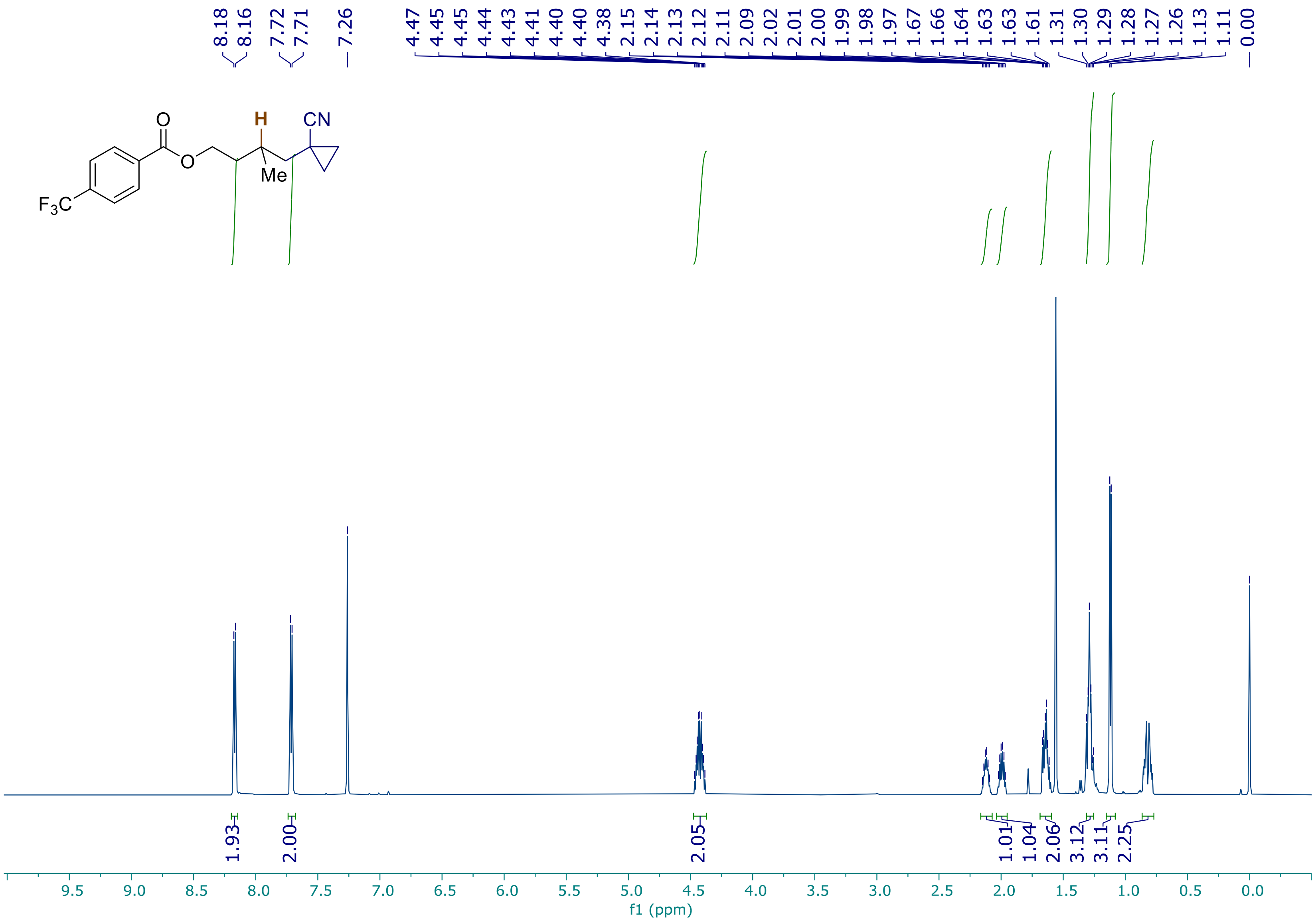

^{13}C NMR of **28**

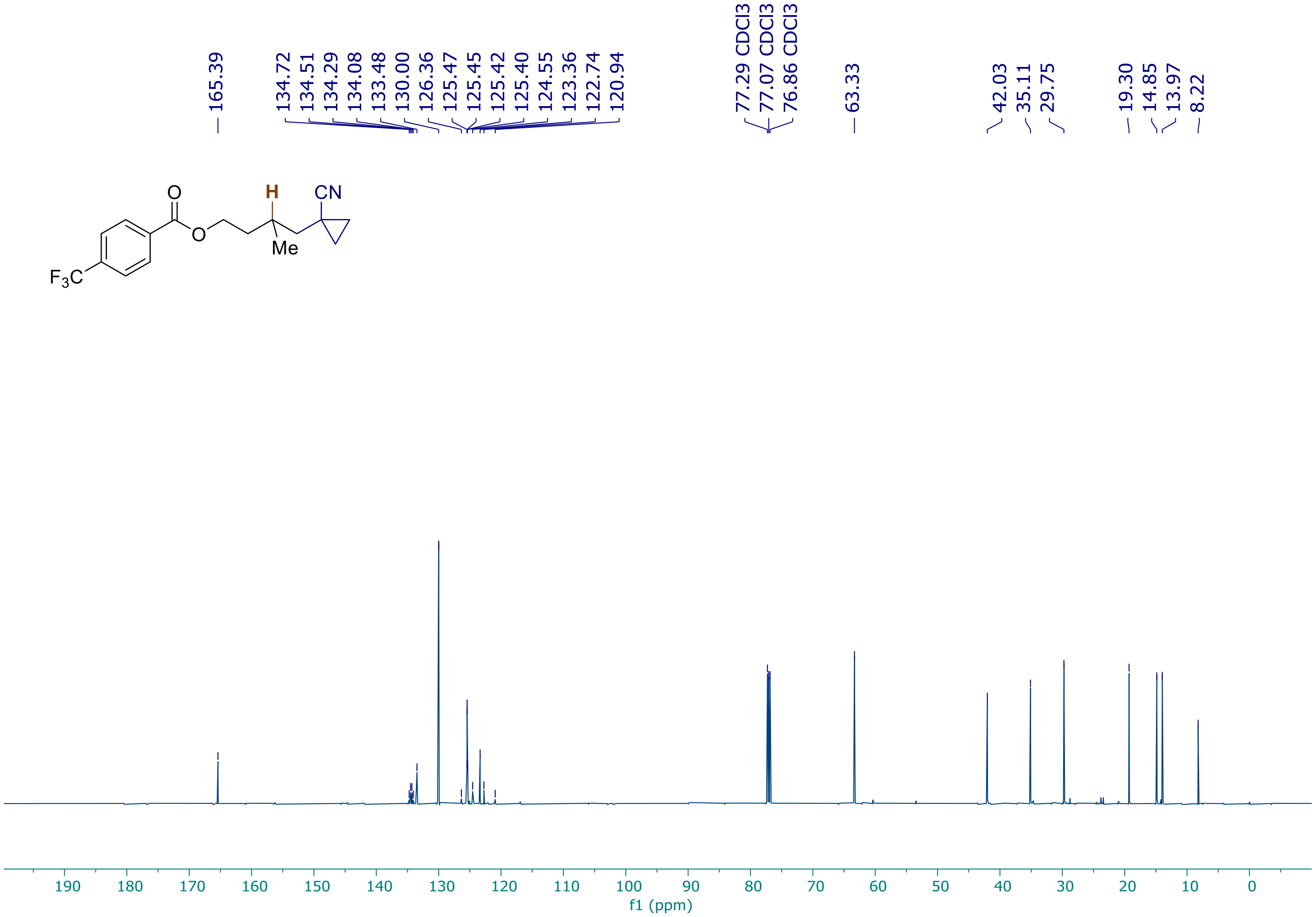
165.39
134.72
134.51
134.29
134.08
133.48
130.00
126.36
125.47
125.45
125.42
125.40
124.55
123.36
122.74
120.94
77.29 CDCl3
77.07 CDCl3
76.86 CDCl3
63.33
42.03
35.11
29.75
19.30
14.85
13.97
8.22
190
180
170
160
150
140
130
120
110
100
90
80
70
60
50
40
30
20
10
0
f1 (ppm)

^{19}F NMR of **28**

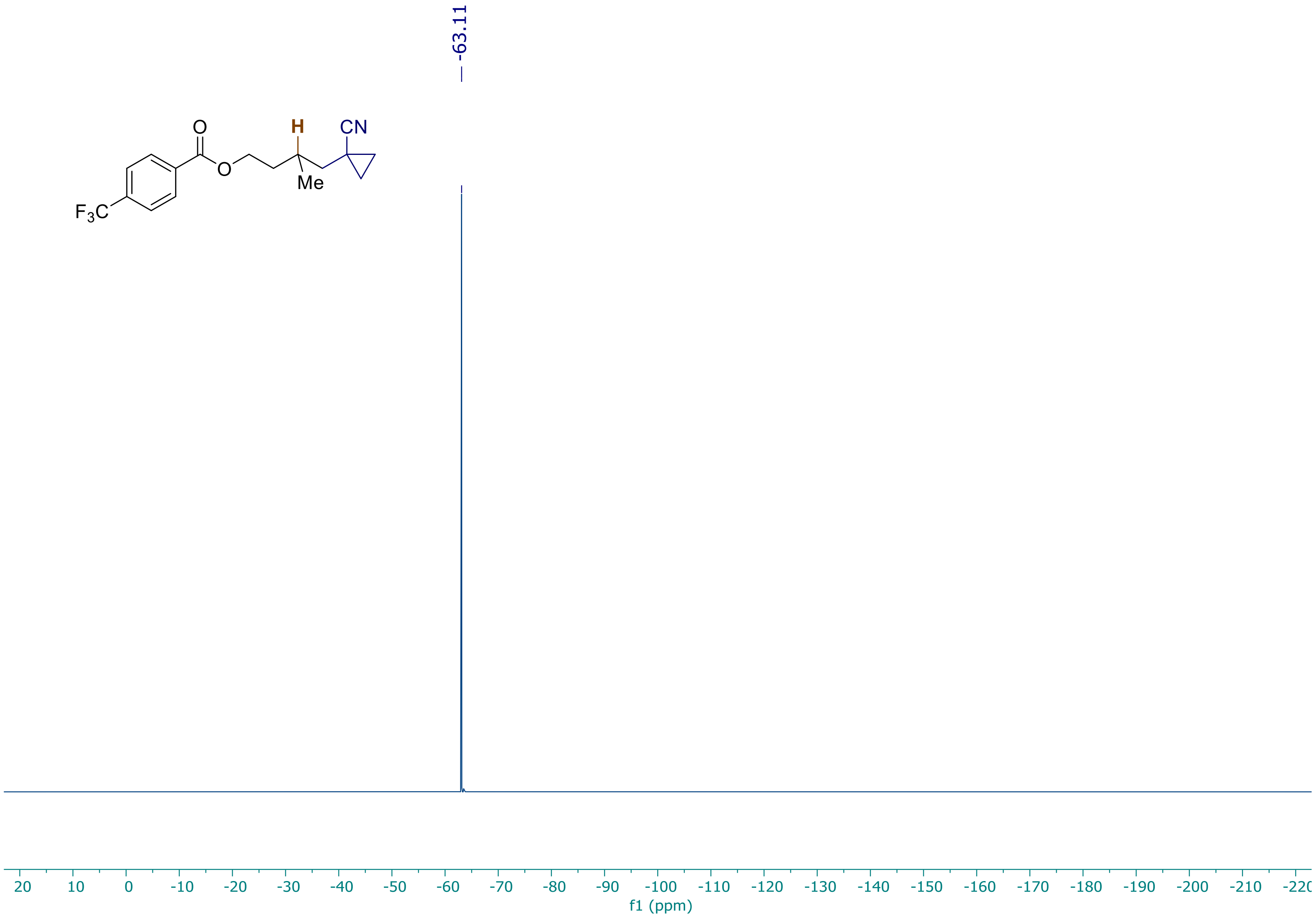

-63.11
O
H
CN
O
Me
F3C
20
10
0
-10
-20
-30
-40
-50
-60
-70
-80
-90
-100
-110
-120
-130
-140
-150
-160
-170
-180
-190
-200
-210
-220
f1 (ppm)

^{1}H NMR of **29**

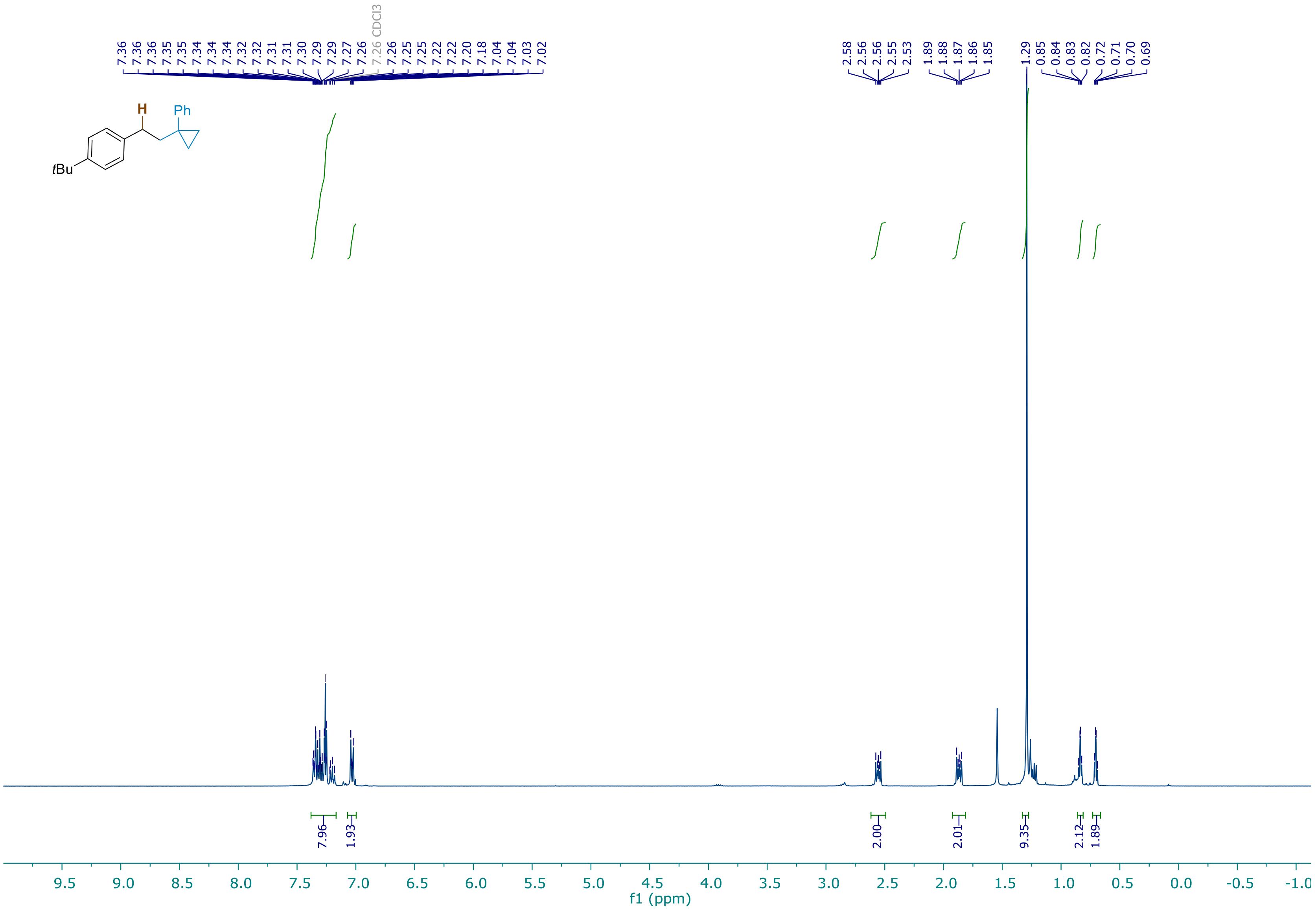

tBu
H
Ph
7.36
7.36
7.36
7.35
7.35
7.34
7.34
7.34
7.32
7.32
7.31
7.31
7.30
7.29
7.29
7.27
7.26
7.26 CDCl3
7.26
7.25
7.25
7.22
7.22
7.20
7.18
7.04
7.04
7.03
7.02
2.58
2.56
2.56
2.55
2.53
1.89
1.88
1.87
1.86
1.85
1.29
0.85
0.84
0.83
0.82
0.72
0.71
0.70
0.69
7.96
1.93
2.00
2.01
9.35
2.12
1.89
f1 (ppm)

^{13}C NMR of **29**

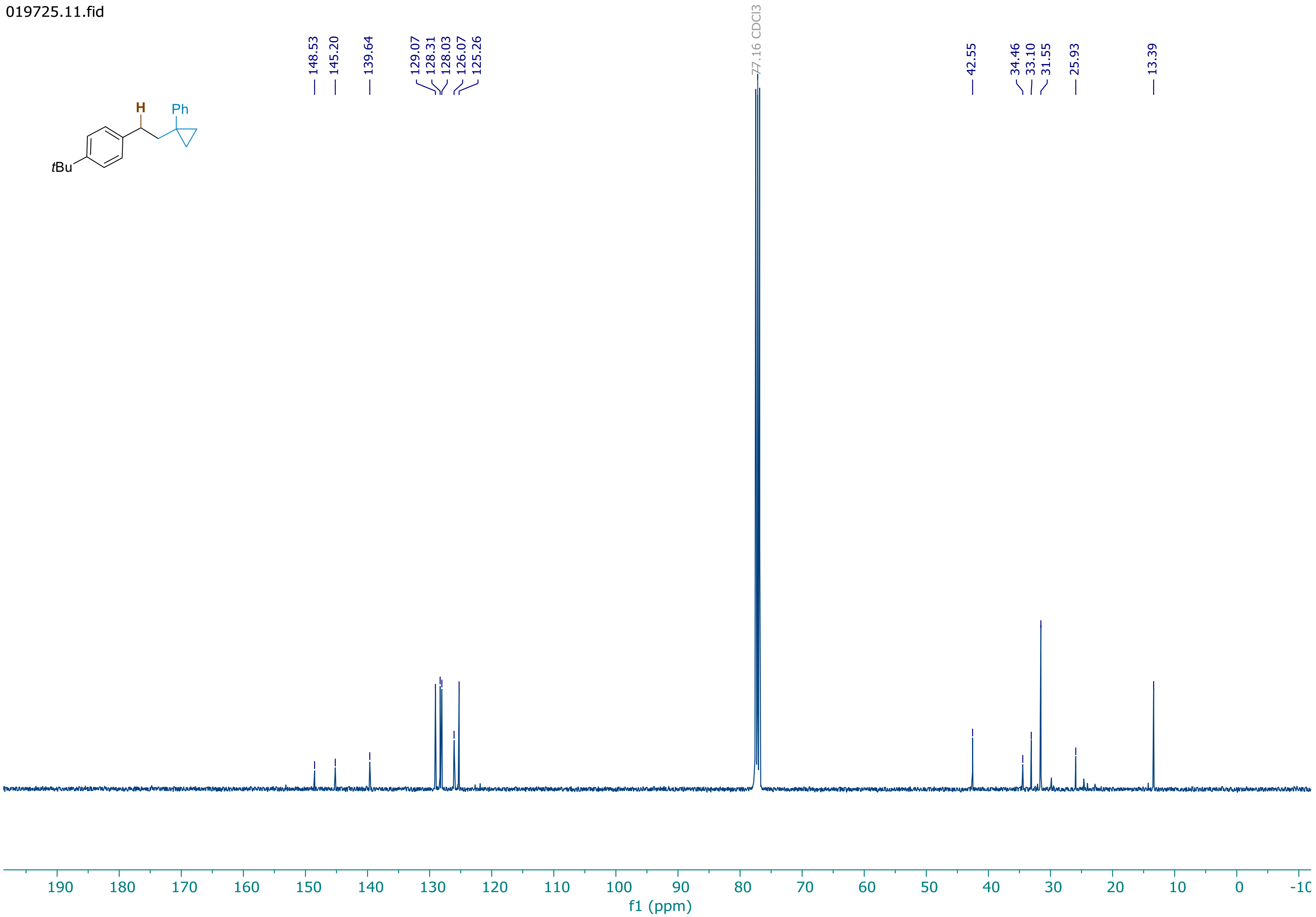

019725.11.fid
H
Ph
tBu
148.53
145.20
139.64
129.07
128.31
128.03
126.07
125.26
77.16 CDCl3
42.55
34.46
33.10
31.55
25.93
13.39
190 180 170 160 150 140 130 120 110 100 90 80 70 60 50 40 30 20 10 0 -10
f1 (ppm)

^{1}H NMR of **30**

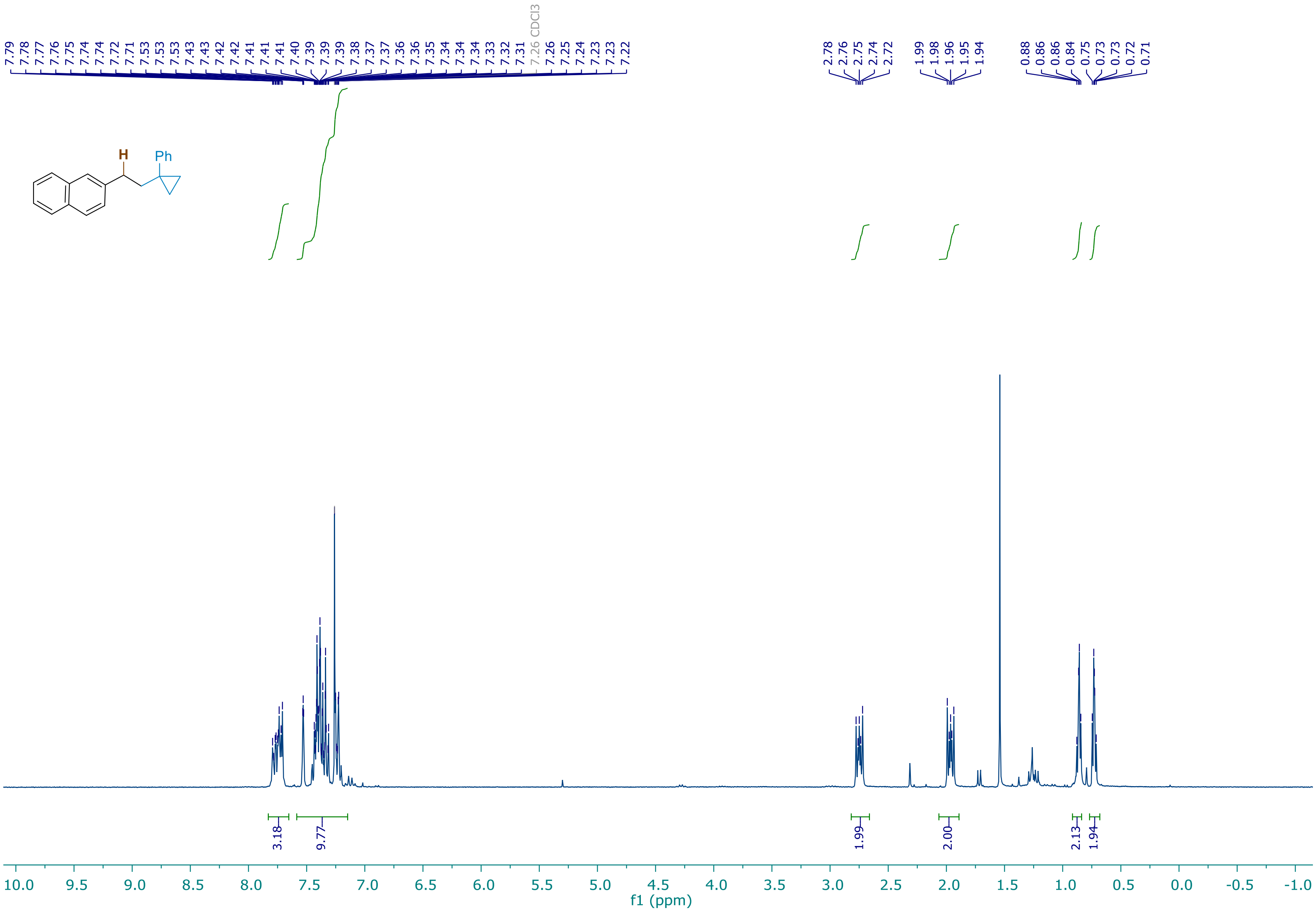
7.79 7.78 7.77 7.76 7.75 7.74 7.74 7.72 7.71 7.53 7.53 7.53 7.43 7.43 7.42 7.42 7.41 7.41 7.41 7.40 7.39 7.39 7.39 7.38 7.37 7.37 7.36 7.35 7.34 7.34 7.33 7.32 7.31 7.26 CDCl3 7.26 7.25 7.24 7.23 7.23 7.22
2.78 2.76 2.75 2.74 2.72
1.99 1.98 1.96 1.95 1.94
0.88 0.86 0.86 0.84 0.75 0.73 0.73 0.72 0.71
H
Ph
3.18
9.77
1.99
2.00
2.13
1.94
10.0 9.5 9.0 8.5 8.0 7.5 7.0 6.5 6.0 5.5 5.0 4.5 4.0 3.5 3.0 2.5 2.0 1.5 1.0 0.5 0.0 -0.5 -1.0
f1 (ppm)

^{13}C NMR of **30**

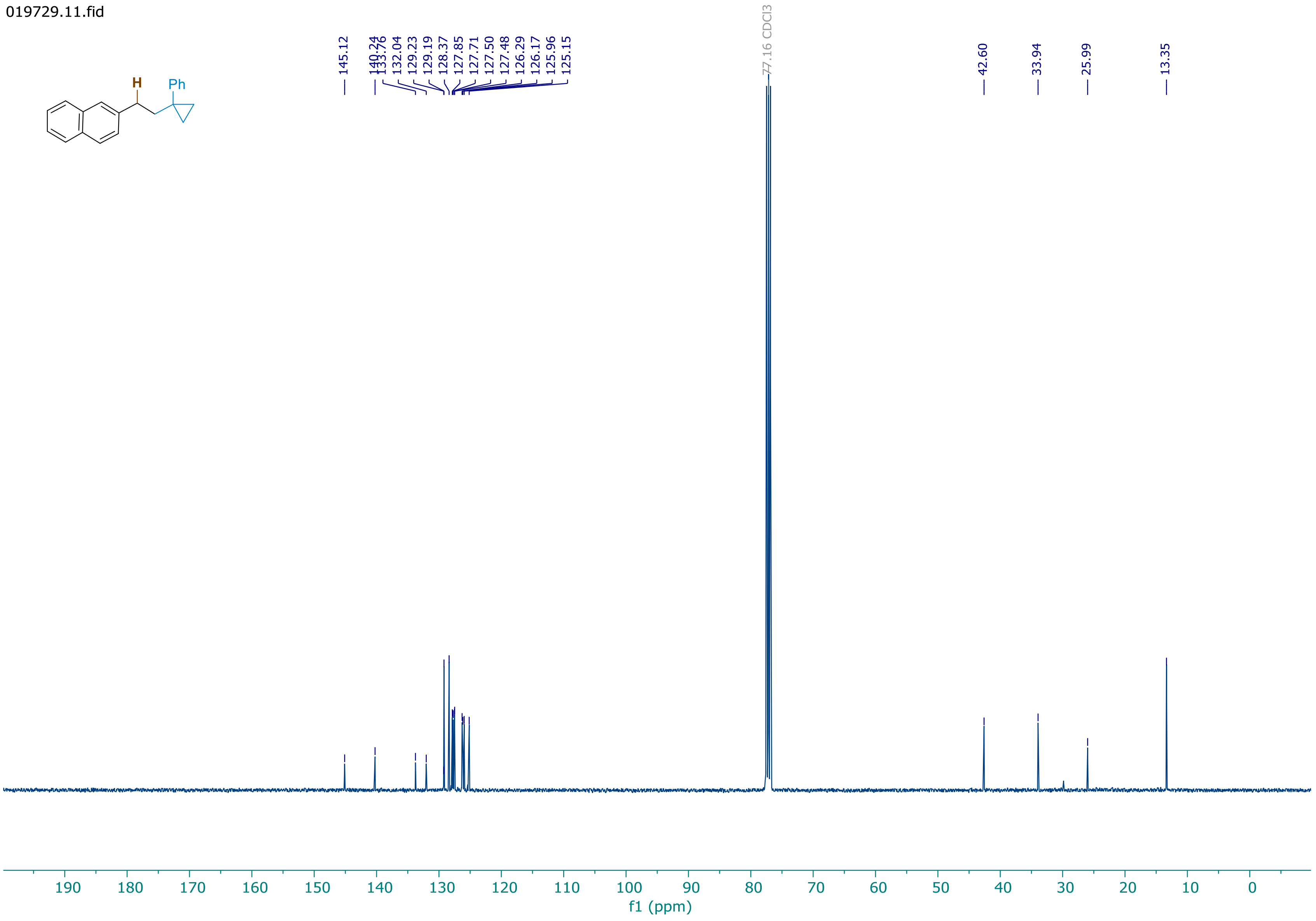
019729.11.fid
H
Ph
145.12
140.24
133.76
132.04
129.23
129.19
128.37
127.85
127.71
127.50
127.48
126.29
126.17
125.96
125.15
77.16 CDCl3
42.60
33.94
25.99
13.35
190
180
170
160
150
140
130
120
110
100
90
80
70
60
50
40
30
20
10
0
f1 (ppm)

^{1}H NMR of **31**

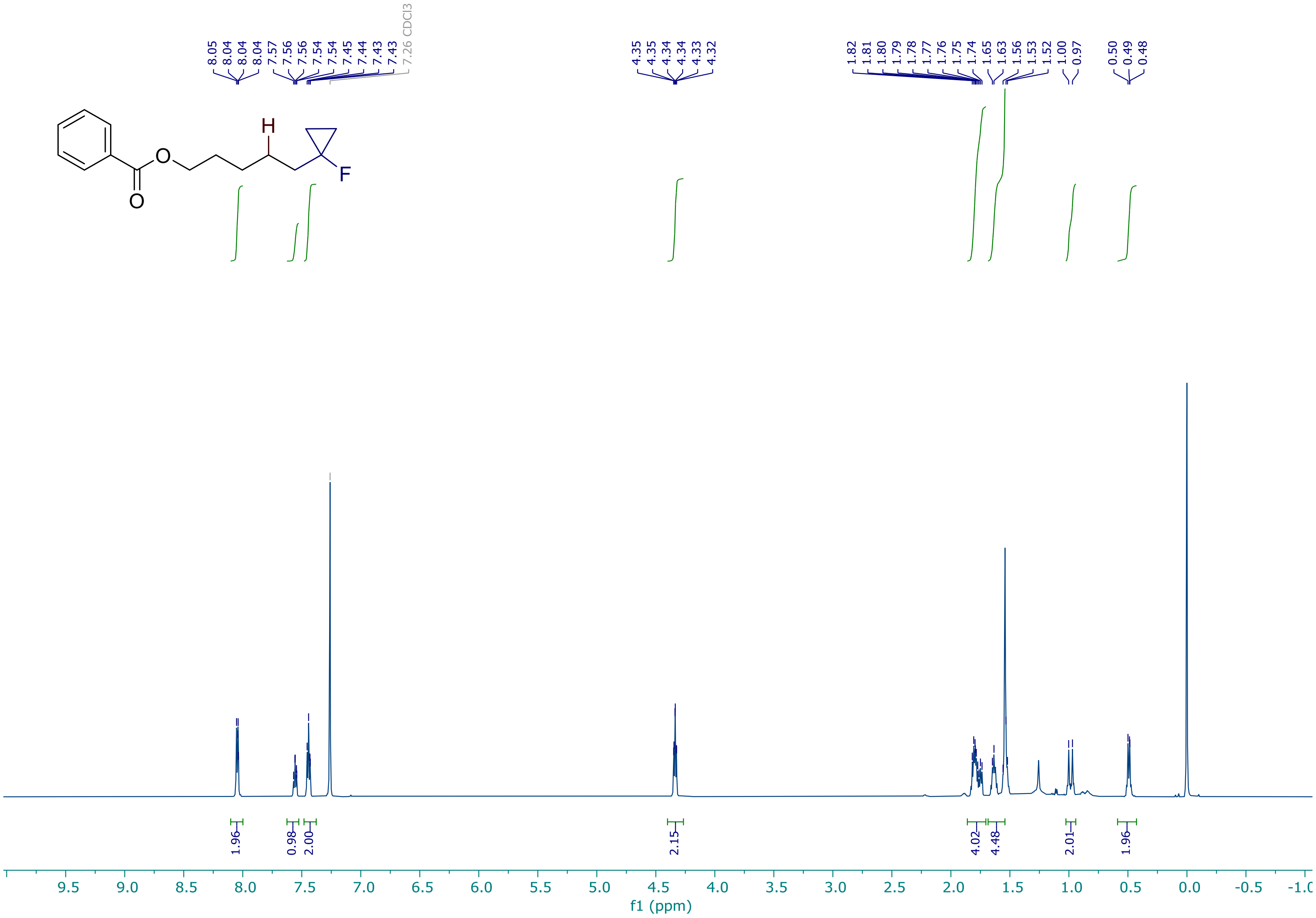
8.05
8.04
8.04
8.04
7.57
7.56
7.56
7.54
7.54
7.45
7.44
7.43
7.43
7.26 CDCl3
4.35
4.35
4.34
4.34
4.33
4.32
1.82
1.81
1.80
1.79
1.78
1.77
1.76
1.75
1.74
1.65
1.63
1.56
1.53
1.52
1.00
0.97
0.50
0.49
0.48
H
O
O
F
1.96
0.98
2.00
2.15
4.02
4.48
2.01
1.96
9.5
9.0
8.5
8.0
7.5
7.0
6.5
6.0
5.5
5.0
4.5
4.0
3.5
3.0
2.5
2.0
1.5
1.0
0.5
0.0
-0.5
f1 (ppm)

^{13}C NMR of **31**

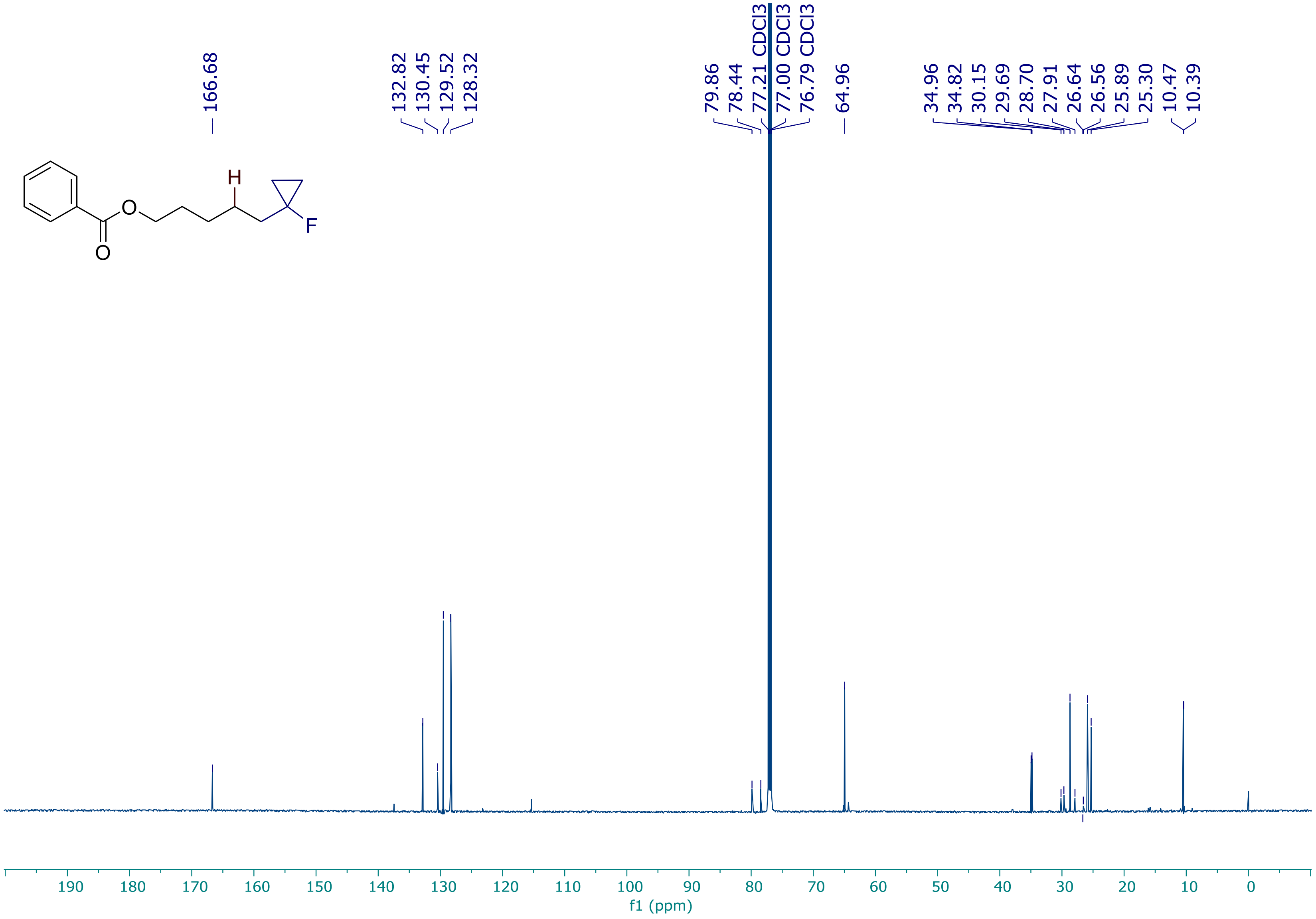

166.68
132.82
130.45
129.52
128.32
79.86
78.44
77.21 CDCl3
77.00 CDCl3
76.79 CDCl3
64.96
34.96
34.82
30.15
29.69
28.70
27.91
26.64
26.56
25.89
25.30
10.47
10.39
H
O
O
F
190
180
170
160
150
140
130
120
110
100
90
80
70
60
50
40
30
20
10
0
f1 (ppm)

^{19}F NMR of **31**

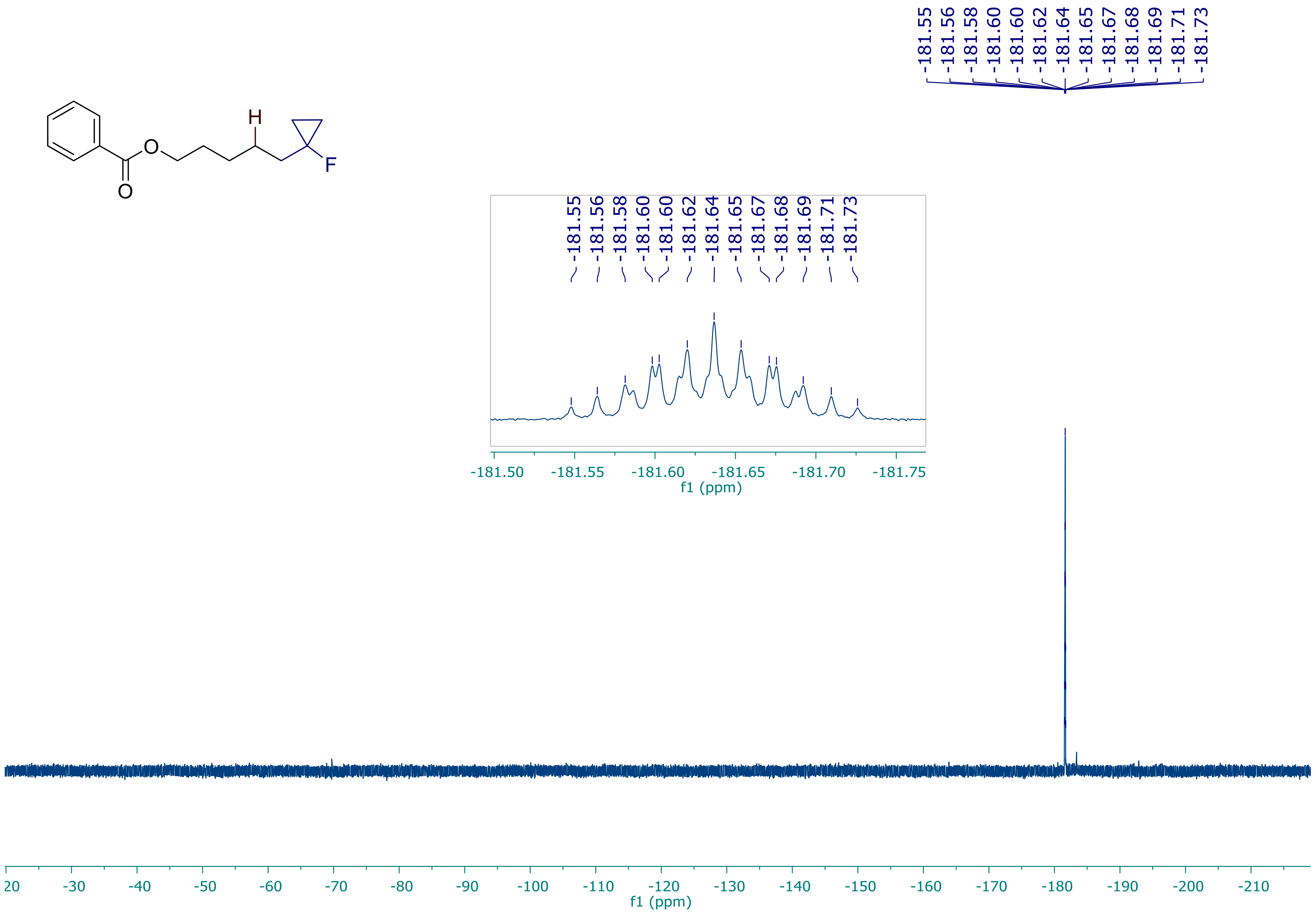

H
O
O
F
-181.55
-181.56
-181.58
-181.60
-181.60
-181.62
-181.64
-181.65
-181.67
-181.68
-181.69
-181.71
-181.73
-181.50
-181.55
-181.60
-181.65
-181.70
-181.75
f1 (ppm)
20
-30
-40
-50
-60
-70
-80
-90
-100
-110
-120
-130
-140
-150
-160
-170
-180
-190
-200
-210
f1 (ppm)

^{1}H NMR of **32**

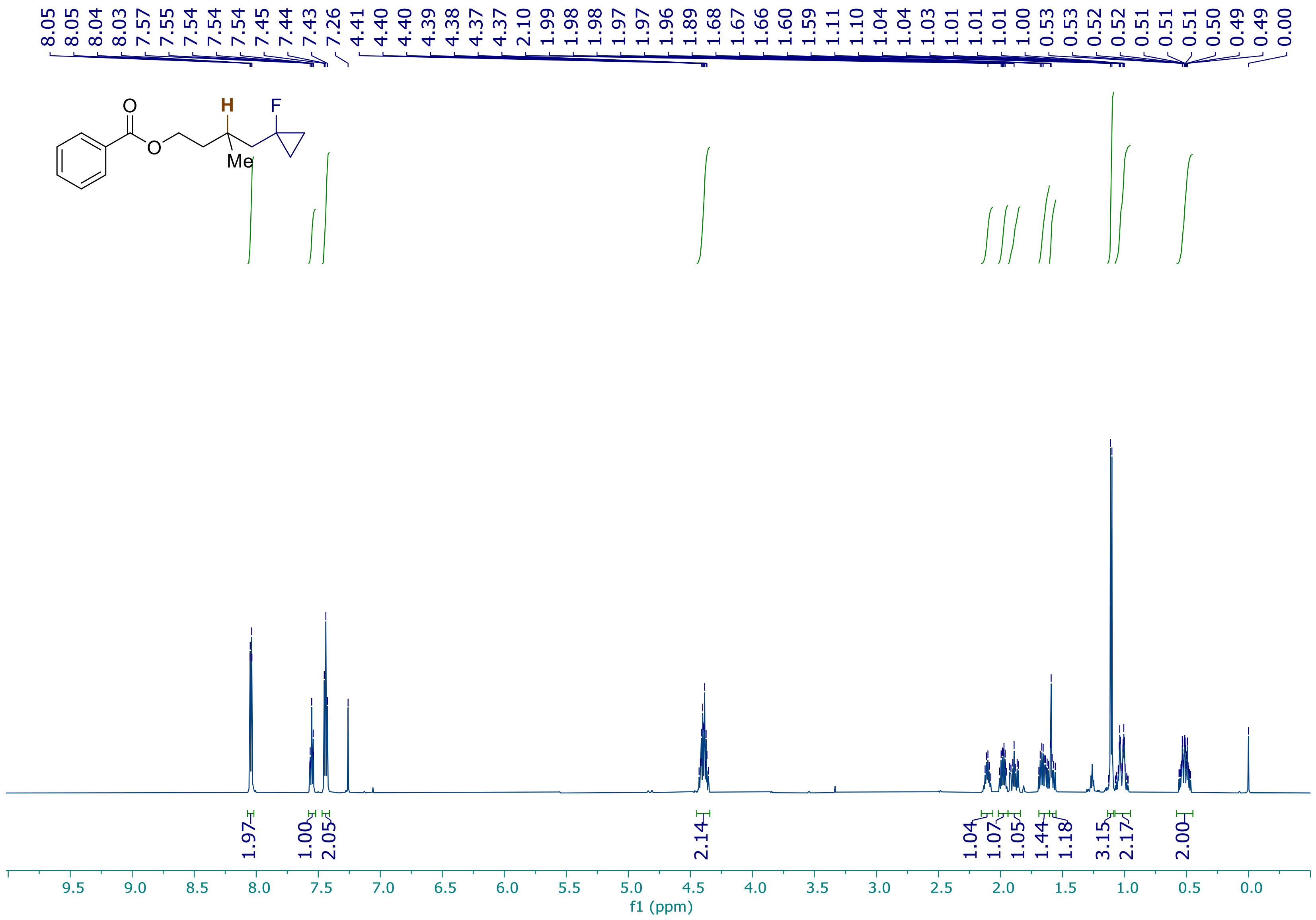

^{13}C NMR of **32**

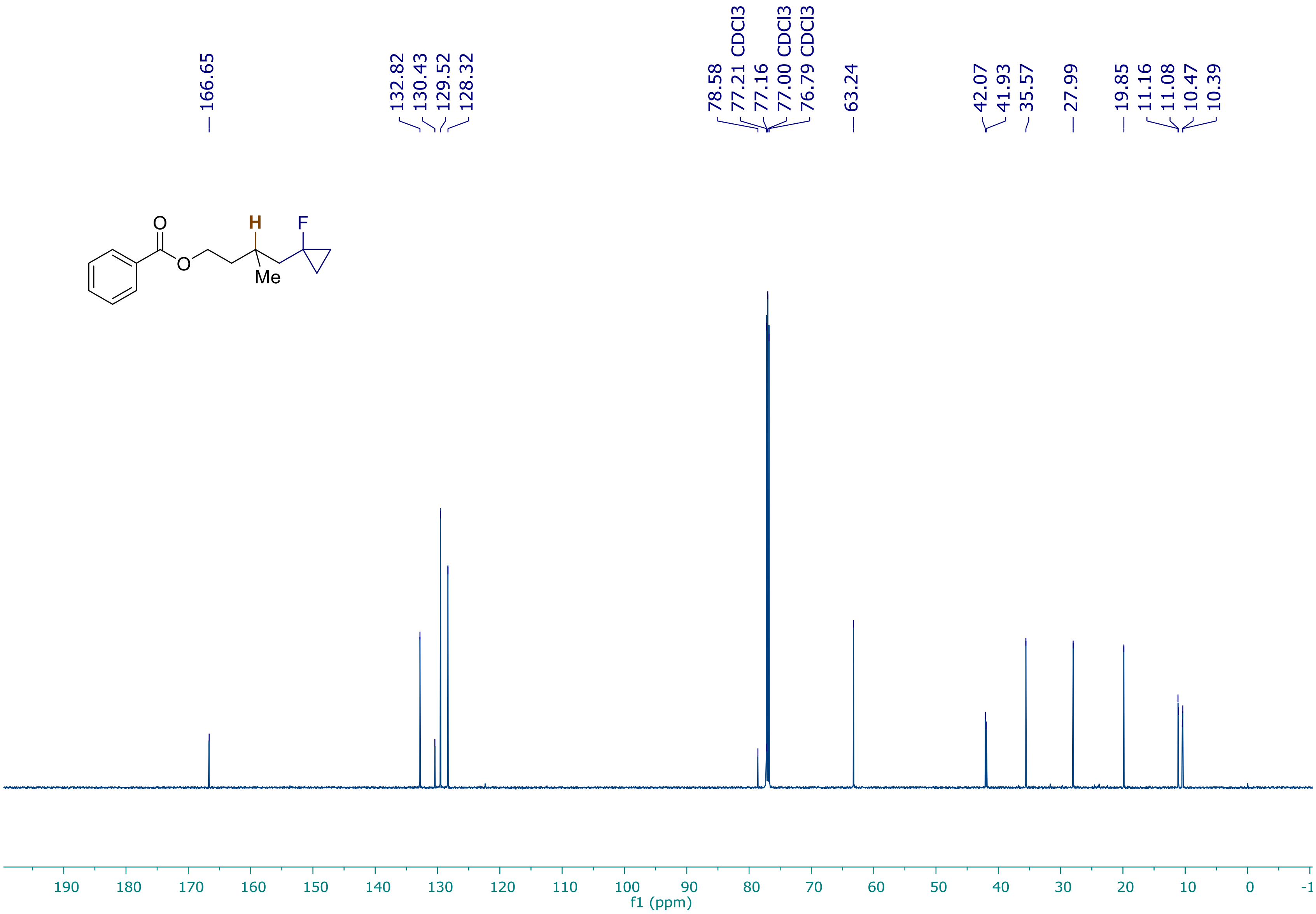
166.65
132.82
130.43
129.52
128.32
78.58
77.21 CDCl3
77.16
77.00 CDCl3
76.79 CDCl3
63.24
42.07
41.93
35.57
27.99
19.85
11.16
11.08
10.47
10.39
O
H
F
O
Me
190
180
170
160
150
140
130
120
110
100
90
80
70
60
50
40
30
20
10
0
-1
f1 (ppm)

^{19}F NMR of **32**

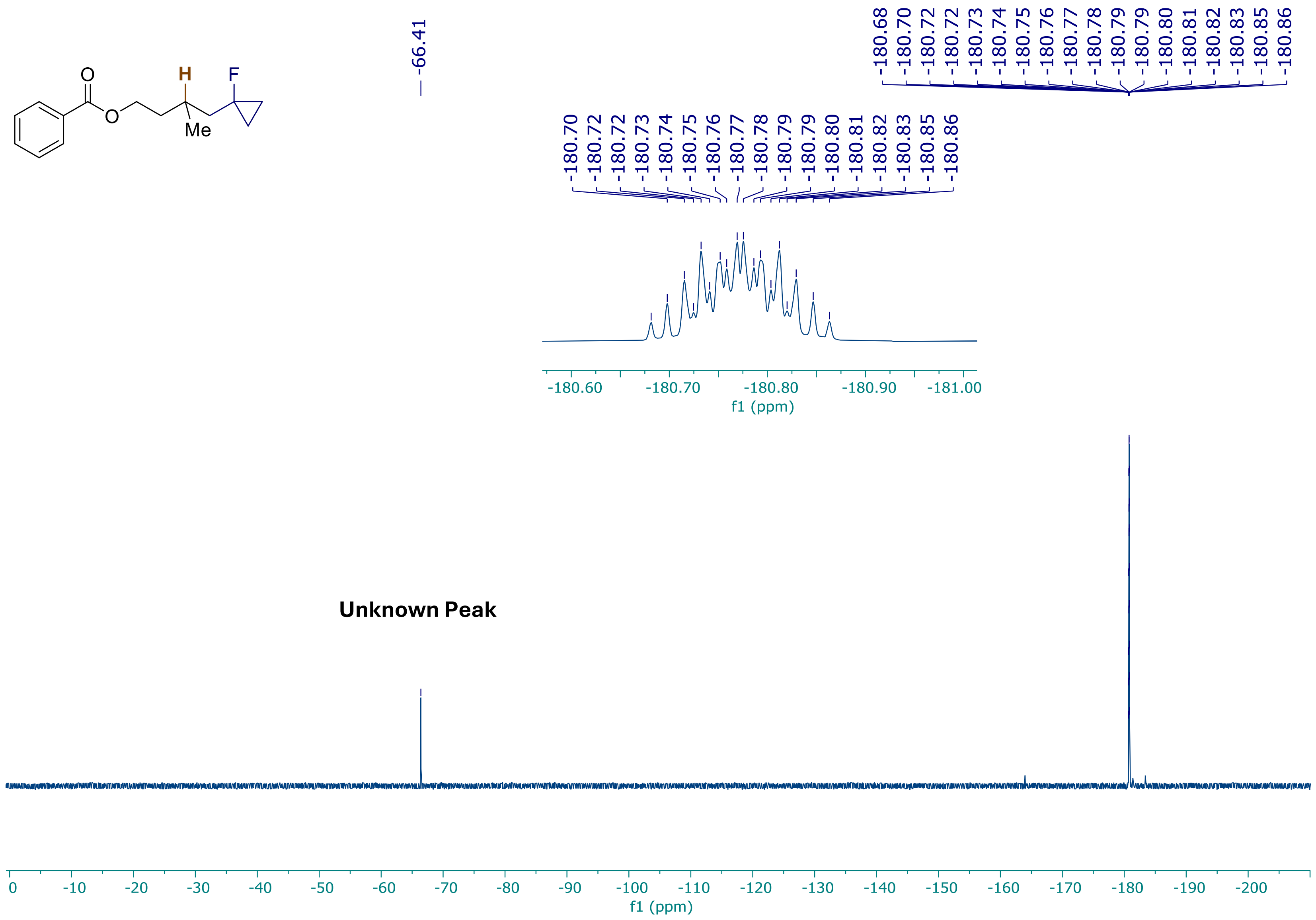

O
O
H
F
Me
-66.41
-180.68
-180.70
-180.72
-180.72
-180.73
-180.74
-180.75
-180.76
-180.77
-180.78
-180.79
-180.79
-180.80
-180.81
-180.82
-180.83
-180.85
-180.86
-180.70
-180.72
-180.72
-180.73
-180.74
-180.75
-180.76
-180.77
-180.78
-180.79
-180.80
-180.81
-180.82
-180.83
-180.85
-180.86
-180.60 -180.70 -180.80 -180.90 -181.00
f1 (ppm)
Unknown Peak
0 -10 -20 -30 -40 -50 -60 -70 -80 -90 -100 -110 -120 -130 -140 -150 -160 -170 -180 -190 -200
f1 (ppm)

^{1}H NMR of **33**

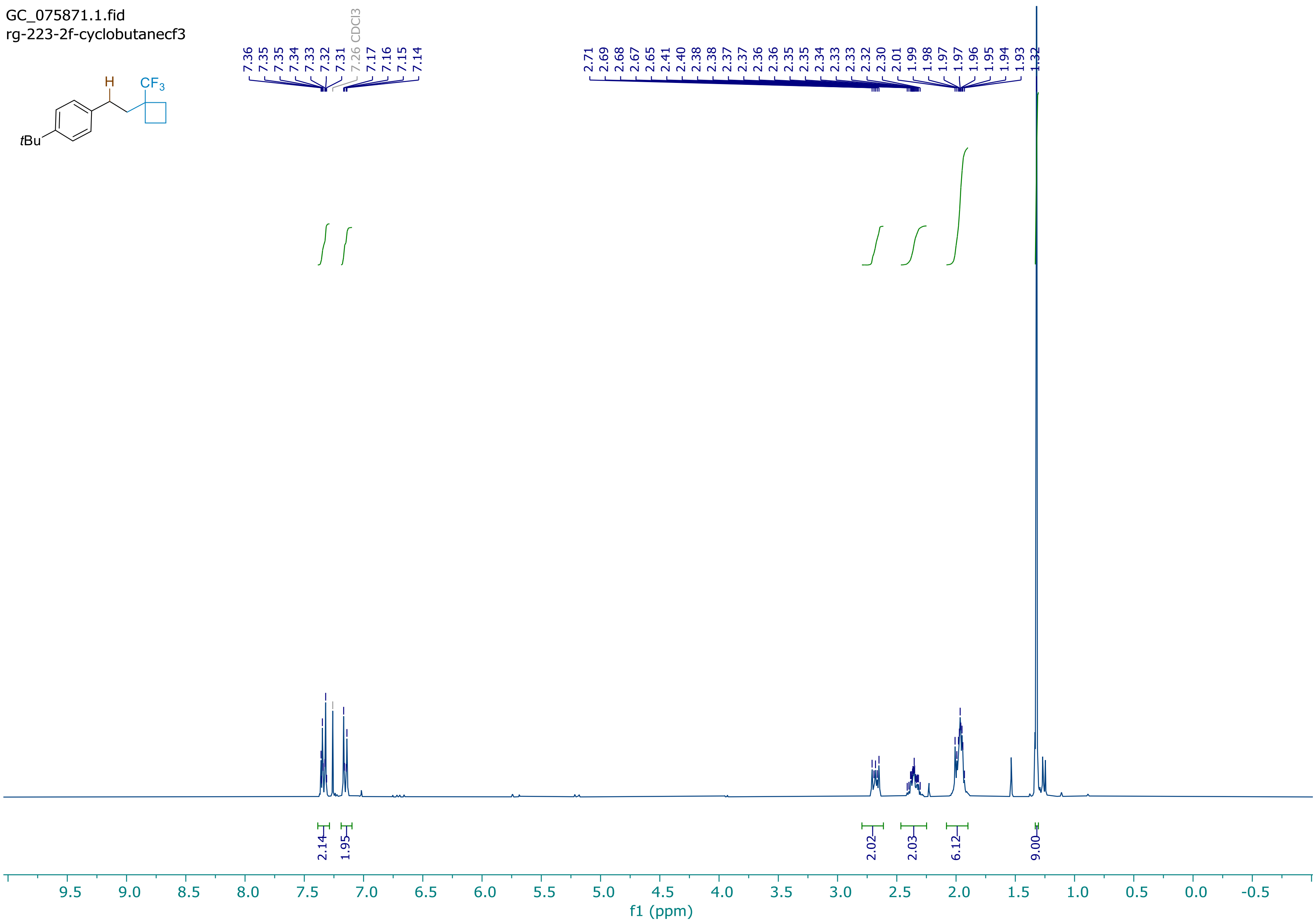

GC_075871.1.fid
rg-223-2f-cyclobutanecf3
H
CF3
tBu
7.36
7.35
7.35
7.34
7.33
7.32
7.31
7.26 CDCl3
7.17
7.16
7.15
7.14
2.71
2.69
2.68
2.67
2.65
2.41
2.40
2.38
2.38
2.37
2.37
2.36
2.36
2.35
2.35
2.34
2.33
2.33
2.32
2.30
2.01
1.99
1.98
1.97
1.97
1.96
1.95
1.94
1.93
1.32
2.14
1.95
2.02
2.03
6.12
9.00
f1 (ppm)

^{13}C NMR of **33**

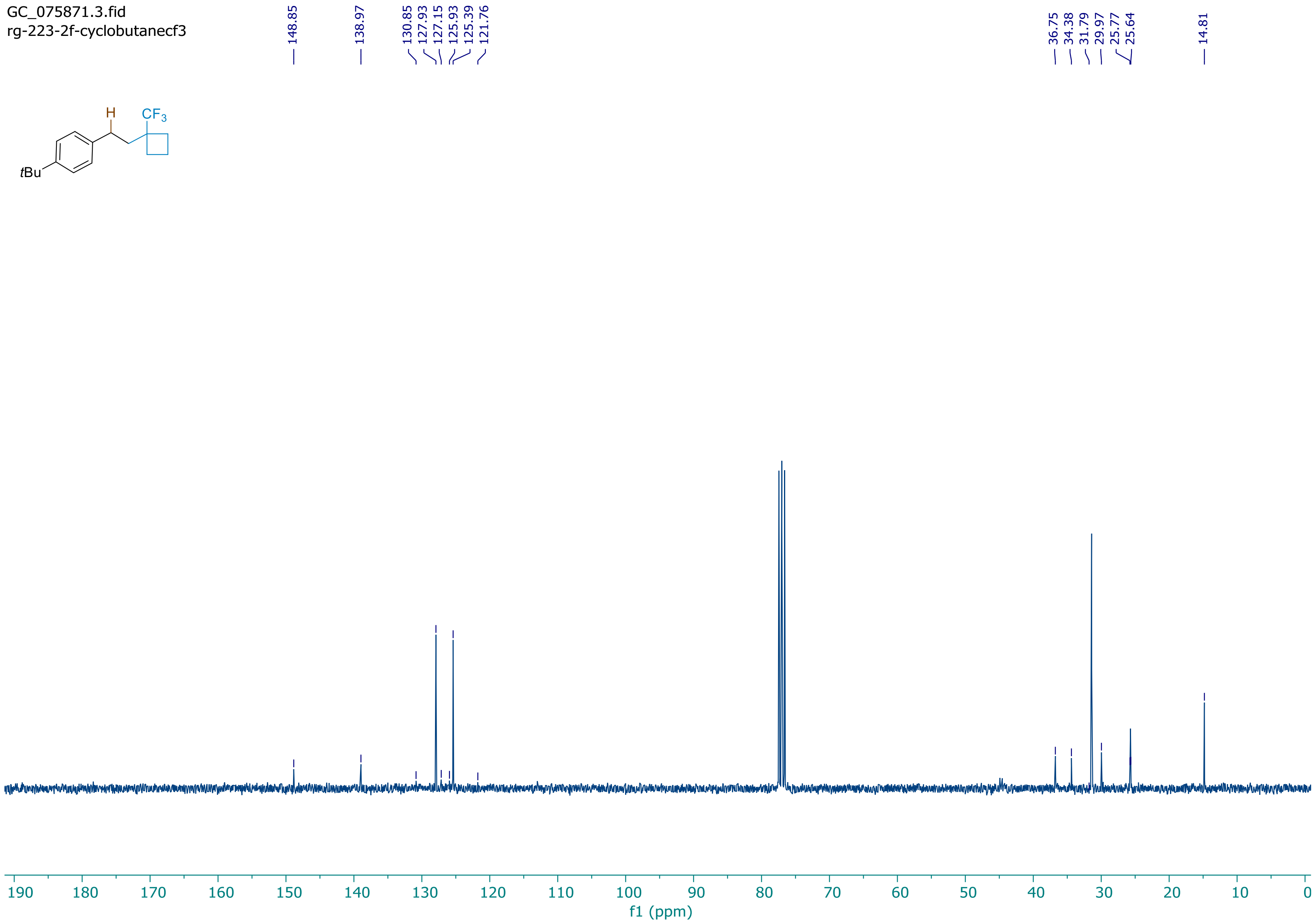
GC_075871.3.fid
rg-223-2f-cyclobutanecf3
H
CF3
tBu
148.85
138.97
130.85
127.93
127.15
125.93
125.39
121.76
36.75
34.38
31.79
29.97
25.77
25.64
14.81
190
180
170
160
150
140
130
120
110
100
90
80
70
60
50
40
30
20
10
0
f1 (ppm)

^{19}F NMR of **33**

GC_075871.2.fid
rg-223-2f-cyclobutanecf3

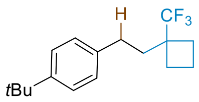

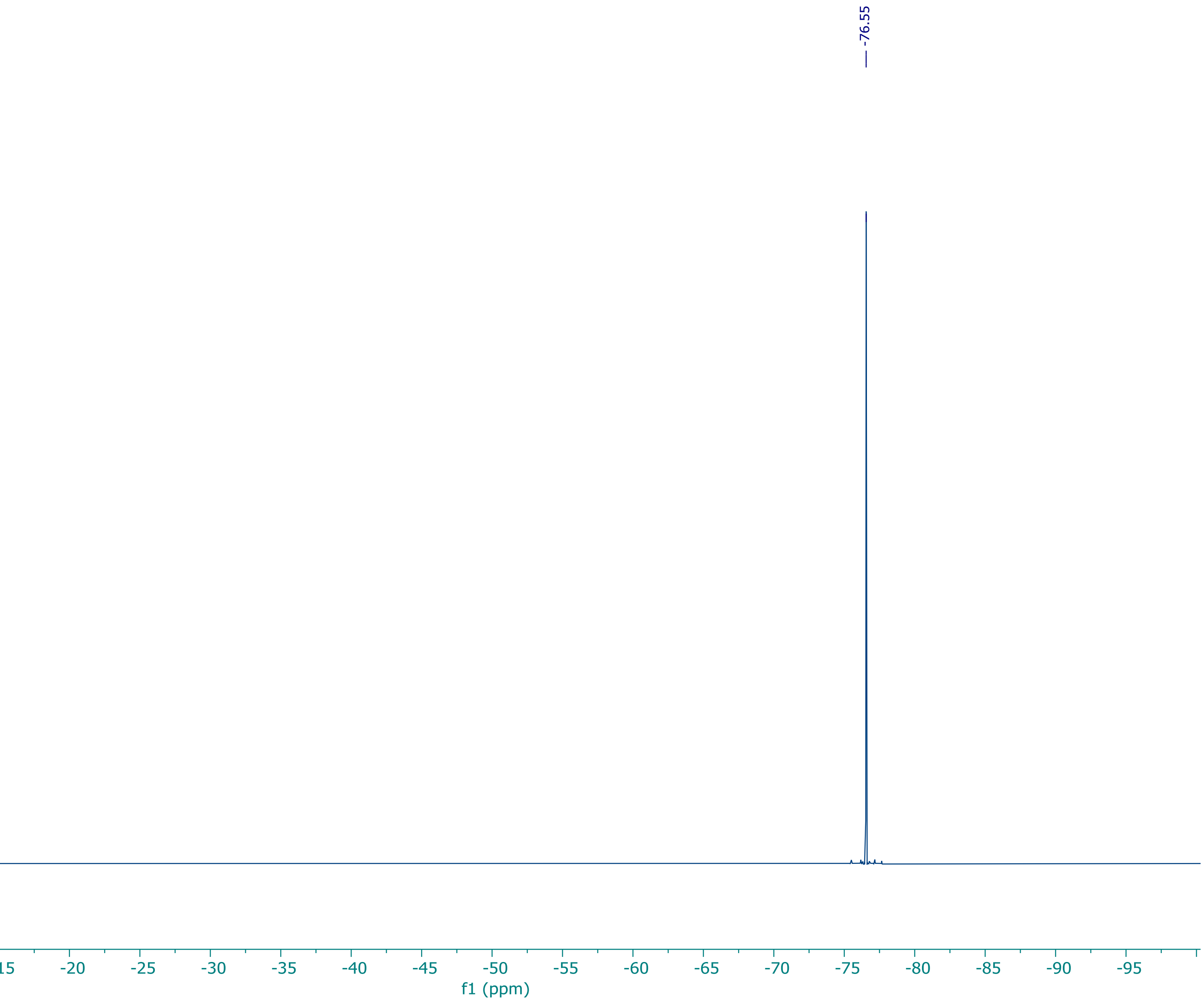

^{1}H NMR of **34**

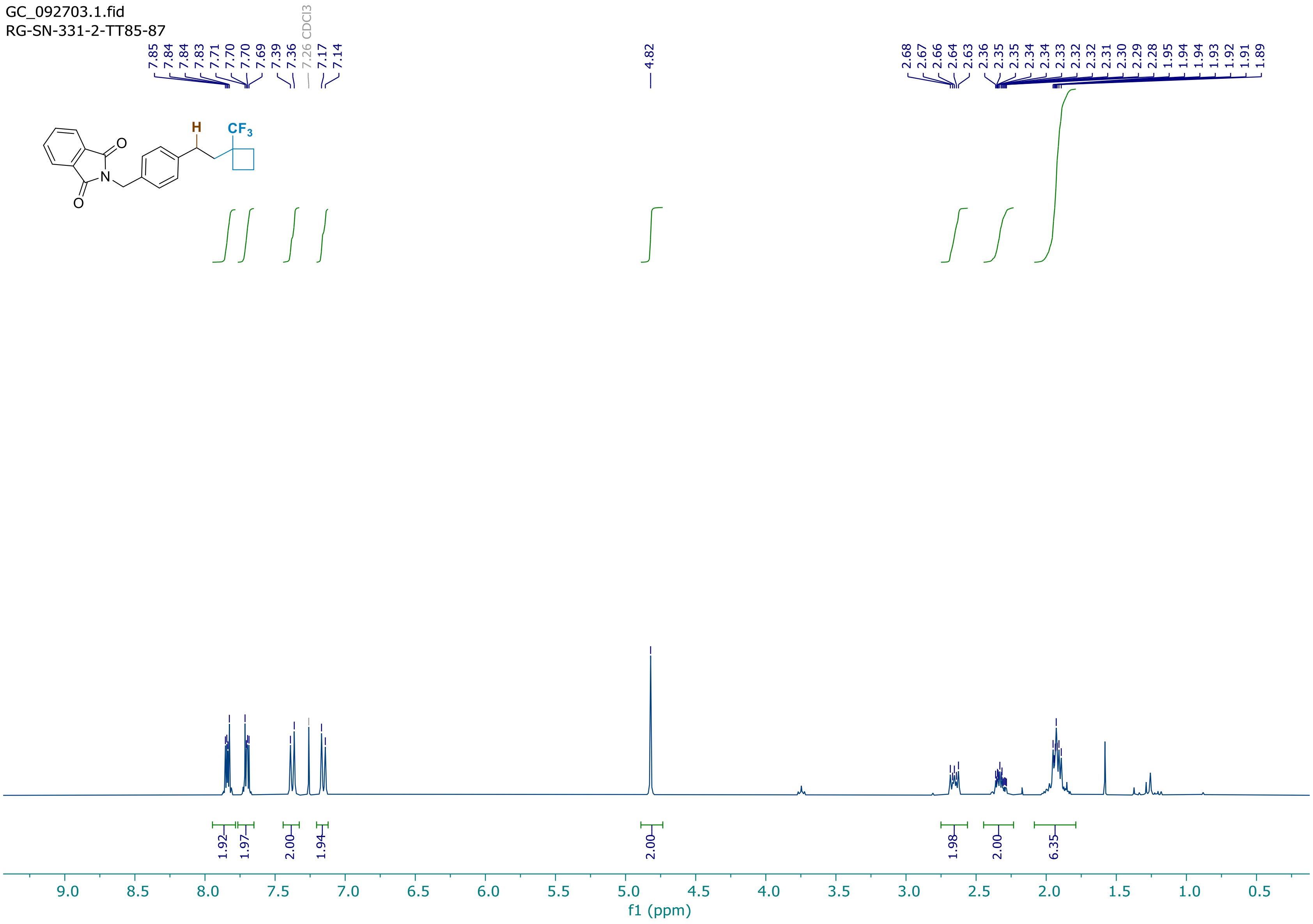

^{13}C NMR of **34**

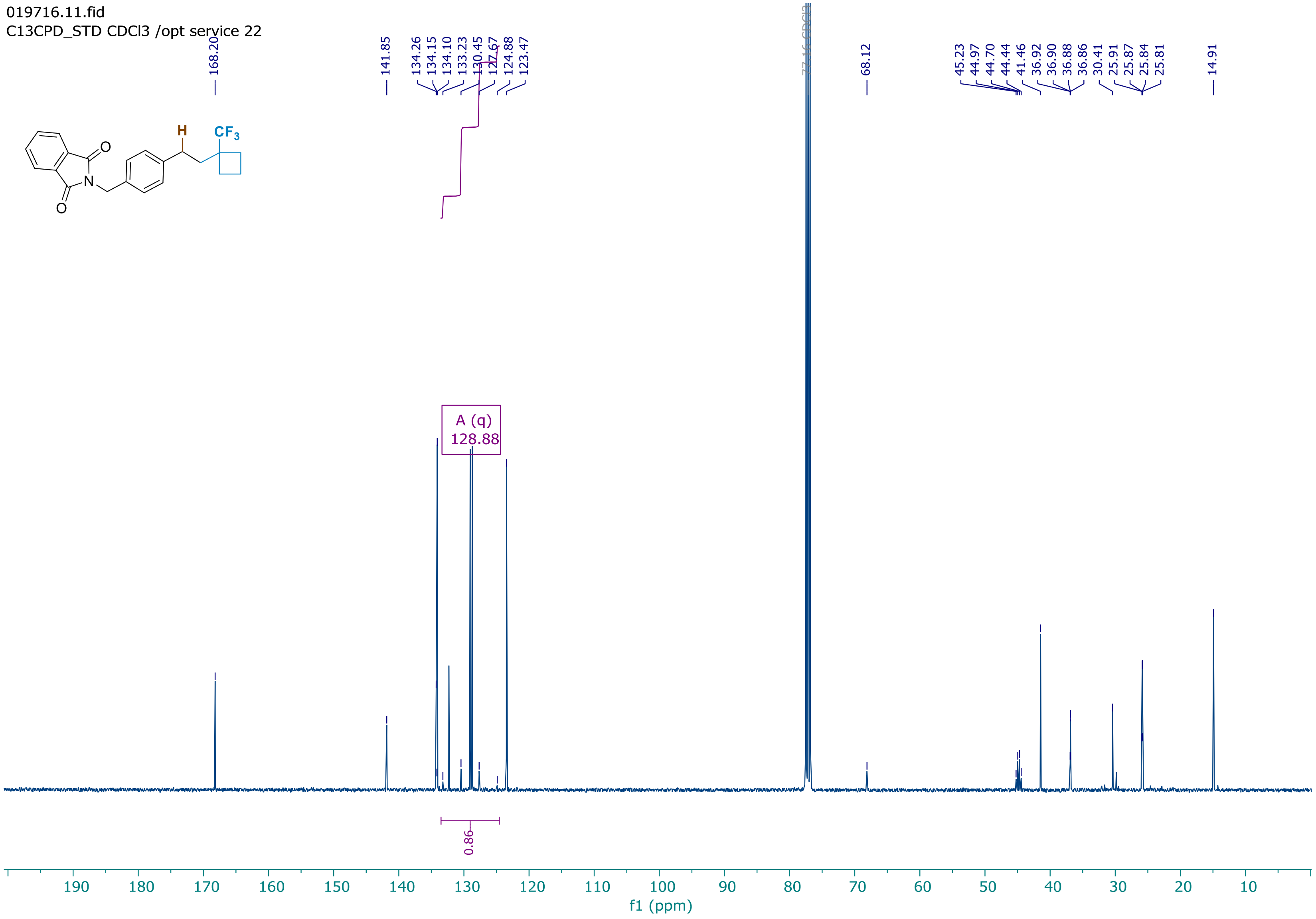

^{19}F NMR of **34**

GC_092703.2.fid
RG-SN-331-2-TT85-87

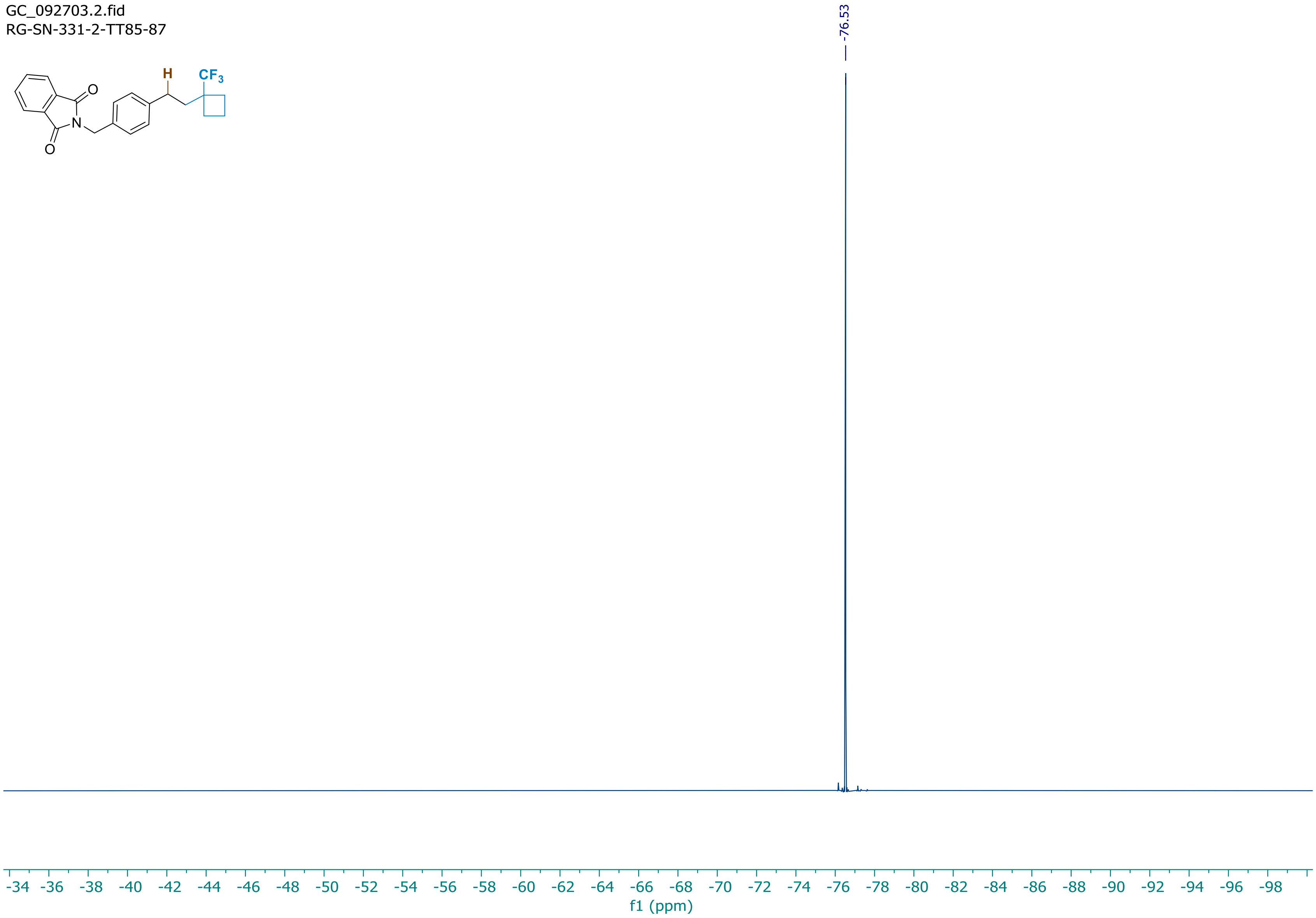

^{1}H NMR of **35**

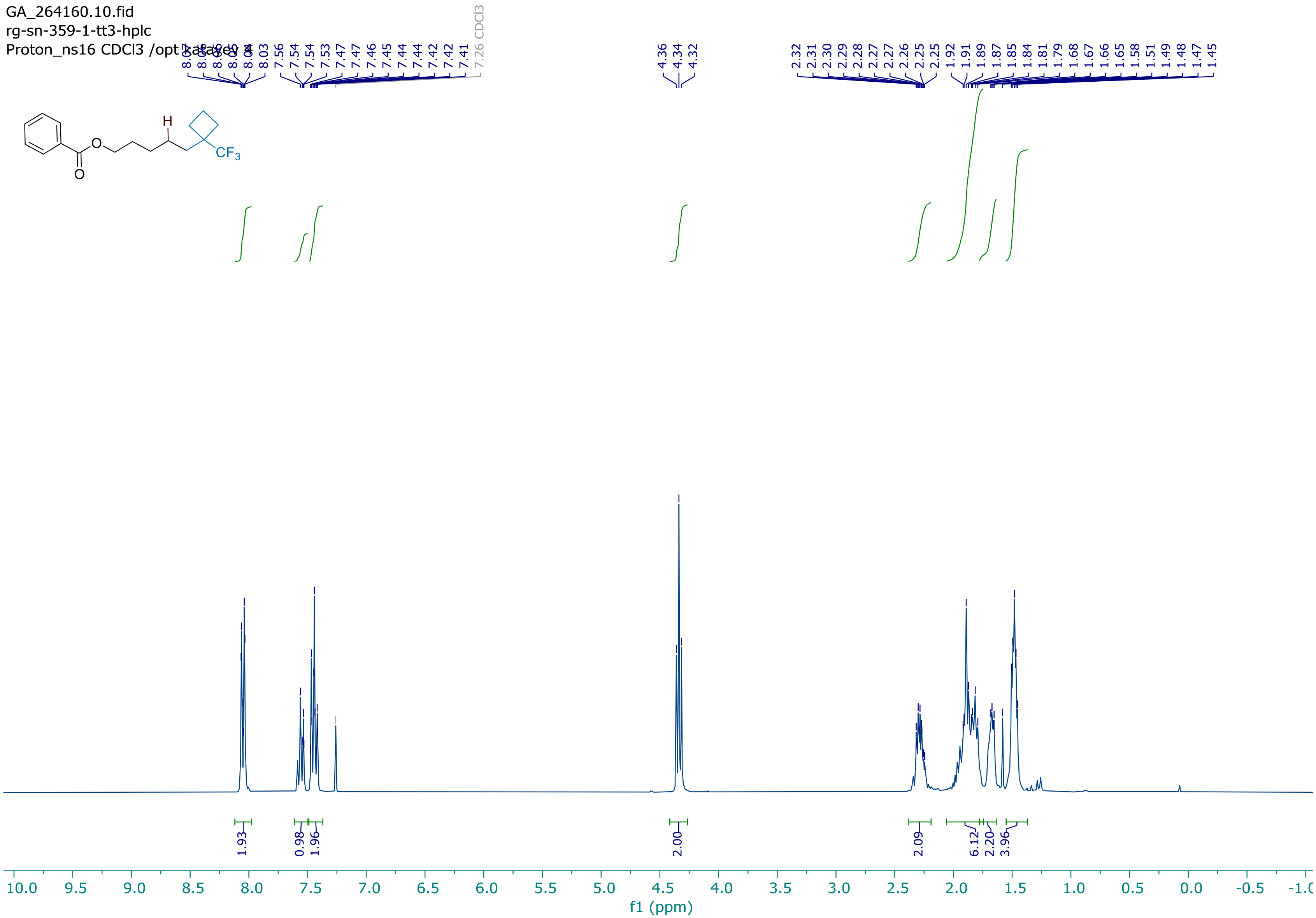

GA_264160.10.fid
rg-sn-359-1-tt3-hplc
Proton_ns16 CDCl3 /opt
7.26 CDCl3
1.93
0.98
1.96
2.00
2.09
6.12
2.20
3.96
f1 (ppm)

^{13}C NMR of **35**

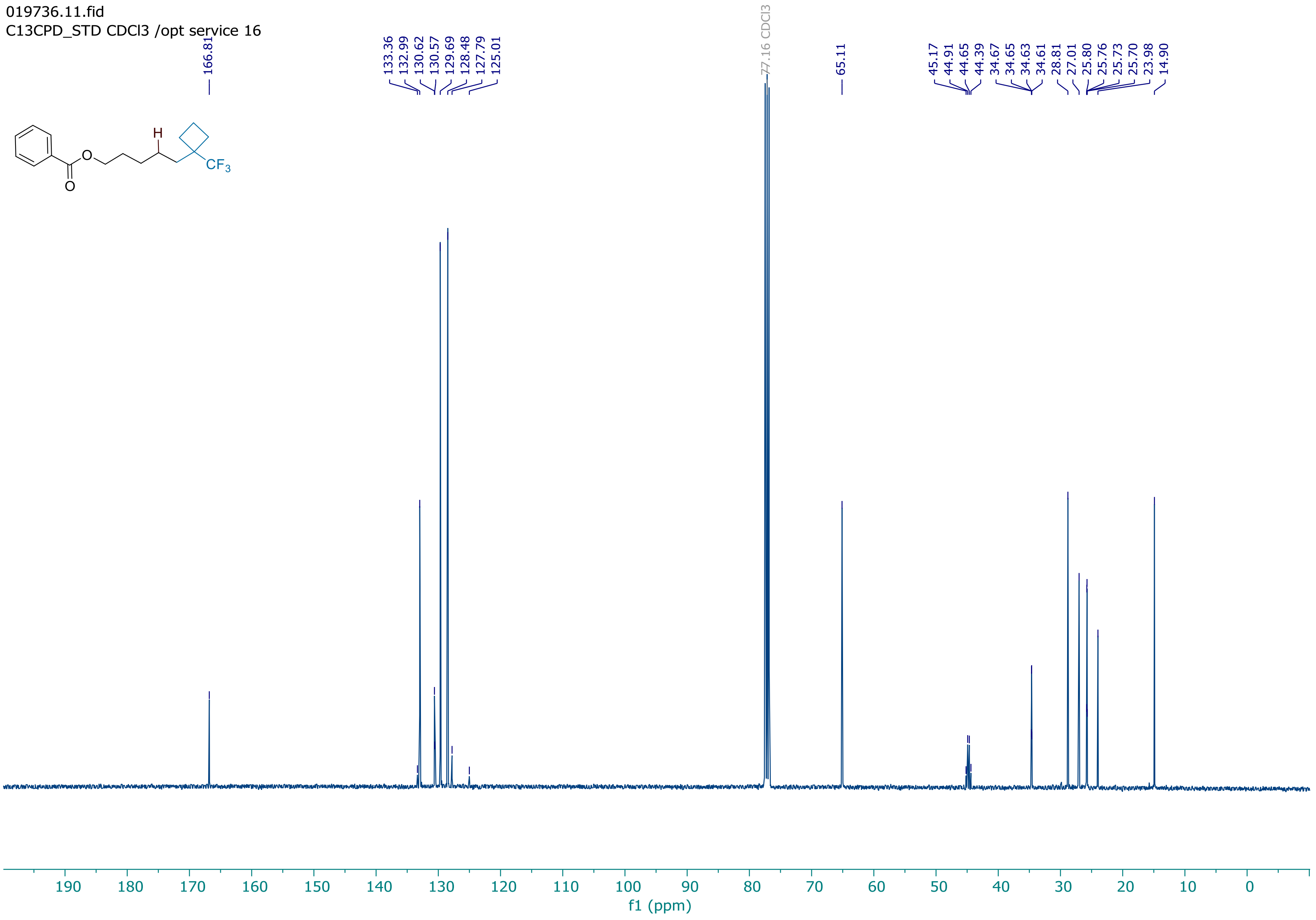
019736.11.fid
C13CPD_STD CDCl3 /opt service 16
166.81
133.36
132.99
130.62
130.57
129.69
128.48
127.79
125.01
77.16 CDCl3
65.11
45.17
44.91
44.65
44.39
34.67
34.65
34.63
34.61
28.81
27.01
25.80
25.76
25.73
25.70
23.98
14.90
H
O
O
CF3
190 180 170 160 150 140 130 120 110 100 90 80 70 60 50 40 30 20 10 0
f1 (ppm)

^{19}F NMR of **35**

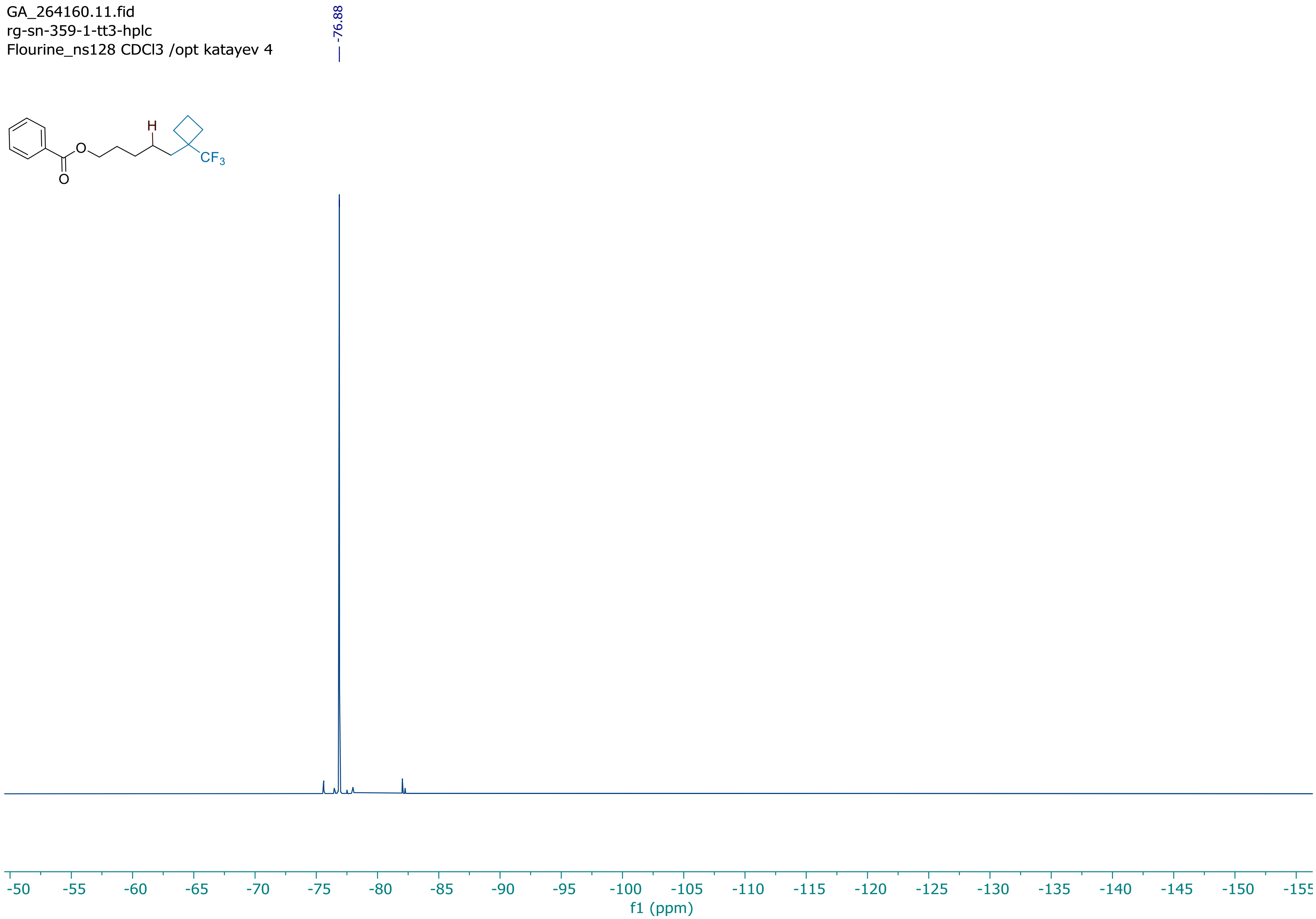
GA_264160.11.fid
rg-sn-359-1-tt3-hplc
Flourine_ns128 CDCl3 /opt katayev 4
-76.88
H
O
O
CF3
-50
-55
-60
-65
-70
-75
-80
-85
-90
-95
-100
-105
-110
-115
-120
-125
-130
-135
-140
-145
-150
f1 (ppm)

^{1}H NMR of **36**

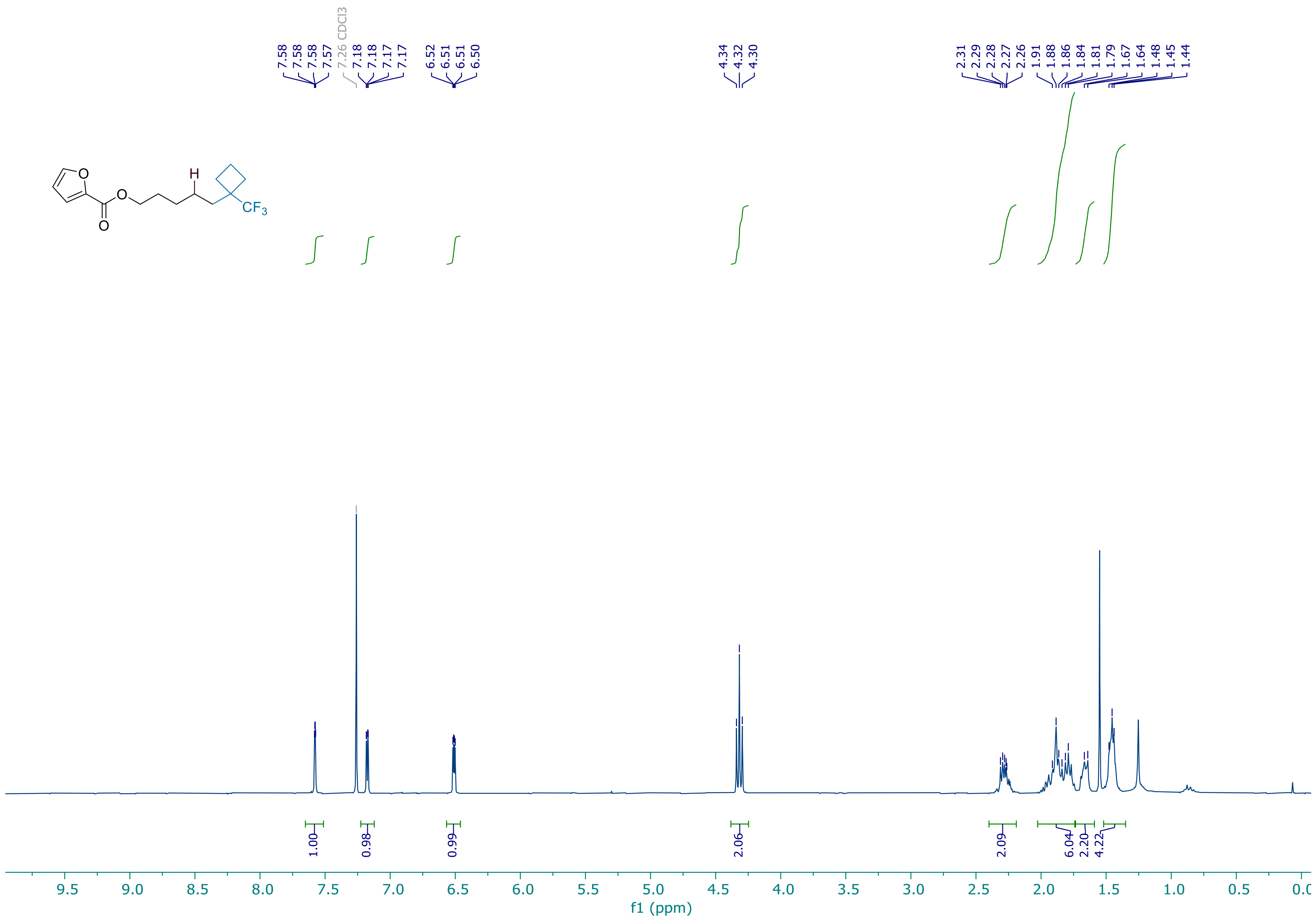

^{13}C NMR of **36**

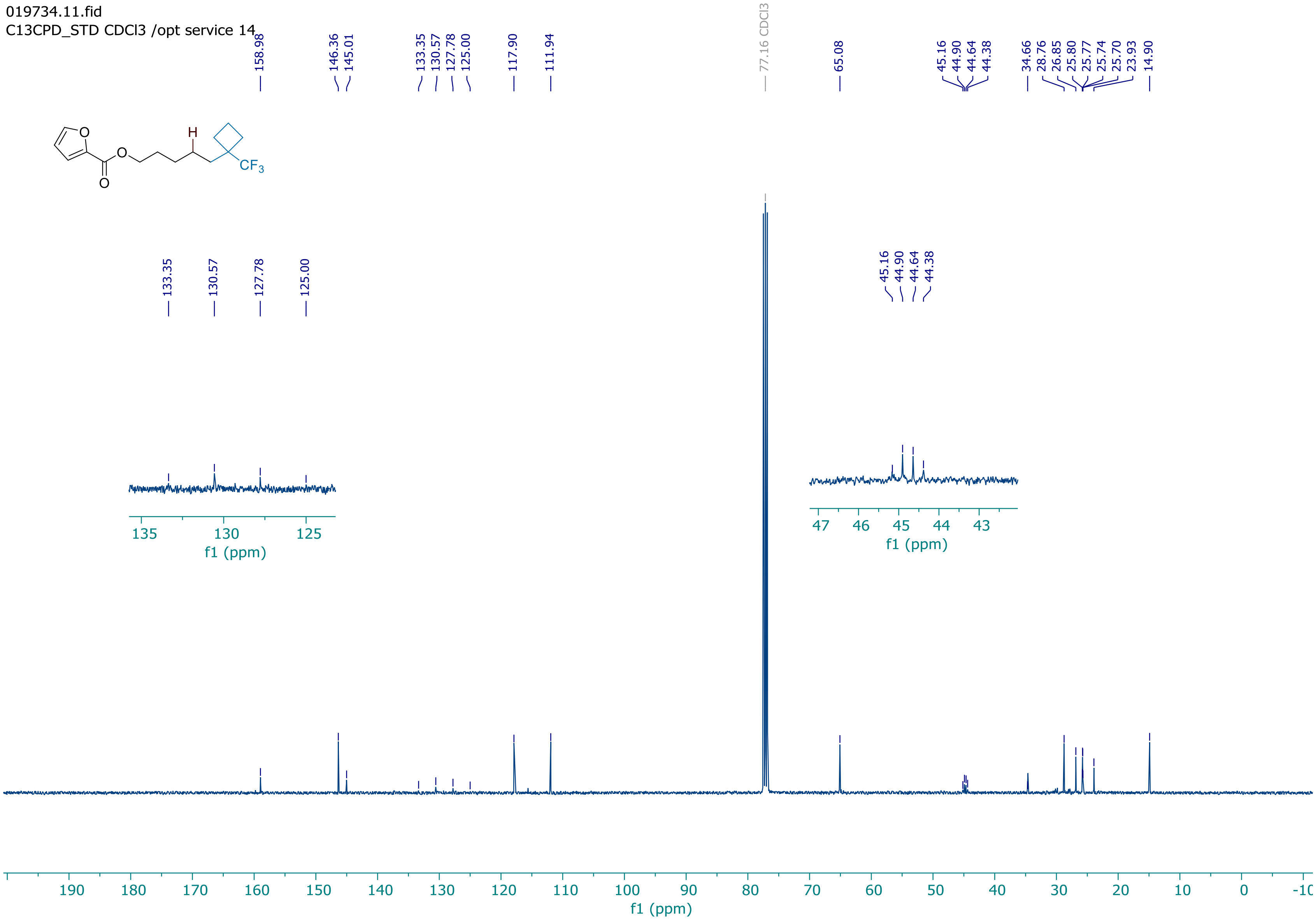
019734.11.fid
C13CPD_STD CDCl3 /opt service 14
158.98
146.36
145.01
133.35
130.57
127.78
125.00
117.90
111.94
77.16 CDCl3
65.08
45.16
44.90
44.64
44.38
34.66
28.76
26.85
25.80
25.77
25.74
25.70
23.93
14.90
O
O
O
H
CF3
f1 (ppm)

^{19}F NMR of **36**

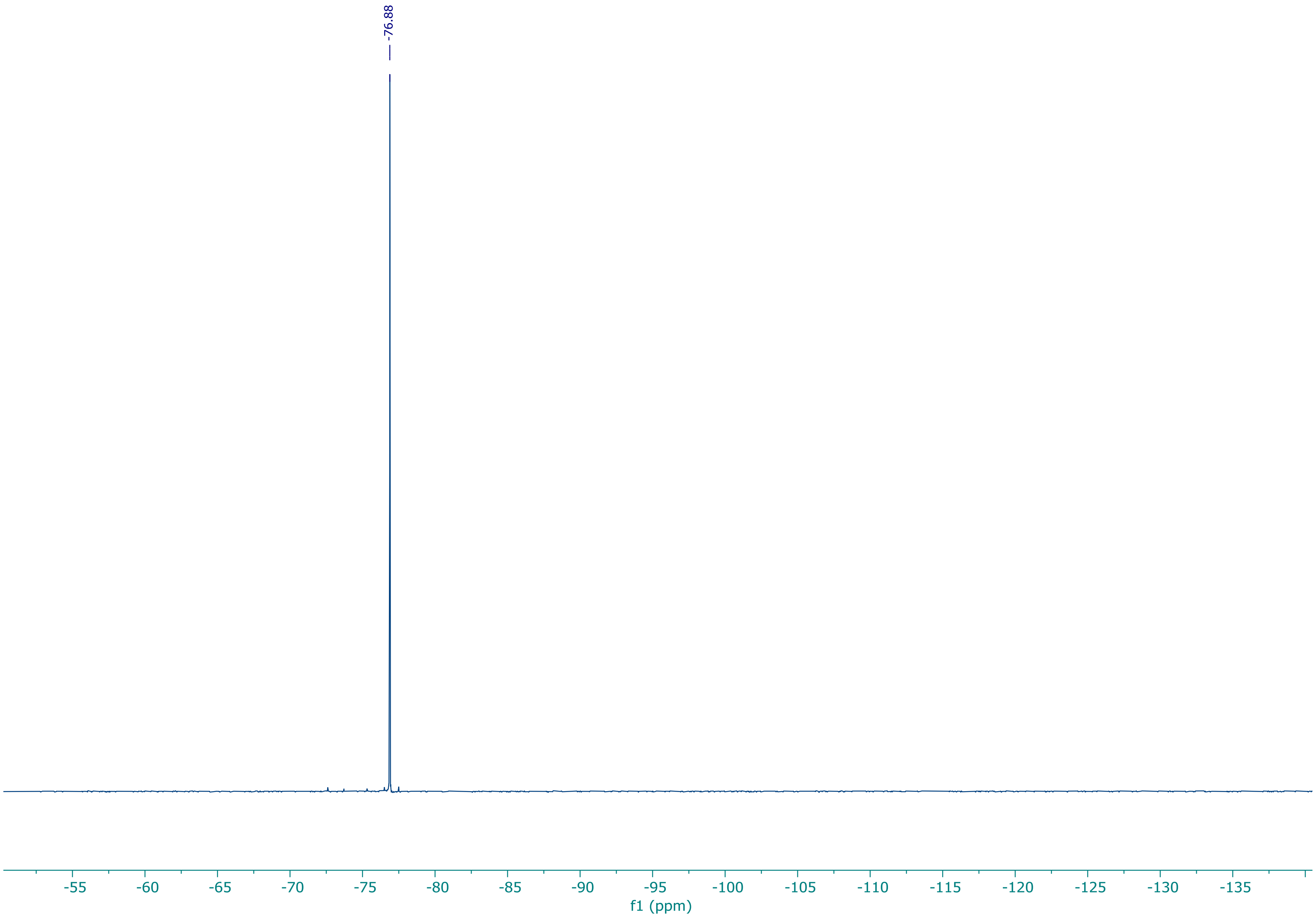

-76.88
-55
-60
-65
-70
-75
-80
-85
-90
-95
-100
-105
-110
-115
-120
-125
-130
-135
f1 (ppm)

^{1}H NMR of **37**

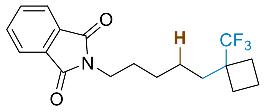

H
CF3
N
O
O

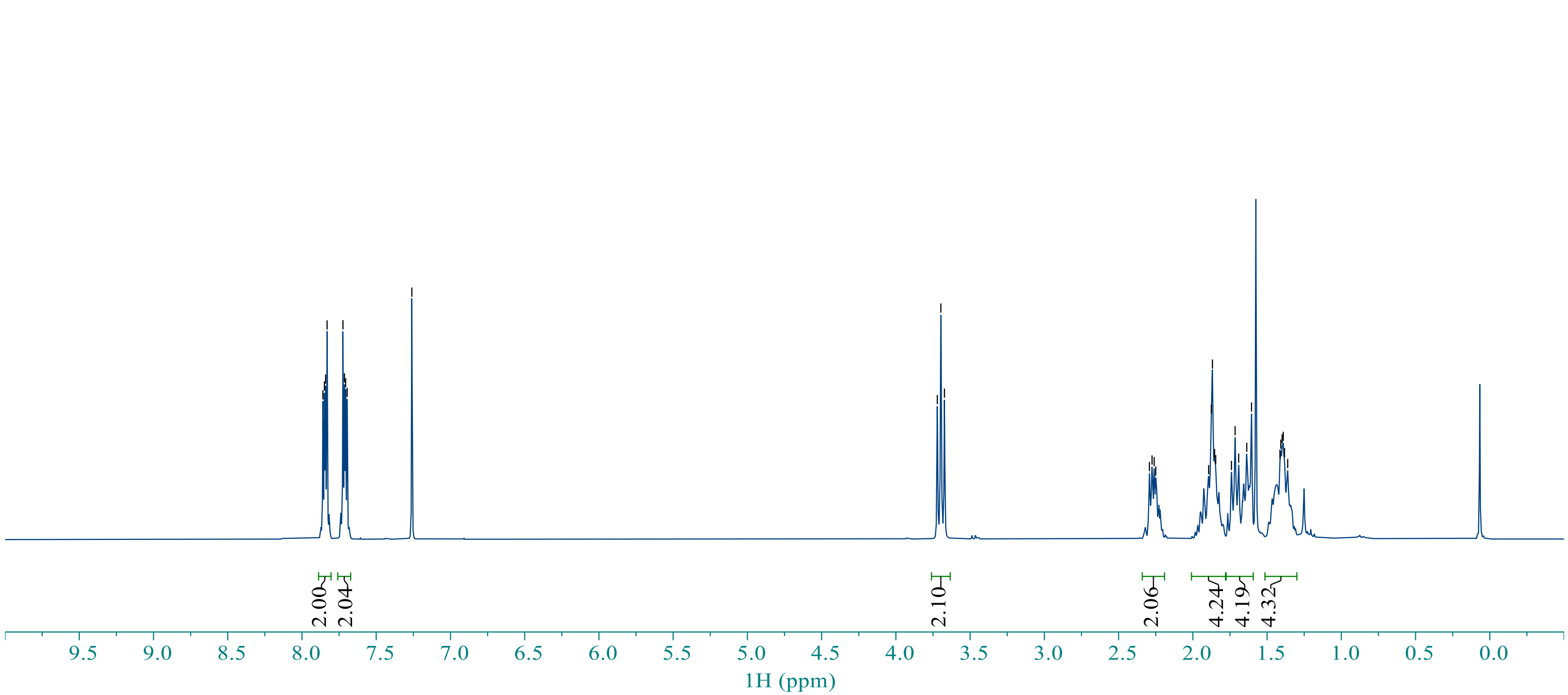

7.86
7.85
7.84
7.83
7.72
7.71
7.71
7.70
7.26 CDCl3
3.72
3.70
3.67
2.29
2.27
2.26
2.25
1.88
1.87
1.85
1.85
1.72
1.69
1.64
1.60
1.41
1.41
1.40
1.39
1.38
1.36
2.00
2.04
2.10
2.06
4.24
4.19
4.32
9.5
9.0
8.5
8.0
7.5
7.0
6.5
6.0
5.5
5.0
4.5
4.0
3.5
3.0
2.5
2.0
1.5
1.0
0.5
0.0
1H (ppm)

^{13}C NMR of **37**

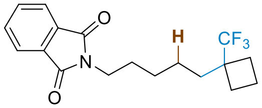
O
N
H
CF_3
O

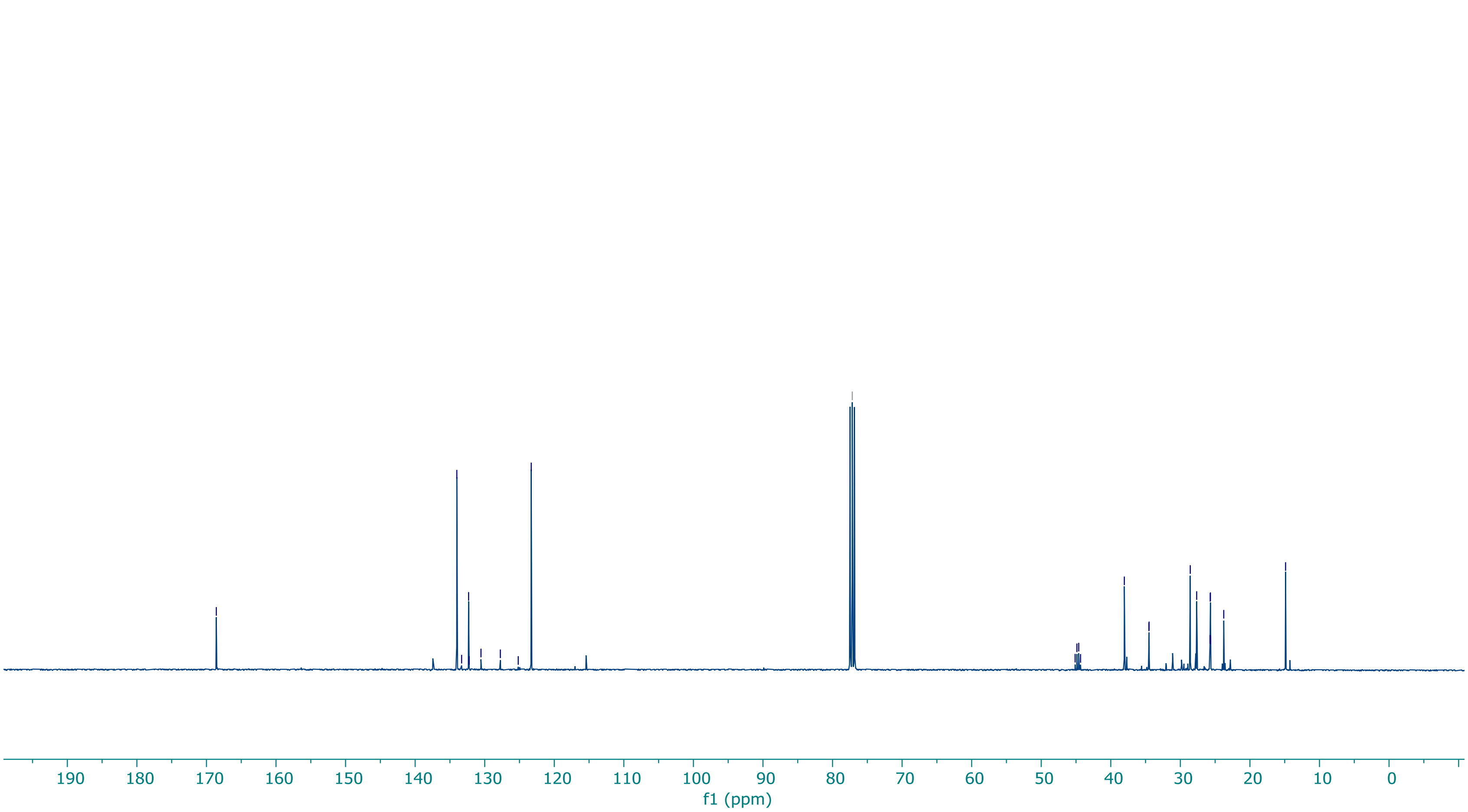
019733.11.fid
C13CPD_STD CDCl3 /opt service 13
168.58
134.01
133.32
132.31
132.24
130.54
127.75
125.18
123.31
77.16 CDCl3
45.13
44.87
44.61
44.34
38.05
34.51
34.49
28.58
27.65
25.74
25.71
25.68
25.65
23.76
14.87
190
180
170
160
150
140
130
120
110
100
90
80
70
60
50
40
30
20
10
0
f1 (ppm)

^{13}C NMR of **37**

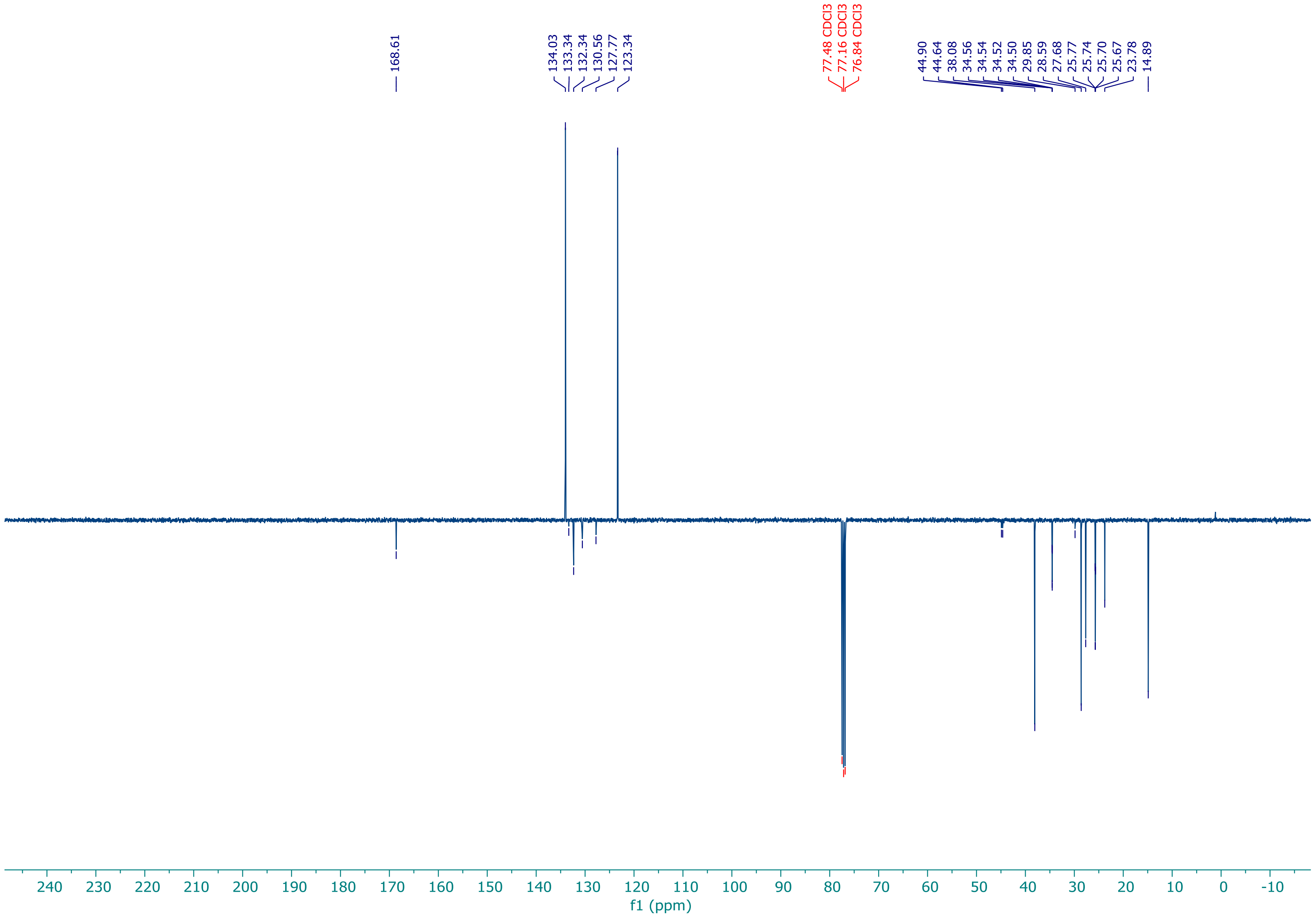
168.61
134.03
133.34
132.34
130.56
127.77
123.34
77.48 CDCl3
77.16 CDCl3
76.84 CDCl3
44.90
44.64
38.08
34.56
34.54
34.52
34.50
29.85
28.59
27.68
25.77
25.74
25.70
25.67
23.78
14.89
240 230 220 210 200 190 180 170 160 150 140 130 120 110 100 90 80 70 60 50 40 30 20 10 0 -10
f1 (ppm)

^{19}F NMR of **37**

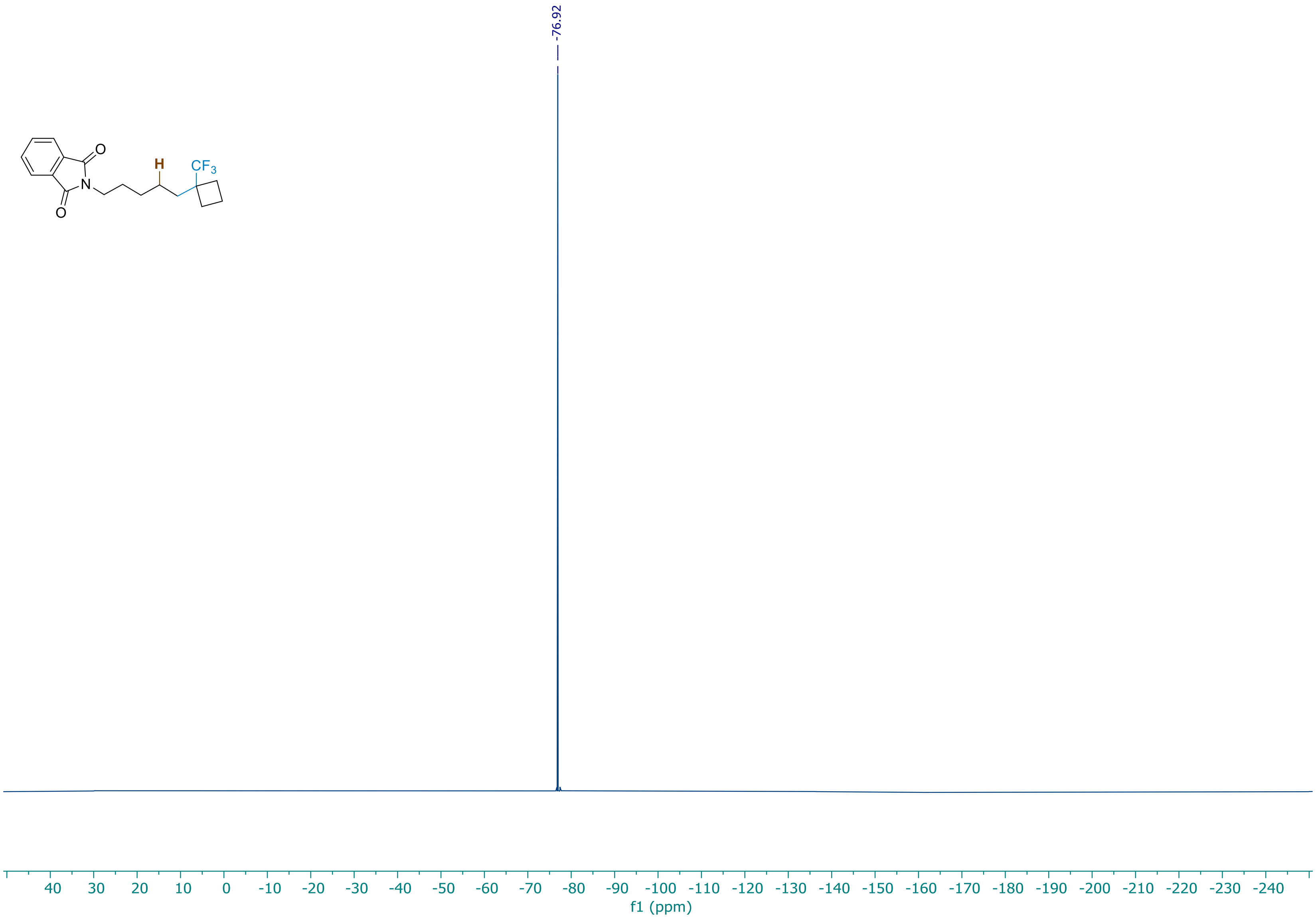

-76.92
O
N
H
CF3
O
40 30 20 10 0 -10 -20 -30 -40 -50 -60 -70 -80 -90 -100 -110 -120 -130 -140 -150 -160 -170 -180 -190 -200 -210 -220 -230 -240
f1 (ppm)

^{1}H NMR of **38**

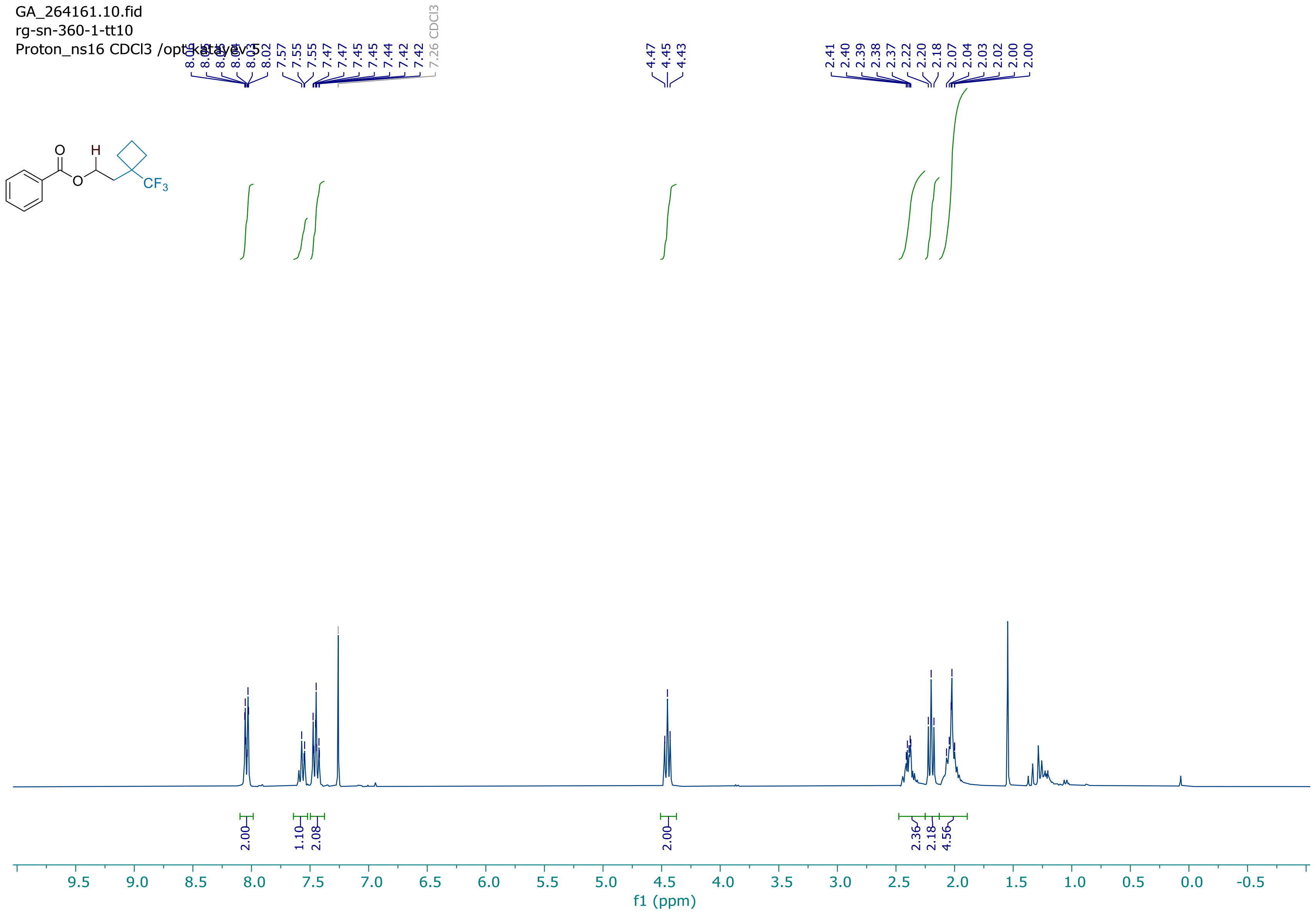
GA_264161.10.fid
rg-sn-360-1-tt10
Proton_ns16 CDCl3 /opt katayev 5
O
H
O
CF3
7.57
7.55
7.55
7.47
7.47
7.45
7.45
7.44
7.42
7.42
7.26 CDCl3
4.47
4.45
4.43
2.41
2.40
2.39
2.38
2.37
2.22
2.20
2.18
2.07
2.04
2.03
2.02
2.00
2.00
2.00
1.10
2.08
2.00
2.36
2.18
4.56
f1 (ppm)

^{13}C NMR of **38**

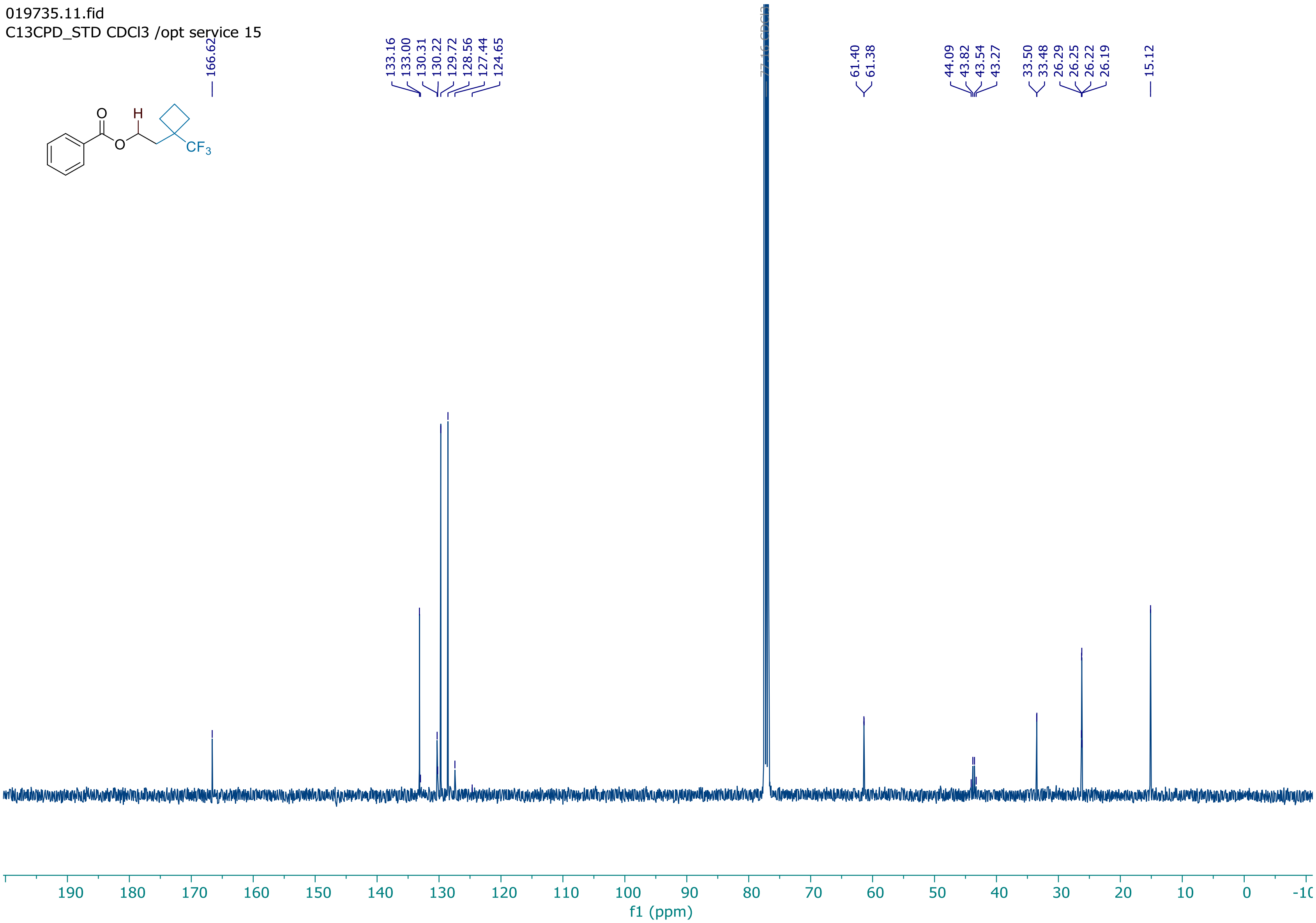
019735.11.fid
C13CPD_STD CDCl3 /opt service 15
166.62
133.16
133.00
130.31
130.22
129.72
128.56
127.44
124.65
77.16 CDCl3
61.40
61.38
44.09
43.82
43.54
43.27
33.50
33.48
26.29
26.25
26.22
26.19
15.12
O
H
O
CF3
190 180 170 160 150 140 130 120 110 100 90 80 70 60 50 40 30 20 10 0 -10
f1 (ppm)

^{19}F NMR of **38**

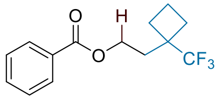

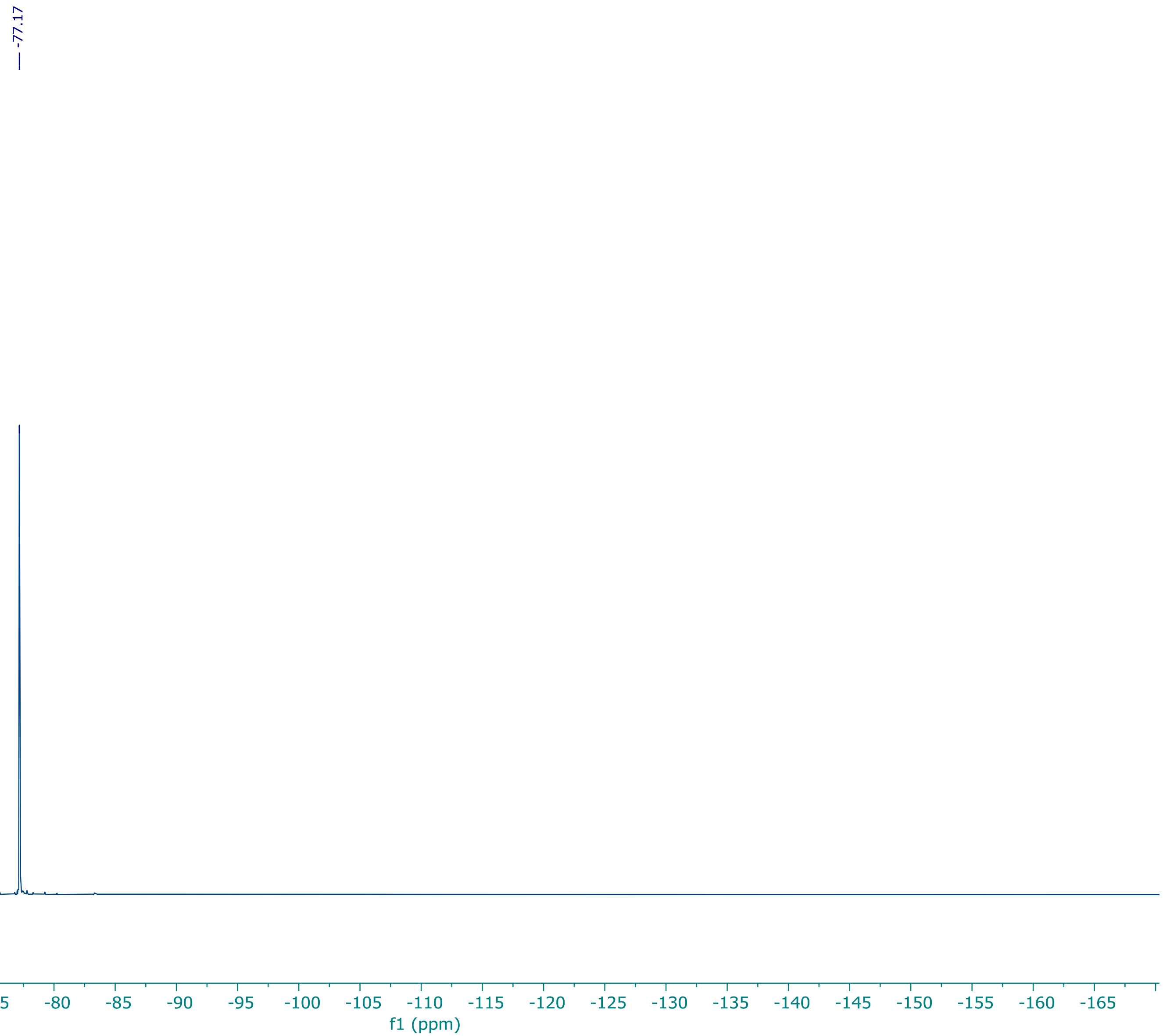
GA_264161.11.fid
rg-sn-360-1-tt10
Flourine_ns128 CDCl3 /opt katayev 5
-77.17
-55
-60
-65
-70
-75
-80
-85
-90
-95
-100
-105
-110
-115
-120
-125
-130
-135
-140
-145
-150
-155
-160
-165
f1 (ppm)

^{1}H NMR of **39**

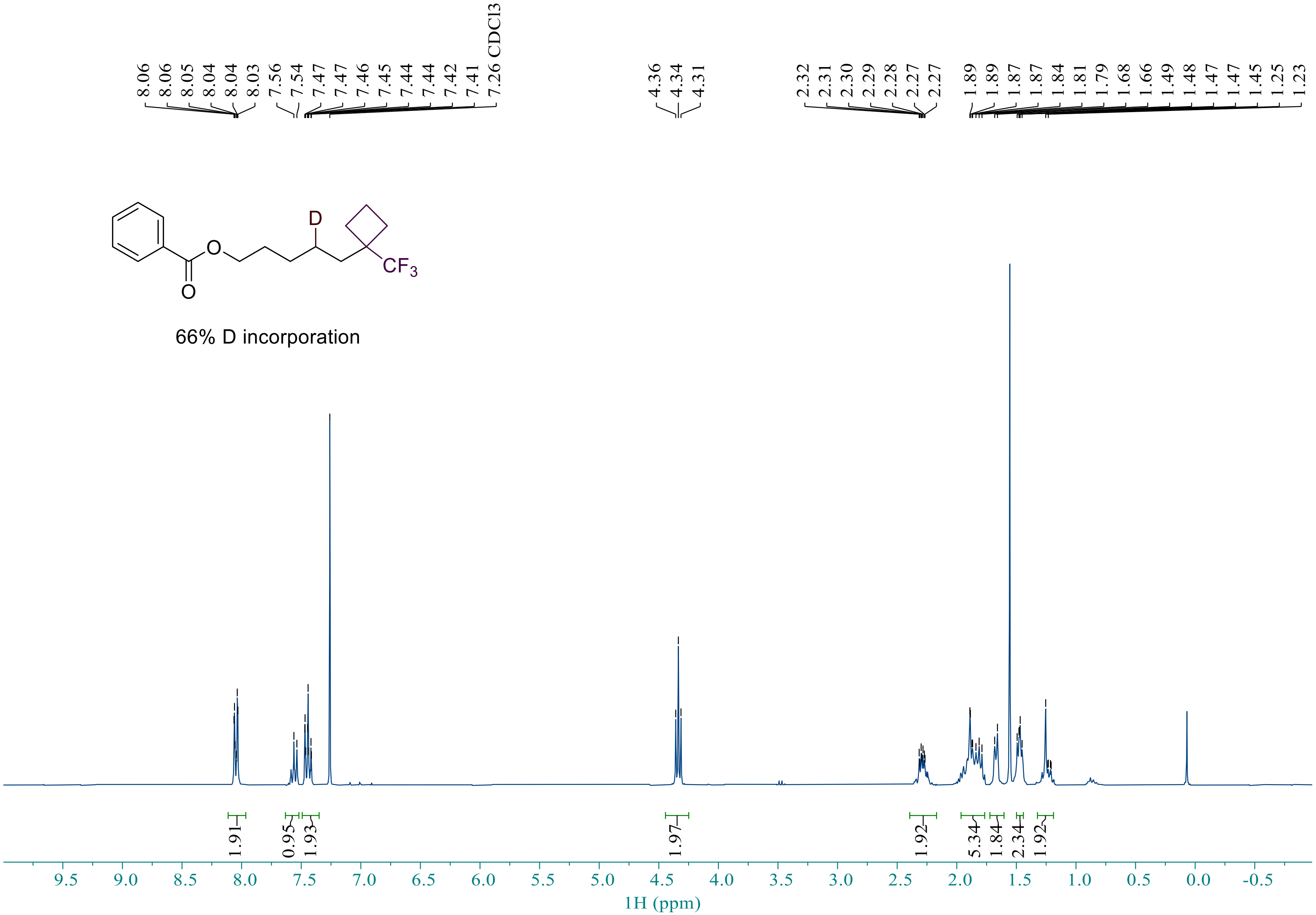

8.06
8.06
8.05
8.04
8.04
8.03
7.56
7.54
7.47
7.47
7.46
7.45
7.44
7.44
7.42
7.41
7.26 CDCl3
4.36
4.34
4.31
2.32
2.31
2.30
2.29
2.28
2.27
2.27
1.89
1.89
1.87
1.87
1.84
1.81
1.79
1.68
1.66
1.49
1.48
1.47
1.47
1.45
1.25
1.23
D
O
O
CF3
66% D incorporation
1.91
0.95
1.93
1.97
1.92
5.34
1.84
2.34
1.92
9.5
9.0
8.5
8.0
7.5
7.0
6.5
6.0
5.5
5.0
4.5
4.0
3.5
3.0
2.5
2.0
1.5
1.0
0.5
0.0
-0.5
1H (ppm)

^{13}C NMR of **39**

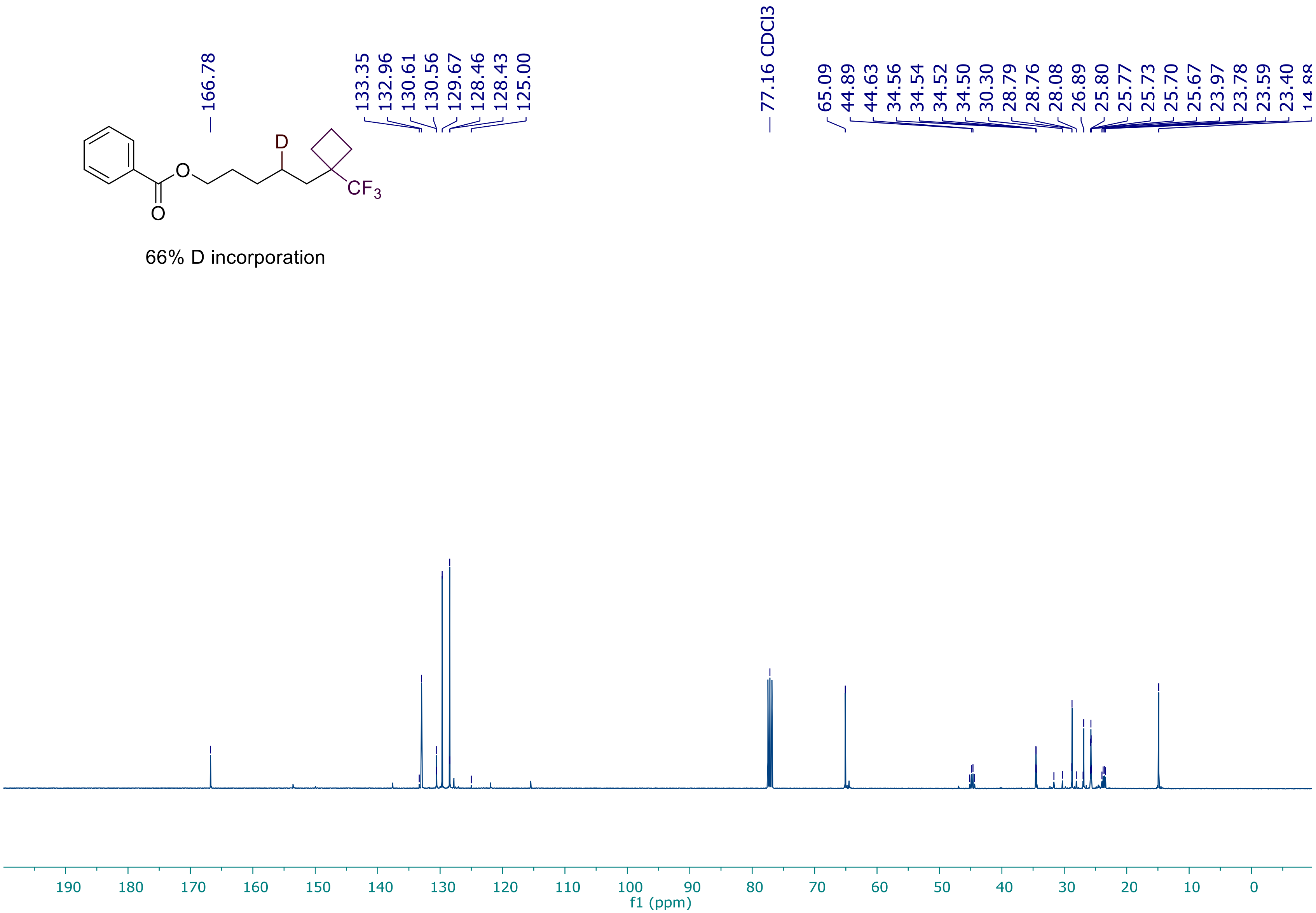
166.78
133.35
132.96
130.61
130.56
129.67
128.46
128.43
125.00
77.16 CDCl3
65.09
44.89
44.63
34.56
34.54
34.52
34.50
30.30
28.79
28.76
28.08
26.89
25.80
25.77
25.73
25.70
25.67
23.97
23.78
23.59
23.40
14.88
D
O
O
CF3
66% D incorporation
190
180
170
160
150
140
130
120
110
100
90
80
70
60
50
40
30
20
10
0
f1 (ppm)

^{19}F NMR of **39**

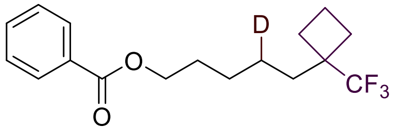

66% D incorporation

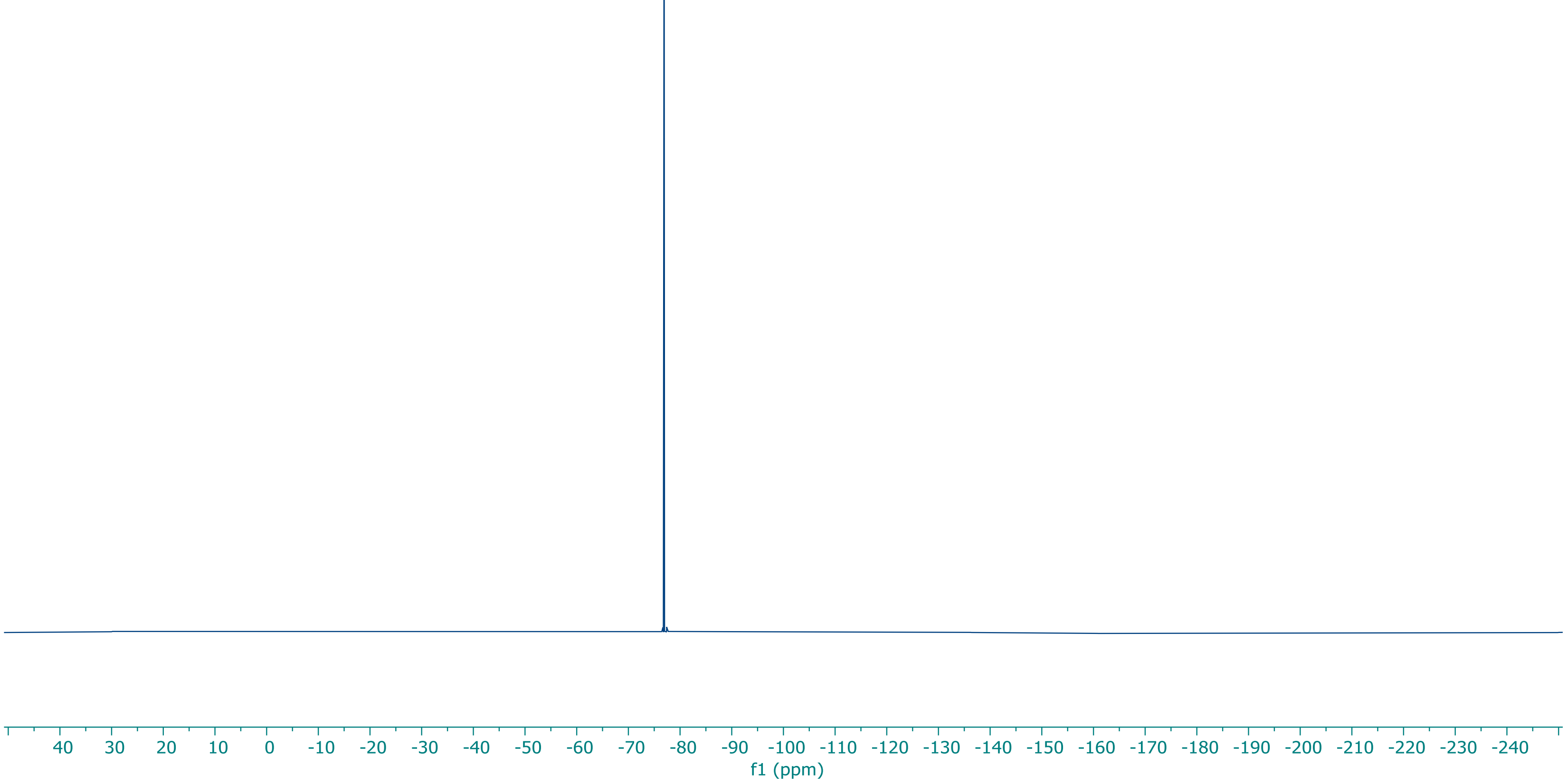

^{1}H NMR of **40**

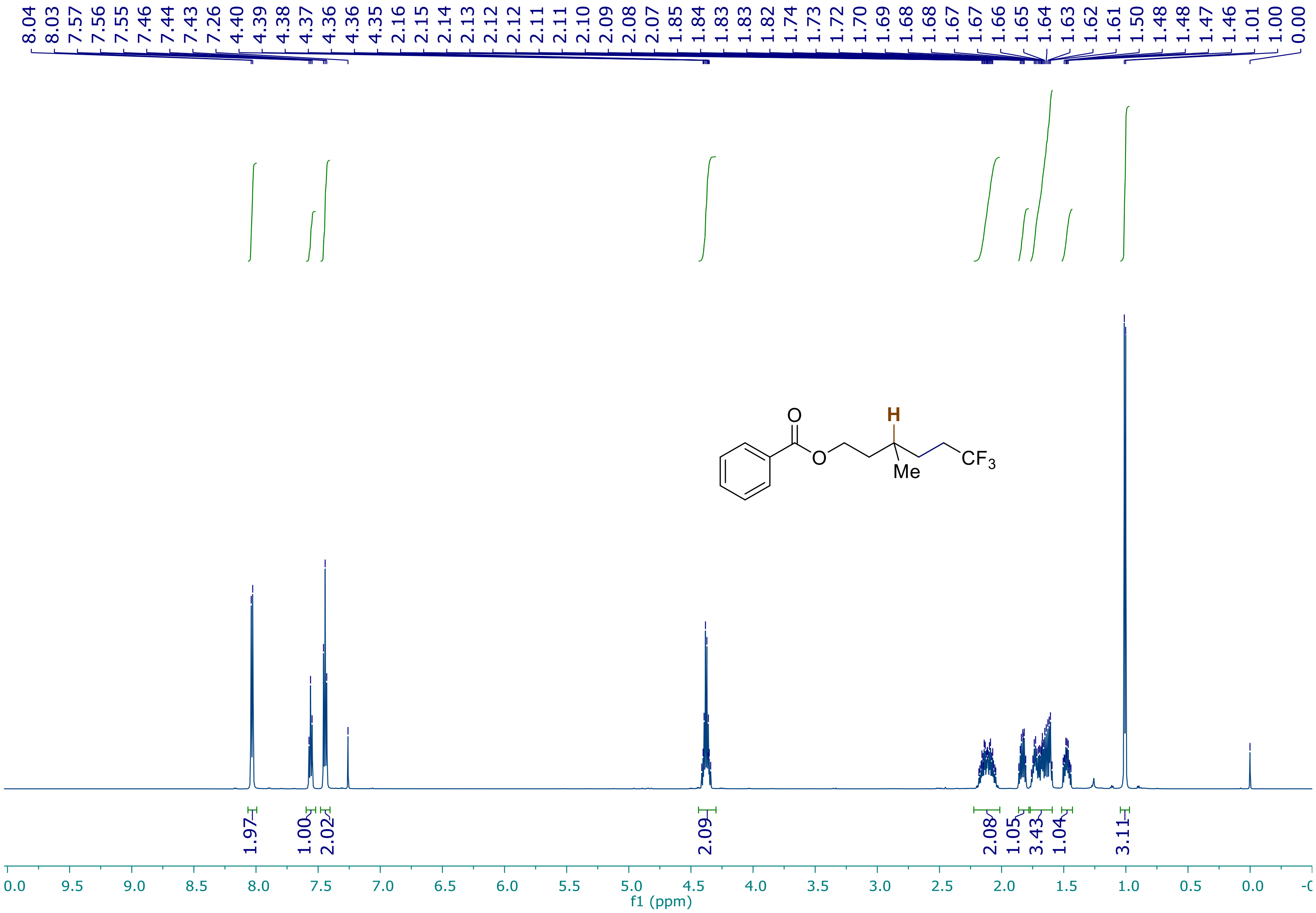
8.04 8.03 7.57 7.56 7.55 7.46 7.44 7.43 7.26 4.40 4.39 4.38 4.37 4.36 4.36 4.35 2.16 2.15 2.15 2.14 2.13 2.12 2.12 2.11 2.11 2.10 2.09 2.08 2.07 1.85 1.84 1.83 1.83 1.82 1.74 1.73 1.72 1.70 1.69 1.68 1.68 1.67 1.67 1.66 1.65 1.64 1.63 1.62 1.61 1.50 1.48 1.48 1.47 1.46 1.01 1.00 0.00
O
O
H
Me
CF3
1.97
1.00
2.02
2.09
2.08
1.05
3.43
1.04
3.11
f1 (ppm)

^{13}C NMR of **40**

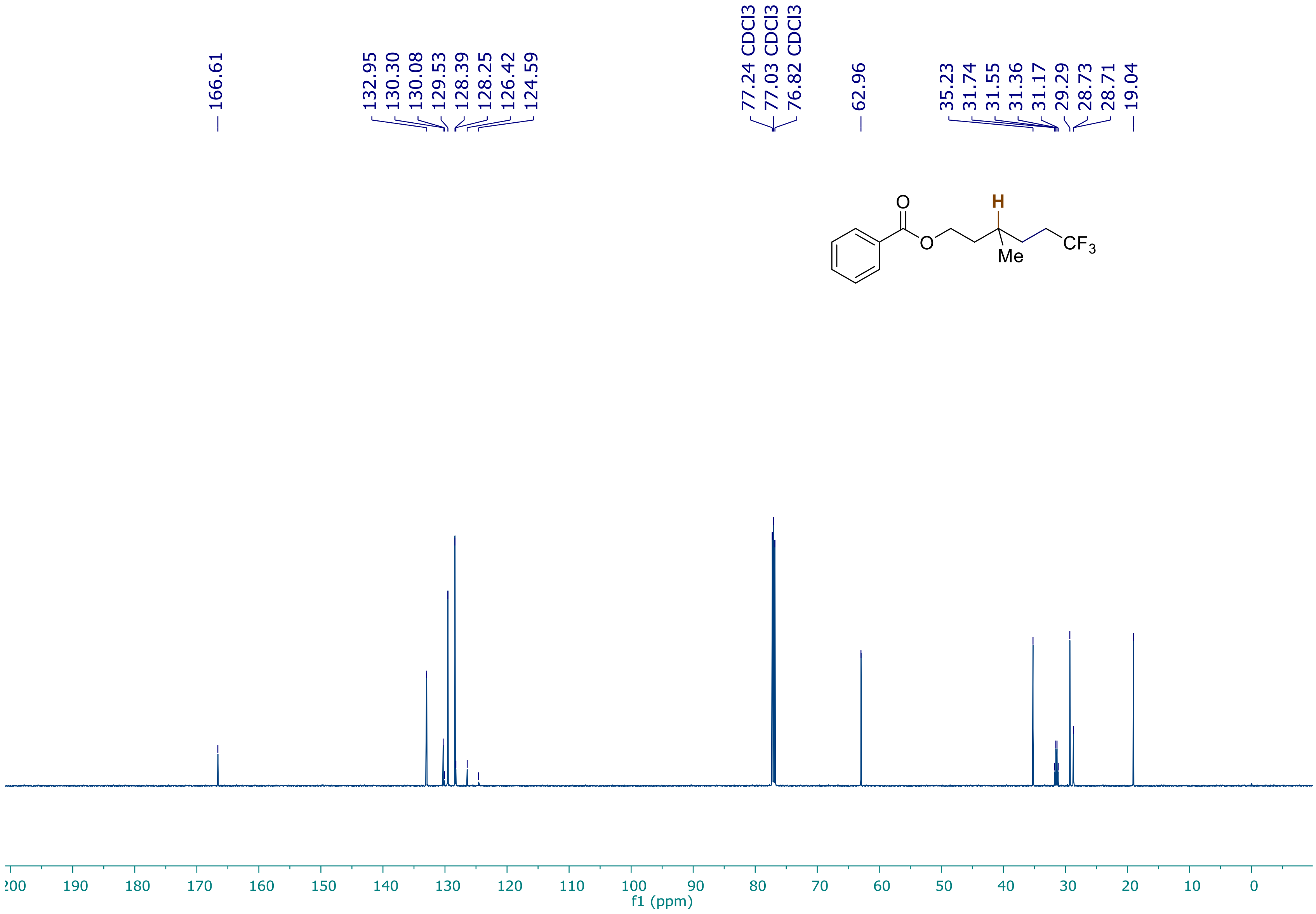

166.61
132.95
130.30
130.08
129.53
128.39
128.25
126.42
124.59
77.24 CDCl3
77.03 CDCl3
76.82 CDCl3
62.96
35.23
31.74
31.55
31.36
31.17
29.29
28.73
28.71
19.04
O
H
O
Me
CF3
200 190 180 170 160 150 140 130 120 110 100 90 80 70 60 50 40 30 20 10 0
f1 (ppm)

^{19}F NMR of **40**

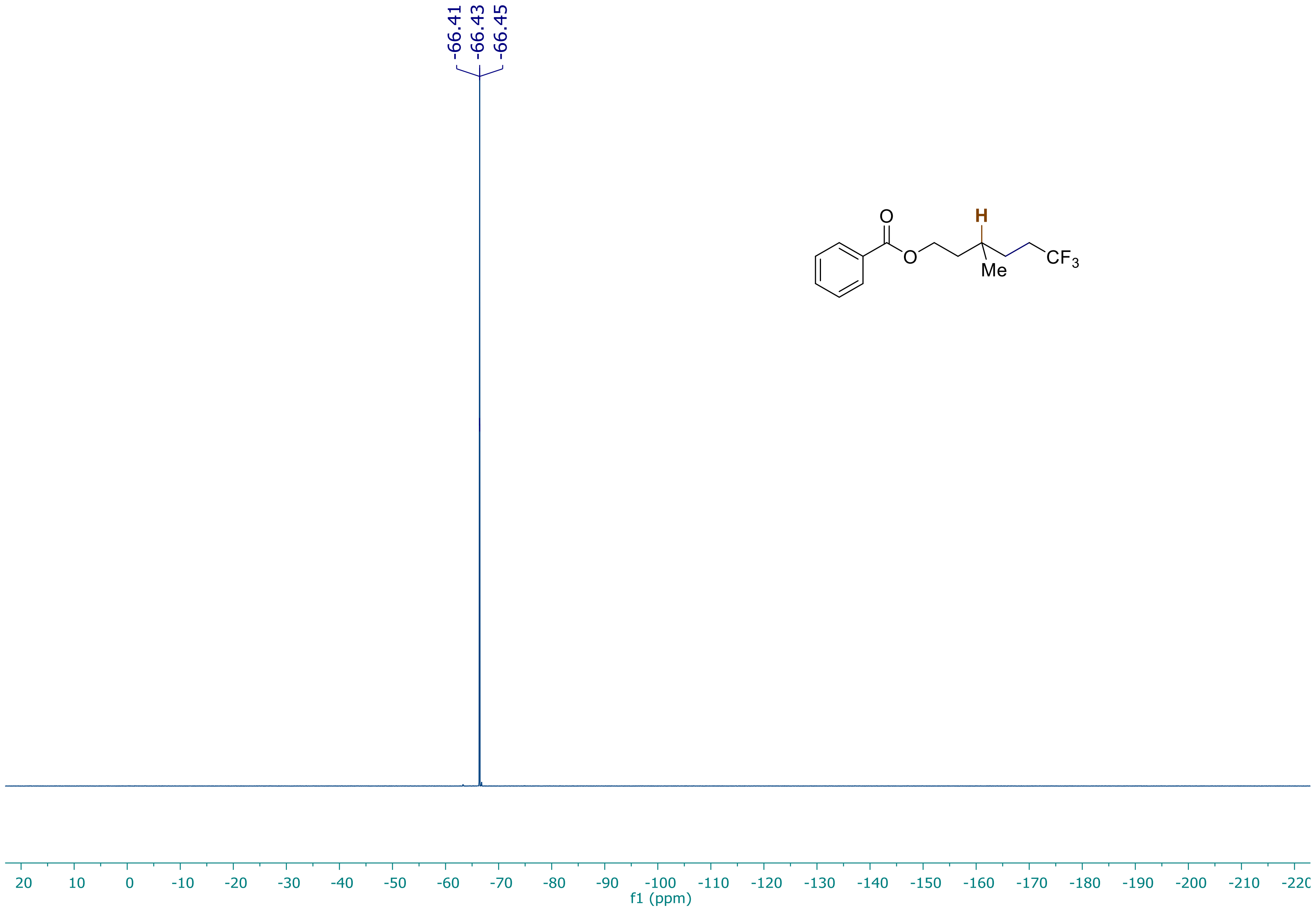

^{1}H NMR of **41**

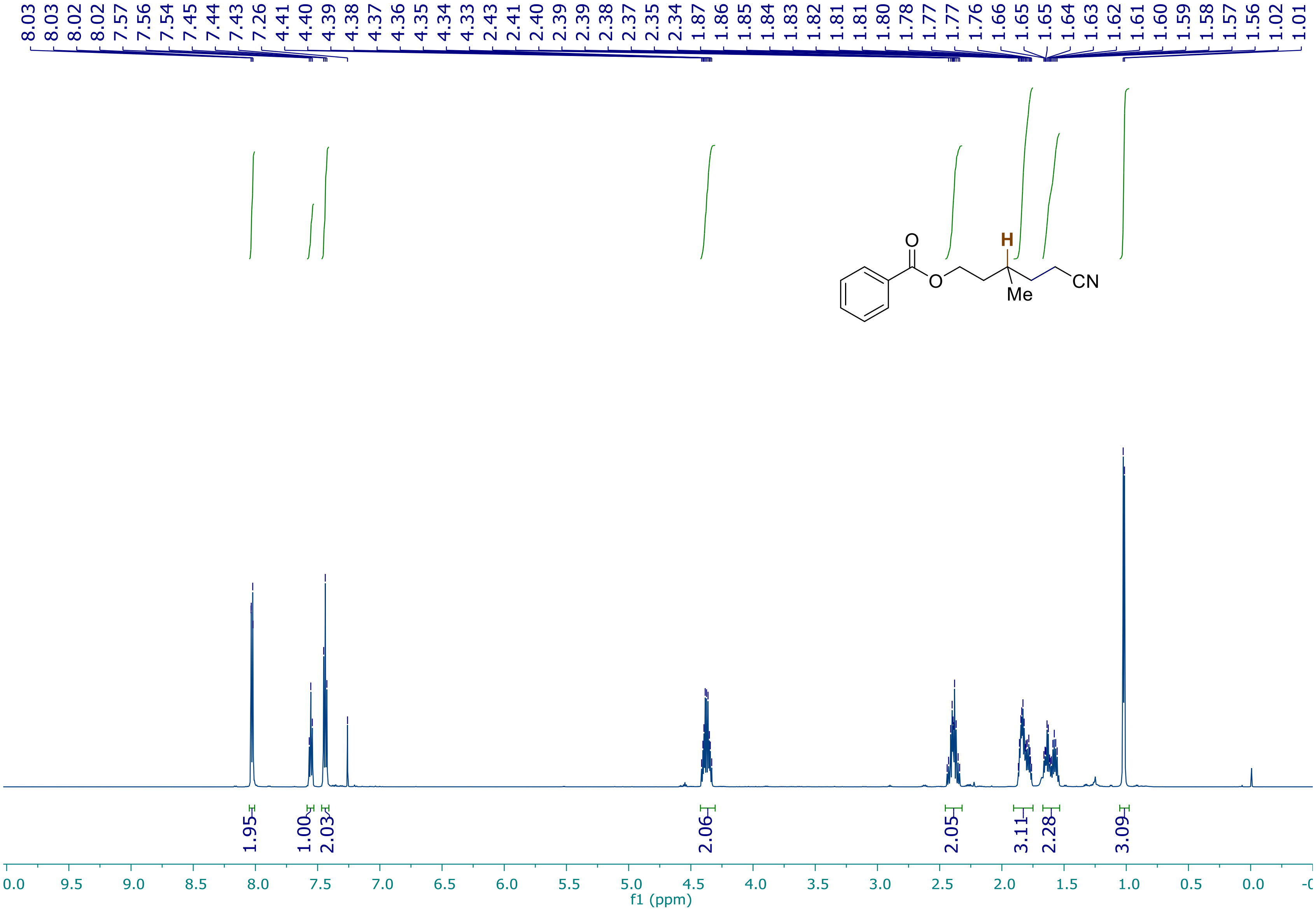

8.03 8.03 8.02 8.02 7.57 7.56 7.54 7.45 7.44 7.43 7.26 4.41 4.40 4.39 4.38 4.37 4.36 4.35 4.34 4.33 2.43 2.41 2.40 2.39 2.39 2.38 2.38 2.37 2.35 2.34 1.87 1.86 1.85 1.84 1.83 1.82 1.81 1.81 1.80 1.78 1.77 1.77 1.76 1.66 1.65 1.65 1.64 1.63 1.62 1.61 1.60 1.59 1.58 1.57 1.56 1.02 1.01
O
H
O
Me
CN
1.95
1.00
2.03
2.06
2.05
3.11
2.28
3.09
f1 (ppm)

^{13}C NMR of **41**

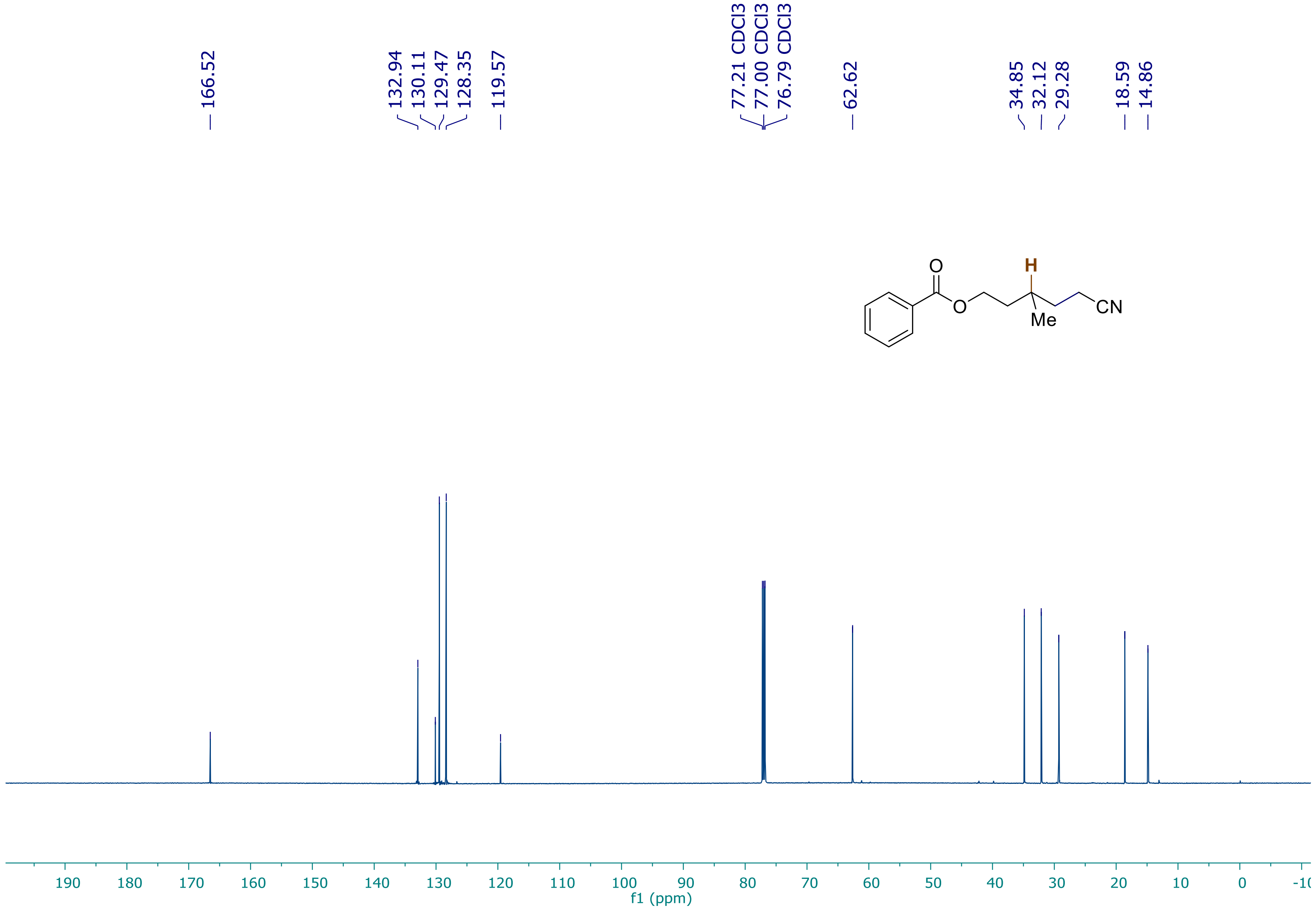
166.52
132.94
130.11
129.47
128.35
119.57
77.21 CDCl3
77.00 CDCl3
76.79 CDCl3
62.62
34.85
32.12
29.28
18.59
14.86
O
O
H
Me
CN
190
180
170
160
150
140
130
120
110
100
90
80
70
60
50
40
30
20
10
0
f1 (ppm)

^{1}H NMR of **42**

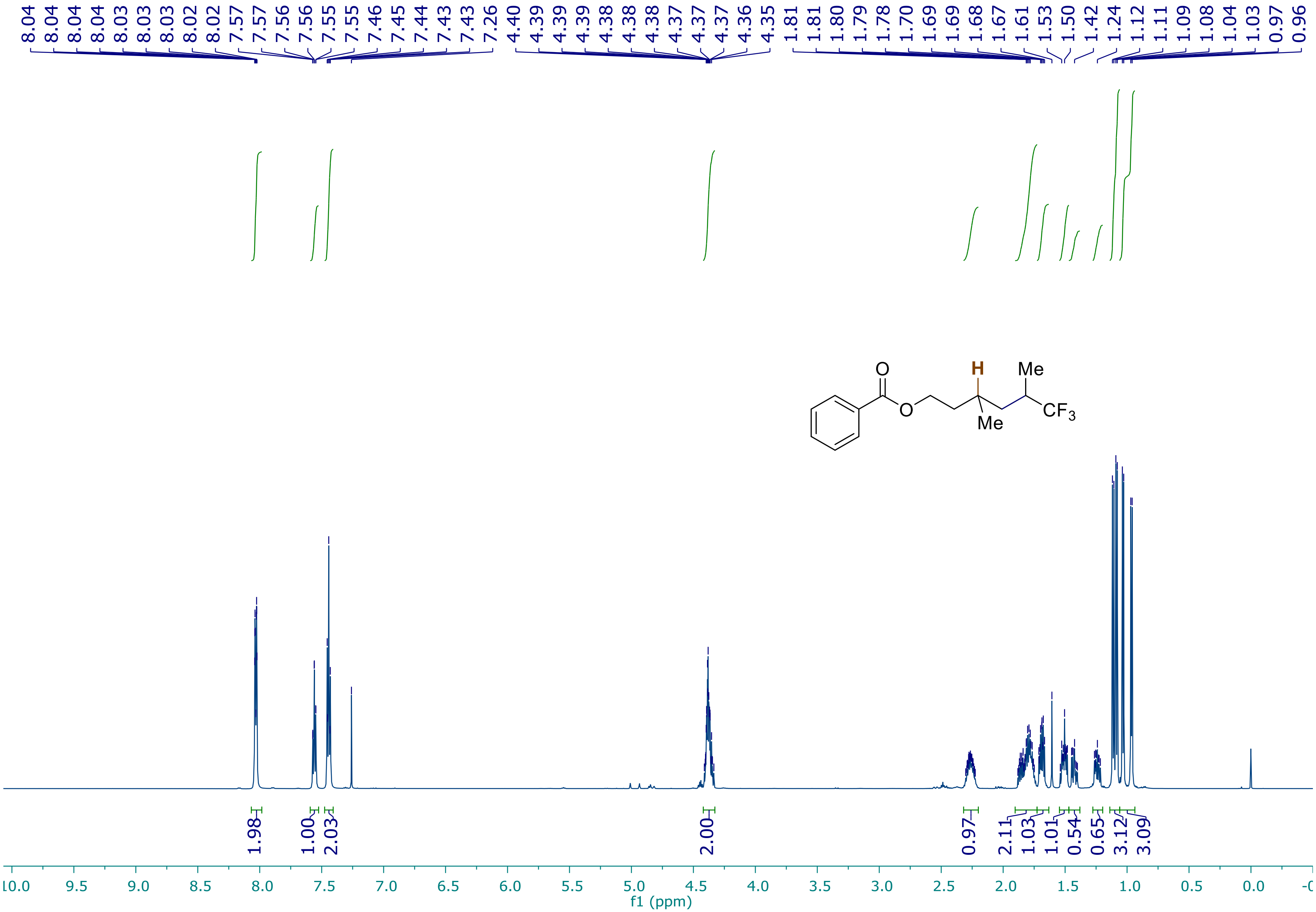

8.04 8.04 8.04 8.04 8.03 8.03 8.03 8.02 8.02 7.57 7.57 7.56 7.56 7.55 7.55 7.46 7.45 7.44 7.43 7.43 7.26 4.40 4.39 4.39 4.39 4.38 4.38 4.38 4.37 4.37 4.37 4.36 4.35 1.81 1.81 1.80 1.79 1.78 1.70 1.69 1.69 1.68 1.67 1.61 1.53 1.50 1.42 1.24 1.12 1.11 1.09 1.08 1.04 1.03 0.97 0.96
O
O
H
Me
Me
CF3
1.98
1.00
2.03
2.00
0.97
2.11
1.03
1.01
0.54
0.65
3.12
3.09
10.0 9.5 9.0 8.5 8.0 7.5 7.0 6.5 6.0 5.5 5.0 4.5 4.0 3.5 3.0 2.5 2.0 1.5 1.0 0.5 0.0
f1 (ppm)

^{13}C NMR of **42**

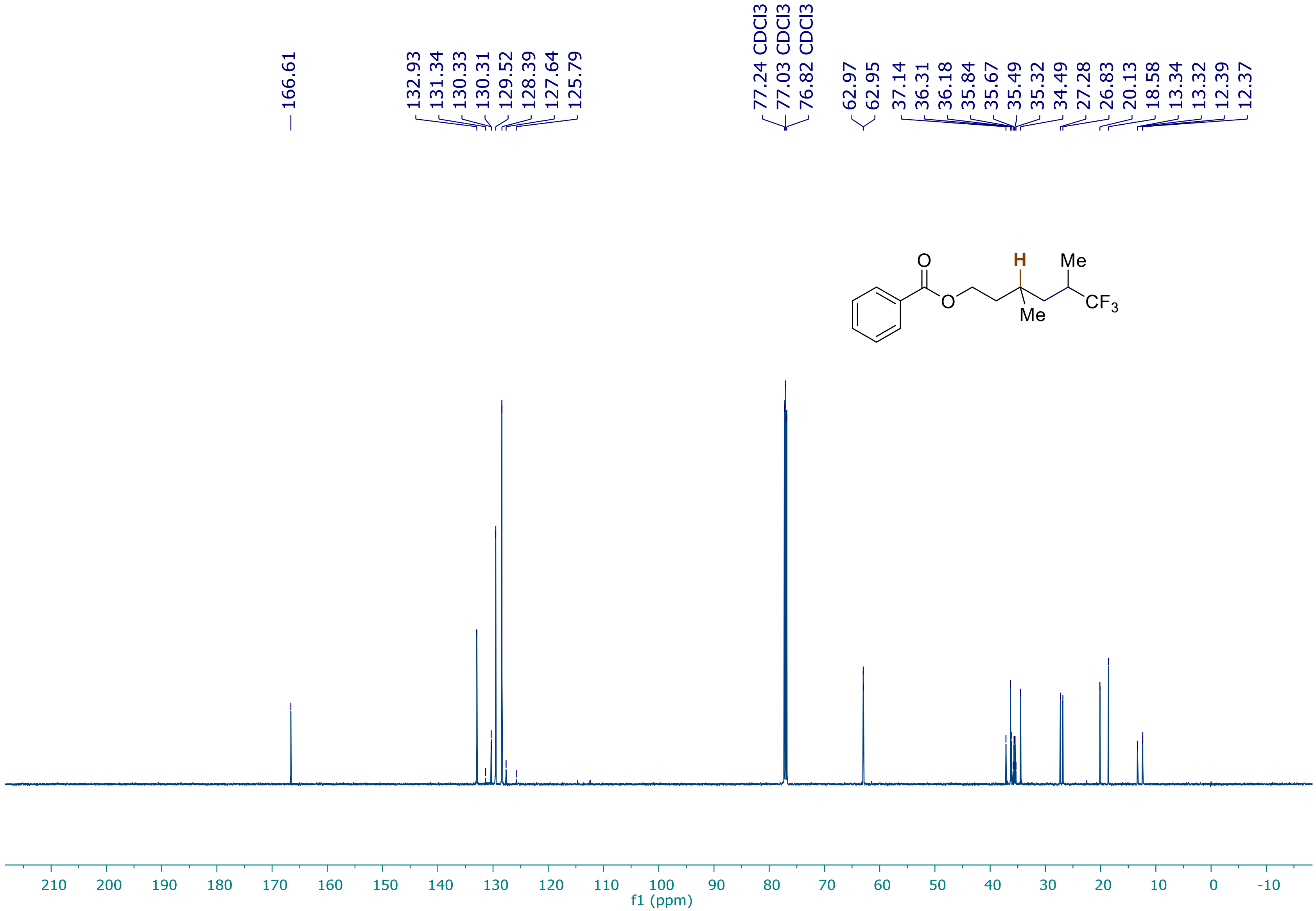

^{19}F NMR of **42**

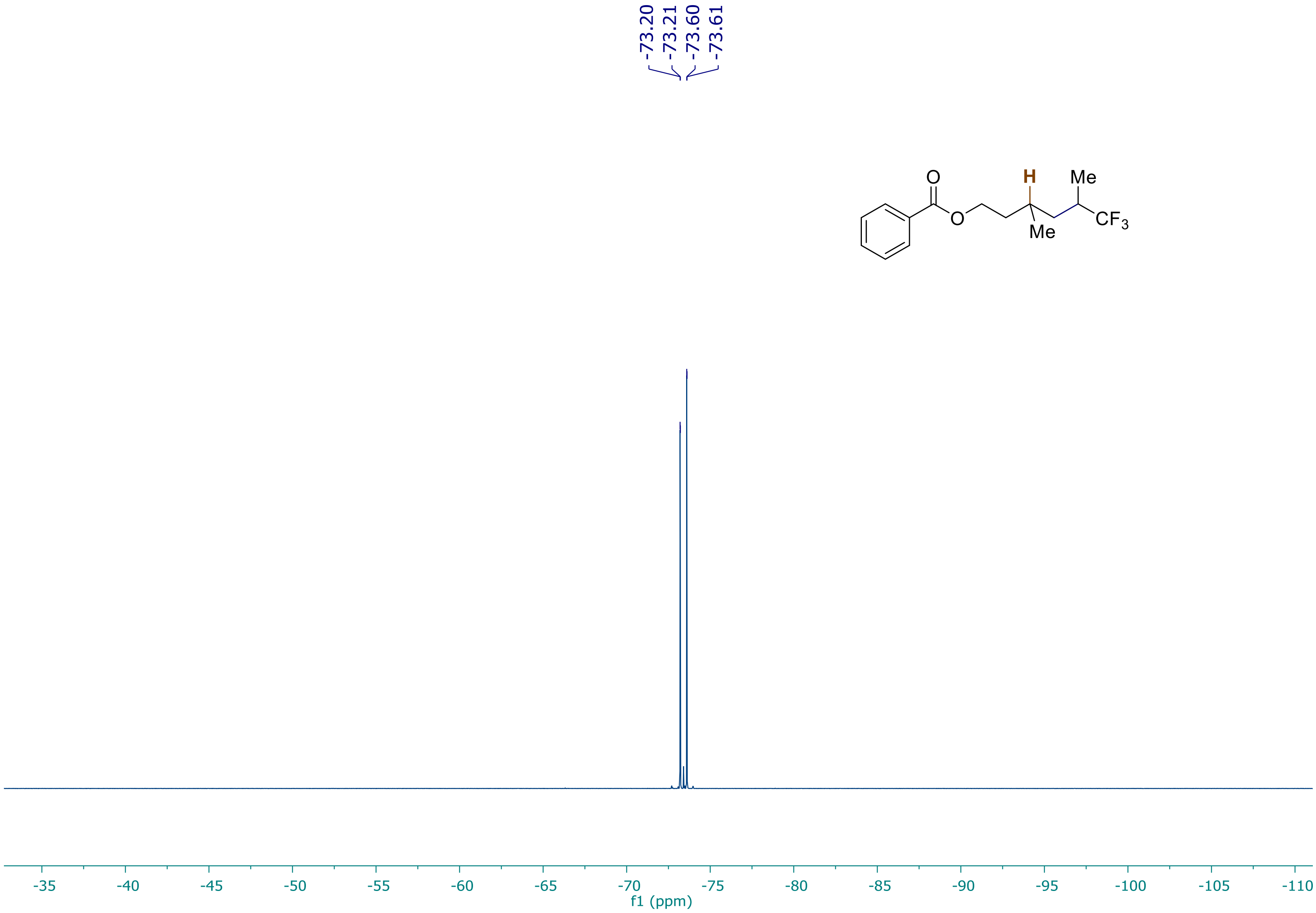

^{1}H NMR of **43**

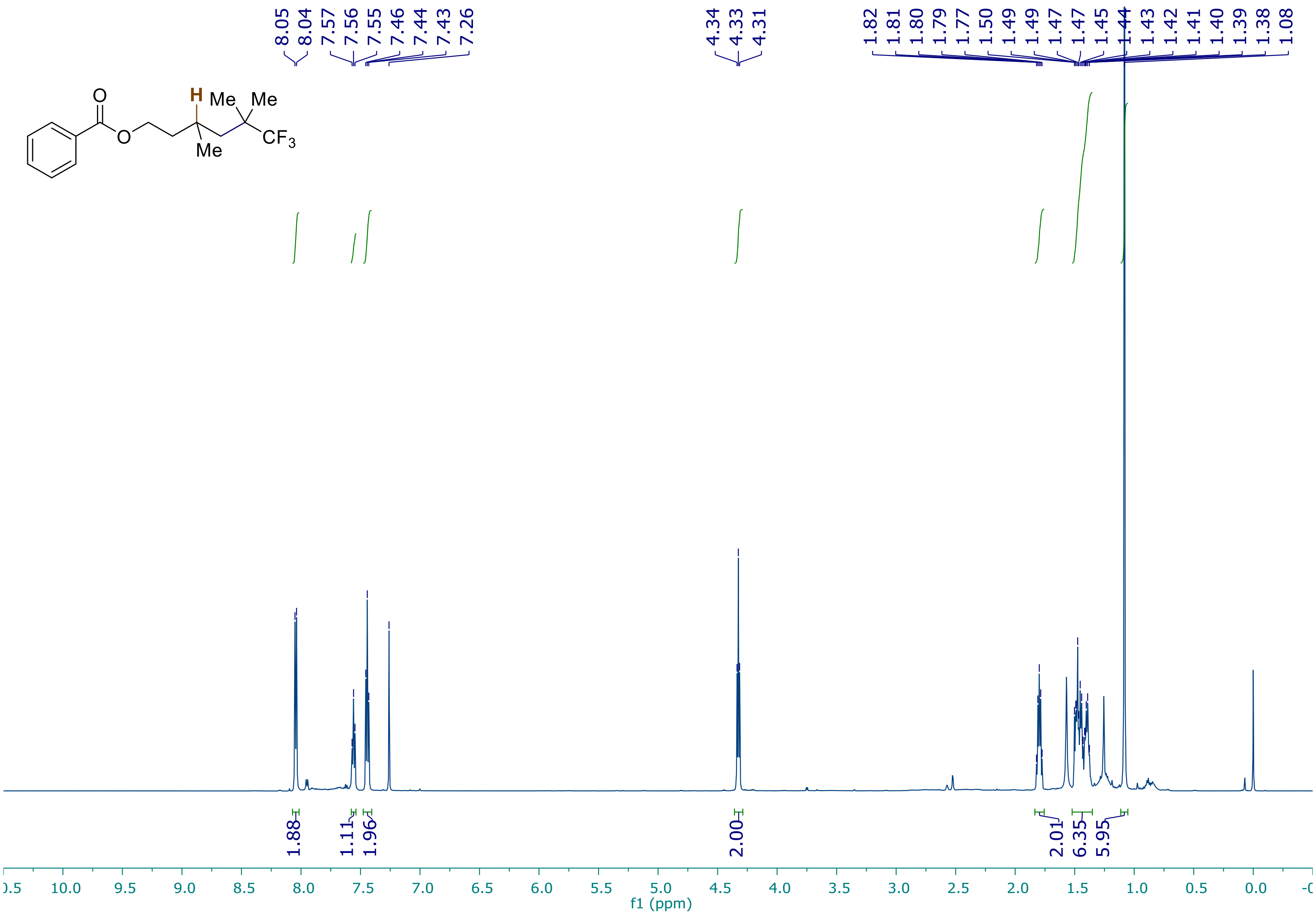

8.05
8.04
7.57
7.56
7.55
7.46
7.44
7.43
7.26
4.34
4.33
4.31
1.82
1.81
1.80
1.79
1.77
1.50
1.49
1.49
1.47
1.47
1.45
1.44
1.43
1.42
1.41
1.40
1.39
1.38
1.08
O
O
H
Me
Me
Me
CF3
1.88
1.11
1.96
2.00
2.01
6.35
5.95
f1 (ppm)

^{13}C NMR of **43**

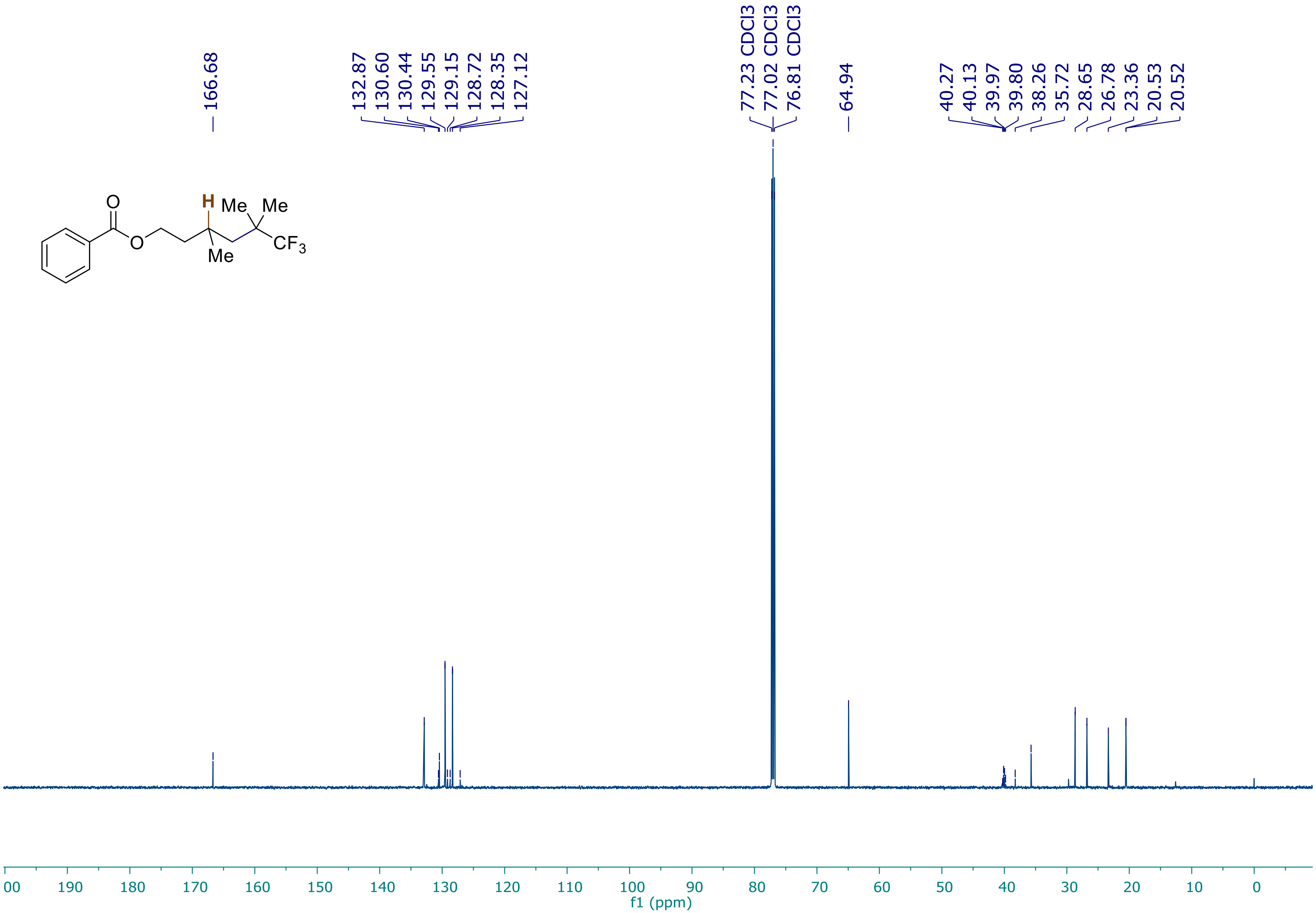

^{19}F NMR of **43**

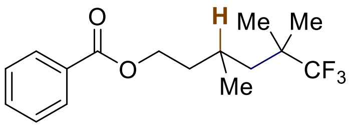

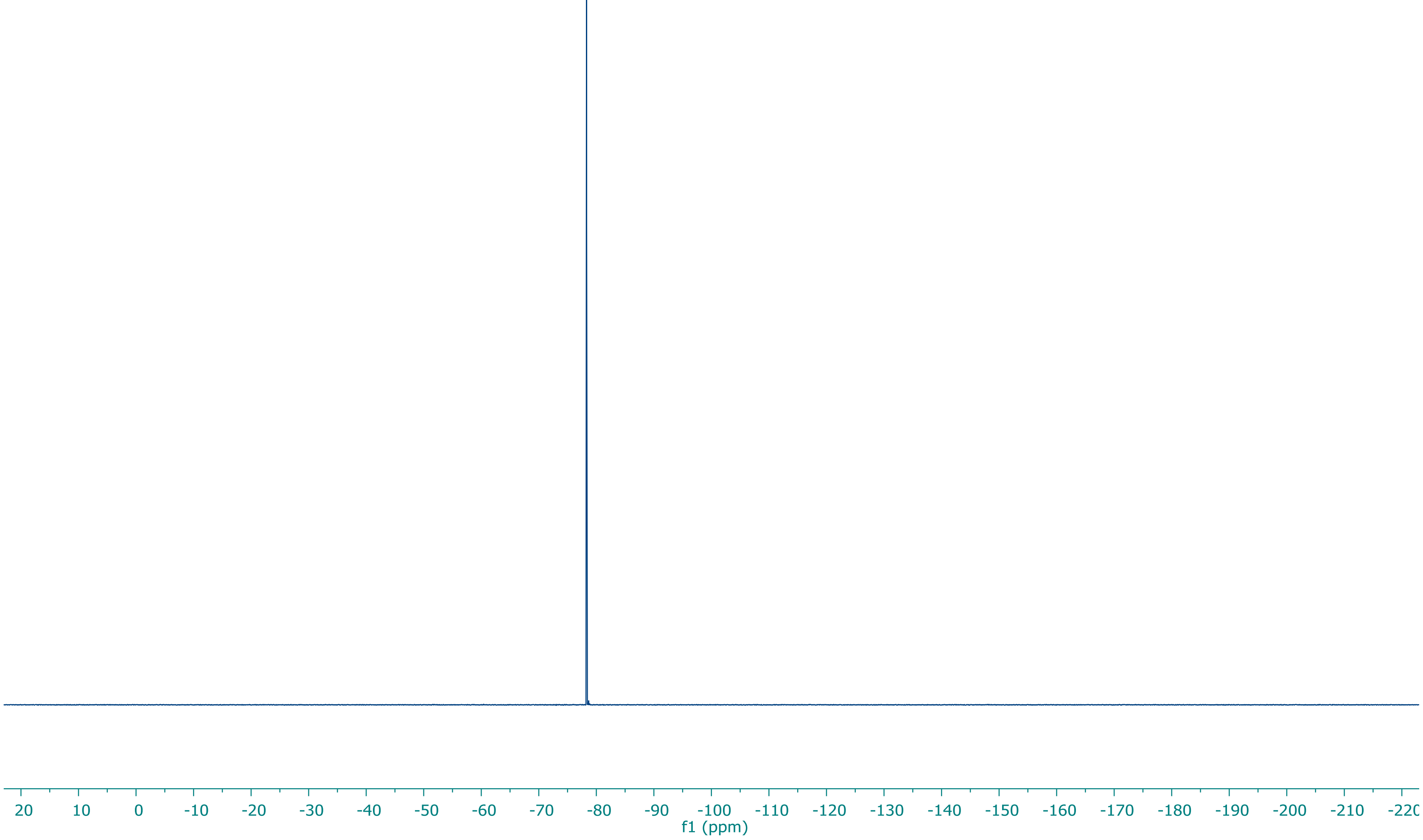
-78.30
20
10
0
-10
-20
-30
-40
-50
-60
-70
-80
-90
-100
-110
-120
-130
-140
-150
-160
-170
-180
-190
-200
-210
-220
f1 (ppm)

^{1}H NMR of **46**

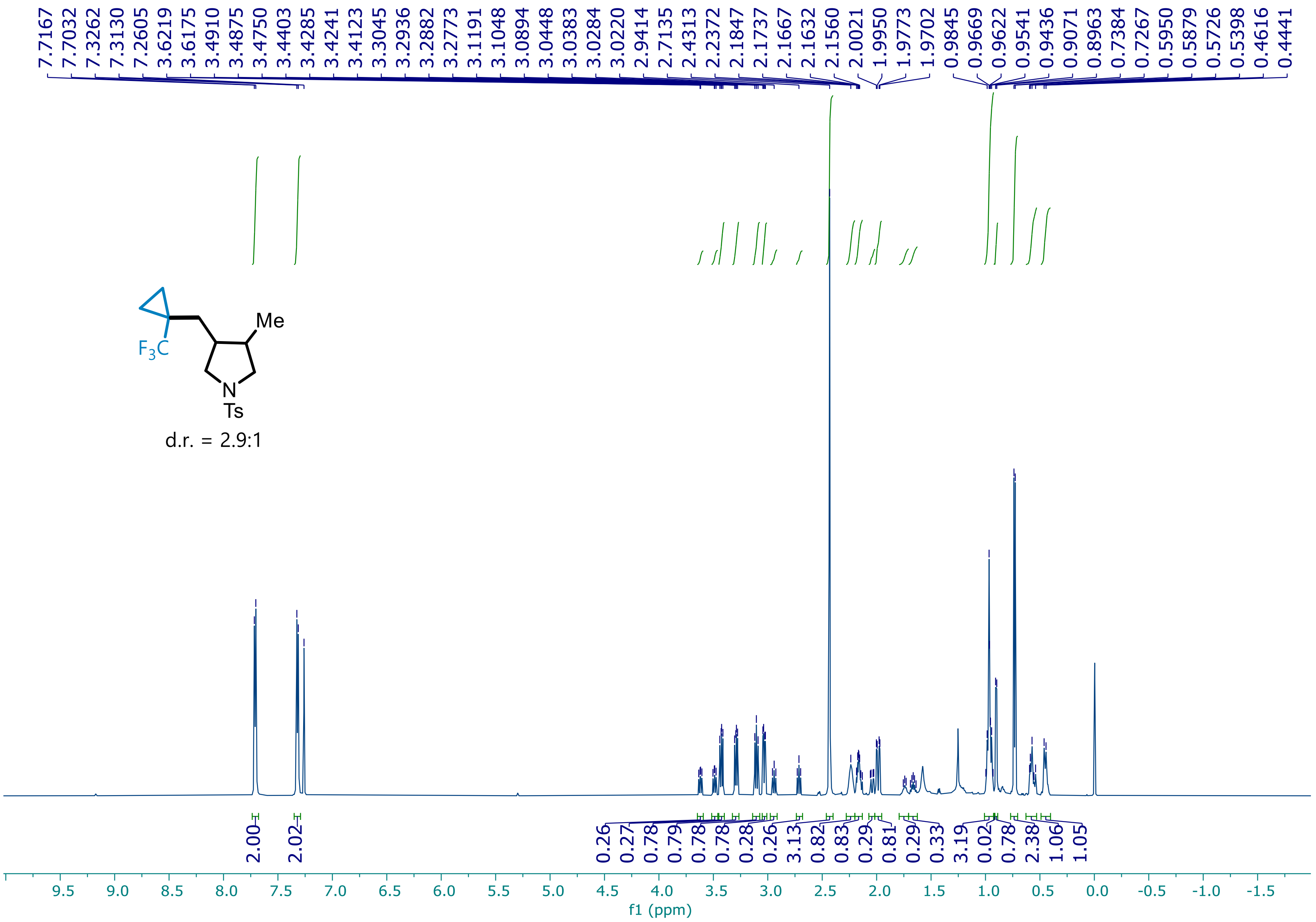
7.7167
7.7032
7.3262
7.3130
7.2605
3.6219
3.6175
3.4910
3.4875
3.4750
3.4403
3.4285
3.4241
3.4123
3.3045
3.2936
3.2882
3.2773
3.1191
3.1048
3.0894
3.0448
3.0383
3.0284
3.0220
2.9414
2.7135
2.4313
2.2372
2.1847
2.1737
2.1667
2.1632
2.1560
2.0021
1.9950
1.9773
1.9702
0.9845
0.9669
0.9622
0.9541
0.9436
0.9071
0.8963
0.7384
0.7267
0.5950
0.5879
0.5726
0.5398
0.4616
0.4441
Me
F_3C
N
Ts
d.r. = 2.9:1
2.00
2.02
0.26
0.27
0.78
0.79
0.78
0.78
0.28
0.26
3.13
0.82
0.83
0.29
0.81
0.29
0.33
3.19
0.02
0.78
2.38
1.06
1.05
f1 (ppm)

^{13}C NMR of **46**

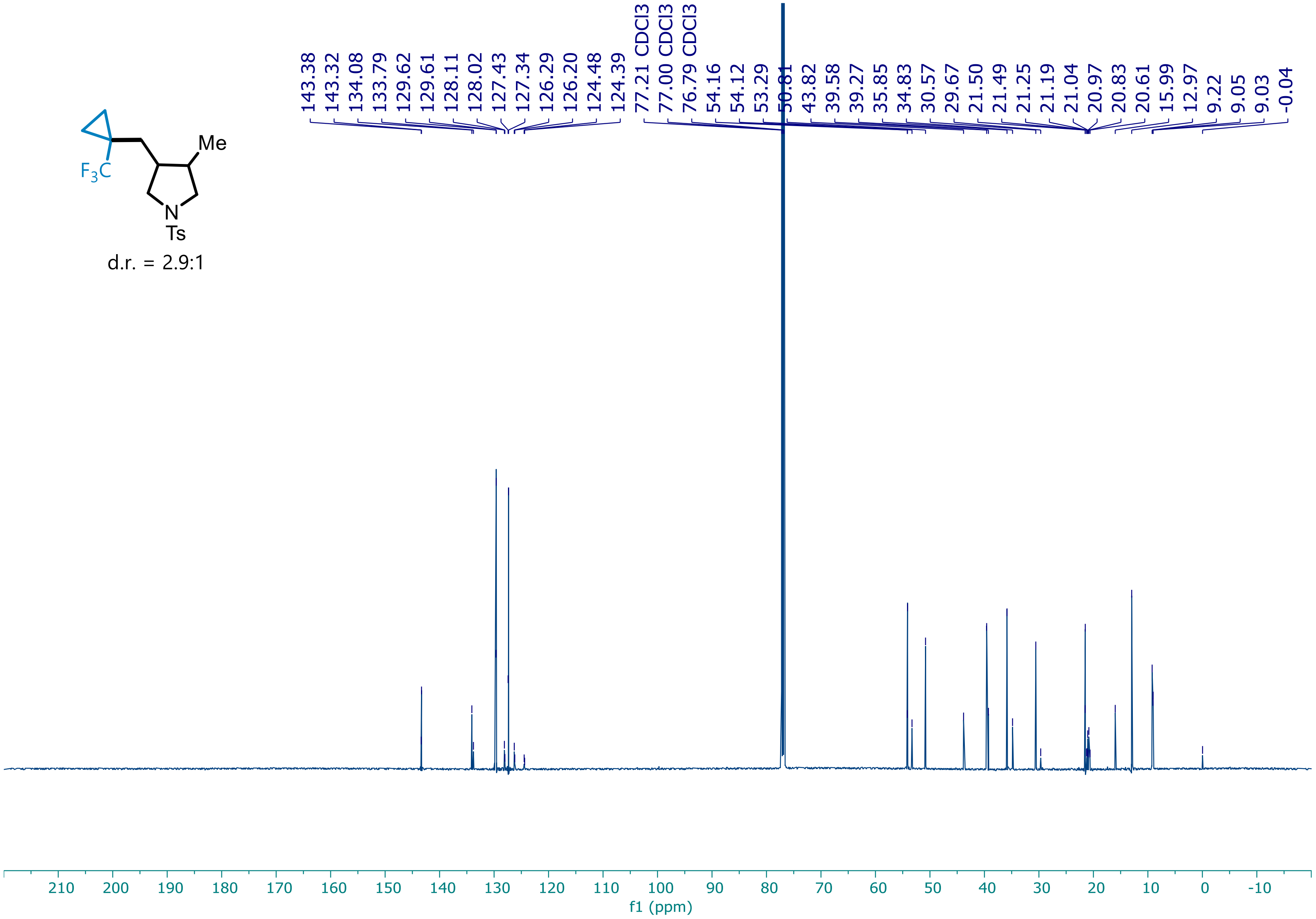

143.38
143.32
134.08
133.79
129.62
129.61
128.11
128.02
127.43
127.34
126.29
126.20
124.48
124.39
77.21 CDCl3
77.00 CDCl3
76.79 CDCl3
54.16
54.12
53.29
50.81
43.82
39.58
39.27
35.85
34.83
30.57
29.67
21.50
21.49
21.25
21.19
21.04
20.97
20.83
20.61
15.99
12.97
9.22
9.05
9.03
-0.04
Me
F3C
N
Ts
d.r. = 2.9:1
210
200
190
180
170
160
150
140
130
120
110
100
90
80
70
60
50
40
30
20
10
0
-10
f1 (ppm)

^{19}F NMR of **46**

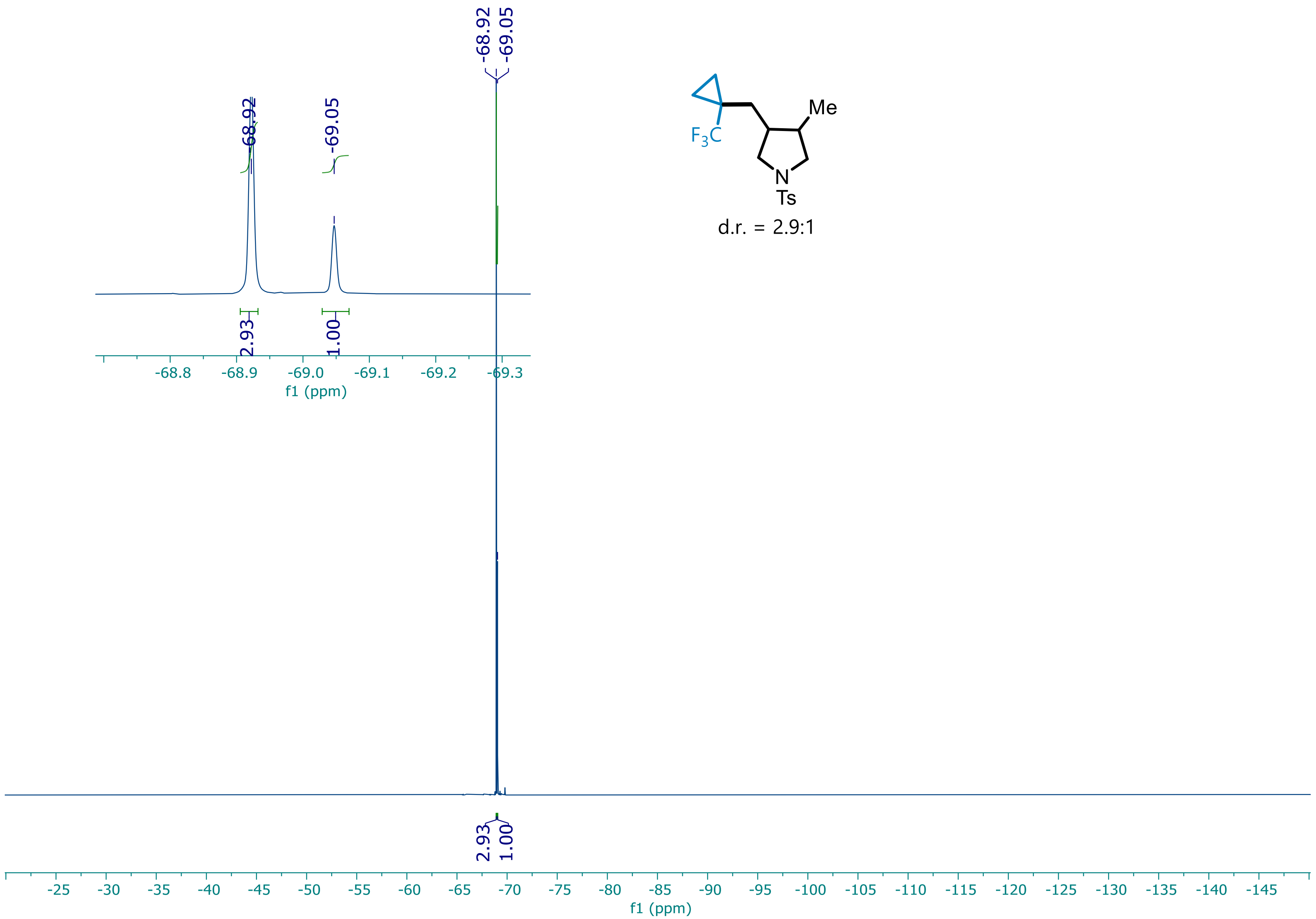

-68.92
-69.05
2.93
1.00
-68.8
-68.9
-69.0
-69.1
-69.2
-69.3
f1 (ppm)
Me
F3C
N
Ts
d.r. = 2.9:1
-25 -30 -35 -40 -45 -50 -55 -60 -65 -70 -75 -80 -85 -90 -95 -100 -105 -110 -115 -120 -125 -130 -135 -140 -145
f1 (ppm)

^{1}H NMR of **47 (in MeCN-d3)**

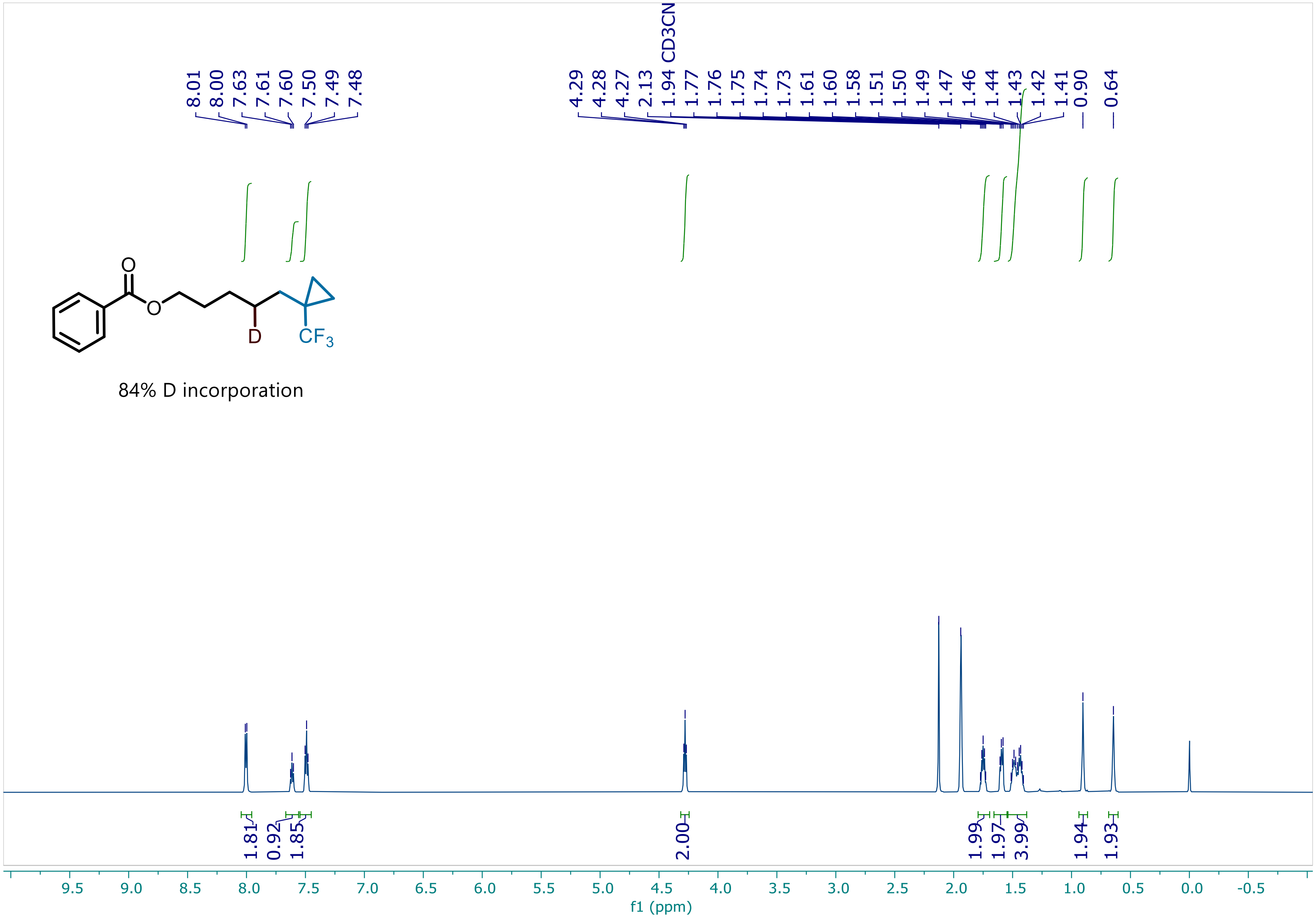

^{13}C NMR of **47**

019737.11.fid
C13CPD_STD CDCl3 /opt service 16

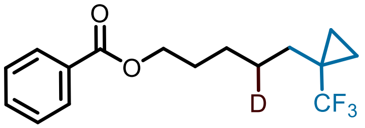

84% D incorporation

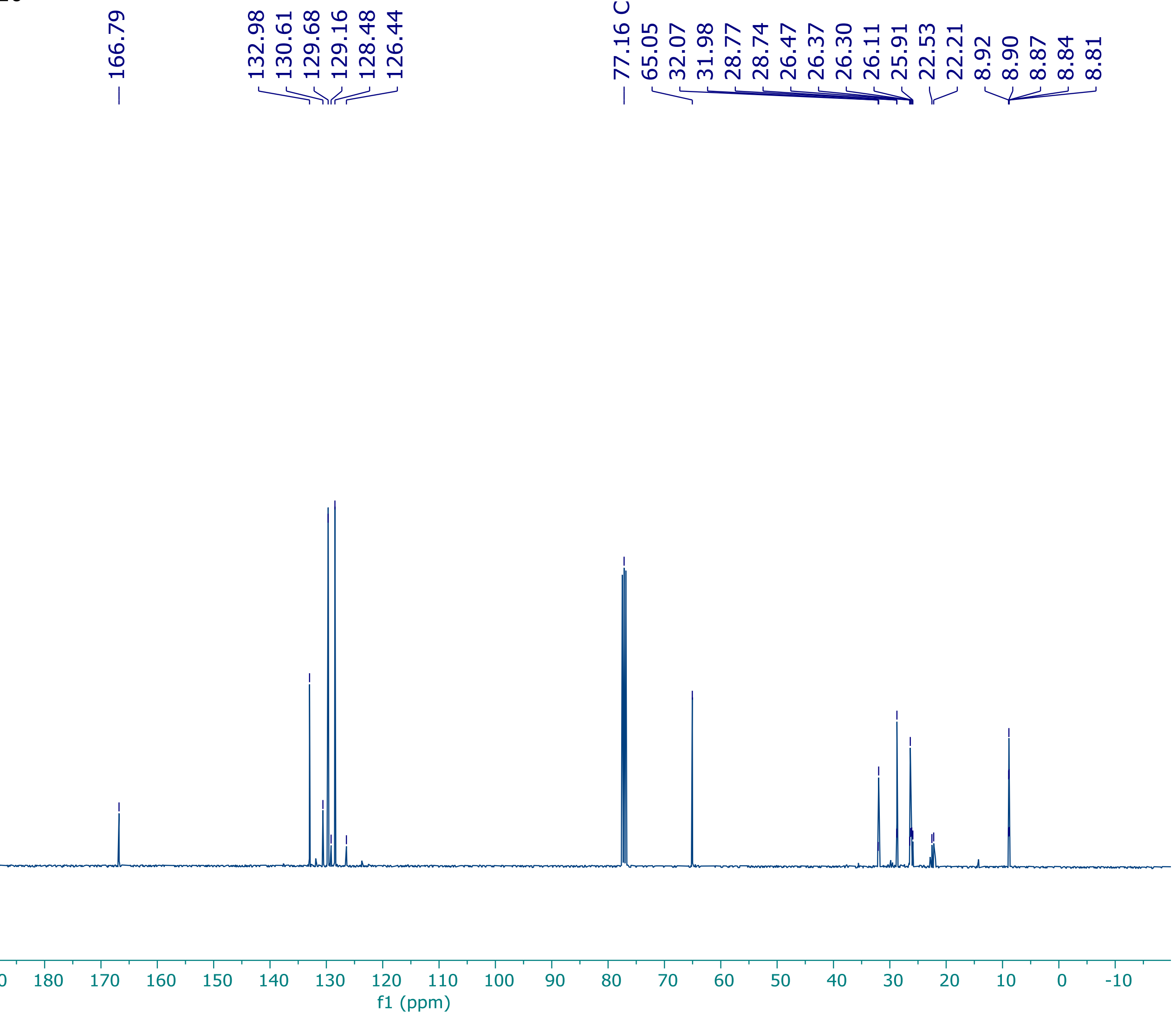

^{19}F NMR of **47**

019737.12.fid
F19_STD CDCl3 /opt service 16

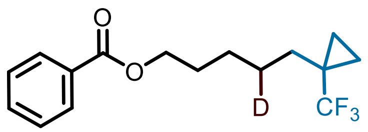

84% D incorporation

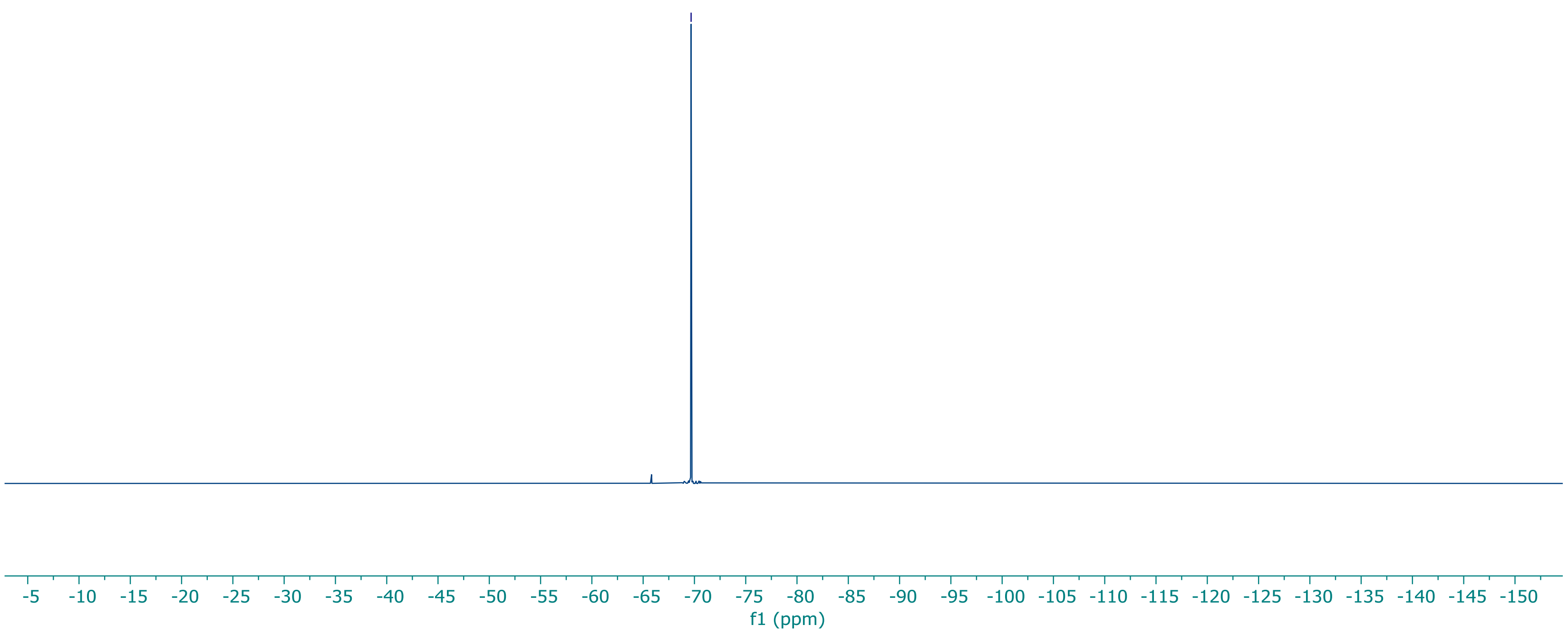

Supplement: Supplementary file 1 — Supporting Information [file ANIE-64-e202508377-s001.pdf]
